# Supplementary material for: Spatially selective delivery of living magnetic microrobots through torque-focusing
Source: Nat Commun. 2024 Mar 9;15:2160. doi: 10.1038/s41467-024-46407-4 (PMC10924878; doi:10.1038/s41467-024-46407-4)
Supplement: Supplementary file 1 — Supplementary Information [file 41467_2024_46407_MOESM1_ESM.pdf]

## Supplementary Material

# Spatially selective delivery of living magnetic microrobots through torque-focusing

Nima Mirkhani[1], Michael G. Christiansen[1], Tinotenda Gwisai[1], Stefano Menghini[1], Simone Schuerle[1,*]

[1]Institute for Translational Medicine, Department of Health Sciences and Technology, ETH Zurich, CH-8092 Zurich, Switzerland

*Email: simone.schuerle@hest.ethz.ch

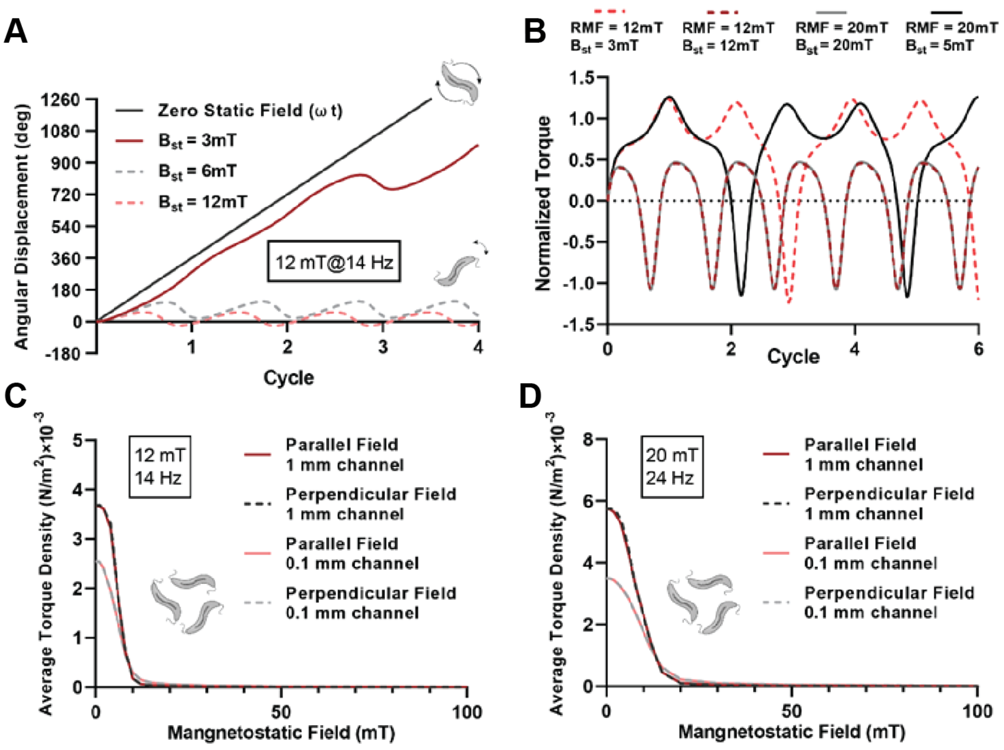


**Fig. S1: Selection field requirements for suppression of torque-driven transport.** (**A**) Angular displacement of individual microrobots exposed to different magnitudes of the static gating field with RMF 12 mT and 14 Hz. (**B**) Applied magnetic torque on a single microrobot under various RMF and gating field values. Average torque drops to almost zero reflecting the transition from rotation with back-and-forth motion to symmetric oscillation. (**C, D**) Decay of the average torque density across channels of varying widths as a function of differently oriented magnetostatic fields under RMF of (**C**) 12 mT and 14 Hz and (**D**) 20 mT and 24 Hz. Graphical elements (bacteria) have been adapted from Schuerle *et al.,Sci. Adv.* 2019; 5:eaav480. Reprinted with permission from AAAS.

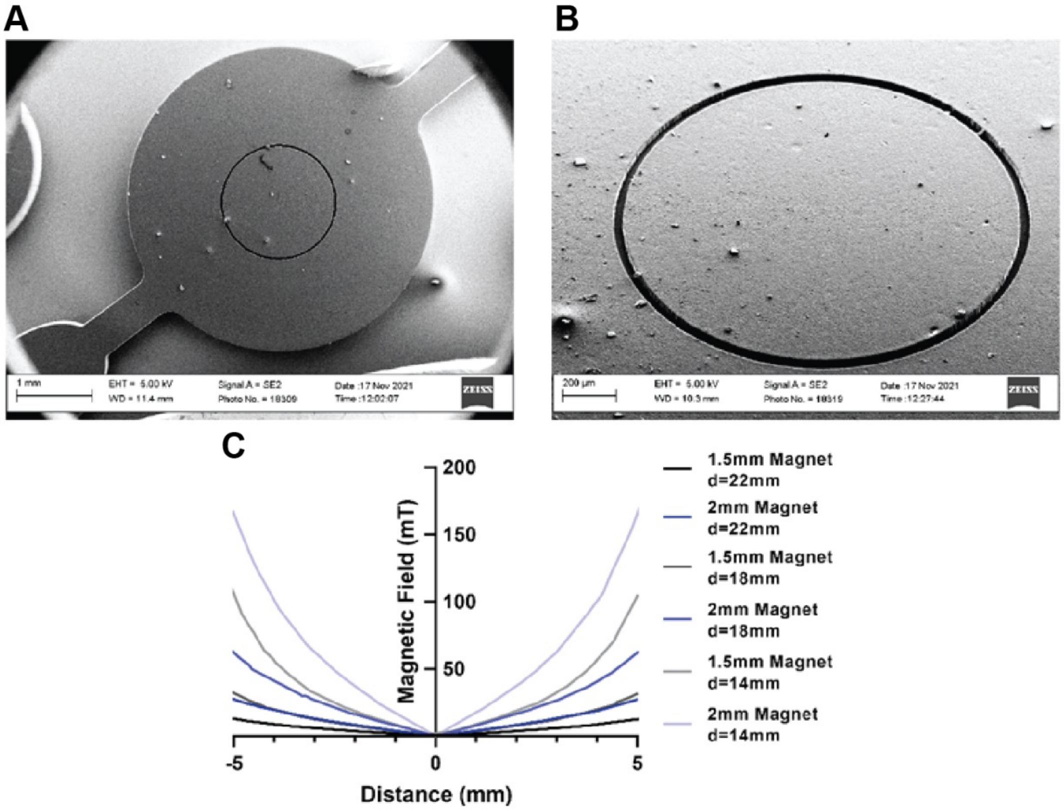


**Fig. S2: Design and characterization of the in vitro platform to study selective actuation of MTB and selective transport of NPs.** (**A**) SEM micrograph of the microfluidic master mold from top for the device shown in Fig 2A of the main text. (**B**) SEM micrograph of the microfabricated ridge in a ring shape serving as the contact line to pin viscous collagen upon filling into the chambers. (**C**) Magnetic field profiles throughout the working volume of the device layout shown in Fig 2B of the main text, as predicted by finite element modelling assuming two differently sized small blocks of NdFeB magnets at different distances. Small magnets at *d*=18 mm and large magnets at *d*=22 mm provide adequate resolution for selective actuation of target well.

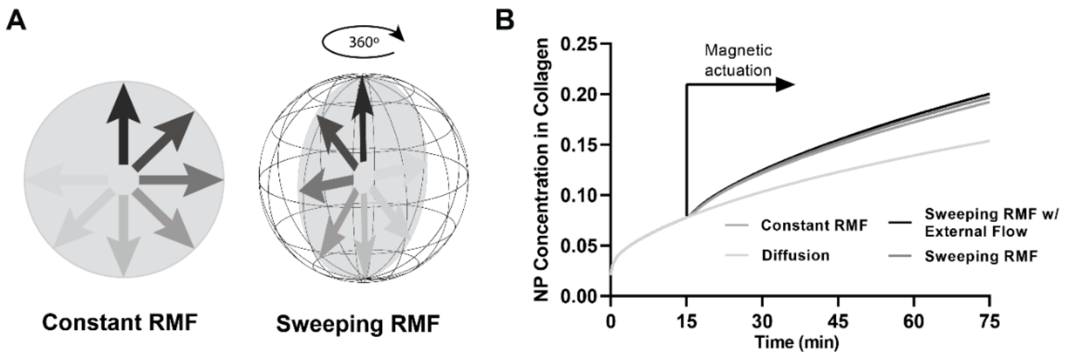


**Fig. S3**: **Simulated magnetically enhanced selective transport of NPs into collagen gels.** (**A**) Schematic of sweeping RMF as an actuation scheme for isotropic targets. Plane of rotation undergoes one revolution during the experiment with sweeping RMF opposed to constant RMF where the plane of rotation is fixed during the actuation period. (**B**) Computationally resolved time evolution of NP concentration in collagen modelled as a porous material. Sweeping RMF slightly outperforms constant RMF, and presence of fluid flow inside the chambers has subtle effect on increase of the transport.

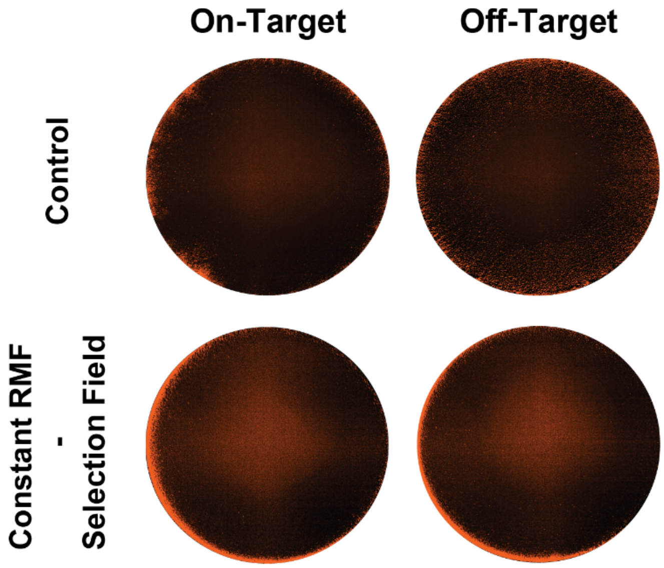


**Fig. S4: Images of change in NP distribution.** The differences in fluorescent intensity between the initial time point and the end point in collagen areas indicate a higher increase under the constant RMF without a selection field (bottom row) in both target and off-target wells, compared to the control (top row)

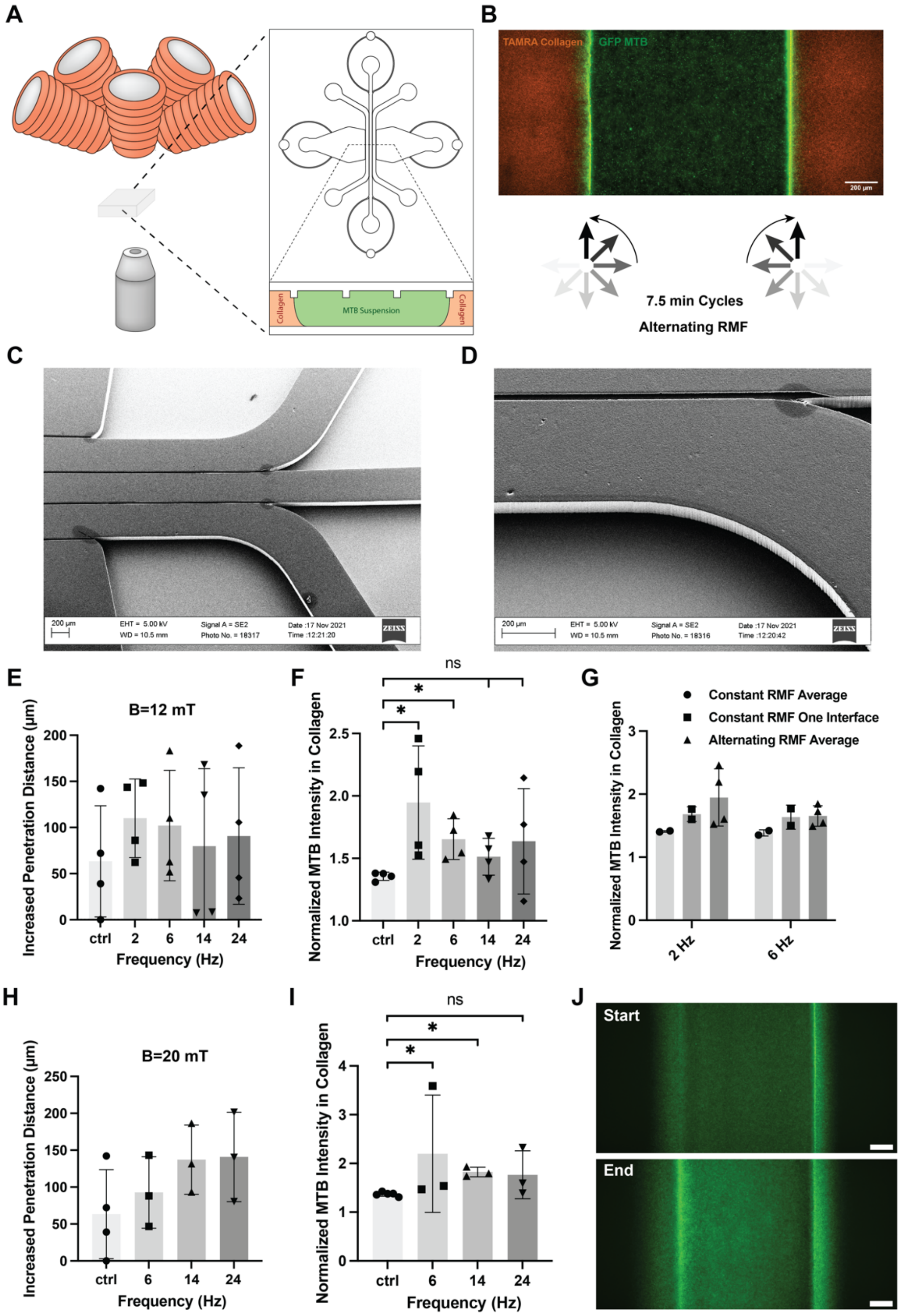


**Fig. S5: In vitro test platform to study influence of RMF driving condition on MTB penetration into tissue mimicking matrices.** (**A**) Schematic of the contact line pinning microfluidic devices exposed to magnetic actuation. (**B**) Compartmentalization of the bacteria and collagen gel through microfabricated contact lines. Suspension of GFP-expressing bacteria (green) is introduced between two chambers filled with TAMRA labelled collagen (red). Alternating RMF was used for balanced penetration of the bacteria at both interfaces. (**C**,**D**) SEM micrographs of the microfabricated ridge in a ring shape acting as the contact

line. (**E**) Increased penetration distance over the course of the experiments (30 min) under RMF at 12 mT with different frequencies. (**F**) Integrated MTB signal in collagen at the end of the magnetic actuation sequence at 12 mT, normalized by the initial timepoint (means ± SD; *$p$<0.05, **$p$<0.01, ***$p$<0.001, ****$p$<0.0001, Mann Whitney test, two-tailed). (**G**) Comparison of MTB penetration under different actuation schemes. Targeted single interface at constant RMF exhibits similar values to the average of both interfaces under the alternating RMF. (**H**) Increased penetration distance over the course of the experiments (30 min) under RMF at 12 mT with different frequencies. (**I**) MTB signal in collagen at the end of the magnetic actuation at 12 mT normalized by the initial time point (means ± SD; *$p$<0.05, **$p$<0.01, ***$p$<0.001, ****$p$<0.0001, Mann Whitney test, two-tailed). (**J**) Representative images of the bacteria before and after the magnetic actuation (scale bars: 200 μm). Source data are provided as a Source Data file. Graphical elements (electromagnet) have been adapted from Schuerle *et al.,Sci. Adv.* 2019; 5:eaav480. Reprinted with permission from AAAS.

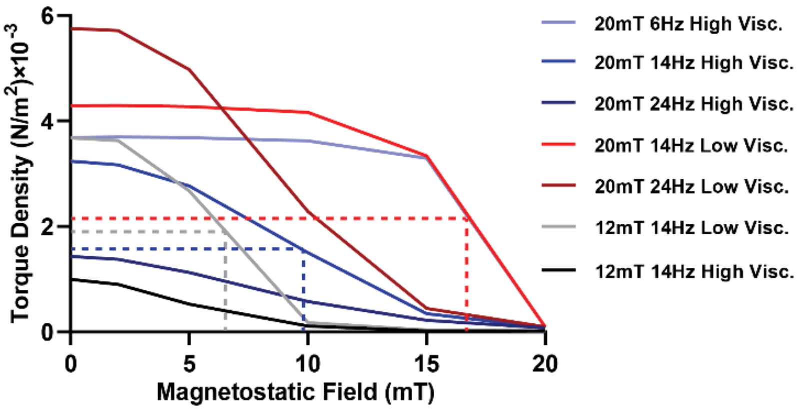


**Fig. S6: Simulated average torque densities in bacterial suspensions under various RMF parameters in low and high viscosity mediums.** Increasing the field magnitude in the presence of higher resistance compensates for the loss in torque density under weaker fields and increases the required magnetostatic field to suppress off-target transport.

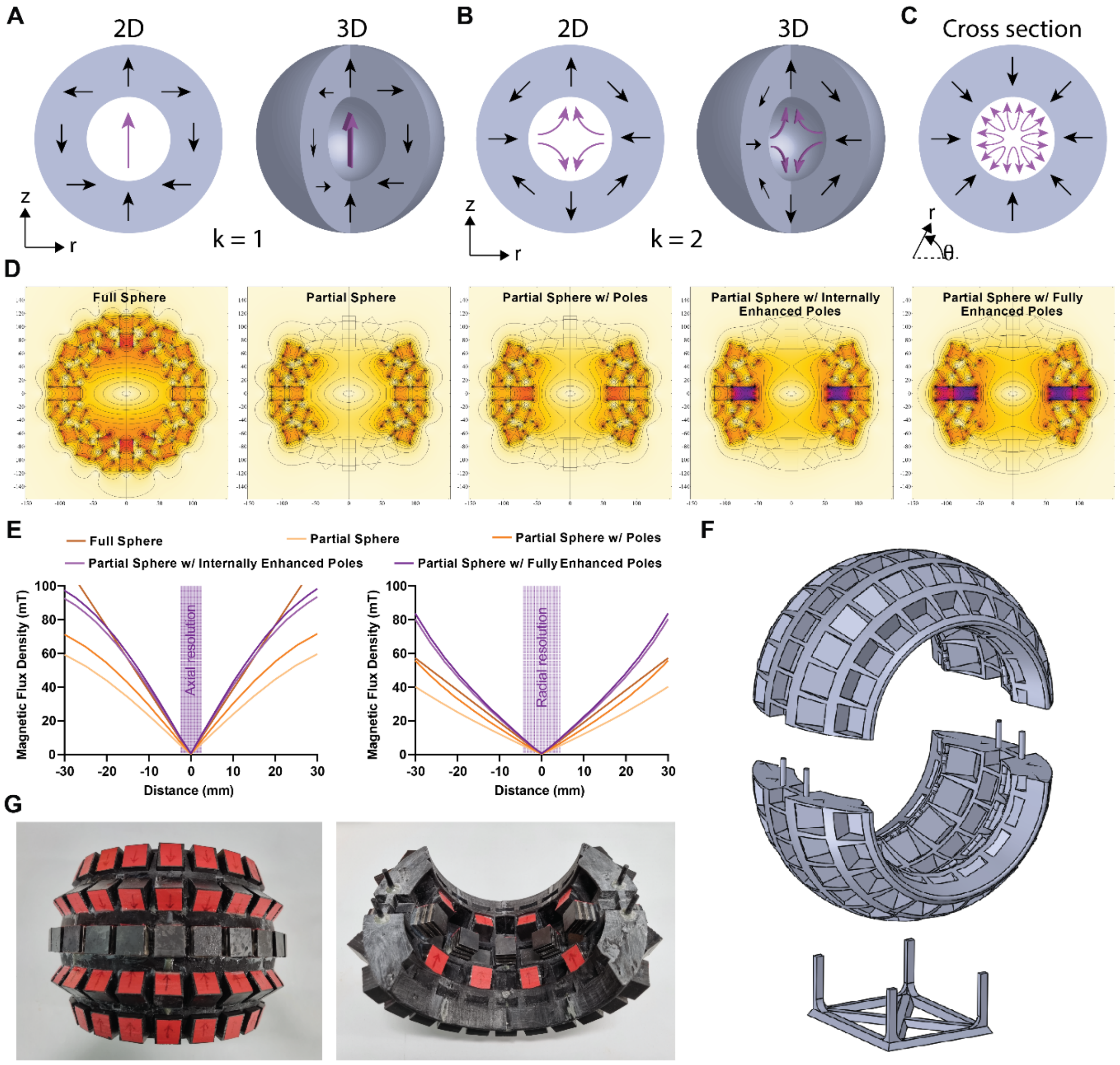


**Fig. S7: Description and simulation of magic sphere array to generate the desired magnetostatic selection field.** (**A**) Magic sphere representing the transformation of Halbach cylinder $k$=1 into 3D for uniform magnetic fields. (**B**) 3D transformation of the Halbach cylinder with $k$=2 to create a zero point in 3D space. (**C**) Cross section of the sphere resulting from revolution of the $k$=2 array. (**D**) Computational modeling of the magnetic field generated by the $k$=2 magic sphere formed by ferrite block magnets. Geometric constraints from the DC coil lead to a partial sphere with somewhat reduced resolution. Adding additional magnets to the middle row and supplementing them with stronger NdFeB magnets restores the lost confinement of the zero point. (**E**) Simulated magnetic field profiles in axial and radial directions. Higher resolution in the axial direction arises from deviation of the array in cross sectional plane from ideal $k$=2 arrangement. Profiles confirm the desired 1 cm resolution was attained by creating enhanced poles in the middle row of the sphere. (**F**) Designed geometry for the skeleton of the modified magic sphere. Indentations on both inner and outer surfaces are incorporated as slots for stacks of block magnets. (**G**) Fabricated modified magic sphere with mounted magnets. Inner and outer layers of magnets are assembled in way that ensures 2-fold symmetry of the zero point, assuming both halves are brought together fully. In practice, the gradient was somewhat stronger in the y direction than the x direction due to a gap that remained after clamping both halves together.

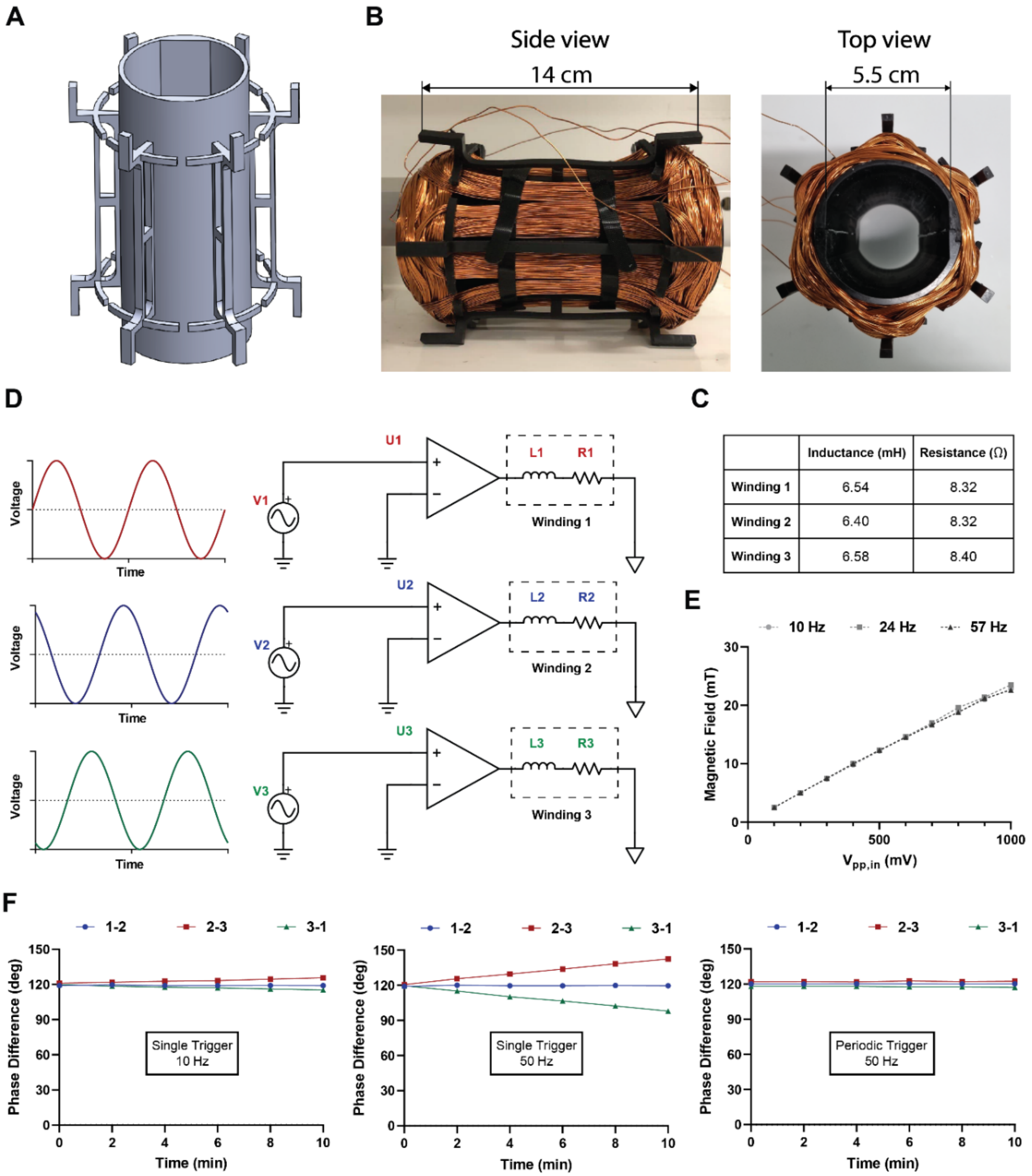


| | Inductance (mH) | Resistance (Ω) |
|---|---|---|
| Winding 1 | 6.54 | 8.32 |
| Winding 2 | 6.40 | 8.32 |
| Winding 3 | 6.58 | 8.40 |

**Fig. S8**: **Supplementary description of the three-phase coil design for generating full-body RMF for mice.** (**A**) CAD geometry of the stator designed for the three-phase winding. Dedicated slots to each winding ensure compact structure of the AC coil. (**B**) Wound AC coil around the 3D printed stator. The central portion of the coil exhibits highest compactness providing space for the DC coil. (**C**) Electrical properties of individual windings forming the AC coil. Symmetric windings yield less than 3% variability in electrical properties. (**D**) Electric circuit of the RMF generating component. Phase-shifted input signals from Digilent devices are amplified and sent to the windings of the coil. (**E**) Magnitude of RMF generated by the AC coil as a function of peak-to-peak input voltage. Amplification of the signal from the waveform generators is consistent in the desired range of frequencies and fulfills the 20 mT requirement for the magnitude. (**F**)

Cross-triggering strategy for synchronizing input from two waveform generators. When two waveforms are generated with a certain phase lag from two different devices, minute deviations in each cycle propagate over time and disrupt the phase different particular at higher frequencies. Periodic triggering resets the phase lags leading to consistent generation of uniform RMF.

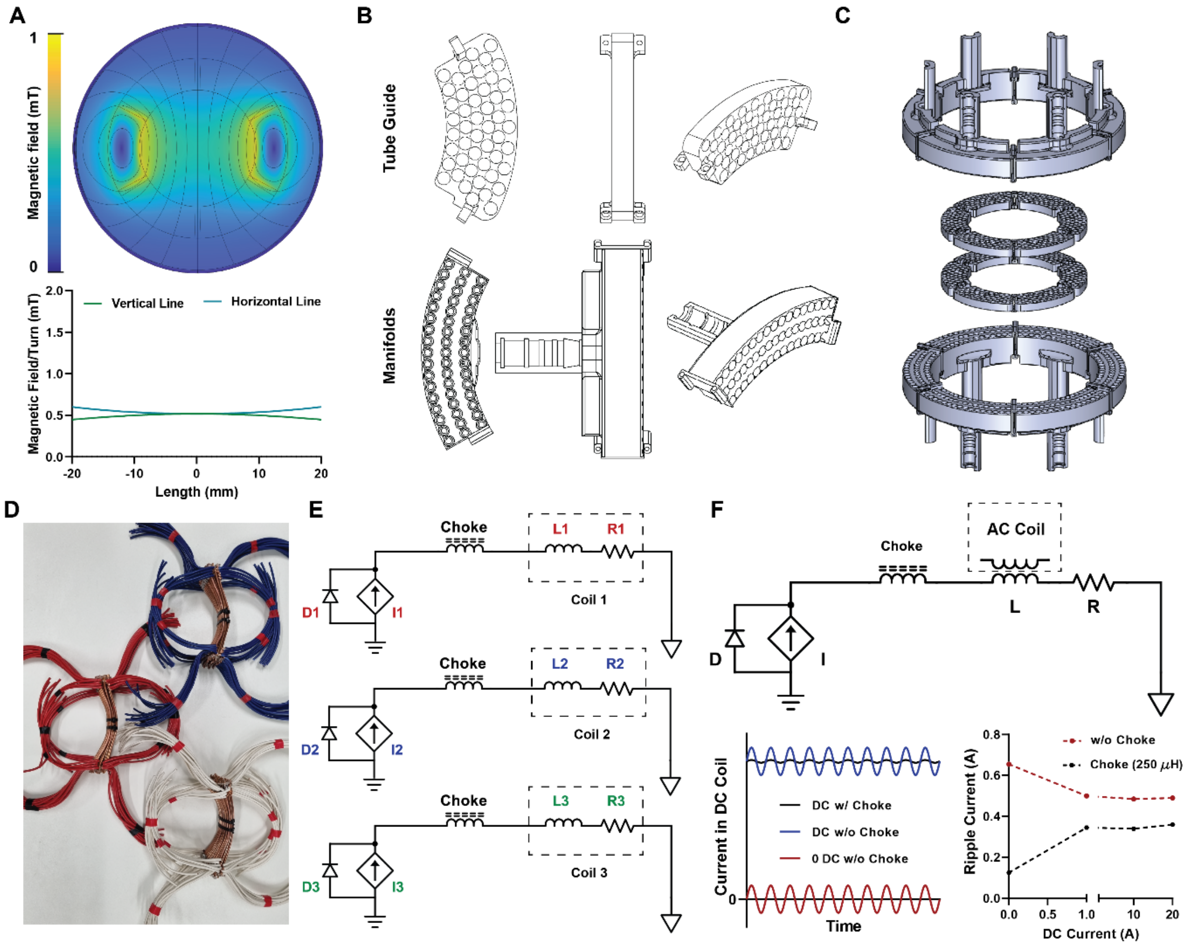


**Fig. S9**: **Design and fabrication of the DC offset coils.** (**A**) Idealized 2D simulation of the static field generated per each turn of one coil pair. 0.5 mT per ampere-turn provides an upper bound for the attainable field from each phase. (**B**) Tube guide and manifold geometries. Tube guides serve to hold the copper tubes straight and in close contact with the AC coil. Note that an insulating laquer is applied to the copper to minimize the risk of short circuits. Manifolds are fabricated to collect the circulating water in the hydraulic circuit enabling efficient heat dissipation in the system. (**C**) CAD assembly of the tube guides and manifolds. Distances are set according to the geometrical constraints from the AC coil and the array of magnets. (**D**) Single coils composed of copper tubes and connecting wires, before their full assembly. The original DC offset coil was planned to contain 3 pairs of parallel tube asseblies, but space contraints later caused this to be revised to 2 pairs. (**E**) The originally planned electrical circuit of the system to power the DC offset coils. Chokes are included to suppress the ripple current due to inductive coupling with the RMF coils and diodes parallel to the power supplies protect them from reverse current caused by inductive reactance in the circuit. The diodes and chokes were omitted from the design actually used for the mouse-scale setup because they were found to be unnecessary. (**F**) Effect of the choke in circuits with two smaller inductively coupled test coils. Reduced suppression of ripple noise at higher currents implies that large iron powder cores with lower permeability and relatively modest number of turns are required to avoid saturating the choke. These considerations would become more important if the operating frequency of the setup were raised substantially.

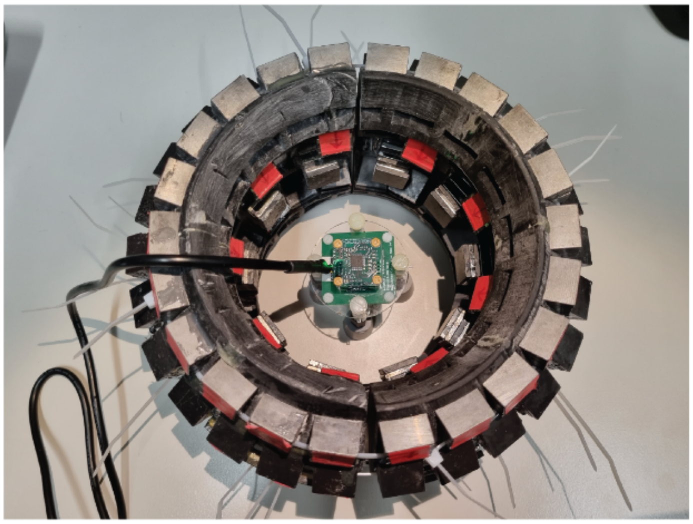

**Fig. S10**: **Characterization of the magnetostatic field generated by the array of permanent magnets**. Assembled magic sphere for magnetic characterization is shown. A cube of Hall sensors (Magnebotix prototype) located at the center of the sphere measures the selection field from the magnets in a 3D volume.

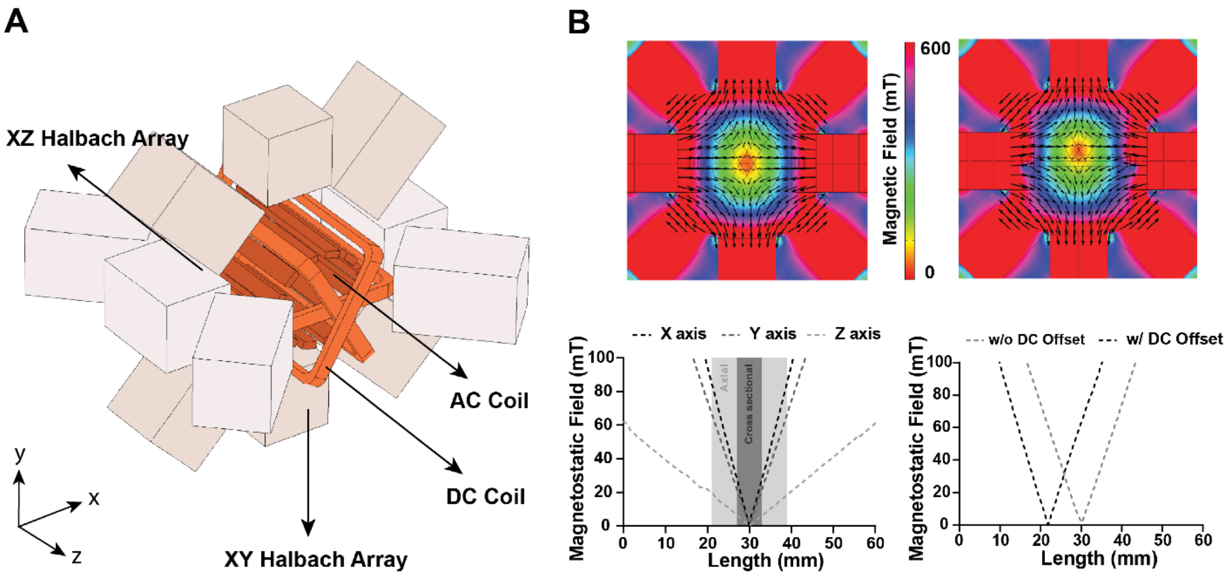


**Fig. S11: Preliminary computational study of displacement of the field-free point.** (**A**) Simplified multilayer configuration envisioned for the selection field setup. Note that the configuration of permanent magnets reflects an earlier approximation of the array that was ultimately used, but the concept remains the same. The DC coil positioned between the AC coil and array of magnets is responsible for adding offset to the magnetostatic field from the permanent magnets and moving the zero point. (**B**) Computational modeling of the static field from the Halbach arrays and the DC coil. Running current through the DC coils leads to shift in the zero-point location. Source data are provided as a Source Data file.

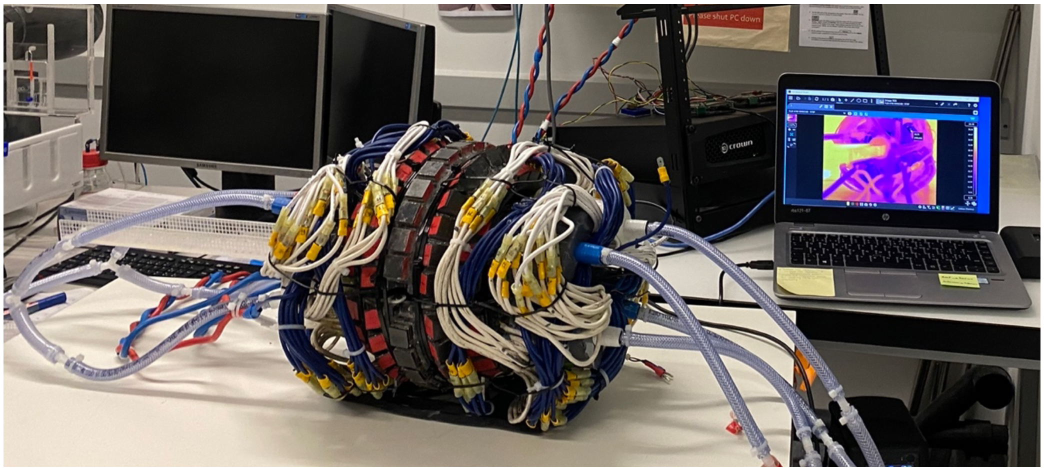

**Fig. S12: Photograph of the assembled prototype.** An IR thermography readout monitoring the setup, and the class D audio amplifier powering the RMF coils are visible in the background. The Digilent devices that served as function generators are controlled with the computer and are sitting atop the audio amplifier.

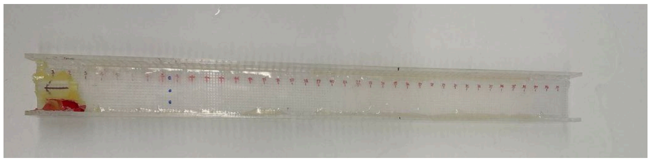

**Fig S13: Finely gridded sliding track for axial positioning of tumors**. A gridded track made from laser cut acrylic sheets was used to position mice with tumors within the setup in the in vivo study.

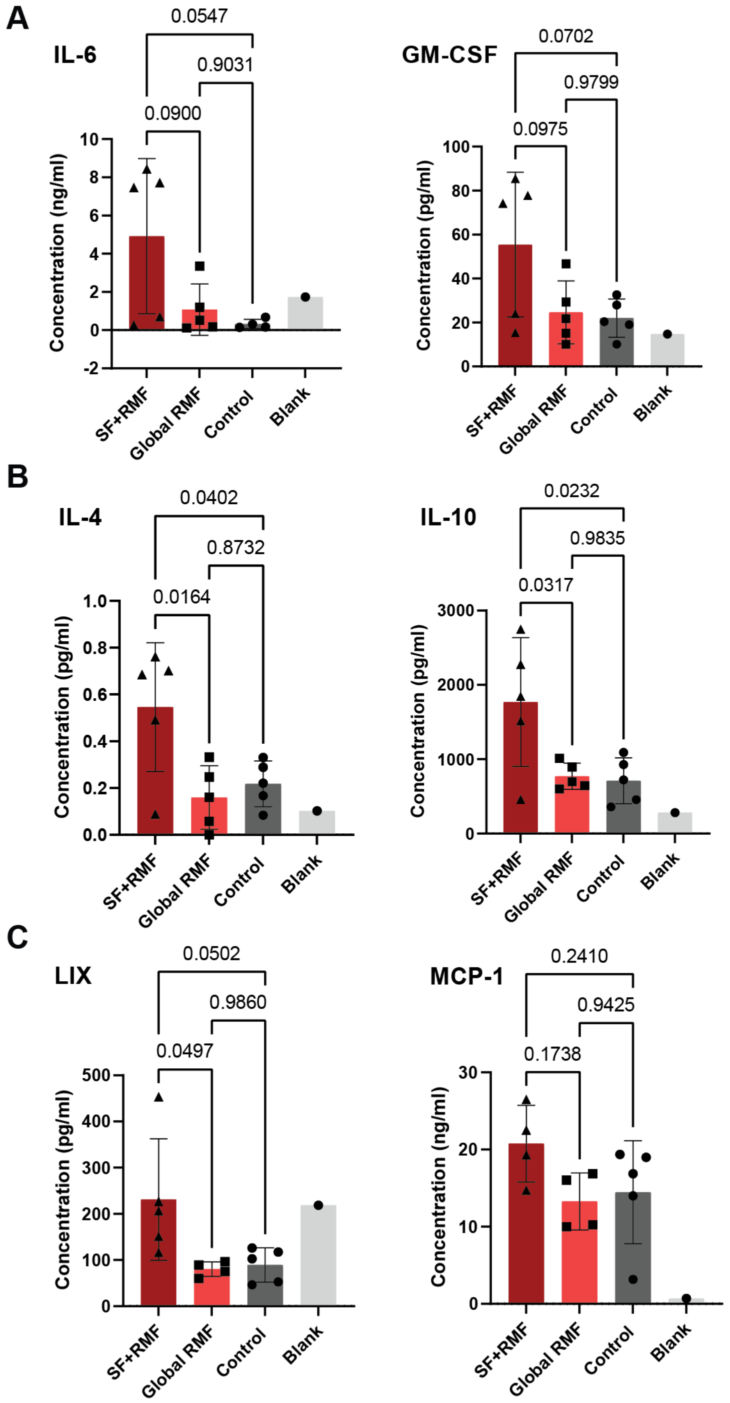


**Fig. S14: Serum cytokine levels.** Cytokines in blood samples collected at 24 hr were measured using Limunex multiplex assay. Elevated levels in the SF+RMF group were observed for several (**A**) pro-inflammatory cytokines, (**B**) anti-inflammatory cytokines, and (**C**) chemokines. Source data are provided as a Source Data file.
