# Supplementary material for: Spatially selective delivery of living magnetic microrobots through torque-focusing
Source: Nat Commun. 2024 Mar 9;15:2160. doi: 10.1038/s41467-024-46407-4 (PMC10924878; doi:10.1038/s41467-024-46407-4)
Supplement: Supplementary file 3 — Reporting Summary [file 41467_2024_46407_MOESM3_ESM.pdf]

nature portfolio

Corresponding author(s): Simone Schuerle

Last updated by author(s): Dec 15, 2023

Reporting Summary

Nature Portfolio wishes to improve the reproducibility of the work that we publish. This form provides structure for consistency and transparency in reporting. For further information on Nature Portfolio policies, see our Editorial Policies and the Editorial Policy Checklist.

Statistics

For all statistical analyses, confirm that the following items are present in the figure legend, table legend, main text, or Methods section.

n/a	Confirmed	
☐	☒	The exact sample size (*n*) for each experimental group/condition, given as a discrete number and unit of measurement
☐	☒	A statement on whether measurements were taken from distinct samples or whether the same sample was measured repeatedly
☐	☒	The statistical test(s) used AND whether they are one- or two-sided<br>*Only common tests should be described solely by name; describe more complex techniques in the Methods section.*
☒	☐	A description of all covariates tested
☐	☒	A description of any assumptions or corrections, such as tests of normality and adjustment for multiple comparisons
☐	☒	A full description of the statistical parameters including central tendency (e.g. means) or other basic estimates (e.g. regression coefficient) AND variation (e.g. standard deviation) or associated estimates of uncertainty (e.g. confidence intervals)
☐	☒	For null hypothesis testing, the test statistic (e.g. *F*, *t*, *r*) with confidence intervals, effect sizes, degrees of freedom and *P* value noted<br>*Give P values as exact values whenever suitable.*
☒	☐	For Bayesian analysis, information on the choice of priors and Markov chain Monte Carlo settings
☒	☐	For hierarchical and complex designs, identification of the appropriate level for tests and full reporting of outcomes
☒	☐	Estimates of effect sizes (e.g. Cohen's *d*, Pearson's *r*), indicating how they were calculated

Our web collection on statistics for biologists contains articles on many of the points above.

Software and code

Policy information about availability of computer code

Data collection	Digilent WaveForms, Metrolab THM1176, NIS-Elements AR 5.02.03, SparkControl MethodEditor (Tecan)<br>AzureSpot (Azure Biosystems), Hallprobe array (Magnebotix prototype)
Data analysis	MATLAB 2022b, GraphPad Prism 8.0, COMSOL Multiphysics 6, Fiji ImageJ, Excel

For manuscripts utilizing custom algorithms or software that are central to the research but not yet described in published literature, software must be made available to editors and reviewers. We strongly encourage code deposition in a community repository (e.g. GitHub). See the Nature Portfolio guidelines for submitting code & software for further information.

Data

Policy information about availability of data

All manuscripts must include a data availability statement. This statement should provide the following information, where applicable:

- Accession codes, unique identifiers, or web links for publicly available datasets
- A description of any restrictions on data availability
- For clinical datasets or third party data, please ensure that the statement adheres to our policy

The authors confirm that the data supporting the findings of this study are available within the article and its supplementary materials. Source data are provided with this paper in a separate source data file. Any additional data or designs (e.g. CAD) can be made available upon request to the corresponding author.

Research involving human participants, their data, or biological material

Policy information about studies with human participants or human data. See also policy information about sex, gender (identity/presentation), and sexual orientation and race, ethnicity and racism.

Reporting on sex and gender	N/A
Reporting on race, ethnicity, or other socially relevant groupings	N/A
Population characteristics	N/A
Recruitment	N/A
Ethics oversight	N/A

Note that full information on the approval of the study protocol must also be provided in the manuscript.

Field-specific reporting

Please select the one below that is the best fit for your research. If you are not sure, read the appropriate sections before making your selection.

☒ Life sciences ☐ Behavioural & social sciences ☐ Ecological, evolutionary & environmental sciences

For a reference copy of the document with all sections, see nature.com/documents/nr-reporting-summary-flat.pdf

Life sciences study design

All studies must disclose on these points even when the disclosure is negative.

Sample size	18 female BALB/c nude mice. Sample size was estimated based on effect sizes reported in other studies on magnetically accumulated bacteria. Sample sizes had to be kept as low as possible to ensure approval of the protocol.
Data exclusions	In the serum cytokine analysis, outliers were removed using the ROUT method with (Q=5%).
Replication	The experimental in vivo procedures described were conducted only once. It was not possible to repeat the experiment due to license restrictions on the allowable number of mice. All in vitro experiments were repeated several times in the course of the development of the methods.
Randomization	Mice were initially ranked according to their tumor size. Experiments were performed over the course of three days starting with the mice having larger tumors. A random generator was used each day to equally distribute subjects among the intervention groups.<br>Spheroids for in vitro experiments were randomly assigned. Random assignment was not relevant in other experiments, especially those focused on characterization.
Blinding	Since quantification methods used in this study such as colony counting are less prone to any subjective variation, no blinding was applied.

Reporting for specific materials, systems and methods

We require information from authors about some types of materials, experimental systems and methods used in many studies. Here, indicate whether each material, system or method listed is relevant to your study. If you are not sure if a list item applies to your research, read the appropriate section before selecting a response.

Materials & experimental systems

n/a	Involved in the study	
☒	☐	Antibodies
☐	☒	Eukaryotic cell lines
☒	☐	Palaeontology and archaeology
☐	☒	Animals and other organisms
☒	☐	Clinical data
☒	☐	Dual use research of concern
☒	☐	Plants

Methods

n/a	Involved in the study	
☒	☐	ChIP-seq
☒	☐	Flow cytometry
☒	☐	MRI-based neuroimaging

Eukaryotic cell lines

Policy information about cell lines and Sex and Gender in Research

Cell line source(s)	MCF-7 (purchased from ATCC, catalog number HTB-22) human adenocarcinoma cells. This established cell line is derived from a 69-year-old female suffering from a metastatic breast cancer.
Authentication	The MCF7 was verified by the supplier, ATCC. According to their website, they typically use STR profiling for human cell types.
Mycoplasma contamination	We confirm that the stock cell lines was tested negative for mycoplasma contamination.
Commonly misidentified lines (See ICLAC register)	No commonly misidentified cell lines were used in the study.

Animals and other research organisms

Policy information about studies involving animals; ARRIVE guidelines recommended for reporting animal research, and Sex and Gender in Research

Laboratory animals	18 female BALB/c nude mice. They arrived in the animal facility at 8 weeks old and went through a acclimatization period for 6 weeks. Animals were housed in cages with a HEPA-filtered air supply, with the air in the room also separately HEPA-filtered and maintained at an ambient temperature of 22±2 °C and a relative humidity of 55±10 %. The specific temperature and relative humidity experienced in the cages depends on the number of animals (always ≥2 and ≤5), but does not deviate dramatically from these values. The animals were maintained on a 12 hour light/dark cycle with 10 minute transitional fades. The experiments were performed during regular working hours, which coincided with the light part of the cycle.
Wild animals	The study did not involve wild animals.
Reporting on sex	Sex was not considered in this study.
Field-collected samples	The study did not involve samples collected from the field.
Ethics oversight	Animal experiments were carried out under license number ZH151/2020 and in compliance with the regulations from Veterinäramt Kanton Zürich, Switzerland.

Note that full information on the approval of the study protocol must also be provided in the manuscript.

Plants

Seed stocks	N/A
Novel plant genotypes	N/A
Authentication	N/A
