# Supplementary material for: Spatially selective delivery of living magnetic microrobots through torque-focusing
Source: Nat Commun. 2024 Mar 9;15:2160. doi: 10.1038/s41467-024-46407-4 (PMC10924878; doi:10.1038/s41467-024-46407-4)
Supplement: Supplementary file 4 — Source Data [file 41467_2024_46407_MOESM4_ESM.zip › Data_Sorted_by_Figures/Fig_4/4H/Field Characterization/x axis measurements/ReadingHallProbeData.pdf]

```
NotebookDirectory[]
```
Notebook–Verzeichnis

```
P:\Michael\MetroLabHallProbe\230224\x0_y-5_z0\
```

(-5,0,0) Blue offset 0A White Offset 0A RMF off

```
Data = Import[NotebookDirectory[] <> "x-5_y0_z0_no_offset_RMFoff.txt", "Table"];
```
importi··· Notebook–Verzeichnis Tabelle

```
Bvst = Table[{AbsoluteTime[Data[[i]][[9]]] - AbsoluteTime[Data[[2]][[9]]],
    AbsoluteTime[Data[[i]][[2]]]}, {i, 2, Length[Data]}];
```
Tabelle absolute Zeit seit 1900 absolute Zeit seit 1900
absolute Zeit seit 1900 Länge

```
Bxvst = Table[{AbsoluteTime[Data[[i]][[9]]] - AbsoluteTime[Data[[2]][[9]]],
    AbsoluteTime[Data[[i]][[3]]]}, {i, 2, Length[Data]}];
```
Tabelle absolute Zeit seit 1900 absolute Zeit seit 1900
absolute Zeit seit 1900 Länge

```
Byvst = Table[{AbsoluteTime[Data[[i]][[9]]] - AbsoluteTime[Data[[2]][[9]]],
    AbsoluteTime[Data[[i]][[4]]]}, {i, 2, Length[Data]}];
```
Tabelle absolute Zeit seit 1900 absolute Zeit seit 1900
absolute Zeit seit 1900 Länge

```
Bzvst = Table[{AbsoluteTime[Data[[i]][[9]]] - AbsoluteTime[Data[[2]][[9]]],
    AbsoluteTime[Data[[i]][[5]]]}, {i, 2, Length[Data]}];
```
Tabelle absolute Zeit seit 1900 absolute Zeit seit 1900
absolute Zeit seit 1900 Länge

```
ListPlot[{Bvst, Bxvst, Byvst, Bzvst}, Joined → True]
```
listenbezogene Graphik verknüpft? wahr

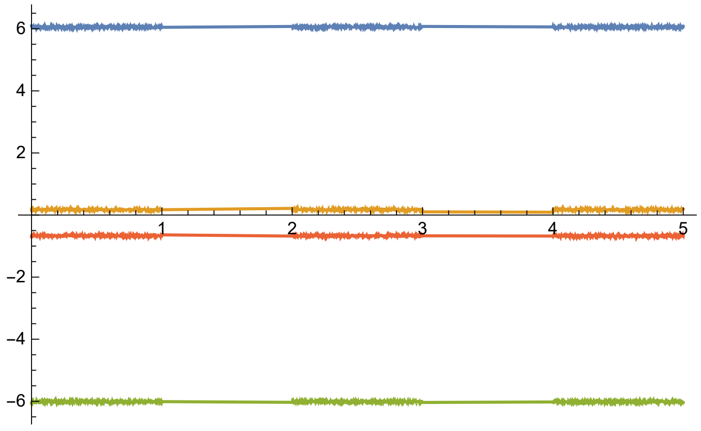


```
Start = 100;
Stop = 1000;
```

```
ListPlot[{Table[{Bvst[[i]][[1]], Bvst[[i]][[2]]}, {i, Start, Stop}],
  Table[{Bxvst[[i]][[1]], Bxvst[[i]][[2]]}, {i, Start, Stop}],
  Table[{Byvst[[i]][[1]], Byvst[[i]][[2]]}, {i, Start, Stop}],
  Table[{Bzvst[[i]][[1]], Bzvst[[i]][[2]]}, {i, Start, Stop}]}, Joined → True]
```

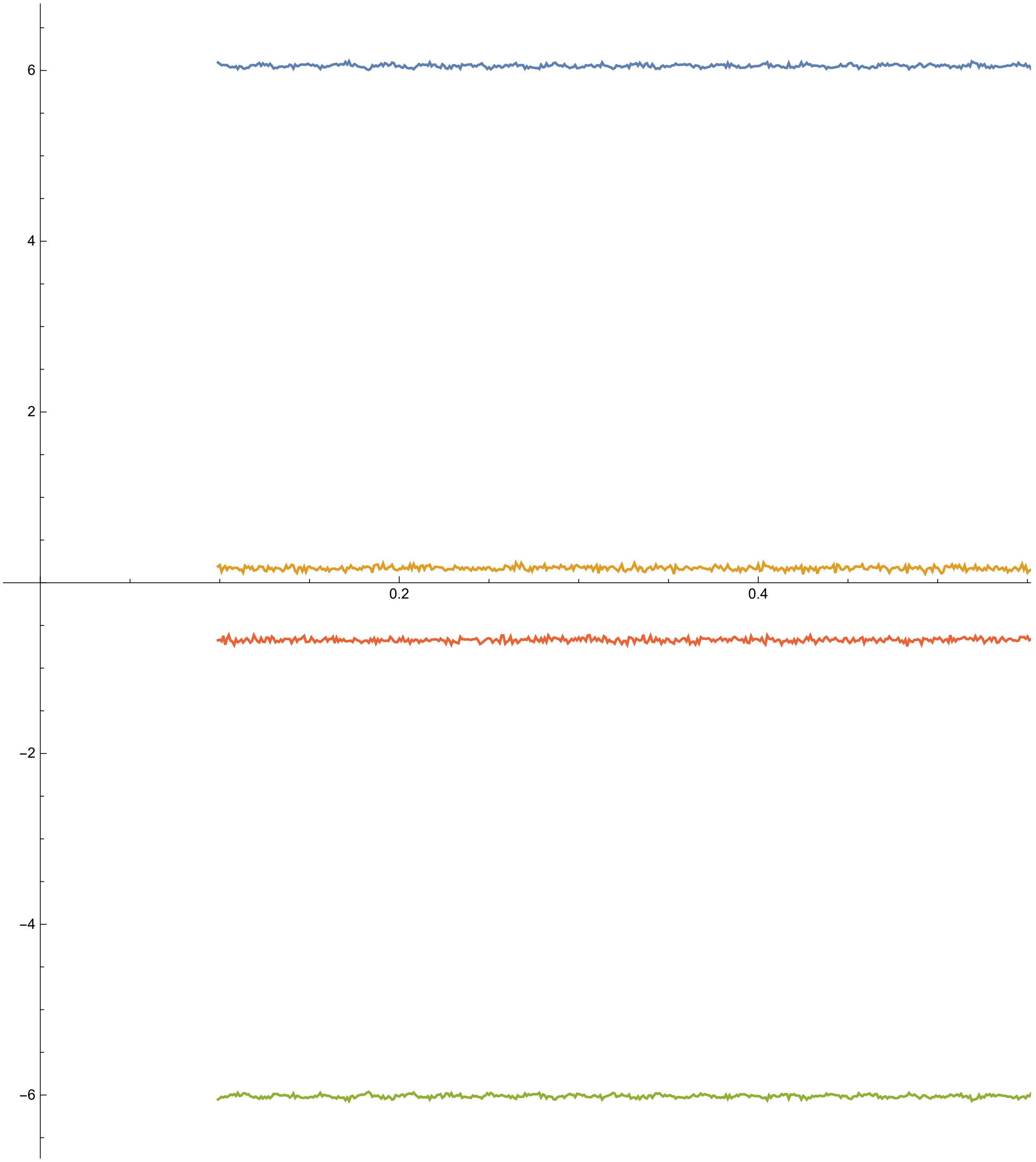

```
ListPlot[{Table[{Bvst[[i]][[1]], Bvst[[i]][[2]]}, {i, Start, Start + 50}],
  Table[{Bxvst[[i]][[1]], Bxvst[[i]][[2]]}, {i, Start, Start + 50}],
  Table[{Byvst[[i]][[1]], Byvst[[i]][[2]]}, {i, Start, Start + 50}],
  Table[{Bzvst[[i]][[1]], Bzvst[[i]][[2]]}, {i, Start, Start + 50}]}, Joined → True]
```

listenbezo··· Tabelle Tabelle Tabelle Tabelle verknüpft? wahr

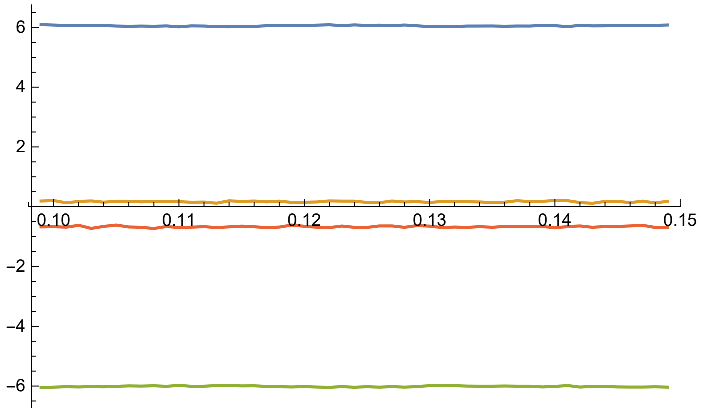


```
TableForm[{Mean[Table[Bvst[[i]][[2]], {i, Start, Stop}]],
  Mean[Table[Bxvst[[i]][[2]], {i, Start, Stop}]],
  Mean[Table[Byvst[[i]][[2]], {i, Start, Stop}]],
  Mean[Table[Bzvst[[i]][[2]], {i, Start, Stop}]]}, TableDirections → Row]
```

Tabellendars··· arit··· Tabelle arit··· Tabelle arit··· Tabelle arit··· Tabelle Richtung der Tabellen··· Zeile

6.05444 0.169789 -6.01487 -0.669121

```
TableForm[Table[{(Bvst[[i]][[1]] - Bvst[[Start]][[1]]) * 1000, Bvst[[i]][[2]],
  Bxvst[[i]][[2]], Byvst[[i]][[2]], Bzvst[[i]][[2]]}, {i, Start, Start + 50}]]
```

Tabellendar·· Tabelle

0.	6.09364	0.190249	-6.05321	-0.674489
1.0004	6.07749	0.206314	-6.03731	-0.666411
2.00033	6.06138	0.130096	-6.0211	-0.685381
3.00026	6.06456	0.175761	-6.03002	-0.621977
4.00019	6.06315	0.193676	-6.01642	-0.725972
5.00011	6.06327	0.151377	-6.02549	-0.658663
6.00004	6.04516	0.182824	-6.01114	-0.61376
7.00045	6.03508	0.182148	-5.99416	-0.677505
8.00037	6.04367	0.163033	-6.002	-0.68943
9.0003	6.03615	0.174608	-5.98938	-0.729363
10.0002	6.04927	0.175329	-6.01042	-0.66168
11.0002	6.01783	0.170922	-5.97487	-0.697098
12.0001	6.05082	0.147083	-6.01008	-0.685388
13.	6.04512	0.155285	-6.00638	-0.665412
14.0004	6.02392	0.117891	-5.98188	-0.700659
15.0003	6.02028	0.198172	-5.97921	-0.673441
16.0003	6.03232	0.175426	-5.99459	-0.650438
17.0002	6.03058	0.190225	-5.99028	-0.669528
18.0001	6.0568	0.162922	-6.01382	-0.701611
19.0001	6.06245	0.186134	-6.0211	-0.681958
20.0005	6.06303	0.143845	-6.03018	-0.613649
21.0004	6.05484	0.14741	-6.01755	-0.6545
22.0003	6.07401	0.159106	-6.03307	-0.685863
23.0002	6.08814	0.193993	-6.04484	-0.698404
24.0002	6.05517	0.186491	-6.01763	-0.646903
25.0001	6.08313	0.186072	-6.04097	-0.690262
26.	6.06246	0.143012	-6.02096	-0.693513
27.0004	6.07335	0.134555	-6.03775	-0.642674
28.0004	6.05493	0.190532	-6.01769	-0.642945
29.0003	6.07838	0.159069	-6.037	-0.689923
30.0002	6.05329	0.171656	-6.01789	-0.630751
31.0001	6.0234	0.13842	-5.98663	-0.649934
32.0001	6.03295	0.177937	-5.98986	-0.697399
33.0005	6.02719	0.171099	-5.98612	-0.681269
34.0004	6.04417	0.166992	-6.00194	-0.69347
35.0003	6.04522	0.159285	-6.00637	-0.665454
36.0003	6.04694	0.134039	-6.00604	-0.689192
37.0002	6.0364	0.151349	-5.99849	-0.65825
38.0001	6.04585	0.202355	-6.00643	-0.658897
39.	6.04478	0.167357	-6.00647	-0.658537
40.0004	6.06798	0.17838	-6.02946	-0.659002
41.0004	6.05865	0.209878	-6.0137	-0.706094
42.0003	6.02263	0.202258	-5.98233	-0.66553
43.0002	6.06946	0.138551	-6.03375	-0.642654
44.0001	6.05051	0.114044	-6.01006	-0.689047
45.0001	6.05334	0.178333	-6.01441	-0.661772
46.	6.06838	0.182348	-6.0294	-0.662042
47.0004	6.06937	0.134551	-6.03375	-0.642613
48.0003	6.06887	0.186765	-6.03401	-0.622152
49.0003	6.06606	0.126017	-6.02498	-0.6934
50.0002	6.07942	0.182027	-6.03692	-0.694159

(5,0,0) Blue offset -16A White Offset -49A RMF on

```
Data = Import[NotebookDirectory[] <> "x5_y0_z0_B-16_W-49_RMFon.txt", "Table"];
       importi··· Notebook–Verzeichnis                                     Tabelle

Bvst = Table[{AbsoluteTime[Data[[i]][[9]]] - AbsoluteTime[Data[[2]][[9]]],
       Tabelle  absolute Zeit seit 1900            absolute Zeit seit 1900
    AbsoluteTime[Data[[i]][[2]]]}, {i, 2, Length[Data]}];
    absolute Zeit seit 1900                   Länge
Bxvst = Table[{AbsoluteTime[Data[[i]][[9]]] - AbsoluteTime[Data[[2]][[9]]],
        Tabelle  absolute Zeit seit 1900            absolute Zeit seit 1900
    AbsoluteTime[Data[[i]][[3]]]}, {i, 2, Length[Data]}];
    absolute Zeit seit 1900                   Länge
Byvst = Table[{AbsoluteTime[Data[[i]][[9]]] - AbsoluteTime[Data[[2]][[9]]],
        Tabelle  absolute Zeit seit 1900            absolute Zeit seit 1900
    AbsoluteTime[Data[[i]][[4]]]}, {i, 2, Length[Data]}];
    absolute Zeit seit 1900                   Länge
Bzvst = Table[{AbsoluteTime[Data[[i]][[9]]] - AbsoluteTime[Data[[2]][[9]]],
        Tabelle  absolute Zeit seit 1900            absolute Zeit seit 1900
    AbsoluteTime[Data[[i]][[5]]]}, {i, 2, Length[Data]}];
    absolute Zeit seit 1900                   Länge
```

```
ListPlot[{Bvst, Bxvst, Byvst, Bzvst}, Joined → True]
listenbezogene Graphik                   verknüpft? wahr
```

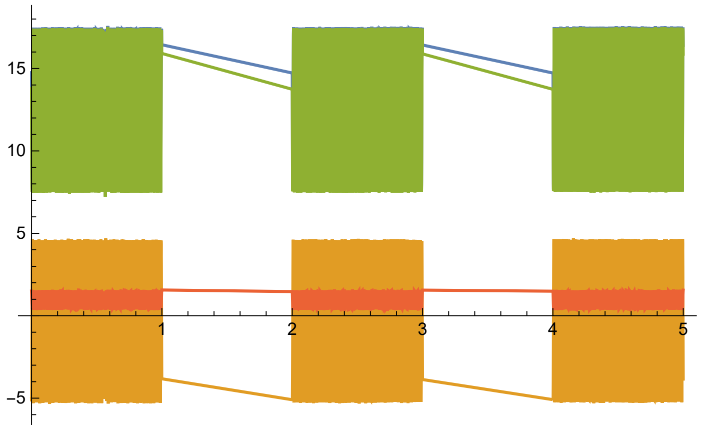


```
Start = 100;
Stop = 1000;
```

```
ListPlot[{Table[{Bvst[[i]][[1]], Bvst[[i]][[2]]}, {i, Start, Stop}],
  Table[{Bxvst[[i]][[1]], Bxvst[[i]][[2]]}, {i, Start, Stop}],
  Table[{Byvst[[i]][[1]], Byvst[[i]][[2]]}, {i, Start, Stop}],
  Table[{Bzvst[[i]][[1]], Bzvst[[i]][[2]]}, {i, Start, Stop}]}, Joined → True]
```

listenbezo··· Tabelle Tabelle Tabelle Tabelle verknüpft? wahr

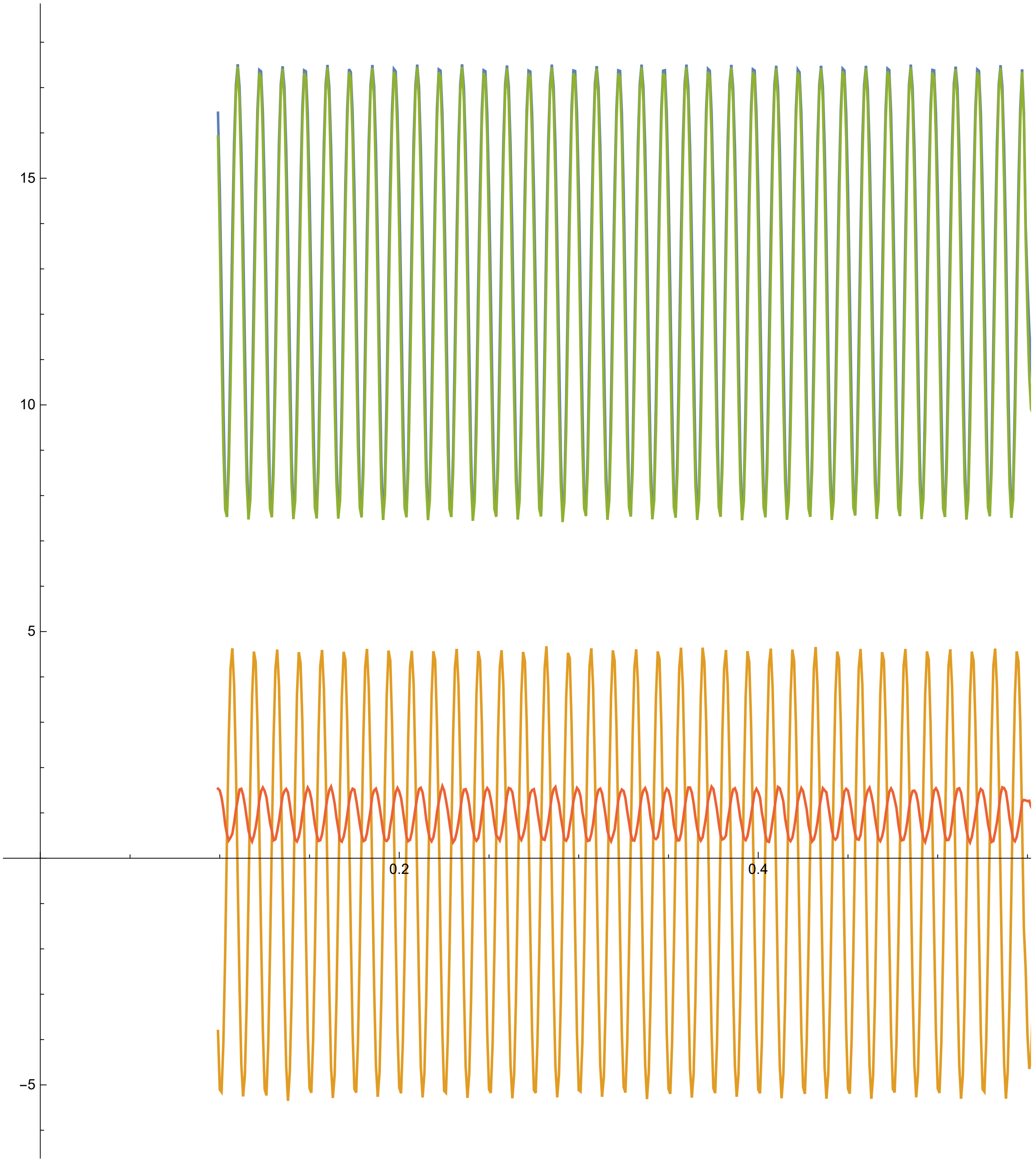

```
ListPlot[{Table[{Bvst[[i]][[1]], Bvst[[i]][[2]]}, {i, Start, Start + 50}],
  Table[{Bxvst[[i]][[1]], Bxvst[[i]][[2]]}, {i, Start, Start + 50}],
  Table[{Byvst[[i]][[1]], Byvst[[i]][[2]]}, {i, Start, Start + 50}],
  Table[{Bzvst[[i]][[1]], Bzvst[[i]][[2]]}, {i, Start, Start + 50}]}, Joined → True]
```

listenbezo··· Tabelle Tabelle Tabelle Tabelle verknüpft? wahr

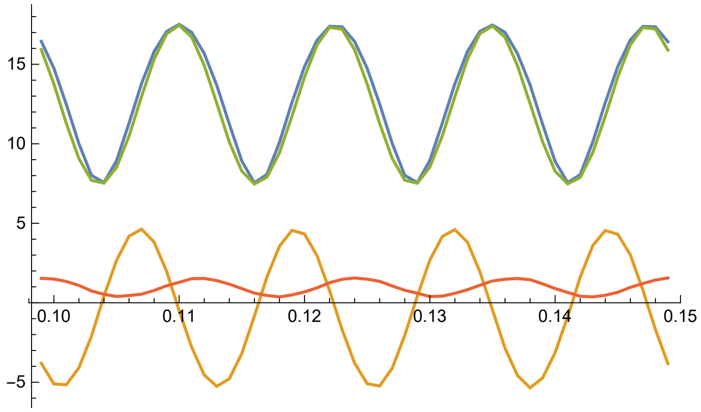


```
TableForm[{Mean[Table[Bvst[[i]][[2]], {i, Start, Stop}]],
  Mean[Table[Bxvst[[i]][[2]], {i, Start, Stop}]],
  Mean[Table[Byvst[[i]][[2]], {i, Start, Stop}]],
  Mean[Table[Bzvst[[i]][[2]], {i, Start, Stop}]]}, TableDirections → Row]
```

Tabellendars··· arit··· Tabelle arit··· Tabelle arit··· Tabelle arit··· Tabelle Richtung der Tabellen··· Zeile

12.993	−0.332161	12.4507	0.964828

```
TableForm[Table[{(Bvst[[i]][[1]] - Bvst[[Start]][[1]]) * 1000, Bvst[[i]][[2]],
Tabellendar·· Tabelle
   Bxvst[[i]][[2]], Byvst[[i]][[2]], Bzvst[[i]][[2]]}, {i, Start, Start + 50}]]
```

0.	16.445	-3.8113	15.9235	1.53466
0.999928	14.7569	-5.10322	13.7655	1.49502
1.99986	12.4623	-5.15787	11.2656	1.33839
2.99978	10.0023	-4.08175	9.0662	1.09068
3.99971	8.01568	-2.1056	7.69838	0.743364
4.99964	7.558	0.425575	7.52718	0.532619
6.00004	8.93295	2.66609	8.51673	0.393604
6.99997	11.2998	4.18947	10.4846	0.454952
7.9999	13.7949	4.62841	12.984	0.540599
8.99982	15.8085	3.81401	15.3219	0.775727
9.99975	17.0622	1.97604	16.9137	1.06903
10.9997	17.5131	-0.495514	17.4589	1.28487
11.9996	17.0072	-2.80336	16.7059	1.51621
13.	15.678	-4.54419	14.9267	1.53099
13.9999	13.6774	-5.25381	12.5508	1.39501
14.9999	11.2418	-4.78021	10.1074	1.16979
15.9998	8.91325	-3.18483	8.27709	0.890351
16.9997	7.54644	-0.840646	7.47471	0.608886
17.9996	8.07331	1.61143	7.89759	0.458036
18.9996	10.0898	3.55081	9.4371	0.37053
20.	12.6009	4.55741	11.7375	0.494293
20.9999	14.8544	4.32741	14.1937	0.681195
21.9998	16.5124	2.9606	16.2176	0.940228
22.9998	17.3904	0.766483	17.3275	1.26285
23.9997	17.3578	-1.68431	17.2127	1.4764
24.9996	16.4325	-3.79504	15.9122	1.55832
26.	14.7443	-5.09133	13.7577	1.48278
27.	12.4942	-5.23278	11.2654	1.34716
27.9999	10.0228	-4.11312	9.0787	1.05619
28.9998	8.02008	-2.05316	7.71277	0.787051
29.9997	7.55694	0.406271	7.52214	0.599753
30.9997	8.94919	2.68208	8.52875	0.393623
31.9996	11.3104	4.17305	10.5042	0.416413
33.	13.773	4.60096	12.9682	0.59265
33.9999	15.8025	3.81451	15.3132	0.822597
34.9998	17.0757	1.94136	16.9292	1.10163
35.9998	17.4704	-0.450561	17.4106	1.37168
36.9997	17.0103	-2.85573	16.7034	1.4807
37.9996	15.6679	-4.57621	14.9067	1.52702
39.	13.703	-5.35222	12.5308	1.45073
40.	11.2311	-4.72502	10.1192	1.1894
40.9999	8.88803	-3.13378	8.26909	0.893703
41.9998	7.56094	-0.87525	7.48207	0.648361
42.9997	8.05573	1.61096	7.88231	0.410799
43.9997	10.113	3.5978	9.44415	0.370153
44.9996	12.5857	4.54517	11.7269	0.470253
46.	14.8622	4.30718	14.209	0.661633
46.9999	16.5364	3.02088	16.2292	0.968789
47.9999	17.3808	0.794954	17.3204	1.21044
48.9998	17.3571	-1.6688	17.2175	1.4293
49.9997	16.4212	-3.83002	15.8921	1.55837

(-5,0,0) Blue offset -16A White Offset -49A RMF off

```
Data = Import[NotebookDirectory[] <> "x-5_y0_z0_B-16_W-49_RMFoff.txt", "Table"];
       importi··· Notebook-Verzeichnis                                   Tabelle

Bvst = Table[{AbsoluteTime[Data[[i]][[9]]] - AbsoluteTime[Data[[2]][[9]]],
       Tabelle absolute Zeit seit 1900           absolute Zeit seit 1900
    AbsoluteTime[Data[[i]][[2]]]}, {i, 2, Length[Data]}];
    absolute Zeit seit 1900                   Länge
Bxvst = Table[{AbsoluteTime[Data[[i]][[9]]] - AbsoluteTime[Data[[2]][[9]]],
        Tabelle absolute Zeit seit 1900           absolute Zeit seit 1900
    AbsoluteTime[Data[[i]][[3]]]}, {i, 2, Length[Data]}];
    absolute Zeit seit 1900                   Länge
Byvst = Table[{AbsoluteTime[Data[[i]][[9]]] - AbsoluteTime[Data[[2]][[9]]],
        Tabelle absolute Zeit seit 1900           absolute Zeit seit 1900
    AbsoluteTime[Data[[i]][[4]]]}, {i, 2, Length[Data]}];
    absolute Zeit seit 1900                   Länge
Bzvst = Table[{AbsoluteTime[Data[[i]][[9]]] - AbsoluteTime[Data[[2]][[9]]],
        Tabelle absolute Zeit seit 1900           absolute Zeit seit 1900
    AbsoluteTime[Data[[i]][[5]]]}, {i, 2, Length[Data]}];
    absolute Zeit seit 1900                   Länge

ListPlot[{Bvst, Bxvst, Byvst, Bzvst}, Joined → True]
listenbezogene Graphik                  verknüpft? wahr
```

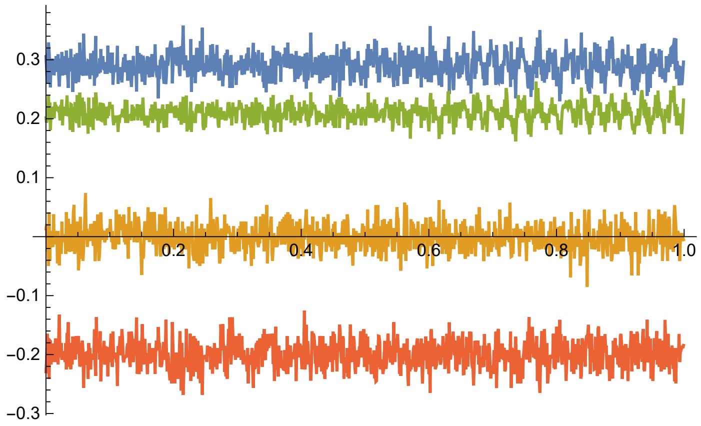


```
Start = 100;
Stop = 1000;
```

```
ListPlot[{Table[{Bvst[[i]][[1]], Bvst[[i]][[2]]}, {i, Start, Stop}],
  Table[{Bxvst[[i]][[1]], Bxvst[[i]][[2]]}, {i, Start, Stop}],
  Table[{Byvst[[i]][[1]], Byvst[[i]][[2]]}, {i, Start, Stop}],
  Table[{Bzvst[[i]][[1]], Bzvst[[i]][[2]]}, {i, Start, Stop}]}, Joined → True]
```

listenbezo… Tabelle Tabelle Tabelle Tabelle verknüpft? wahr

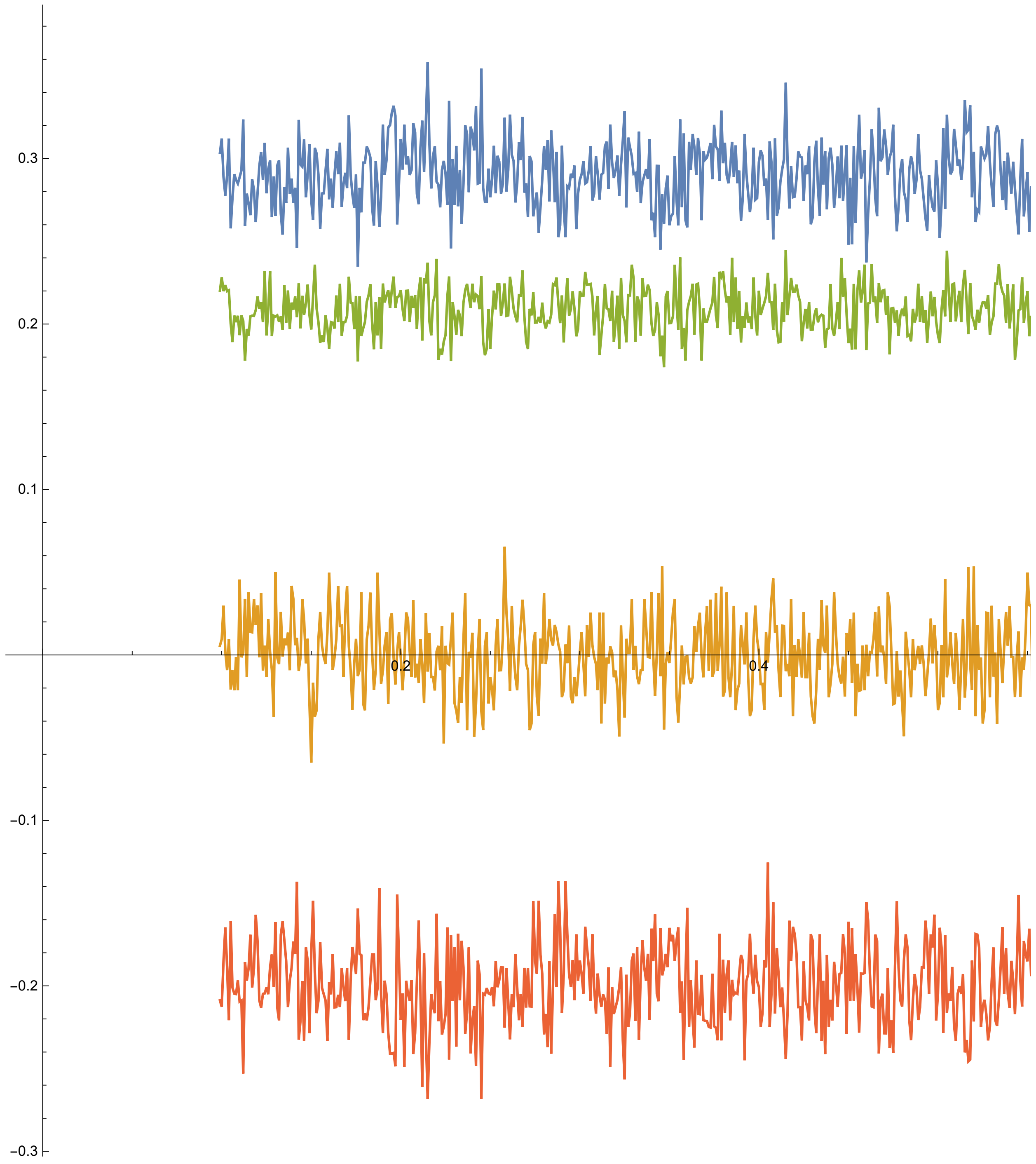

```
ListPlot[{Table[{Bvst[[i]][[1]], Bvst[[i]][[2]]}, {i, Start, Start + 50}],
  Table[{Bxvst[[i]][[1]], Bxvst[[i]][[2]]}, {i, Start, Start + 50}],
  Table[{Byvst[[i]][[1]], Byvst[[i]][[2]]}, {i, Start, Start + 50}],
  Table[{Bzvst[[i]][[1]], Bzvst[[i]][[2]]}, {i, Start, Start + 50}]}, Joined → True]
```

listenbezo··· Tabelle Tabelle Tabelle Tabelle verknüpft? wahr

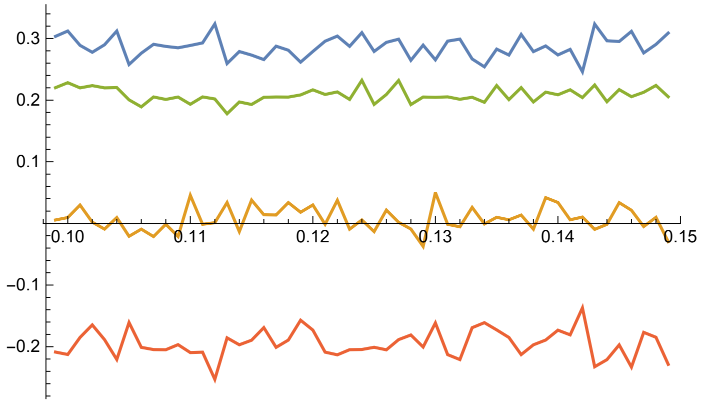


```
TableForm[{Mean[Table[Bvst[[i]][[2]], {i, Start, Stop}]],
  Mean[Table[Bxvst[[i]][[2]], {i, Start, Stop}]],
  Mean[Table[Byvst[[i]][[2]], {i, Start, Stop}]],
  Mean[Table[Bzvst[[i]][[2]], {i, Start, Stop}]]}, TableDirections → Row]
```

Tabellendars··· arit··· Tabelle arit··· Tabelle arit··· Tabelle arit··· Tabelle Richtung der Tabellen··· Zeile

0.290548	−0.00119047	0.209161	−0.199484

```
TableForm[Table[{(Bvst[[i]][[1]] - Bvst[[Start]][[1]]) * 1000, Bvst[[i]][[2]],
Tabellendar·· Tabelle
   Bxvst[[i]][[2]], Byvst[[i]][[2]], Bzvst[[i]][[2]]}, {i, Start, Start + 50}]]
```

0.	0.303475	0.005591	0.220221	-0.208731
0.999928	0.31213	0.009541	0.228285	-0.212649
1.99986	0.288889	0.029837	0.219883	-0.184982
2.99978	0.277635	0.00204	0.223544	-0.164636
4.00019	0.289831	-0.009202	0.219897	-0.18858
5.00011	0.312104	0.009467	0.220409	-0.22077
6.00004	0.2578	-0.020894	0.200458	-0.160752
6.99997	0.276154	-0.009294	0.189084	-0.201052
7.9999	0.290567	-0.021351	0.205129	-0.204683
8.99982	0.287174	-0.001348	0.201153	-0.20495
9.99975	0.284895	-0.021269	0.205007	-0.196684
11.0002	0.288701	0.045616	0.193271	-0.209555
12.0001	0.292829	-0.001394	0.205214	-0.208888
13.	0.32366	0.001157	0.20189	-0.252973
13.9999	0.259355	0.03388	0.177892	-0.185665
14.9999	0.278849	-0.013261	0.197018	-0.196889
15.9998	0.273064	0.037823	0.192956	-0.189476
16.9997	0.265789	0.014018	0.204621	-0.169049
18.0001	0.287534	0.013688	0.20511	-0.201044
19.0001	0.281005	0.033811	0.20495	-0.189252
20.	0.261603	0.018137	0.208442	-0.157031
20.9999	0.278919	0.029964	0.2167	-0.17303
21.9998	0.295603	-0.001398	0.209214	-0.208827
22.9998	0.303901	0.037555	0.21332	-0.213167
24.0002	0.287258	-0.009348	0.201144	-0.204868
25.0001	0.309466	0.00562	0.232158	-0.204548
26.	0.278993	-0.013298	0.193079	-0.200949
27.	0.293728	0.021642	0.20918	-0.205064
27.9999	0.298866	0.001785	0.231909	-0.18851
28.9998	0.264585	-0.009092	0.192779	-0.180994
29.9997	0.289214	-0.037309	0.205049	-0.200519
31.0001	0.265338	0.050099	0.204542	-0.161421
32.0001	0.295738	-0.001435	0.205275	-0.212887
33.	0.29898	-0.005513	0.201393	-0.220906
33.9999	0.266781	0.026017	0.204635	-0.169173
34.9998	0.254033	-0.00089	0.196482	-0.161018
35.9998	0.282774	0.009957	0.223675	-0.172717
37.0002	0.273152	0.005858	0.200856	-0.185025
38.0001	0.306596	0.013549	0.220291	-0.212813
39.	0.27872	-0.009261	0.197022	-0.19693
40.	0.287924	0.041802	0.212959	-0.189212
40.9999	0.273328	0.033972	0.208706	-0.173193
41.9998	0.28234	0.005882	0.216794	-0.180782
42.9997	0.246065	0.010348	0.204128	-0.137014
44.0001	0.323398	-0.00966	0.224569	-0.232511
45.0001	0.296333	-0.001509	0.197398	-0.221008
46.	0.295114	0.033716	0.217071	-0.197067
46.9999	0.31157	0.021358	0.205608	-0.233121
47.9999	0.276603	-0.005072	0.21272	-0.17673
48.9998	0.290395	0.009834	0.223858	-0.184715
50.0002	0.308799	-0.029598	0.205486	-0.228597

(-5,0,0) Blue offset -16A White Offset -49A RMF off

```
Data = Import[NotebookDirectory[] <> "x-5_y0_z0_B-16_W-49_RMFoff.txt", "Table"];
       [importi··· [Notebook–Verzeichnis                                  [Tabelle

Bvst = Table[{AbsoluteTime[Data[[i]][[9]]] - AbsoluteTime[Data[[2]][[9]]],
       [Tabelle [absolute Zeit seit 1900       [absolute Zeit seit 1900
    AbsoluteTime[Data[[i]][[2]]]}, {i, 2, Length[Data]}];
    [absolute Zeit seit 1900                 [Länge
Bxvst = Table[{AbsoluteTime[Data[[i]][[9]]] - AbsoluteTime[Data[[2]][[9]]],
        [Tabelle [absolute Zeit seit 1900       [absolute Zeit seit 1900
    AbsoluteTime[Data[[i]][[3]]]}, {i, 2, Length[Data]}];
    [absolute Zeit seit 1900                 [Länge
Byvst = Table[{AbsoluteTime[Data[[i]][[9]]] - AbsoluteTime[Data[[2]][[9]]],
        [Tabelle [absolute Zeit seit 1900       [absolute Zeit seit 1900
    AbsoluteTime[Data[[i]][[4]]]}, {i, 2, Length[Data]}];
    [absolute Zeit seit 1900                 [Länge
Bzvst = Table[{AbsoluteTime[Data[[i]][[9]]] - AbsoluteTime[Data[[2]][[9]]],
        [Tabelle [absolute Zeit seit 1900       [absolute Zeit seit 1900
    AbsoluteTime[Data[[i]][[5]]]}, {i, 2, Length[Data]}];
    [absolute Zeit seit 1900                 [Länge
```

```
ListPlot[{Bvst, Bxvst, Byvst, Bzvst}, Joined → True]
[listenbezogene Graphik                 [verknüpft? [wahr
```

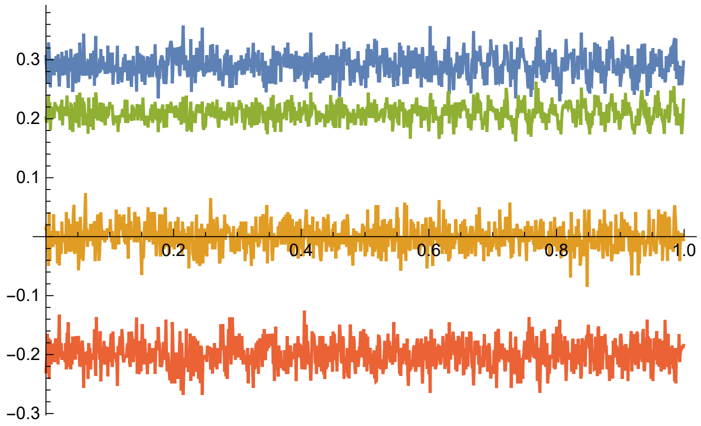


```
Start = 100;
Stop = 1000;
```

```
ListPlot[{Table[{Bvst[[i]][[1]], Bvst[[i]][[2]]}, {i, Start, Stop}],
  Table[{Bxvst[[i]][[1]], Bxvst[[i]][[2]]}, {i, Start, Stop}],
  Table[{Byvst[[i]][[1]], Byvst[[i]][[2]]}, {i, Start, Stop}],
  Table[{Bzvst[[i]][[1]], Bzvst[[i]][[2]]}, {i, Start, Stop}]}, Joined → True]
```

listenbezo··· Tabelle
Tabelle
Tabelle
Tabelle verknüpft? wahr

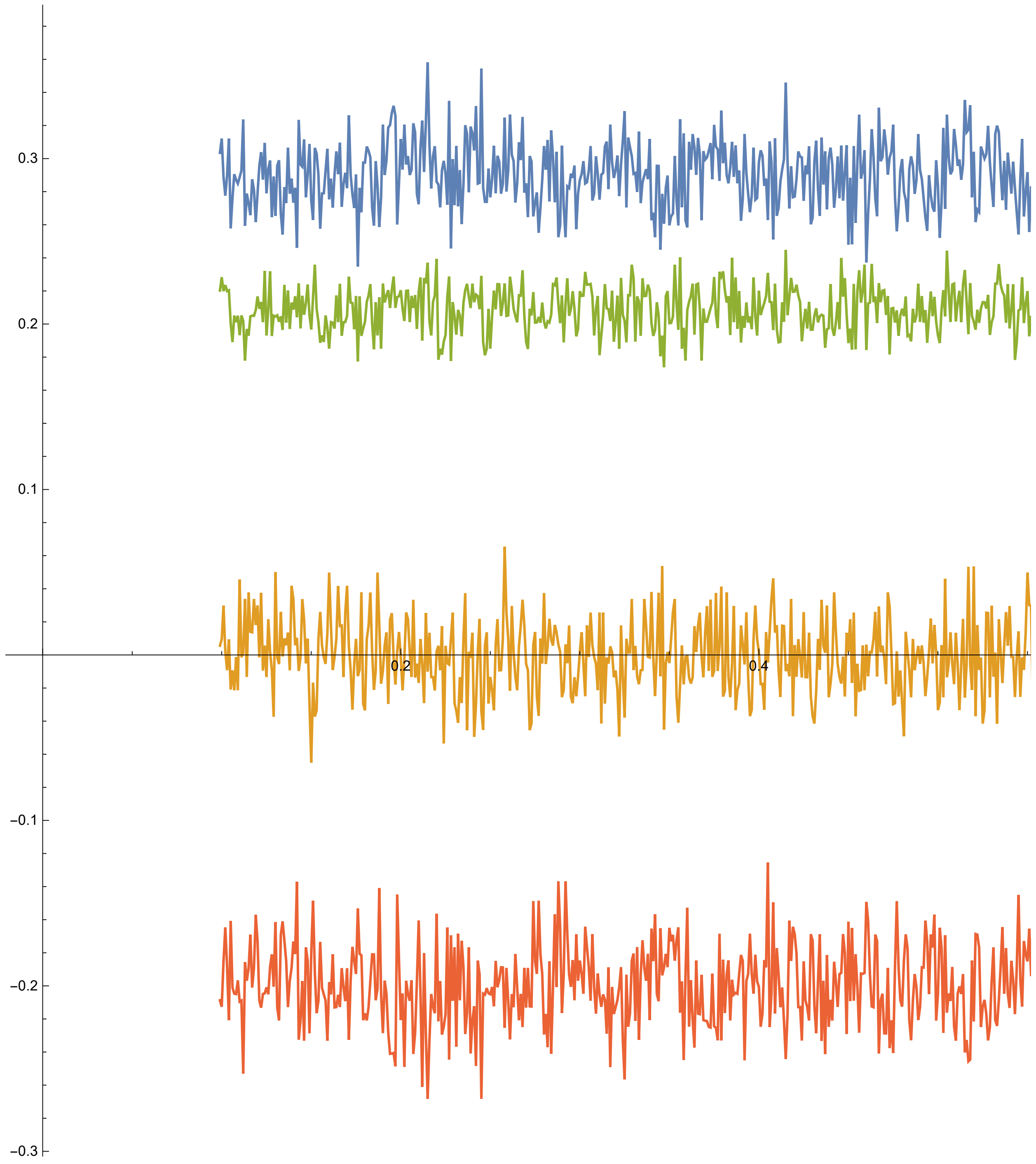

```
ListPlot[{Table[{Bvst[[i]][[1]], Bvst[[i]][[2]]}, {i, Start, Start + 50}],
  Table[{Bxvst[[i]][[1]], Bxvst[[i]][[2]]}, {i, Start, Start + 50}],
  Table[{Byvst[[i]][[1]], Byvst[[i]][[2]]}, {i, Start, Start + 50}],
  Table[{Bzvst[[i]][[1]], Bzvst[[i]][[2]]}, {i, Start, Start + 50}]}, Joined → True]
```

listenbezo··· Tabelle Tabelle Tabelle Tabelle verknüpft? wahr

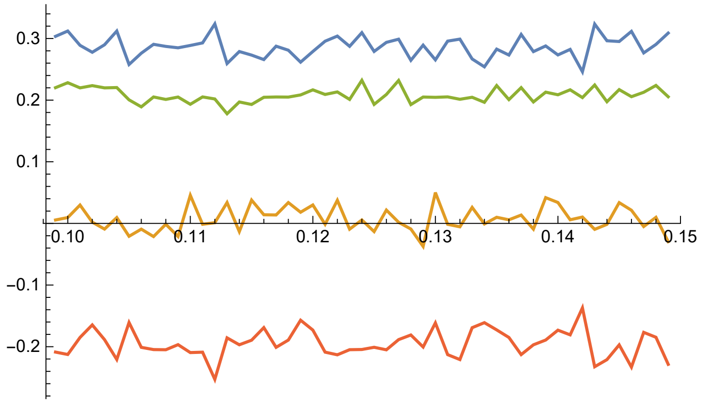


```
TableForm[{Mean[Table[Bvst[[i]][[2]], {i, Start, Stop}]],
  Mean[Table[Bxvst[[i]][[2]], {i, Start, Stop}]],
  Mean[Table[Byvst[[i]][[2]], {i, Start, Stop}]],
  Mean[Table[Bzvst[[i]][[2]], {i, Start, Stop}]]}, TableDirections → Row]
```

Tabellendars··· arit··· Tabelle arit··· Tabelle arit··· Tabelle arit··· Tabelle Richtung der Tabellen··· Zeile

0.290548	-0.00119047	0.209161	-0.199484

```
TableForm[Table[{(Bvst[[i]][[1]] - Bvst[[Start]][[1]]) * 1000, Bvst[[i]][[2]],
  Bxvst[[i]][[2]], Byvst[[i]][[2]], Bzvst[[i]][[2]]}, {i, Start, Start + 50}]]
```

Tabellendar·· Tabelle

0.	0.303475	0.005591	0.220221	-0.208731
0.999928	0.31213	0.009541	0.228285	-0.212649
1.99986	0.288889	0.029837	0.219883	-0.184982
2.99978	0.277635	0.00204	0.223544	-0.164636
4.00019	0.289831	-0.009202	0.219897	-0.18858
5.00011	0.312104	0.009467	0.220409	-0.22077
6.00004	0.2578	-0.020894	0.200458	-0.160752
6.99997	0.276154	-0.009294	0.189084	-0.201052
7.9999	0.290567	-0.021351	0.205129	-0.204683
8.99982	0.287174	-0.001348	0.201153	-0.20495
9.99975	0.284895	-0.021269	0.205007	-0.196684
11.0002	0.288701	0.045616	0.193271	-0.209555
12.0001	0.292829	-0.001394	0.205214	-0.208888
13.	0.32366	0.001157	0.20189	-0.252973
13.9999	0.259355	0.03388	0.177892	-0.185665
14.9999	0.278849	-0.013261	0.197018	-0.196889
15.9998	0.273064	0.037823	0.192956	-0.189476
16.9997	0.265789	0.014018	0.204621	-0.169049
18.0001	0.287534	0.013688	0.20511	-0.201044
19.0001	0.281005	0.033811	0.20495	-0.189252
20.	0.261603	0.018137	0.208442	-0.157031
20.9999	0.278919	0.029964	0.2167	-0.17303
21.9998	0.295603	-0.001398	0.209214	-0.208827
22.9998	0.303901	0.037555	0.21332	-0.213167
24.0002	0.287258	-0.009348	0.201144	-0.204868
25.0001	0.309466	0.00562	0.232158	-0.204548
26.	0.278993	-0.013298	0.193079	-0.200949
27.	0.293728	0.021642	0.20918	-0.205064
27.9999	0.298866	0.001785	0.231909	-0.18851
28.9998	0.264585	-0.009092	0.192779	-0.180994
29.9997	0.289214	-0.037309	0.205049	-0.200519
31.0001	0.265338	0.050099	0.204542	-0.161421
32.0001	0.295738	-0.001435	0.205275	-0.212887
33.	0.29898	-0.005513	0.201393	-0.220906
33.9999	0.266781	0.026017	0.204635	-0.169173
34.9998	0.254033	-0.00089	0.196482	-0.161018
35.9998	0.282774	0.009957	0.223675	-0.172717
37.0002	0.273152	0.005858	0.200856	-0.185025
38.0001	0.306596	0.013549	0.220291	-0.212813
39.	0.27872	-0.009261	0.197022	-0.19693
40.	0.287924	0.041802	0.212959	-0.189212
40.9999	0.273328	0.033972	0.208706	-0.173193
41.9998	0.28234	0.005882	0.216794	-0.180782
42.9997	0.246065	0.010348	0.204128	-0.137014
44.0001	0.323398	-0.00966	0.224569	-0.232511
45.0001	0.296333	-0.001509	0.197398	-0.221008
46.	0.295114	0.033716	0.217071	-0.197067
46.9999	0.31157	0.021358	0.205608	-0.233121
47.9999	0.276603	-0.005072	0.21272	-0.17673
48.9998	0.290395	0.009834	0.223858	-0.184715
50.0002	0.308799	-0.029598	0.205486	-0.228597

(-5,0,0) Blue offset -16A White Offset -49A RMF on

```
Data = Import[NotebookDirectory[] <> "x-5_y0_z0_B-16_W-49_RMFon.txt", "Table"];
       importi··· Notebook-Verzeichnis                                      Tabelle

Bvst = Table[{AbsoluteTime[Data[[i]][[9]]] - AbsoluteTime[Data[[2]][[9]]],
       Tabelle  absolute Zeit seit 1900         absolute Zeit seit 1900
    AbsoluteTime[Data[[i]][[2]]]}, {i, 2, Length[Data]}];
    absolute Zeit seit 1900                   Länge
Bxvst = Table[{AbsoluteTime[Data[[i]][[9]]] - AbsoluteTime[Data[[2]][[9]]],
        Tabelle  absolute Zeit seit 1900         absolute Zeit seit 1900
    AbsoluteTime[Data[[i]][[3]]]}, {i, 2, Length[Data]}];
    absolute Zeit seit 1900                   Länge
Byvst = Table[{AbsoluteTime[Data[[i]][[9]]] - AbsoluteTime[Data[[2]][[9]]],
        Tabelle  absolute Zeit seit 1900         absolute Zeit seit 1900
    AbsoluteTime[Data[[i]][[4]]]}, {i, 2, Length[Data]}];
    absolute Zeit seit 1900                   Länge
Bzvst = Table[{AbsoluteTime[Data[[i]][[9]]] - AbsoluteTime[Data[[2]][[9]]],
        Tabelle  absolute Zeit seit 1900         absolute Zeit seit 1900
    AbsoluteTime[Data[[i]][[5]]]}, {i, 2, Length[Data]}];
    absolute Zeit seit 1900                   Länge
```

```
ListPlot[{Bvst, Bxvst, Byvst, Bzvst}, Joined → True]
listenbezogene Graphik                verknüpft? wahr
```

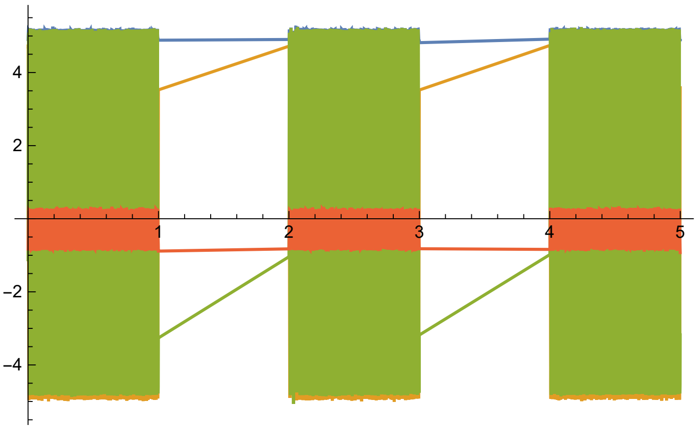


```
Start = 100;
Stop = 1000;
```

```
ListPlot[{Table[{Bvst[[i]][[1]], Bvst[[i]][[2]]}, {i, Start, Stop}],
  Table[{Bxvst[[i]][[1]], Bxvst[[i]][[2]]}, {i, Start, Stop}],
  Table[{Byvst[[i]][[1]], Byvst[[i]][[2]]}, {i, Start, Stop}],
  Table[{Bzvst[[i]][[1]], Bzvst[[i]][[2]]}, {i, Start, Stop}]}, Joined → True]
```

listenbezo··· Tabelle Tabelle Tabelle Tabelle verknüpft? wahr

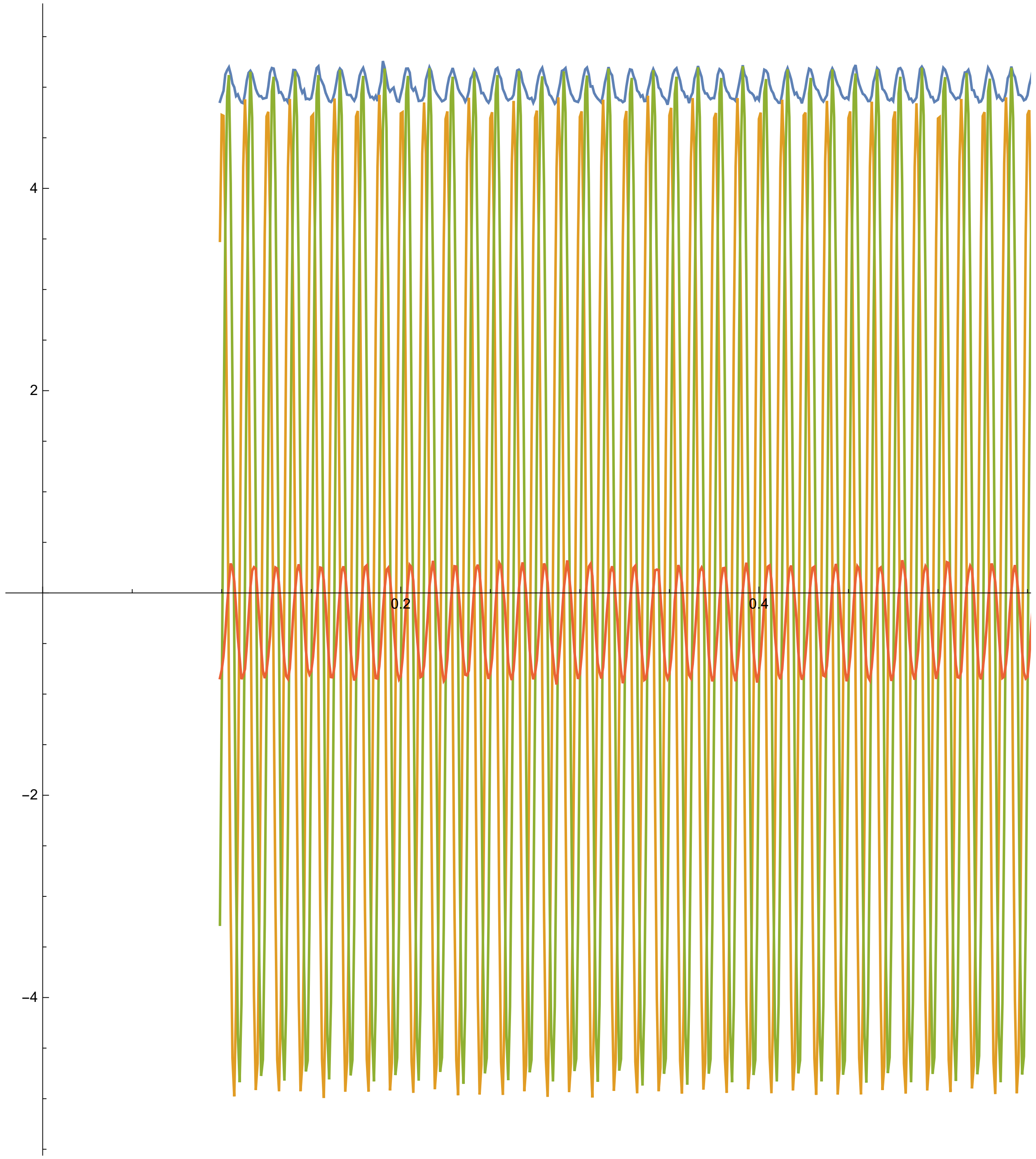

```
ListPlot[{Table[{Bvst[[i]][[1]], Bvst[[i]][[2]]}, {i, Start, Start + 50}],
  Table[{Bxvst[[i]][[1]], Bxvst[[i]][[2]]}, {i, Start, Start + 50}],
  Table[{Byvst[[i]][[1]], Byvst[[i]][[2]]}, {i, Start, Start + 50}],
  Table[{Bzvst[[i]][[1]], Bzvst[[i]][[2]]}, {i, Start, Start + 50}]}, Joined → True]
```

listenbezo··· Tabelle
Tabelle
Tabelle
Tabelle verknüpft? wahr

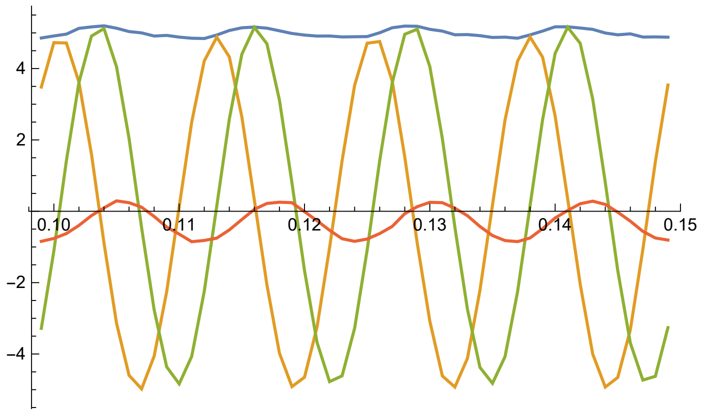


```
TableForm[{Mean[Table[Bvst[[i]][[2]], {i, Start, Stop}]],
  Mean[Table[Bxvst[[i]][[2]], {i, Start, Stop}]],
  Mean[Table[Byvst[[i]][[2]], {i, Start, Stop}]],
  Mean[Table[Bzvst[[i]][[2]], {i, Start, Stop}]]}, TableDirections → Row]
```

Tabellendars··· arit··· Tabelle
arit··· Tabelle
arit··· Tabelle
arit··· Tabelle Richtung der Tabellen··· Zeile

5.00178 −0.0460096 0.168156 −0.289276

```
TableForm[Table[{(Bvst[[i]][[1]] - Bvst[[Start]][[1]]) * 1000, Bvst[[i]][[2]],
Tabellendar·· Tabelle
  Bxvst[[i]][[2]], Byvst[[i]][[2]], Bzvst[[i]][[2]]}, {i, Start, Start + 50}]]
```

0.	4.85668	3.48141	-3.27994	-0.84211
0.999928	4.9139	4.72573	-1.11063	-0.761806
1.99986	4.96464	4.71715	1.41656	-0.624112
2.99978	5.12635	3.62084	3.6072	-0.396326
4.00019	5.16718	1.60501	4.91009	-0.121606
5.00011	5.19739	-0.887151	5.12025	0.094332
6.00004	5.1307	-3.13397	4.05179	0.292218
6.99997	5.0369	-4.59819	2.04183	0.240619
7.9999	4.99987	-4.97755	-0.457923	0.114346
8.99982	4.91162	-4.06343	-2.7553	-0.144196
10.0002	4.92922	-2.25849	-4.35909	-0.44134
11.0002	4.88147	0.126156	-4.83766	-0.640265
12.0001	4.84957	2.50219	-4.06589	-0.852015
13.	4.84178	4.21147	-2.24444	-0.817823
13.9999	4.9402	4.88034	0.148531	-0.752175
14.9999	5.06686	4.32779	2.58274	-0.522277
15.9998	5.14235	2.64854	4.40177	-0.231144
17.0002	5.1626	0.308464	5.15309	0.054501
18.0001	5.13157	-2.05943	4.69509	0.218949
19.0001	5.05825	-3.97529	3.11739	0.25462
20.	4.98351	-4.91373	0.795357	0.240843
20.9999	4.93868	-4.65139	-1.65982	-0.010387
21.9998	4.91047	-3.24652	-3.67476	-0.262676
23.0002	4.91197	-1.02387	-4.77448	-0.532426
24.0002	4.88648	1.41863	-4.61233	-0.769172
25.0001	4.89145	3.5334	-3.27588	-0.842584
26.	4.89629	4.71054	-1.08641	-0.777282
27.	5.00385	4.75714	1.4206	-0.624463
27.9999	5.14365	3.64855	3.60067	-0.424717
28.9998	5.18999	1.5225	4.96119	-0.067966
30.0002	5.18454	-0.902773	5.1037	0.129247
31.0001	5.0991	-3.07939	4.05646	0.25172
32.0001	5.05029	-4.61419	2.03881	0.240738
33.	4.9467	-4.92585	-0.445435	0.085998
33.9999	4.95199	-4.12324	-2.73968	-0.123338
34.9998	4.92173	-2.21531	-4.37428	-0.426015
35.9998	4.87174	0.169725	-4.82099	-0.680467
37.0002	4.88001	2.55752	-4.07431	-0.820709
38.0001	4.85031	4.20716	-2.25895	-0.850006
39.	4.9439	4.88437	0.159474	-0.748049
40.	5.05038	4.32018	2.57018	-0.486384
40.9999	5.17027	2.66896	4.42414	-0.188005
41.9998	5.16934	0.347128	5.15762	0.022166
43.0002	5.1364	-2.04452	4.70723	0.210979
44.0001	5.09792	-4.00303	3.14392	0.283316
45.0001	4.9963	-4.92624	0.811074	0.193204
46.	4.94526	-4.65554	-1.66758	-0.026466
46.9999	4.97301	-3.32165	-3.69062	-0.277146
47.9999	4.88265	-1.06823	-4.73106	-0.562311
48.9998	4.88723	1.39485	-4.62366	-0.749097
50.0002	4.87882	3.53776	-3.26142	-0.806402

(5,0,0) Blue offset 17A White Offset 49A RMF off

```
Data = Import[NotebookDirectory[] <> "x5_y0_z0_B17_W49_RMFoff.txt", "Table"];
       importi··· Notebook-Verzeichnis                             Tabelle

Bvst = Table[{AbsoluteTime[Data[[i]][[9]]] - AbsoluteTime[Data[[2]][[9]]],
       Tabelle absolute Zeit seit 1900            absolute Zeit seit 1900
    AbsoluteTime[Data[[i]][[2]]]}, {i, 2, Length[Data]}];
    absolute Zeit seit 1900                Länge
Bxvst = Table[{AbsoluteTime[Data[[i]][[9]]] - AbsoluteTime[Data[[2]][[9]]],
        Tabelle absolute Zeit seit 1900            absolute Zeit seit 1900
    AbsoluteTime[Data[[i]][[3]]]}, {i, 2, Length[Data]}];
    absolute Zeit seit 1900                Länge
Byvst = Table[{AbsoluteTime[Data[[i]][[9]]] - AbsoluteTime[Data[[2]][[9]]],
        Tabelle absolute Zeit seit 1900            absolute Zeit seit 1900
    AbsoluteTime[Data[[i]][[4]]]}, {i, 2, Length[Data]}];
    absolute Zeit seit 1900                Länge
Bzvst = Table[{AbsoluteTime[Data[[i]][[9]]] - AbsoluteTime[Data[[2]][[9]]],
        Tabelle absolute Zeit seit 1900            absolute Zeit seit 1900
    AbsoluteTime[Data[[i]][[5]]]}, {i, 2, Length[Data]}];
    absolute Zeit seit 1900                Länge
```

```
ListPlot[{Bvst, Bxvst, Byvst, Bzvst}, Joined → True]
listenbezogene Graphik                verknüpft? wahr
```

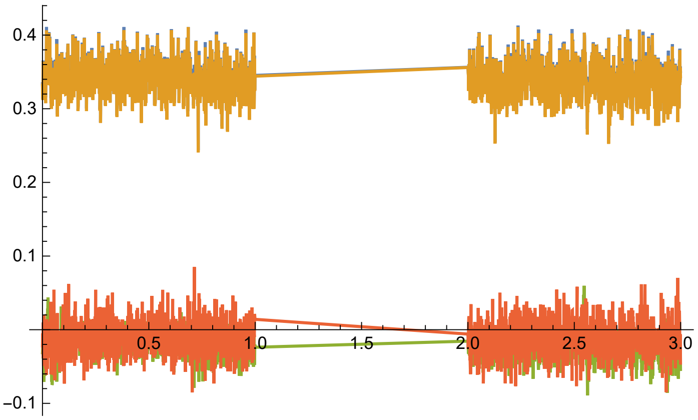


```
Start = 100;
Stop = 1000;
```

```
ListPlot[{Table[{Bvst[[i]][[1]], Bvst[[i]][[2]]}, {i, Start, Stop}],
  Table[{Bxvst[[i]][[1]], Bxvst[[i]][[2]]}, {i, Start, Stop}],
  Table[{Byvst[[i]][[1]], Byvst[[i]][[2]]}, {i, Start, Stop}],
  Table[{Bzvst[[i]][[1]], Bzvst[[i]][[2]]}, {i, Start, Stop}]}, Joined → True]
```

listenbezo··· Tabelle Tabelle Tabelle Tabelle verknüpft? wahr

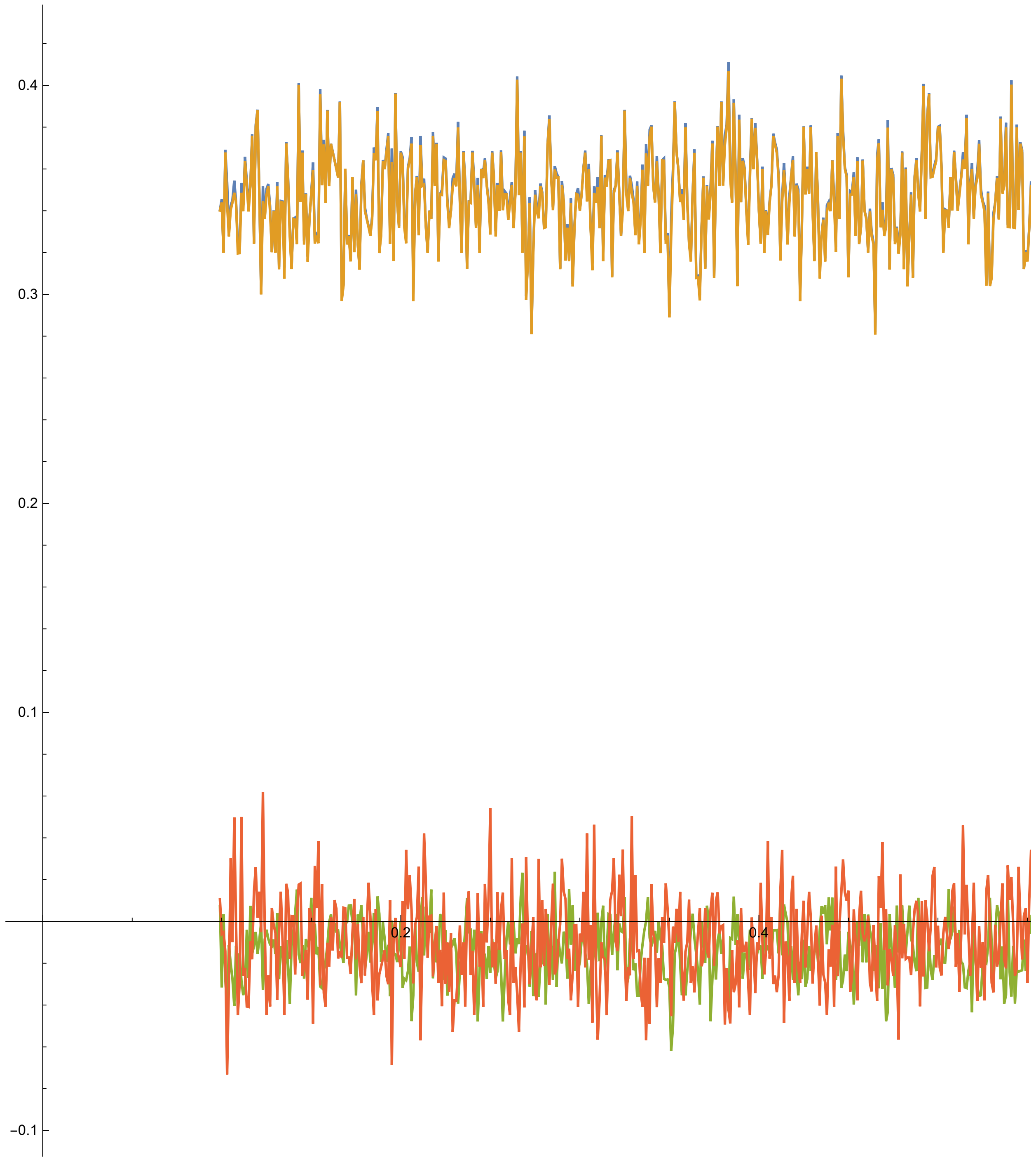

```
ListPlot[{Table[{Bvst[[i]][[1]], Bvst[[i]][[2]]}, {i, Start, Start + 50}],
  Table[{Bxvst[[i]][[1]], Bxvst[[i]][[2]]}, {i, Start, Start + 50}],
  Table[{Byvst[[i]][[1]], Byvst[[i]][[2]]}, {i, Start, Start + 50}],
  Table[{Bzvst[[i]][[1]], Bzvst[[i]][[2]]}, {i, Start, Start + 50}]}, Joined → True]
```

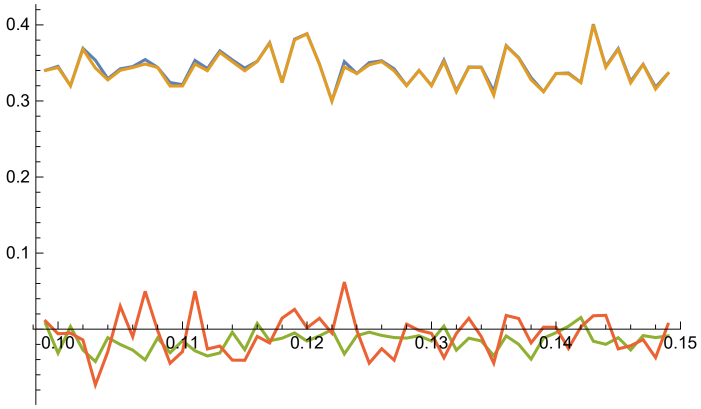


```
TableForm[{Mean[Table[Bvst[[i]][[2]], {i, Start, Stop}]],
  Mean[Table[Bxvst[[i]][[2]], {i, Start, Stop}]],
  Mean[Table[Byvst[[i]][[2]], {i, Start, Stop}]],
  Mean[Table[Bzvst[[i]][[2]], {i, Start, Stop}]]}, TableDirections → Row]
```

0.344046 0.342205 -0.0188798 -0.00988233

```
TableForm[Table[{(Bvst[[i]][[1]] - Bvst[[Start]][[1]]) * 1000, Bvst[[i]][[2]],
Tabellendar·· Tabelle
   Bxvst[[i]][[2]], Byvst[[i]][[2]], Bzvst[[i]][[2]]}, {i, Start, Start + 50}]]
```

0.	0.34036	0.340119	0.007189	0.010605
0.999928	0.345491	0.343994	-0.031558	-0.006029
1.99986	0.32002	0.319959	0.00341	-0.005248
3.00026	0.369199	0.367906	-0.027407	-0.014214
4.00019	0.3536	0.343315	-0.042533	-0.073201
5.00011	0.329248	0.327727	-0.011213	-0.029555
6.00004	0.342281	0.340353	-0.020113	0.030189
6.99997	0.34519	0.343948	-0.027497	-0.009968
7.9999	0.354429	0.348579	-0.040407	0.049798
8.99982	0.344215	0.344015	-0.011621	-0.001724
10.0002	0.324199	0.319593	-0.030991	-0.044776
11.0002	0.321453	0.319731	-0.015222	-0.029534
12.0001	0.353276	0.348567	-0.028408	0.049981
13.	0.342608	0.339792	-0.035257	-0.026046
13.9999	0.365847	0.363828	-0.03129	-0.022232
14.9999	0.353975	0.351605	-0.004017	-0.040693
16.0003	0.343152	0.339629	-0.027028	-0.040921
17.0002	0.352121	0.351912	0.007508	-0.009515
18.0001	0.376601	0.375852	-0.015338	-0.018112
19.0001	0.324721	0.324181	-0.011889	0.014479
20.	0.381215	0.380294	-0.005007	0.026007
20.9999	0.388376	0.388057	-0.015629	0.001761
21.9998	0.348581	0.348176	-0.008861	0.014278
23.0002	0.300008	0.299964	-0.000613	-0.005103
24.0002	0.351733	0.344695	-0.032596	0.061959
25.0001	0.336126	0.336012	-0.008631	-0.001596
26.	0.350443	0.347564	-0.003961	-0.044651
27.	0.352802	0.351764	-0.008245	-0.025757
27.9999	0.342218	0.339613	-0.01103	-0.040677
29.0003	0.320381	0.320098	-0.011772	0.006521
30.0002	0.340125	0.340012	-0.008626	-0.001637
31.0001	0.320406	0.319979	-0.015588	-0.005538
32.0001	0.353651	0.351627	0.003936	-0.037571
33.	0.31326	0.311991	-0.027596	-0.005639
33.9999	0.34468	0.344179	-0.011865	0.014273
34.9998	0.344424	0.343936	-0.015498	-0.009784
36.0003	0.312795	0.307598	-0.035004	-0.044713
37.0002	0.372759	0.372216	-0.008893	0.01803
38.0001	0.357015	0.356187	-0.01985	0.014027
39.	0.330726	0.327879	-0.039392	-0.017985
40.	0.312288	0.312057	-0.01172	0.002604
40.9999	0.336091	0.336049	-0.004692	0.002464
41.9998	0.336733	0.335752	0.003734	-0.025409
43.0002	0.324403	0.324029	0.015291	0.002893
44.0001	0.400924	0.400222	-0.015859	0.017635
45.0001	0.345282	0.344229	-0.019925	0.01815
46.	0.368852	0.367766	-0.011226	-0.025967
46.9999	0.325705	0.323826	-0.027338	-0.02176
47.9999	0.34826	0.347887	-0.008433	-0.013718
49.0003	0.31805	0.315645	-0.011105	-0.03743
50.0002	0.336269	0.336094	-0.008753	0.006402

(-5,0,0) Blue offset 17A White Offset 49A RMF off

```
Data = Import[NotebookDirectory[] <> "x-5_y0_z0_B17_W49_RMFoff.txt", "Table"];
       importi… Notebook-Verzeichnis                                  Tabelle

Bvst = Table[{AbsoluteTime[Data[[i]][[9]]] - AbsoluteTime[Data[[2]][[9]]],
       Tabelle absolute Zeit seit 1900           absolute Zeit seit 1900
    AbsoluteTime[Data[[i]][[2]]]}, {i, 2, Length[Data]}];
    absolute Zeit seit 1900                   Länge
Bxvst = Table[{AbsoluteTime[Data[[i]][[9]]] - AbsoluteTime[Data[[2]][[9]]],
        Tabelle absolute Zeit seit 1900           absolute Zeit seit 1900
    AbsoluteTime[Data[[i]][[3]]]}, {i, 2, Length[Data]}];
    absolute Zeit seit 1900                   Länge
Byvst = Table[{AbsoluteTime[Data[[i]][[9]]] - AbsoluteTime[Data[[2]][[9]]],
        Tabelle absolute Zeit seit 1900           absolute Zeit seit 1900
    AbsoluteTime[Data[[i]][[4]]]}, {i, 2, Length[Data]}];
    absolute Zeit seit 1900                   Länge
Bzvst = Table[{AbsoluteTime[Data[[i]][[9]]] - AbsoluteTime[Data[[2]][[9]]],
        Tabelle absolute Zeit seit 1900           absolute Zeit seit 1900
    AbsoluteTime[Data[[i]][[5]]]}, {i, 2, Length[Data]}];
    absolute Zeit seit 1900                   Länge
```

```
ListPlot[{Bvst, Bxvst, Byvst, Bzvst}, Joined → True]
listenbezogene Graphik                   verknüpft? wahr
```

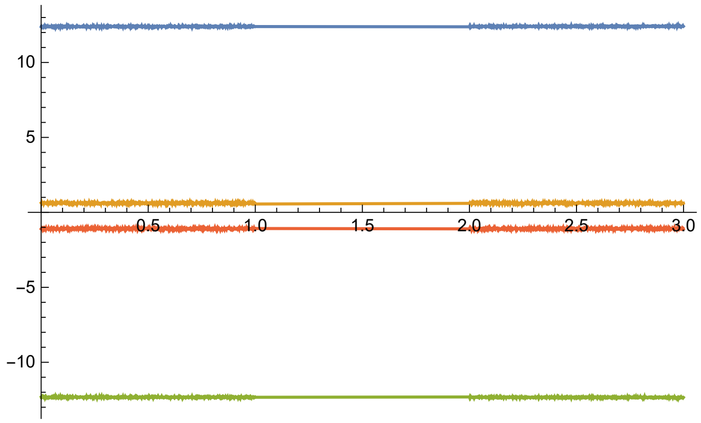


```
Start = 100;
Stop = 1000;
```

```
ListPlot[{Table[{Bvst[[i]][[1]], Bvst[[i]][[2]]}, {i, Start, Stop}],
  Table[{Bxvst[[i]][[1]], Bxvst[[i]][[2]]}, {i, Start, Stop}],
  Table[{Byvst[[i]][[1]], Byvst[[i]][[2]]}, {i, Start, Stop}],
  Table[{Bzvst[[i]][[1]], Bzvst[[i]][[2]]}, {i, Start, Stop}]}, Joined → True]
```

listenbezo⋯ Tabelle Tabelle Tabelle Tabelle verknüpft? wahr

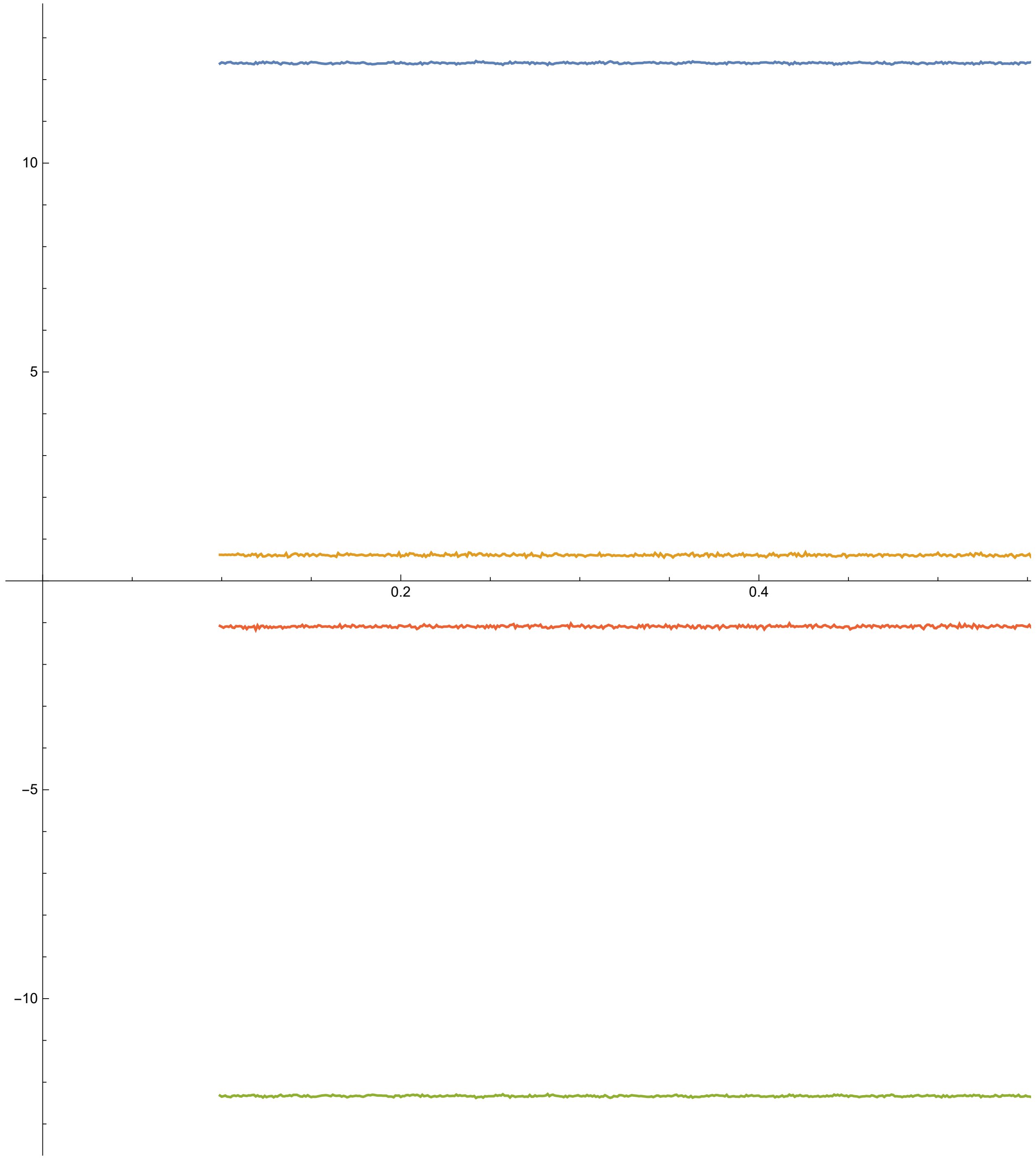

```
ListPlot[{Table[{Bvst[[i]][[1]], Bvst[[i]][[2]]}, {i, Start, Start + 50}],
  Table[{Bxvst[[i]][[1]], Bxvst[[i]][[2]]}, {i, Start, Start + 50}],
  Table[{Byvst[[i]][[1]], Byvst[[i]][[2]]}, {i, Start, Start + 50}],
  Table[{Bzvst[[i]][[1]], Bzvst[[i]][[2]]}, {i, Start, Start + 50}]}, Joined → True]
```

listenbezo··· Tabelle Tabelle Tabelle Tabelle verknüpft? wahr

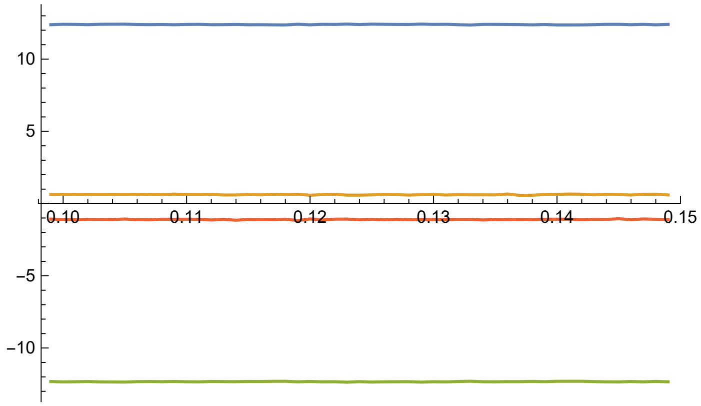


```
TableForm[{Mean[Table[Bvst[[i]][[2]], {i, Start, Stop}]],
  Mean[Table[Bxvst[[i]][[2]], {i, Start, Stop}]],
  Mean[Table[Byvst[[i]][[2]], {i, Start, Stop}]],
  Mean[Table[Bzvst[[i]][[2]], {i, Start, Stop}]]}, TableDirections → Row]
```

Tabellendars···arit··· Tabelle arit··· Tabelle arit··· Tabelle arit··· Tabelle Richtung der Tabellen··· Zeile

```
12.3967  0.615496  -12.3331  -1.09308
```

```
TableForm[Table[{(Bvst[[i]][[1]] - Bvst[[Start]][[1]]) * 1000, Bvst[[i]][[2]],
  Bxvst[[i]][[2]], Byvst[[i]][[2]], Bzvst[[i]][[2]]}, {i, Start, Start + 50}]]
```

Tabellendar·· Tabelle

0.	12.3821	0.624556	-12.3193	-1.0777
0.999928	12.4105	0.624378	-12.346	-1.09811
1.99986	12.4011	0.620119	-12.3347	-1.1219
2.99978	12.3835	0.628391	-12.3191	-1.09374
4.00019	12.4097	0.62046	-12.3462	-1.09007
5.00011	12.415	0.62834	-12.35	-1.10221
6.00004	12.4202	0.620679	-12.3585	-1.07026
6.99997	12.3934	0.632151	-12.3267	-1.1179
7.9999	12.3855	0.620061	-12.3186	-1.12565
8.99982	12.3944	0.624527	-12.3313	-1.08188
9.99975	12.3805	0.652427	-12.3151	-1.08993
11.0002	12.3986	0.628531	-12.3353	-1.08199
12.0001	12.4057	0.620456	-12.3422	-1.09001
13.	12.383	0.631933	-12.3144	-1.13771
13.9999	12.3857	0.592397	-12.3231	-1.09343
14.9999	12.3983	0.595797	-12.3302	-1.15258
15.9998	12.3797	0.620305	-12.315	-1.10159
17.0002	12.3835	0.604227	-12.3189	-1.10949
18.0001	12.3732	0.644254	-12.3069	-1.10572
19.0001	12.3665	0.624498	-12.3033	-1.08145
20.	12.4132	0.643599	-12.3409	-1.17324
20.9999	12.375	0.576678	-12.3156	-1.06515
21.9998	12.409	0.620003	-12.3415	-1.134
22.9998	12.3991	0.644571	-12.3353	-1.07815
24.0002	12.4268	0.584648	-12.3664	-1.07401
25.0001	12.3911	0.584154	-12.3268	-1.1174
26.	12.4204	0.600515	-12.3582	-1.08605
27.	12.4088	0.628085	-12.3416	-1.12608
27.9999	12.3988	0.616408	-12.3351	-1.09386
28.9998	12.3959	0.588075	-12.3306	-1.1255
30.0002	12.4258	0.612395	-12.362	-1.09823
31.0001	12.4009	0.628201	-12.3348	-1.11398
32.0001	12.4098	0.592256	-12.3459	-1.10978
33.	12.3827	0.612392	-12.3191	-1.09357
33.9999	12.3631	0.604414	-12.3002	-1.0892
34.9998	12.4015	0.603955	-12.3344	-1.13773
35.9998	12.4026	0.60033	-12.339	-1.10175
37.0002	12.3949	0.66015	-12.3267	-1.11819
38.0001	12.389	0.56532	-12.327	-1.10121
39.	12.3744	0.58022	-12.3109	-1.10912
40.	12.3945	0.620486	-12.3312	-1.08584
40.9999	12.3673	0.640497	-12.3033	-1.08162
41.9998	12.3681	0.656497	-12.3032	-1.08178
42.9997	12.3701	0.648167	-12.3028	-1.1137
44.0001	12.3857	0.604479	-12.3232	-1.08555
45.0001	12.4066	0.632414	-12.3421	-1.09413
46.	12.4072	0.620831	-12.3467	-1.05408
46.9999	12.3829	0.592228	-12.3189	-1.10937
47.9999	12.4052	0.644702	-12.3425	-1.06626
48.9998	12.376	0.648464	-12.3112	-1.08582
50.0002	12.403	0.596248	-12.3389	-1.10971

(5,0,0) Blue offset 17A White Offset 49A RMF on

```
Data = Import[NotebookDirectory[] <> "x5_y0_z0_B17_W49_RMFon.txt", "Table"];
       importi··· Notebook-Verzeichnis                             Tabelle

Bvst = Table[{AbsoluteTime[Data[[i]][[9]]] - AbsoluteTime[Data[[2]][[9]]],
       Tabelle  absolute Zeit seit 1900          absolute Zeit seit 1900
    AbsoluteTime[Data[[i]][[2]]]}, {i, 2, Length[Data]}];
    absolute Zeit seit 1900                   Länge
Bxvst = Table[{AbsoluteTime[Data[[i]][[9]]] - AbsoluteTime[Data[[2]][[9]]],
        Tabelle  absolute Zeit seit 1900          absolute Zeit seit 1900
    AbsoluteTime[Data[[i]][[3]]]}, {i, 2, Length[Data]}];
    absolute Zeit seit 1900                   Länge
Byvst = Table[{AbsoluteTime[Data[[i]][[9]]] - AbsoluteTime[Data[[2]][[9]]],
        Tabelle  absolute Zeit seit 1900          absolute Zeit seit 1900
    AbsoluteTime[Data[[i]][[4]]]}, {i, 2, Length[Data]}];
    absolute Zeit seit 1900                   Länge
Bzvst = Table[{AbsoluteTime[Data[[i]][[9]]] - AbsoluteTime[Data[[2]][[9]]],
        Tabelle  absolute Zeit seit 1900          absolute Zeit seit 1900
    AbsoluteTime[Data[[i]][[5]]]}, {i, 2, Length[Data]}];
    absolute Zeit seit 1900                   Länge
```

```
ListPlot[{Bvst, Bxvst, Byvst, Bzvst}, Joined → True]
listenbezogene Graphik                 verknüpft? wahr
```

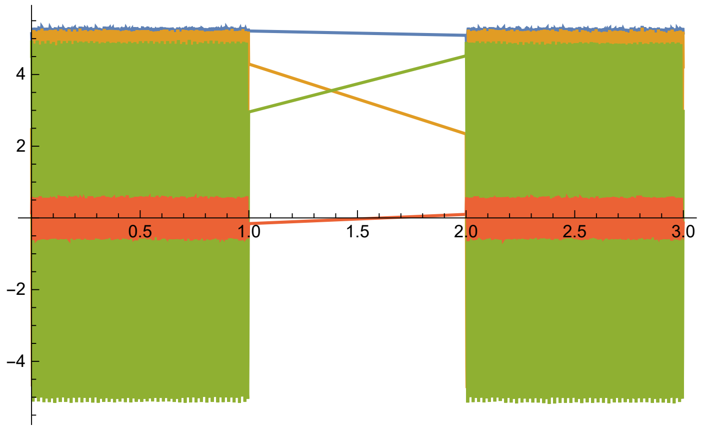


```
Start = 100;
Stop = 1000;
```

```
ListPlot[{Table[{Bvst[[i]][[1]], Bvst[[i]][[2]]}, {i, Start, Stop}],
 listenbezo⋯  Tabelle
  Table[{Bxvst[[i]][[1]], Bxvst[[i]][[2]]}, {i, Start, Stop}],
  Tabelle
  Table[{Byvst[[i]][[1]], Byvst[[i]][[2]]}, {i, Start, Stop}],
  Tabelle
  Table[{Bzvst[[i]][[1]], Bzvst[[i]][[2]]}, {i, Start, Stop}]}, Joined → True]
  Tabelle                                                       verknüpft? wahr
```

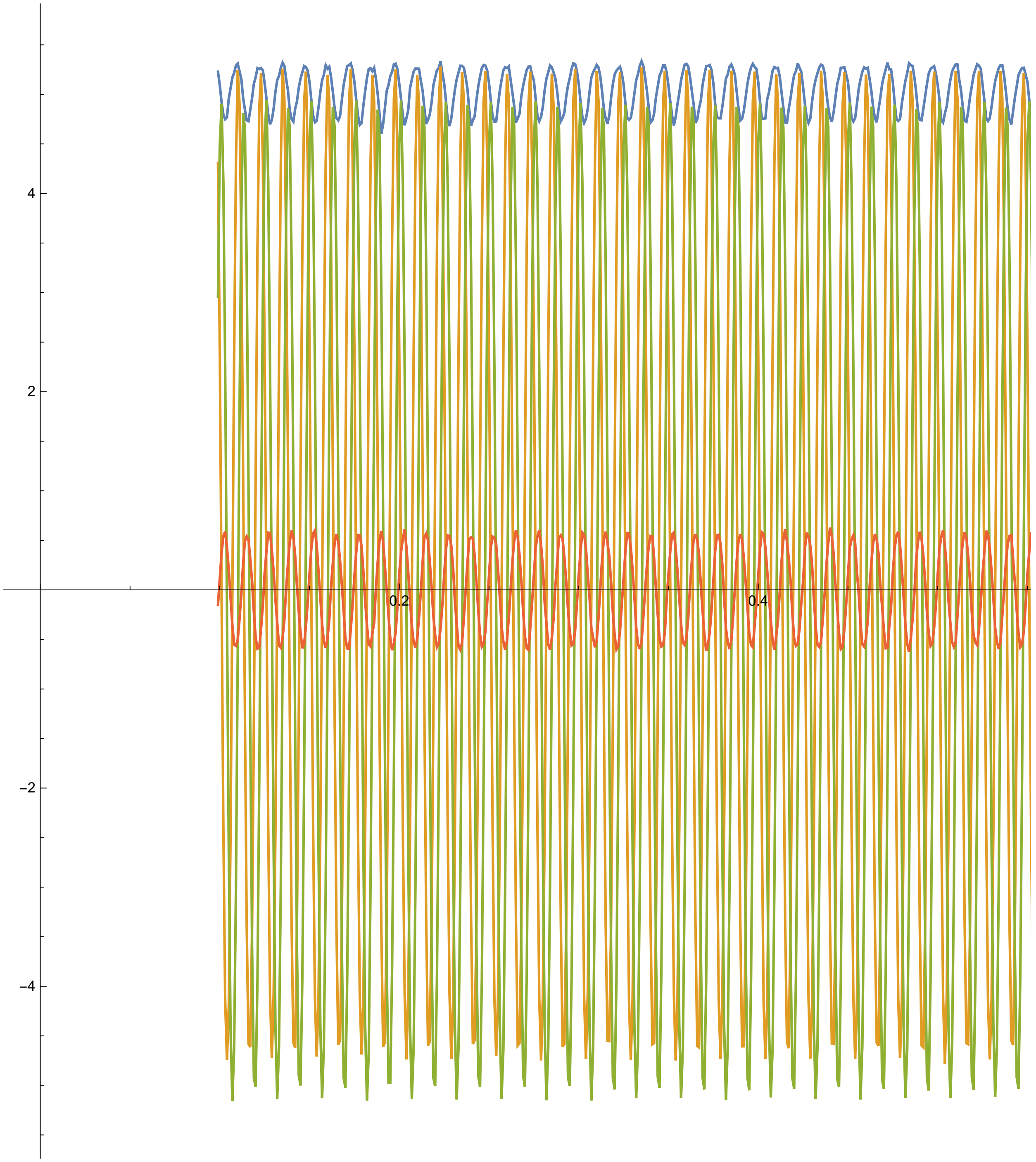

```
ListPlot[{Table[{Bvst[[i]][[1]], Bvst[[i]][[2]]}, {i, Start, Start + 50}],
  Table[{Bxvst[[i]][[1]], Bxvst[[i]][[2]]}, {i, Start, Start + 50}],
  Table[{Byvst[[i]][[1]], Byvst[[i]][[2]]}, {i, Start, Start + 50}],
  Table[{Bzvst[[i]][[1]], Bzvst[[i]][[2]]}, {i, Start, Start + 50}]}, Joined → True]
```

listenbezo··· Tabelle Tabelle Tabelle Tabelle verknüpft? wahr

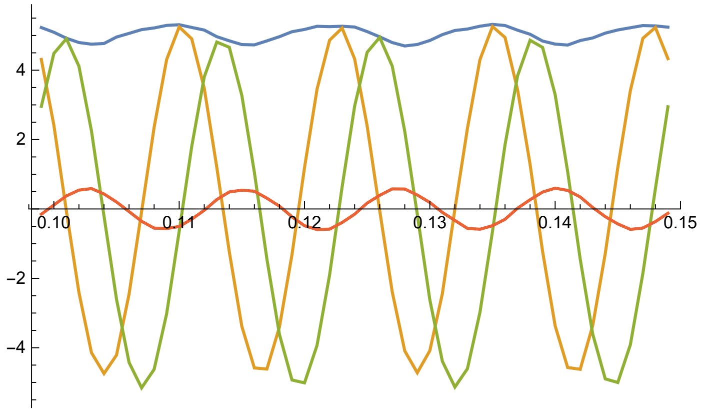


```
TableForm[{Mean[Table[Bvst[[i]][[2]], {i, Start, Stop}]],
  Mean[Table[Bxvst[[i]][[2]], {i, Start, Stop}]],
  Mean[Table[Byvst[[i]][[2]], {i, Start, Stop}]],
  Mean[Table[Bzvst[[i]][[2]], {i, Start, Stop}]]}, TableDirections → Row]
```

Tabellendars··· arit··· Tabelle arit··· Tabelle arit··· Tabelle arit··· Tabelle Richtung der Tabellen··· Zeile

5.0407 0.273468 -0.0983204 -0.00683879

```
TableForm[Table[{(Bvst[[i]][[1]] - Bvst[[Start]][[1]]) * 1000, Bvst[[i]][[2]],
   Bxvst[[i]][[2]], Byvst[[i]][[2]], Bzvst[[i]][[2]]}, {i, Start, Start + 50}]]
```

Tabellendar·· Tabelle

0.	5.2284	4.30917	2.95709	-0.151307
1.0004	5.0932	2.41598	4.48228	0.113541
2.00033	4.92053	-0.062957	4.90577	0.375609
3.00026	4.79703	-2.40543	4.11472	0.54271
4.00019	4.7495	-4.14185	2.24903	0.587137
5.00011	4.76861	-4.74456	-0.20352	0.432882
6.00004	4.95539	-4.21207	-2.60271	0.200724
7.00045	5.06071	-2.45467	-4.42488	-0.07626
8.00037	5.16901	-0.07853	-5.15628	-0.353971
9.0003	5.21731	2.35292	-4.62367	-0.552941
10.0002	5.29092	4.308	-3.0193	-0.564605
11.0002	5.31025	5.24684	-0.642946	-0.505995
12.0001	5.2319	4.90409	1.79877	-0.295148
13.	5.15651	3.47927	3.80562	-0.038777
14.0004	4.96595	1.20214	4.81067	0.270096
15.0003	4.84599	-1.23654	4.65977	0.490972
16.0003	4.73818	-3.3855	3.27065	0.539928
17.0002	4.72944	-4.58224	1.05361	0.510414
18.0001	4.84415	-4.61335	-1.44465	0.30956
19.0001	4.9656	-3.40206	-3.61616	0.080881
20.0005	5.10803	-1.31955	-4.92949	-0.225692
21.0004	5.17399	1.19003	-5.01197	-0.483868
22.0003	5.26483	3.45174	-3.93084	-0.593699
23.0002	5.25476	4.86254	-1.90534	-0.581319
24.0002	5.26627	5.2135	0.632415	-0.391161
25.0001	5.24387	4.32516	2.96111	-0.15141
26.	5.11237	2.38951	4.5164	0.169345
27.0004	4.96028	-0.046833	4.94454	0.392037
28.0004	4.79642	-2.39007	4.1182	0.577611
29.0003	4.69801	-4.09096	2.23728	0.57443
30.0002	4.74061	-4.72093	-0.156023	0.402358
31.0001	4.85535	-4.08519	-2.61737	0.18719
32.0001	5.0245	-2.44294	-4.38952	-0.098845
33.0005	5.14418	-0.006311	-5.13356	-0.330364
34.0004	5.18587	2.32082	-4.60359	-0.560305
35.0003	5.26723	4.30376	-2.98	-0.583965
36.0003	5.32075	5.25908	-0.647282	-0.483184
37.0002	5.28719	4.944	1.84987	-0.29878
38.0001	5.15289	3.44085	3.83567	0.021088
39.	5.03607	1.28604	4.86183	0.266011
40.0004	4.83976	-1.22858	4.65584	0.486829
41.0004	4.75156	-3.36893	3.29677	0.599166
42.0003	4.72446	-4.57002	1.07226	0.534576
43.0002	4.85402	-4.62098	-1.44521	0.345636
44.0001	4.92902	-3.35847	-3.60751	0.041557
45.0001	5.06776	-1.28354	-4.89751	-0.221574
46.	5.1596	1.19055	-5.00177	-0.431709
47.0004	5.22291	3.40776	-3.91395	-0.588987
48.0003	5.28597	4.91782	-1.85877	-0.549172
49.0003	5.27609	5.23083	0.585029	-0.365059
50.0002	5.23995	4.33246	2.94471	-0.124731

(-5,0,0) Blue offset 17A White Offset 49A RMF on

```
Data = Import[NotebookDirectory[] <> "x-5_y0_z0_B17_W49_RMFon.txt", "Table"];
       importi··· Notebook-Verzeichnis                              Tabelle

Bvst = Table[{AbsoluteTime[Data[[i]][[9]]] - AbsoluteTime[Data[[2]][[9]]],
       Tabelle absolute Zeit seit 1900           absolute Zeit seit 1900
    AbsoluteTime[Data[[i]][[2]]]}, {i, 2, Length[Data]}];
    absolute Zeit seit 1900                   Länge
Bxvst = Table[{AbsoluteTime[Data[[i]][[9]]] - AbsoluteTime[Data[[2]][[9]]],
        Tabelle absolute Zeit seit 1900           absolute Zeit seit 1900
    AbsoluteTime[Data[[i]][[3]]]}, {i, 2, Length[Data]}];
    absolute Zeit seit 1900                   Länge
Byvst = Table[{AbsoluteTime[Data[[i]][[9]]] - AbsoluteTime[Data[[2]][[9]]],
        Tabelle absolute Zeit seit 1900           absolute Zeit seit 1900
    AbsoluteTime[Data[[i]][[4]]]}, {i, 2, Length[Data]}];
    absolute Zeit seit 1900                   Länge
Bzvst = Table[{AbsoluteTime[Data[[i]][[9]]] - AbsoluteTime[Data[[2]][[9]]],
        Tabelle absolute Zeit seit 1900           absolute Zeit seit 1900
    AbsoluteTime[Data[[i]][[5]]]}, {i, 2, Length[Data]}];
    absolute Zeit seit 1900                   Länge
```

```
ListPlot[{Bvst, Bxvst, Byvst, Bzvst}, Joined → True]
listenbezogene Graphik                 verknüpft? wahr
```

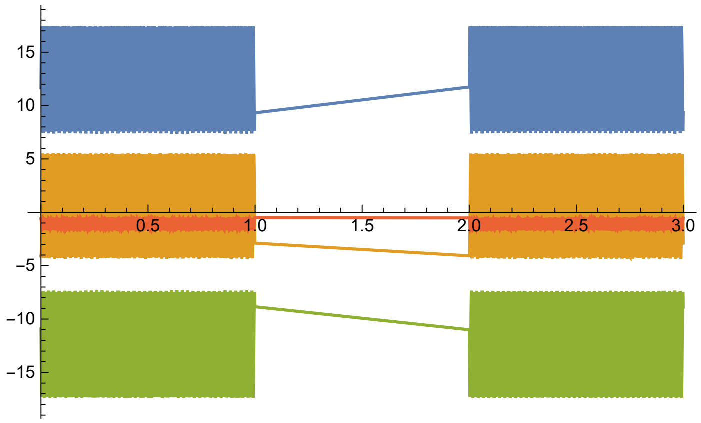


```
Start = 100;
Stop = 1000;
```

```
ListPlot[{Table[{Bvst[[i]][[1]], Bvst[[i]][[2]]}, {i, Start, Stop}],
  Table[{Bxvst[[i]][[1]], Bxvst[[i]][[2]]}, {i, Start, Stop}],
  Table[{Byvst[[i]][[1]], Byvst[[i]][[2]]}, {i, Start, Stop}],
  Table[{Bzvst[[i]][[1]], Bzvst[[i]][[2]]}, {i, Start, Stop}]}, Joined → True]
```

listenbezo··· Tabelle Tabelle Tabelle Tabelle verknüpft? wahr

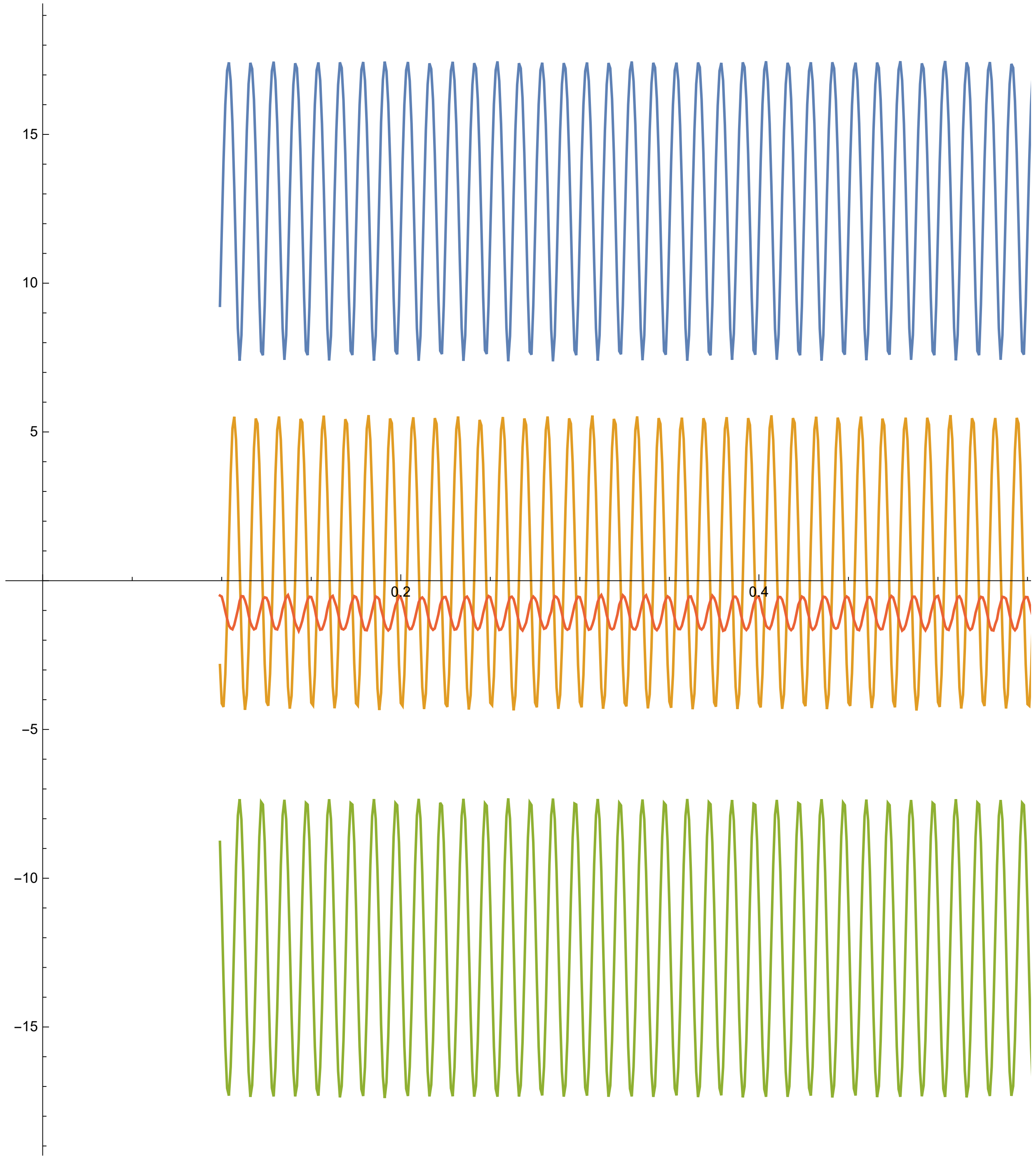

```
ListPlot[{Table[{Bvst[[i]][[1]], Bvst[[i]][[2]]}, {i, Start, Start + 50}],
  Table[{Bxvst[[i]][[1]], Bxvst[[i]][[2]]}, {i, Start, Start + 50}],
  Table[{Byvst[[i]][[1]], Byvst[[i]][[2]]}, {i, Start, Start + 50}],
  Table[{Bzvst[[i]][[1]], Bzvst[[i]][[2]]}, {i, Start, Start + 50}]}, Joined → True]
```

listenbezo··· Tabelle Tabelle Tabelle Tabelle verknüpft? wahr

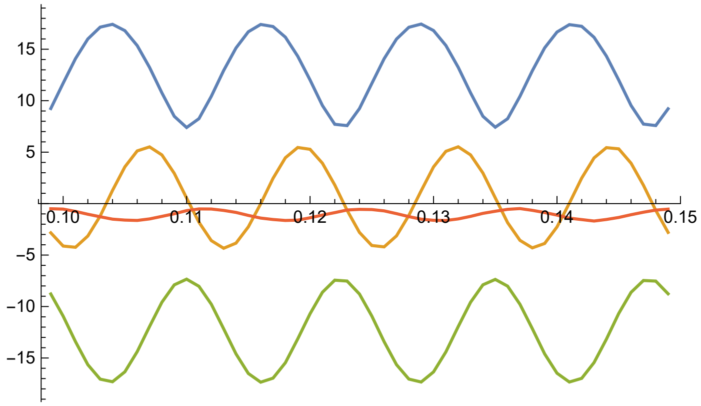


```
TableForm[{Mean[Table[Bvst[[i]][[2]], {i, Start, Stop}]],
  Mean[Table[Bxvst[[i]][[2]], {i, Start, Stop}]],
  Mean[Table[Byvst[[i]][[2]], {i, Start, Stop}]],
  Mean[Table[Bzvst[[i]][[2]], {i, Start, Stop}]]}, TableDirections → Row]
```

Tabellendars··· arit··· Tabelle arit··· Tabelle arit··· Tabelle arit··· Tabelle Richtung der Tabellen··· Zeile

12.9188	0.598607	−12.3575	−1.08675

```
TableForm[Table[{(Bvst[[i]][[1]] - Bvst[[Start]][[1]]) * 1000, Bvst[[i]][[2]],
 Tabellendar·· Tabelle
   Bxvst[[i]][[2]], Byvst[[i]][[2]], Bzvst[[i]][[2]]}, {i, Start, Start + 50}]]
```

0.	9.23977	-2.83687	-8.77933	-0.49887
0.999928	11.6968	-4.12475	-10.9324	-0.534468
1.99986	14.087	-4.24312	-13.4114	-0.758133
2.99978	15.997	-3.14724	-15.6503	-1.03366
3.99971	17.1463	-1.21098	-17.0557	-1.27712
4.99964	17.4208	1.28506	-17.3076	-1.50974
6.00004	16.8047	3.5701	-16.3428	-1.60158
6.99997	15.3532	5.1205	-14.3814	-1.63563
7.9999	13.2317	5.51115	-11.937	-1.48731
8.99982	10.756	4.72985	-9.58031	-1.24024
9.99975	8.48179	2.94238	-7.89385	-0.985017
10.9997	7.39328	0.539711	-7.34187	-0.682768
11.9996	8.25249	-1.8099	-8.03489	-0.518096
13.	10.4251	-3.59402	-9.7718	-0.526221
13.9999	12.9307	-4.33962	-12.1628	-0.66206
14.9999	15.1198	-3.84169	-14.5986	-0.855413
15.9998	16.697	-2.25835	-16.5037	-1.14687
16.9997	17.4064	-0.015879	-17.3496	-1.40495
17.9996	17.2042	2.45535	-16.9579	-1.54447
19.0001	16.1733	4.45473	-15.4612	-1.63725
20.	14.3224	5.45238	-13.1464	-1.60419
20.9999	12.01	5.27779	-10.6998	-1.378
21.9998	9.53218	3.92696	-8.61395	-1.11418
22.9998	7.70601	1.81191	-7.43863	-0.875394
23.9997	7.57773	-0.651503	-7.52406	-0.621257
24.9996	9.23406	-2.79748	-8.78238	-0.558333
26.	11.6688	-4.07021	-10.9206	-0.577859
27.	14.0805	-4.20258	-13.4201	-0.706675
27.9999	15.988	-3.13563	-15.6472	-0.973721
28.9998	17.133	-1.14682	-17.0479	-1.26166
29.9997	17.4454	1.24905	-17.3346	-1.51378
30.9997	16.8161	3.5659	-16.3535	-1.62171
31.9996	15.3656	5.08049	-14.4084	-1.63963
33.	13.2422	5.51916	-11.945	-1.48752
33.9999	10.7614	4.73386	-9.5843	-1.24034
34.9998	8.48407	2.97078	-7.89041	-0.94625
35.9998	7.42228	0.558126	-7.36395	-0.742305
36.9997	8.24763	-1.84931	-8.01834	-0.556443
37.9996	10.4227	-3.56956	-9.78045	-0.482597
39.	12.9019	-4.29955	-12.1469	-0.654228
40.	15.1324	-3.87381	-14.6025	-0.867143
40.9999	16.6697	-2.26605	-16.4772	-1.11538
41.9998	17.391	0.035019	-17.3334	-1.41323
42.9997	17.2279	2.47929	-16.9777	-1.55302
43.9997	16.1593	4.43814	-15.4454	-1.69285
44.9996	14.3363	5.43706	-13.1754	-1.54146
46.	12.0018	5.32109	-10.6732	-1.34603
46.9999	9.53067	3.92334	-8.6185	-1.07821
47.9999	7.72676	1.78835	-7.47025	-0.836627
48.9998	7.58259	-0.655581	-7.52794	-0.629276
49.9997	9.21311	-2.79729	-8.76169	-0.538015

(-5,0,0) Blue offset -16A White Offset -49A RMF off

```
Data = Import[NotebookDirectory[] <> "x-5_y0_z0_B-16_W-49_RMFoff.txt", "Table"];
       importi··· Notebook-Verzeichnis                                     Tabelle
Bvst = Table[{AbsoluteTime[Data[[i]][[9]]] - AbsoluteTime[Data[[2]][[9]]],
       Tabelle absolute Zeit seit 1900          absolute Zeit seit 1900
    AbsoluteTime[Data[[i]][[2]]]}, {i, 2, Length[Data]}];
    absolute Zeit seit 1900                  Länge
Bxvst = Table[{AbsoluteTime[Data[[i]][[9]]] - AbsoluteTime[Data[[2]][[9]]],
        Tabelle absolute Zeit seit 1900          absolute Zeit seit 1900
    AbsoluteTime[Data[[i]][[3]]]}, {i, 2, Length[Data]}];
    absolute Zeit seit 1900                  Länge
Byvst = Table[{AbsoluteTime[Data[[i]][[9]]] - AbsoluteTime[Data[[2]][[9]]],
        Tabelle absolute Zeit seit 1900          absolute Zeit seit 1900
    AbsoluteTime[Data[[i]][[4]]]}, {i, 2, Length[Data]}];
    absolute Zeit seit 1900                  Länge
Bzvst = Table[{AbsoluteTime[Data[[i]][[9]]] - AbsoluteTime[Data[[2]][[9]]],
        Tabelle absolute Zeit seit 1900          absolute Zeit seit 1900
    AbsoluteTime[Data[[i]][[5]]]}, {i, 2, Length[Data]}];
    absolute Zeit seit 1900                  Länge
```

```
ListPlot[{Bvst, Bxvst, Byvst, Bzvst}, Joined → True]
listenbezogene Graphik                verknüpft? wahr
```

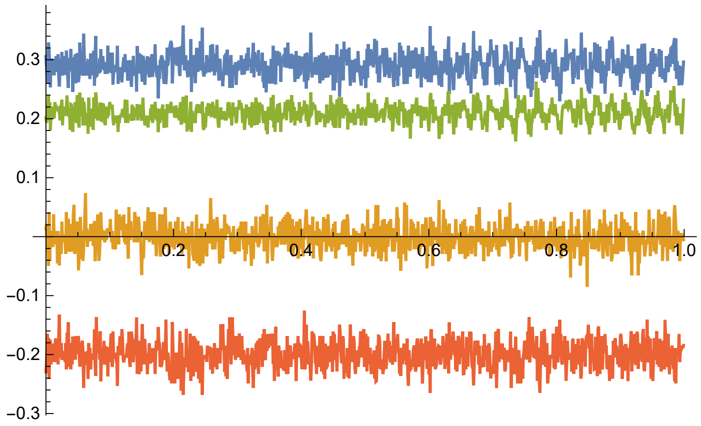


```
Start = 100;
Stop = 1000;
```

```
ListPlot[{Table[{Bvst[[i]][[1]], Bvst[[i]][[2]]}, {i, Start, Stop}],
  Table[{Bxvst[[i]][[1]], Bxvst[[i]][[2]]}, {i, Start, Stop}],
  Table[{Byvst[[i]][[1]], Byvst[[i]][[2]]}, {i, Start, Stop}],
  Table[{Bzvst[[i]][[1]], Bzvst[[i]][[2]]}, {i, Start, Stop}]}, Joined → True]
```

listenbezo··· Tabelle Tabelle Tabelle Tabelle verknüpft? wahr

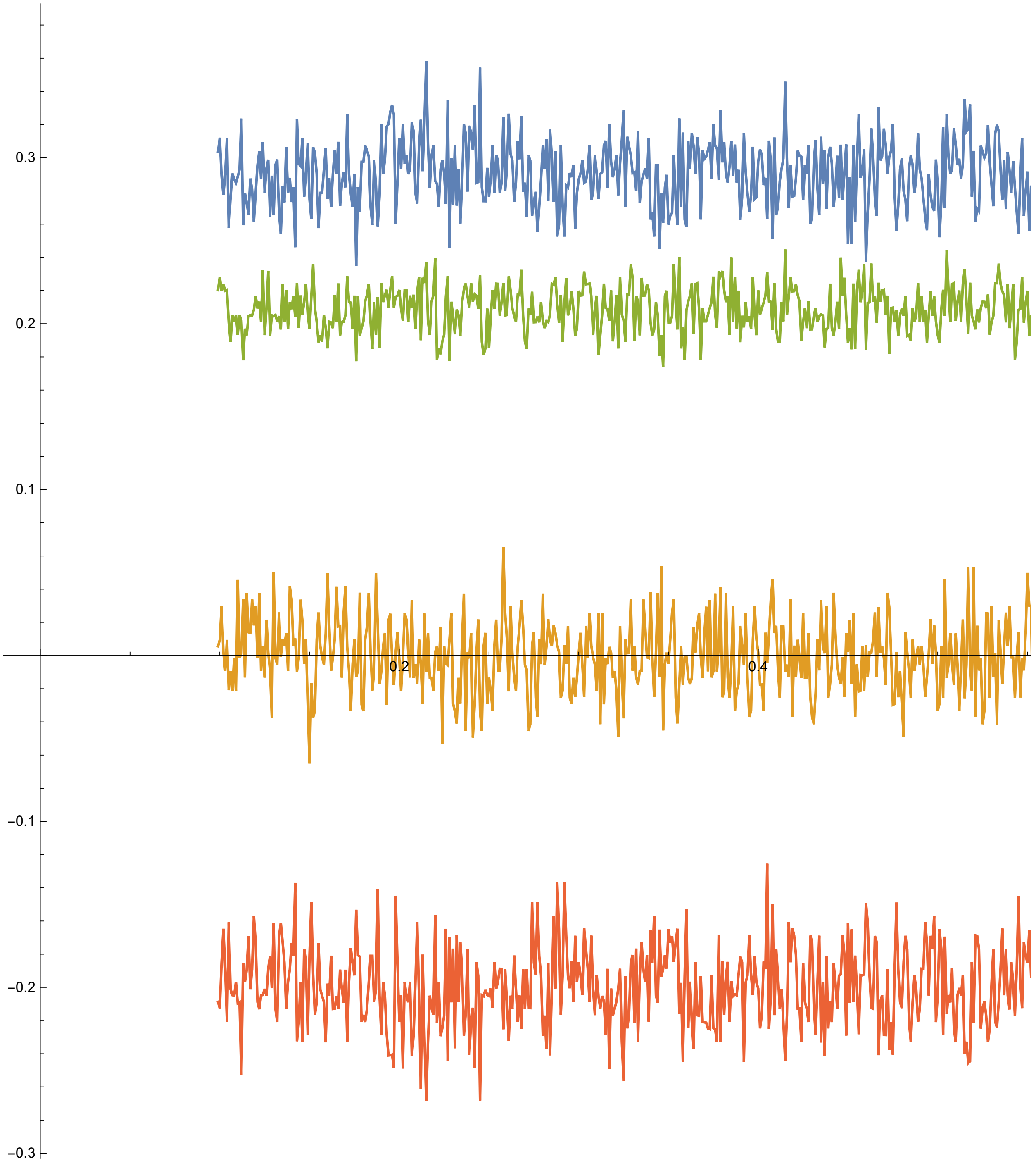

```
ListPlot[{Table[{Bvst[[i]][[1]], Bvst[[i]][[2]]}, {i, Start, Start + 50}],
  Table[{Bxvst[[i]][[1]], Bxvst[[i]][[2]]}, {i, Start, Start + 50}],
  Table[{Byvst[[i]][[1]], Byvst[[i]][[2]]}, {i, Start, Start + 50}],
  Table[{Bzvst[[i]][[1]], Bzvst[[i]][[2]]}, {i, Start, Start + 50}]}, Joined → True]
```

listenbezo··· Tabelle Tabelle Tabelle Tabelle verknüpft? wahr

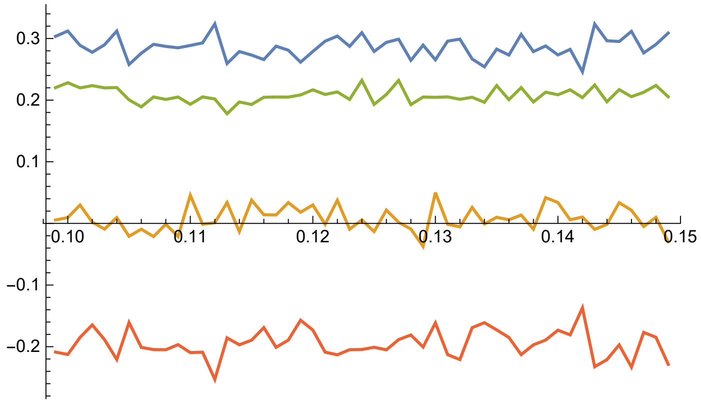


```
TableForm[{Mean[Table[Bvst[[i]][[2]], {i, Start, Stop}]],
  Mean[Table[Bxvst[[i]][[2]], {i, Start, Stop}]],
  Mean[Table[Byvst[[i]][[2]], {i, Start, Stop}]],
  Mean[Table[Bzvst[[i]][[2]], {i, Start, Stop}]]}, TableDirections → Row]
```

Tabellendars··· arit··· Tabelle arit··· Tabelle arit··· Tabelle arit··· Tabelle Richtung der Tabellen··· Zeile

0.290548 −0.00119047 0.209161 −0.199484

```
TableForm[Table[{(Bvst[[i]][[1]] - Bvst[[Start]][[1]]) * 1000, Bvst[[i]][[2]],
Tabellendar·· Tabelle
    Bxvst[[i]][[2]], Byvst[[i]][[2]], Bzvst[[i]][[2]]}, {i, Start, Start + 50}]]
```

0.	0.303475	0.005591	0.220221	-0.208731
0.999928	0.31213	0.009541	0.228285	-0.212649
1.99986	0.288889	0.029837	0.219883	-0.184982
2.99978	0.277635	0.00204	0.223544	-0.164636
4.00019	0.289831	-0.009202	0.219897	-0.18858
5.00011	0.312104	0.009467	0.220409	-0.22077
6.00004	0.2578	-0.020894	0.200458	-0.160752
6.99997	0.276154	-0.009294	0.189084	-0.201052
7.9999	0.290567	-0.021351	0.205129	-0.204683
8.99982	0.287174	-0.001348	0.201153	-0.20495
9.99975	0.284895	-0.021269	0.205007	-0.196684
11.0002	0.288701	0.045616	0.193271	-0.209555
12.0001	0.292829	-0.001394	0.205214	-0.208888
13.	0.32366	0.001157	0.20189	-0.252973
13.9999	0.259355	0.03388	0.177892	-0.185665
14.9999	0.278849	-0.013261	0.197018	-0.196889
15.9998	0.273064	0.037823	0.192956	-0.189476
16.9997	0.265789	0.014018	0.204621	-0.169049
18.0001	0.287534	0.013688	0.20511	-0.201044
19.0001	0.281005	0.033811	0.20495	-0.189252
20.	0.261603	0.018137	0.208442	-0.157031
20.9999	0.278919	0.029964	0.2167	-0.17303
21.9998	0.295603	-0.001398	0.209214	-0.208827
22.9998	0.303901	0.037555	0.21332	-0.213167
24.0002	0.287258	-0.009348	0.201144	-0.204868
25.0001	0.309466	0.00562	0.232158	-0.204548
26.	0.278993	-0.013298	0.193079	-0.200949
27.	0.293728	0.021642	0.20918	-0.205064
27.9999	0.298866	0.001785	0.231909	-0.18851
28.9998	0.264585	-0.009092	0.192779	-0.180994
29.9997	0.289214	-0.037309	0.205049	-0.200519
31.0001	0.265338	0.050099	0.204542	-0.161421
32.0001	0.295738	-0.001435	0.205275	-0.212887
33.	0.29898	-0.005513	0.201393	-0.220906
33.9999	0.266781	0.026017	0.204635	-0.169173
34.9998	0.254033	-0.00089	0.196482	-0.161018
35.9998	0.282774	0.009957	0.223675	-0.172717
37.0002	0.273152	0.005858	0.200856	-0.185025
38.0001	0.306596	0.013549	0.220291	-0.212813
39.	0.27872	-0.009261	0.197022	-0.19693
40.	0.287924	0.041802	0.212959	-0.189212
40.9999	0.273328	0.033972	0.208706	-0.173193
41.9998	0.28234	0.005882	0.216794	-0.180782
42.9997	0.246065	0.010348	0.204128	-0.137014
44.0001	0.323398	-0.00966	0.224569	-0.232511
45.0001	0.296333	-0.001509	0.197398	-0.221008
46.	0.295114	0.033716	0.217071	-0.197067
46.9999	0.31157	0.021358	0.205608	-0.233121
47.9999	0.276603	-0.005072	0.21272	-0.17673
48.9998	0.290395	0.009834	0.223858	-0.184715
50.0002	0.308799	-0.029598	0.205486	-0.228597

(5,0,0) Blue offset 0A White Offset 0A RMF off

```
Data = Import[NotebookDirectory[] <> "x5_y0_z0_no_offset_RMFoff.txt", "Table"];
        importi··· Notebook–Verzeichnis                                    Tabelle

Bvst = Table[{AbsoluteTime[Data[[i]][[9]]] - AbsoluteTime[Data[[2]][[9]]],
        Tabelle  absolute Zeit seit 1900        absolute Zeit seit 1900
    AbsoluteTime[Data[[i]][[2]]]}, {i, 2, Length[Data]}];
    absolute Zeit seit 1900                   Länge
Bxvst = Table[{AbsoluteTime[Data[[i]][[9]]] - AbsoluteTime[Data[[2]][[9]]],
        Tabelle  absolute Zeit seit 1900        absolute Zeit seit 1900
    AbsoluteTime[Data[[i]][[3]]]}, {i, 2, Length[Data]}];
    absolute Zeit seit 1900                   Länge
Byvst = Table[{AbsoluteTime[Data[[i]][[9]]] - AbsoluteTime[Data[[2]][[9]]],
        Tabelle  absolute Zeit seit 1900        absolute Zeit seit 1900
    AbsoluteTime[Data[[i]][[4]]]}, {i, 2, Length[Data]}];
    absolute Zeit seit 1900                   Länge
Bzvst = Table[{AbsoluteTime[Data[[i]][[9]]] - AbsoluteTime[Data[[2]][[9]]],
        Tabelle  absolute Zeit seit 1900        absolute Zeit seit 1900
    AbsoluteTime[Data[[i]][[5]]]}, {i, 2, Length[Data]}];
    absolute Zeit seit 1900                   Länge
```

```
ListPlot[{Bvst, Bxvst, Byvst, Bzvst}, Joined → True]
listenbezogene Graphik                  verknüpft? wahr
```

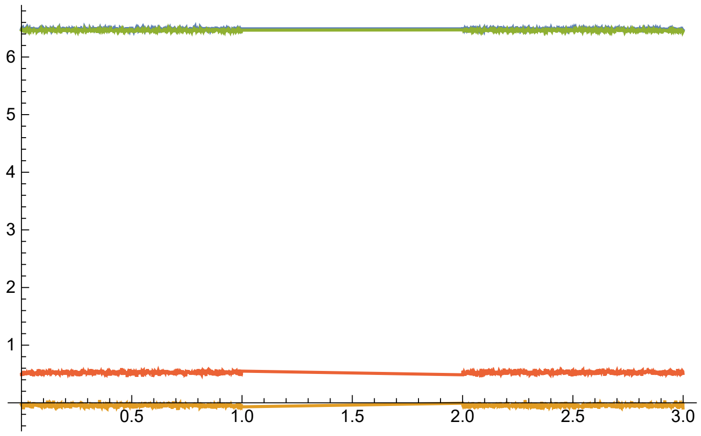


```
Start = 100;
Stop = 1000;
```

```
ListPlot[{Table[{Bvst[[i]][[1]], Bvst[[i]][[2]]}, {i, Start, Stop}],
listenbezo⋯ Tabelle
  Table[{Bxvst[[i]][[1]], Bxvst[[i]][[2]]}, {i, Start, Stop}],
  Tabelle
  Table[{Byvst[[i]][[1]], Byvst[[i]][[2]]}, {i, Start, Stop}],
  Tabelle
  Table[{Bzvst[[i]][[1]], Bzvst[[i]][[2]]}, {i, Start, Stop}]}, Joined → True]
  Tabelle                                                      verknüpft? wahr
```

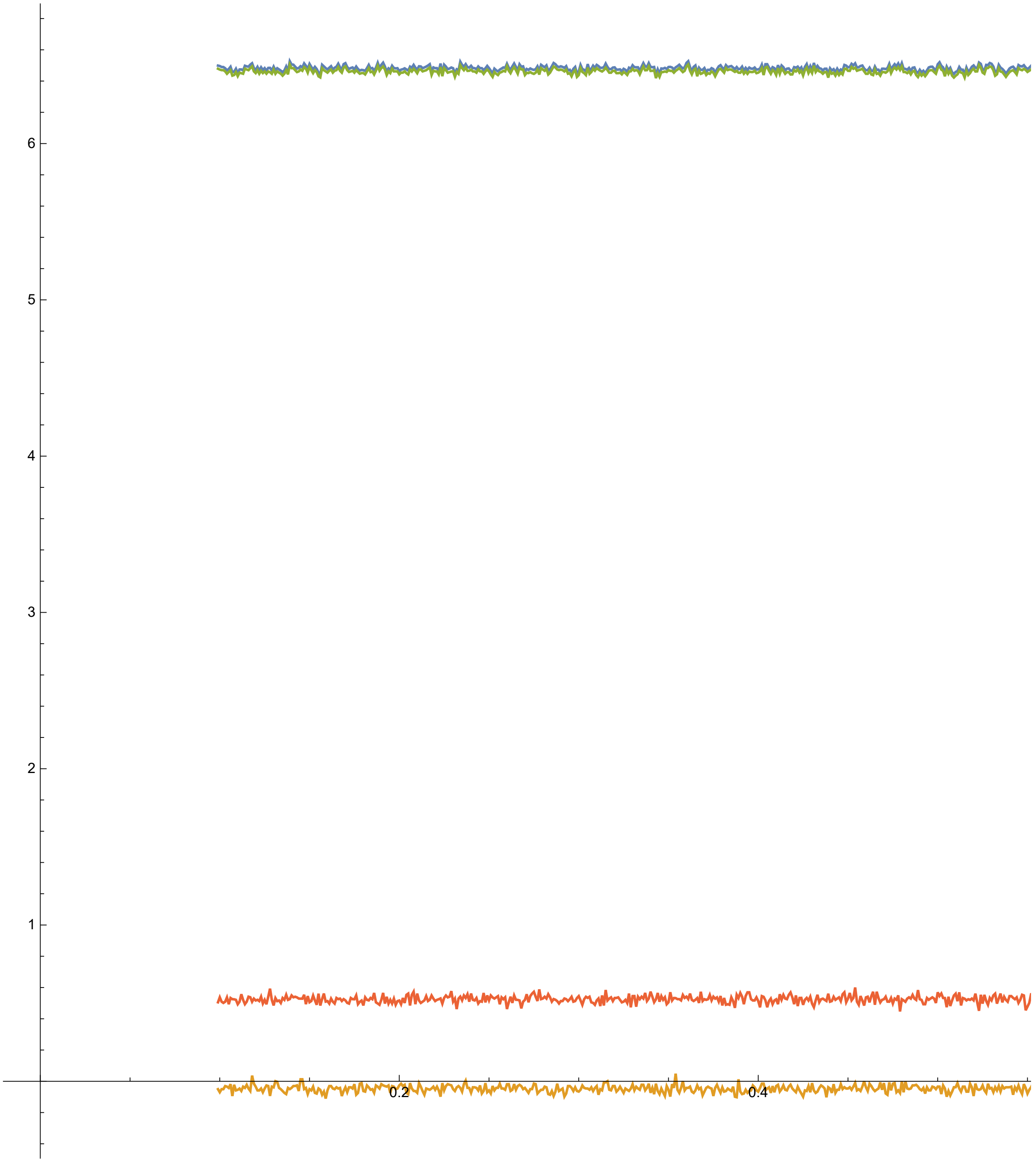

```
ListPlot[{Table[{Bvst[[i]][[1]], Bvst[[i]][[2]]}, {i, Start, Start + 50}],
listenbezo…  Tabelle
  Table[{Bxvst[[i]][[1]], Bxvst[[i]][[2]]}, {i, Start, Start + 50}],
  Tabelle
  Table[{Byvst[[i]][[1]], Byvst[[i]][[2]]}, {i, Start, Start + 50}],
  Tabelle
  Table[{Bzvst[[i]][[1]], Bzvst[[i]][[2]]}, {i, Start, Start + 50}]}, Joined → True]
  Tabelle                                                        verknüpft? wahr
```

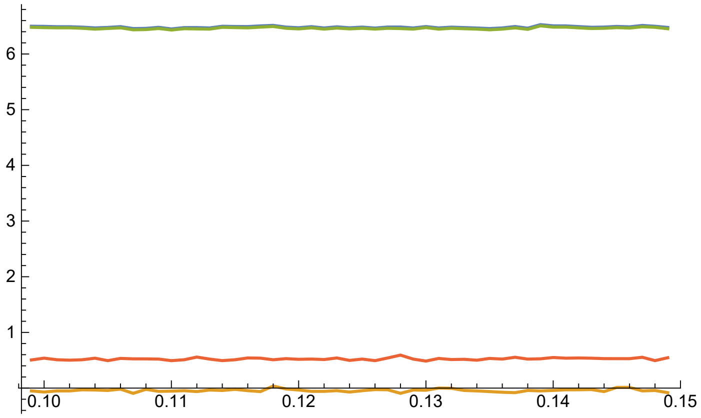


```
TableForm[{Mean[Table[Bvst[[i]][[2]], {i, Start, Stop}]],
Tabellendars…arit…  Tabelle
  Mean[Table[Bxvst[[i]][[2]], {i, Start, Stop}]],
  arit…  Tabelle
  Mean[Table[Byvst[[i]][[2]], {i, Start, Stop}]],
  arit…  Tabelle
  Mean[Table[Bzvst[[i]][[2]], {i, Start, Stop}]]}, TableDirections → Row]
  arit…  Tabelle                                Richtung der Tabellen… Zeile
```

6.48323 -0.0465816 6.46161 0.526004

```
TableForm[Table[{(Bvst[[i]][[1]] - Bvst[[Start]][[1]]) * 1000, Bvst[[i]][[2]],
Tabellendar·· Tabelle
   Bxvst[[i]][[2]], Byvst[[i]][[2]], Bzvst[[i]][[2]]}, {i, Start, Start + 50}]]
```

0.	6.49887	-0.050486	6.47898	0.50552
1.0004	6.49614	-0.07015	6.47347	0.537644
2.00033	6.49014	-0.050435	6.46992	0.509382
3.00026	6.48964	-0.050518	6.47005	0.501383
4.00019	6.48204	-0.030428	6.46195	0.509054
5.00011	6.46892	-0.034124	6.44652	0.536861
6.00004	6.47712	-0.042588	6.45818	0.493119
6.99997	6.49154	-0.01819	6.4696	0.533048
8.00037	6.45673	-0.094232	6.43463	0.525297
9.0003	6.46009	-0.02224	6.43872	0.524617
10.0002	6.479	-0.058299	6.45773	0.521279
11.0002	6.45026	-0.05456	6.43117	0.49283
12.0001	6.47415	-0.046419	6.45393	0.509096
13.	6.4745	-0.06192	6.45018	0.557207
14.0004	6.46786	-0.034289	6.44676	0.520864
15.0003	6.49808	-0.04261	6.47918	0.49344
16.0003	6.49399	-0.022441	6.47396	0.509155
17.0002	6.49221	-0.046106	6.46944	0.541335
18.0001	6.50504	-0.062159	6.48248	0.537699
19.0001	6.51403	0.036535	6.49403	0.508853
20.	6.48329	-0.015223	6.46166	0.528896
21.0004	6.47159	-0.034334	6.45082	0.516926
22.0003	6.49097	-0.058311	6.46973	0.521462
23.0002	6.46751	-0.05837	6.44686	0.513112
24.0002	6.48822	-0.046101	6.46544	0.541274
25.0001	6.47061	-0.070538	6.45109	0.4973
26.	6.4829	-0.046304	6.46175	0.521216
27.0004	6.46606	-0.026578	6.4472	0.492786
28.0004	6.48414	-0.030098	6.46146	0.541048
29.0003	6.48442	-0.093565	6.45661	0.592637
30.0002	6.46786	-0.034289	6.44676	0.520864
31.0001	6.49258	-0.038688	6.4743	0.485324
32.0001	6.46858	0.000833	6.44662	0.532501
33.	6.48223	-0.003388	6.46192	0.512775
34.0004	6.47463	-0.042337	6.45381	0.517054
35.0003	6.46669	-0.050494	6.44705	0.501032
36.0003	6.45688	-0.062151	6.43455	0.532967
37.0002	6.46818	-0.074287	6.44672	0.521275
38.0001	6.49339	-0.08198	6.46922	0.553704
39.	6.46391	-0.042284	6.44275	0.520885
40.0004	6.52709	-0.050307	6.50568	0.525929
41.0004	6.50571	-0.042037	6.48232	0.549491
42.0003	6.50482	-0.030161	6.48252	0.53737
43.0002	6.49212	-0.030106	6.46946	0.541171
44.0001	6.47987	-0.026136	6.45753	0.536947
45.0001	6.48355	-0.06222	6.46161	0.52938
46.	6.49527	0.012763	6.47369	0.528791
47.0004	6.48728	0.012772	6.46569	0.528669
48.0003	6.51302	-0.050002	6.48925	0.55368
49.0003	6.49808	-0.04261	6.47918	0.49344
50.0002	6.47687	-0.082046	6.45334	0.545461
