# Supplementary figures and images for: Spatially selective delivery of living magnetic microrobots through torque-focusing
Source: Nat Commun. 2024 Mar 9;15:2160. doi: 10.1038/s41467-024-46407-4 (PMC10924878; doi:10.1038/s41467-024-46407-4)

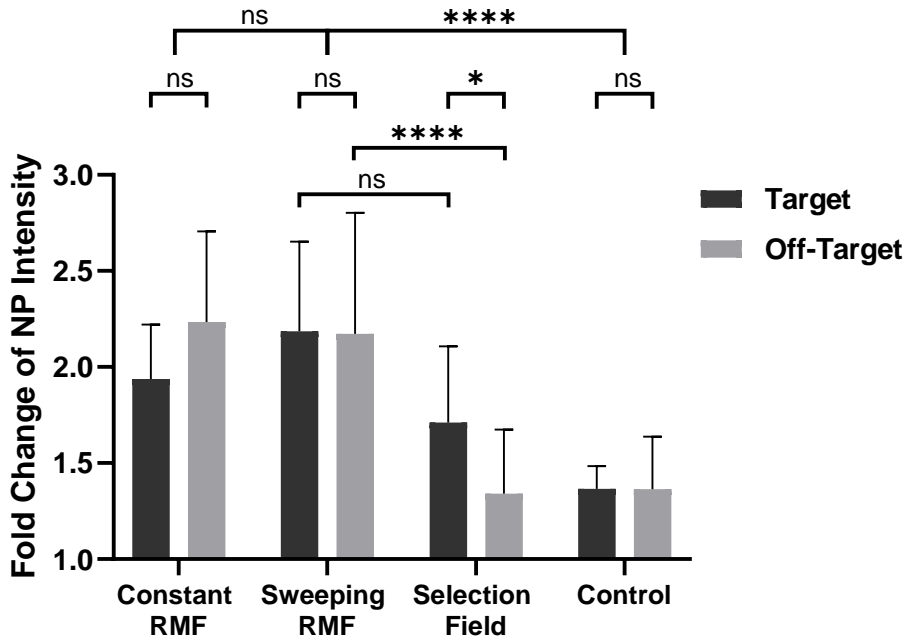

Supplement: Supplementary file 4 — Source Data [file 41467_2024_46407_MOESM4_ESM.zip › Data_Sorted_by_Figures/Fig_2/2G/NP Transport Actuation Groups.pdf]

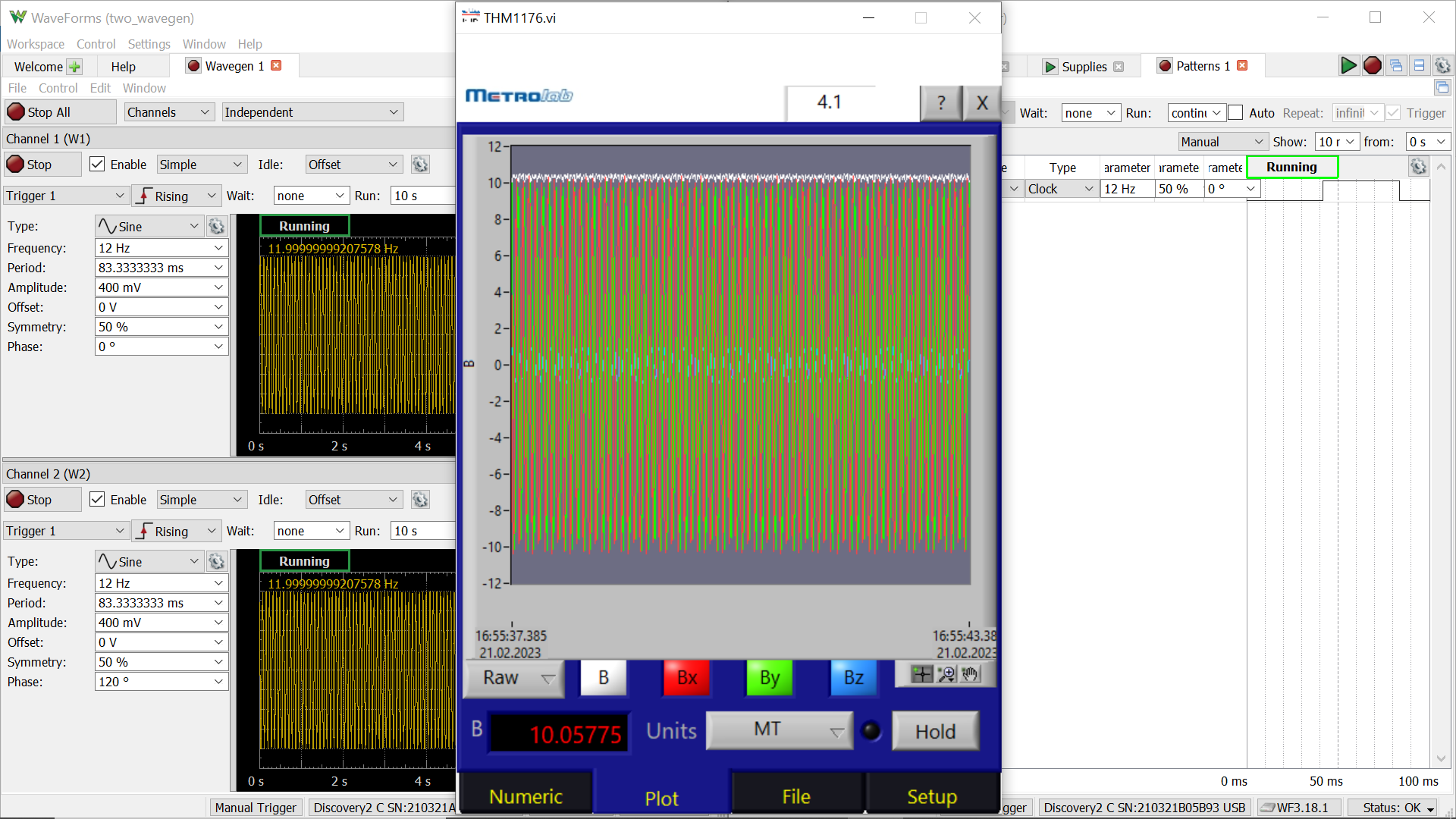

Supplement: Supplementary file 4 — Source Data [file 41467_2024_46407_MOESM4_ESM.zip › Data_Sorted_by_Figures/Fig_4/4J/Selection_Field_Setup_Screenshot.png]

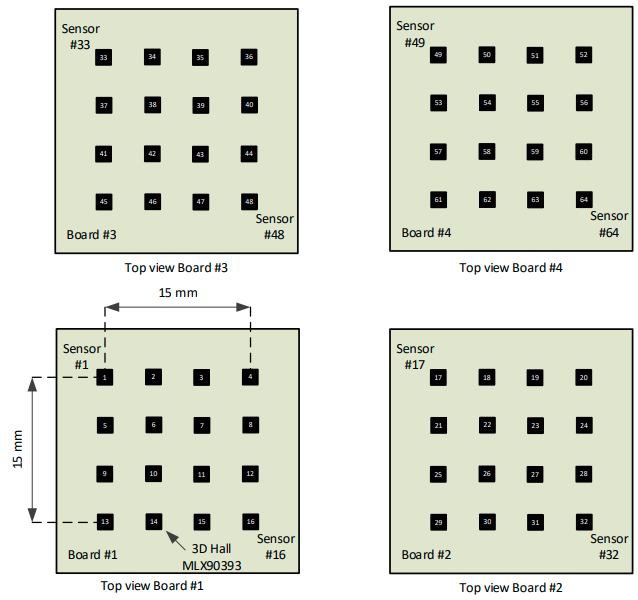

Supplement: Supplementary file 4 — Source Data [file 41467_2024_46407_MOESM4_ESM.zip › Data_Sorted_by_Figures/Fig_4/4E-F/Sensor_Layout.png]

Normalized delta mixing parameter

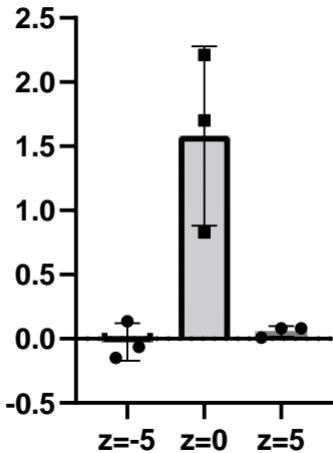

Supplement: Supplementary file 4 — Source Data [file 41467_2024_46407_MOESM4_ESM.zip › Data_Sorted_by_Figures/Fig_4/4J/Mixing/Normalized delta MI z.pdf]

Normalized delta mixing parameter

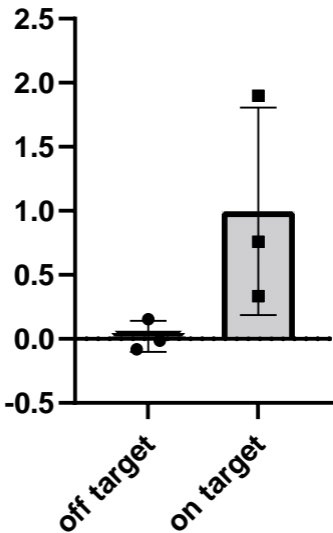

Supplement: Supplementary file 4 — Source Data [file 41467_2024_46407_MOESM4_ESM.zip › Data_Sorted_by_Figures/Fig_4/4H/Mixing/Normalized delta MI negative x.pdf]

Normalized delta mixing parameter

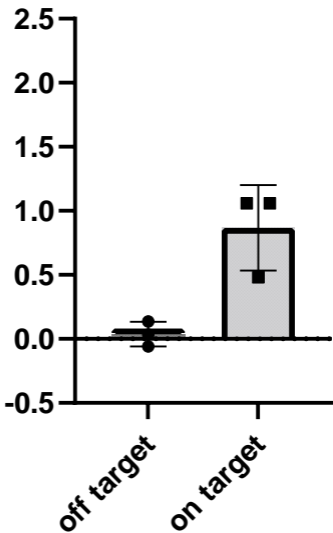

Supplement: Supplementary file 4 — Source Data [file 41467_2024_46407_MOESM4_ESM.zip › Data_Sorted_by_Figures/Fig_4/4H/Mixing/Normalized delta MI positive x.pdf]

Normalized delta mixing parameter

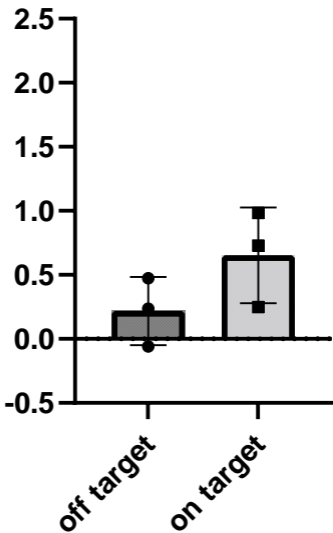

Supplement: Supplementary file 4 — Source Data [file 41467_2024_46407_MOESM4_ESM.zip › Data_Sorted_by_Figures/Fig_4/4I/Mixing/Normalized delta MI negative y.pdf]

Normalized delta mixing parameter

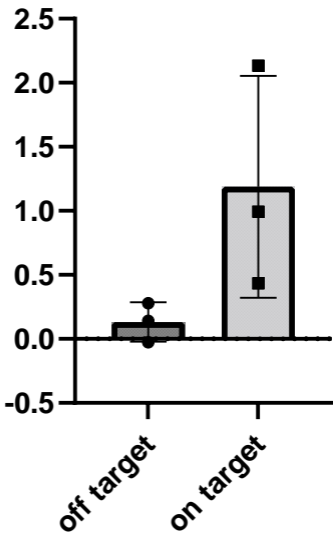

Supplement: Supplementary file 4 — Source Data [file 41467_2024_46407_MOESM4_ESM.zip › Data_Sorted_by_Figures/Fig_4/4I/Mixing/Normalized delta MI positive y.pdf]

# $(0,0,-7.5)$ RMF on

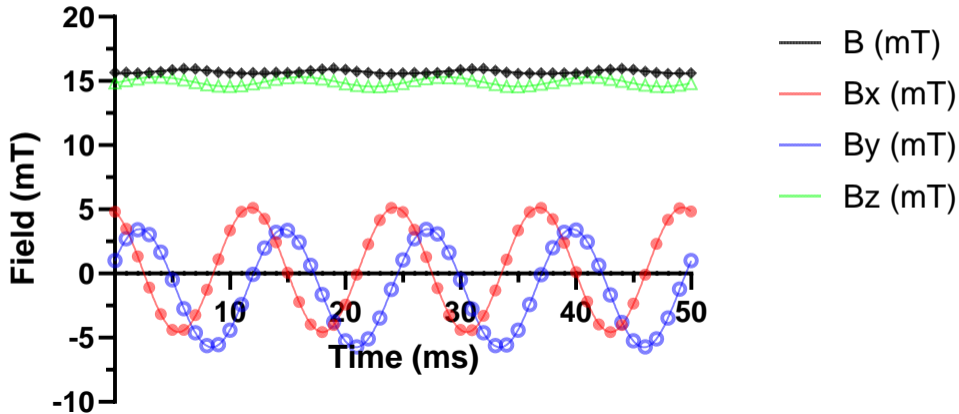

Supplement: Supplementary file 4 — Source Data [file 41467_2024_46407_MOESM4_ESM.zip › Data_Sorted_by_Figures/Fig_4/4J/Field Characterization/z axis measurements/(0,0,-7.5) RMF on.pdf]

# $(0,0,-2.5)$ RMF off

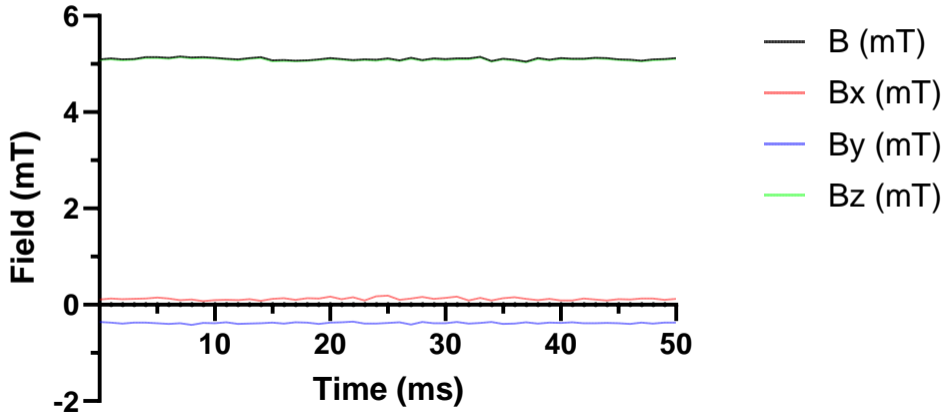

Supplement: Supplementary file 4 — Source Data [file 41467_2024_46407_MOESM4_ESM.zip › Data_Sorted_by_Figures/Fig_4/4J/Field Characterization/z axis measurements/(0,0,-2.5) RMF off.pdf]

# **(0,0,7.5) RMF off**

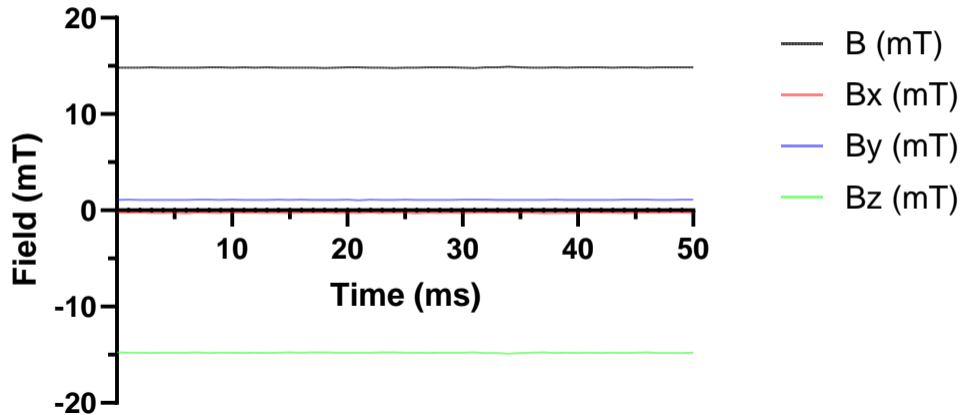

Supplement: Supplementary file 4 — Source Data [file 41467_2024_46407_MOESM4_ESM.zip › Data_Sorted_by_Figures/Fig_4/4J/Field Characterization/z axis measurements/(0,0,7.5) RMF off.pdf]

# $(0,0,-5)$ RMF on

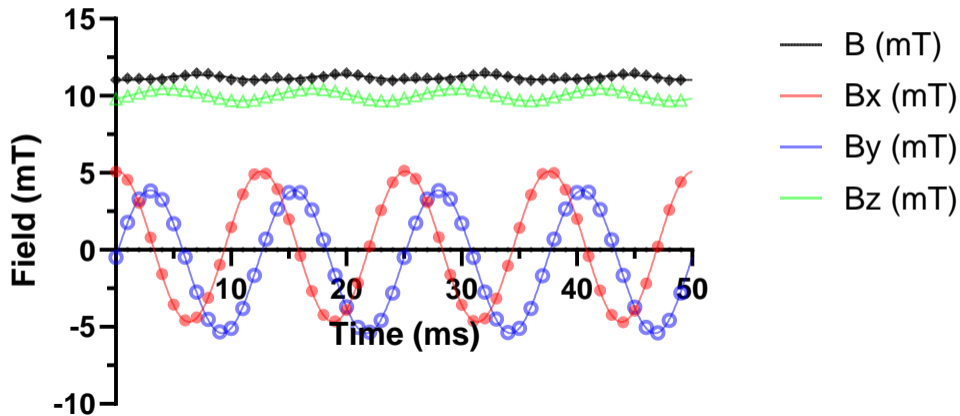

Supplement: Supplementary file 4 — Source Data [file 41467_2024_46407_MOESM4_ESM.zip › Data_Sorted_by_Figures/Fig_4/4J/Field Characterization/z axis measurements/(0,0,-5) RMF on.pdf]

# **(0,0,0) RMF off**

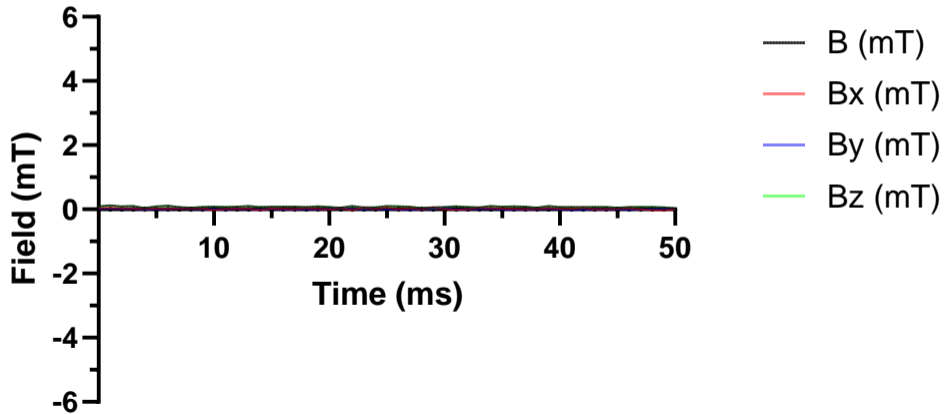

Supplement: Supplementary file 4 — Source Data [file 41467_2024_46407_MOESM4_ESM.zip › Data_Sorted_by_Figures/Fig_4/4J/Field Characterization/z axis measurements/(0,0,0) RMF off.pdf]

# **(0,0,2.5) RMF off**

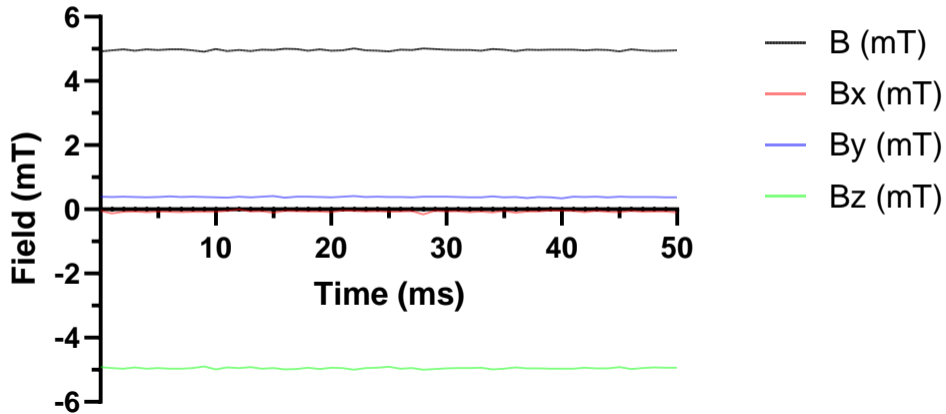

Supplement: Supplementary file 4 — Source Data [file 41467_2024_46407_MOESM4_ESM.zip › Data_Sorted_by_Figures/Fig_4/4J/Field Characterization/z axis measurements/(0,0,2.5) RMF off.pdf]

# $(0,0,-7.5)$ RMF off

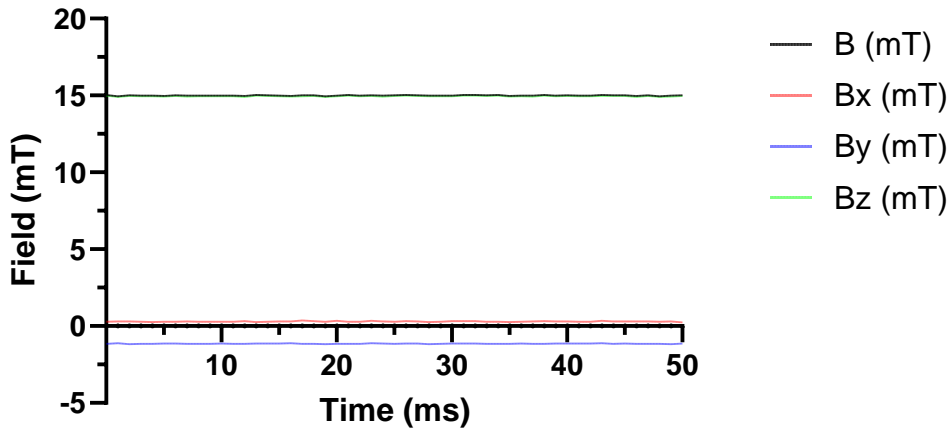

Supplement: Supplementary file 4 — Source Data [file 41467_2024_46407_MOESM4_ESM.zip › Data_Sorted_by_Figures/Fig_4/4J/Field Characterization/z axis measurements/(0,0,-7.5) RMF off.pdf]

# $(0,0,0)$ RMF on

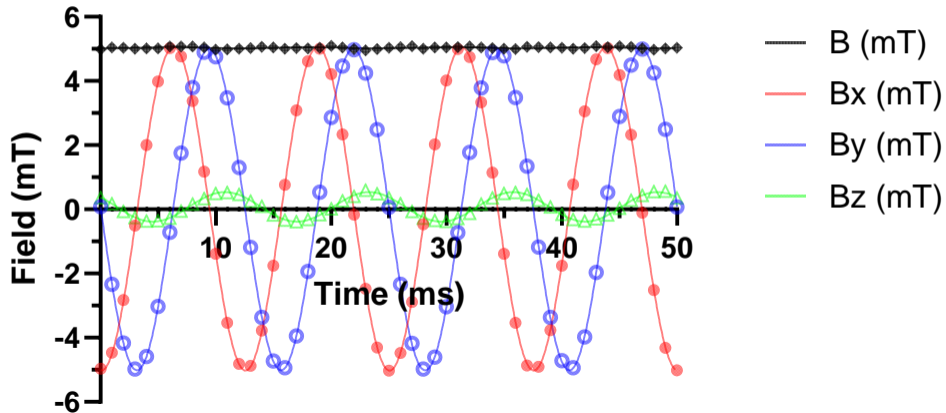

Supplement: Supplementary file 4 — Source Data [file 41467_2024_46407_MOESM4_ESM.zip › Data_Sorted_by_Figures/Fig_4/4J/Field Characterization/z axis measurements/(0,0,0) RMF on.pdf]

# $(0,0,7.5)$ RMF on

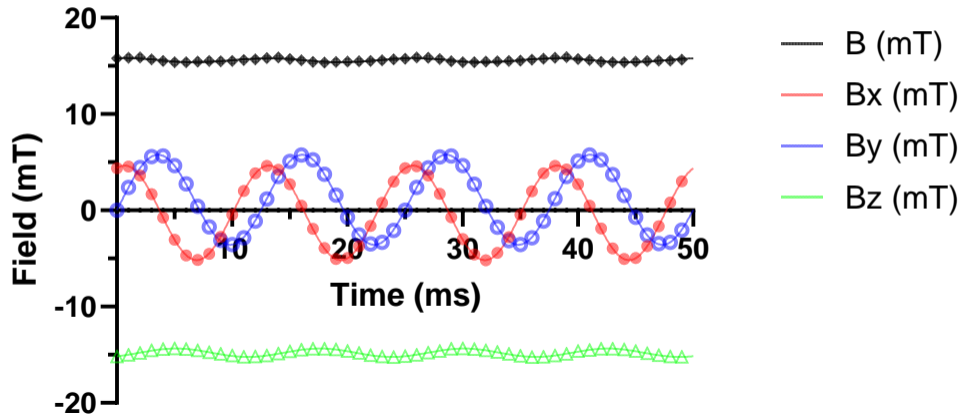

Supplement: Supplementary file 4 — Source Data [file 41467_2024_46407_MOESM4_ESM.zip › Data_Sorted_by_Figures/Fig_4/4J/Field Characterization/z axis measurements/(0,0,7.5) RMF on.pdf]

# $(0,0,5)$ RMF on

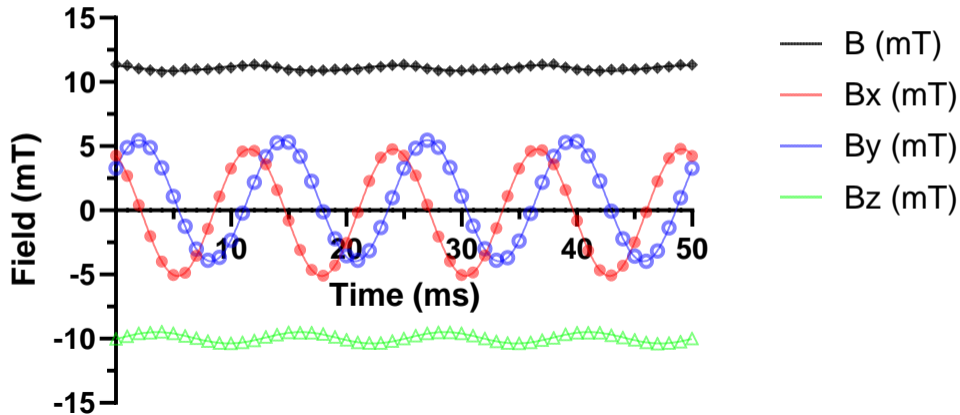

Supplement: Supplementary file 4 — Source Data [file 41467_2024_46407_MOESM4_ESM.zip › Data_Sorted_by_Figures/Fig_4/4J/Field Characterization/z axis measurements/(0,0,5) RMF on.pdf]

# $(0,0,-5)$ RMF off

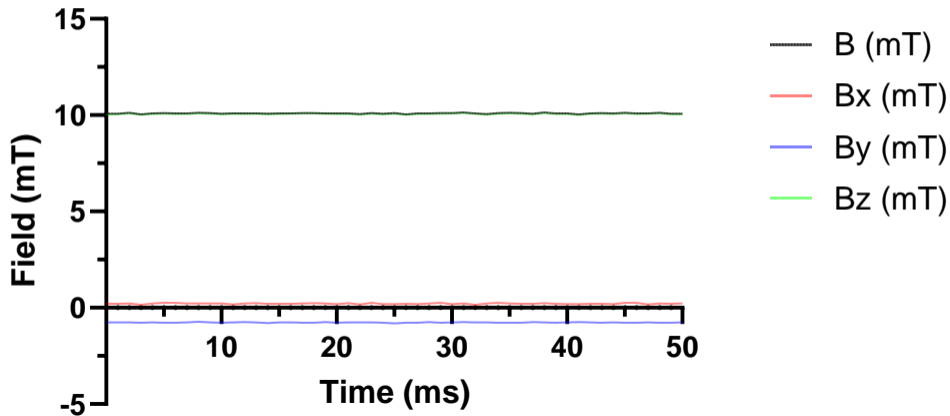

Supplement: Supplementary file 4 — Source Data [file 41467_2024_46407_MOESM4_ESM.zip › Data_Sorted_by_Figures/Fig_4/4J/Field Characterization/z axis measurements/(0,0,-5) RMF off.pdf]

# $(0,0,5)$ RMF off

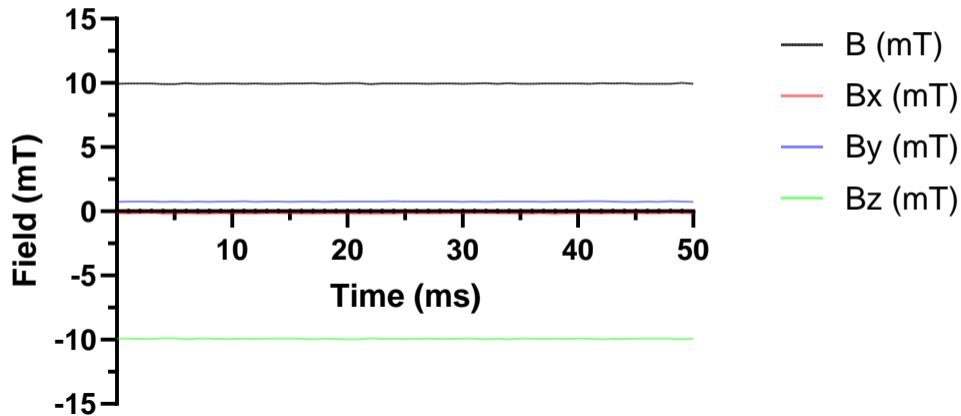

Supplement: Supplementary file 4 — Source Data [file 41467_2024_46407_MOESM4_ESM.zip › Data_Sorted_by_Figures/Fig_4/4J/Field Characterization/z axis measurements/(0,0,5) RMF off.pdf]

# $(0,0,2.5)$ RMF on

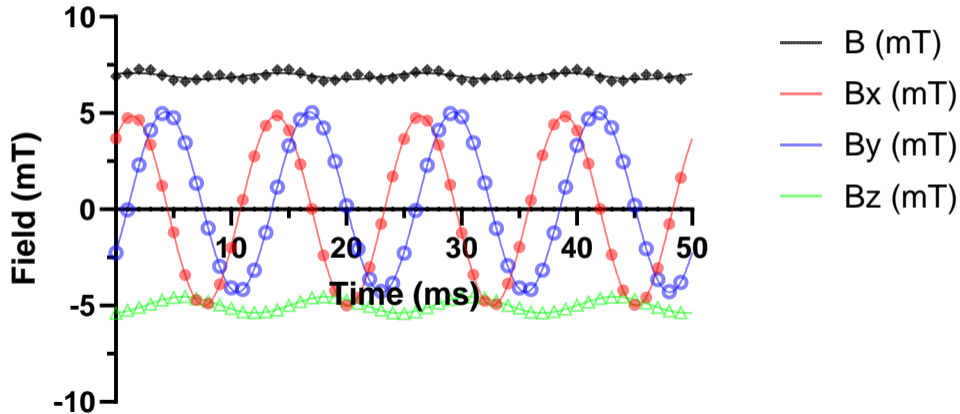

Supplement: Supplementary file 4 — Source Data [file 41467_2024_46407_MOESM4_ESM.zip › Data_Sorted_by_Figures/Fig_4/4J/Field Characterization/z axis measurements/(0,0,2.5) RMF on.pdf]

# $(0,0,-2.5)$ RMF on

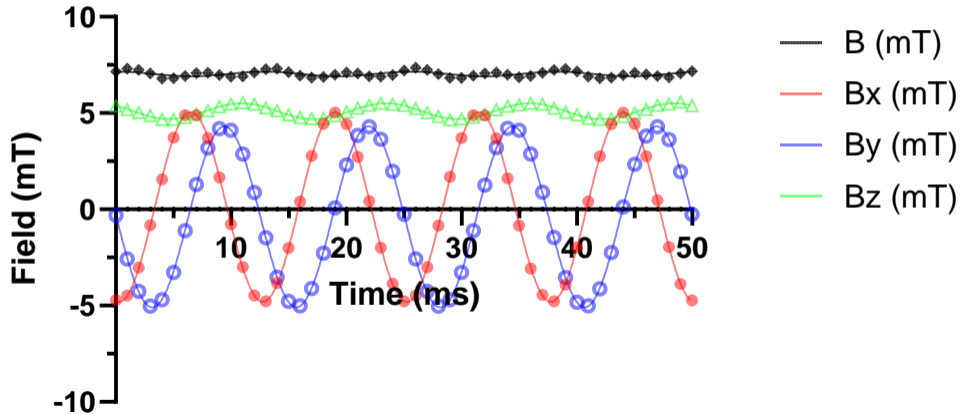

Supplement: Supplementary file 4 — Source Data [file 41467_2024_46407_MOESM4_ESM.zip › Data_Sorted_by_Figures/Fig_4/4J/Field Characterization/z axis measurements/(0,0,-2.5) RMF on.pdf]

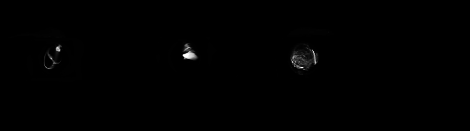

Supplement: Supplementary file 4 — Source Data [file 41467_2024_46407_MOESM4_ESM.zip › Data_Sorted_by_Figures/Fig_4/4H/Mixing/230227 x -5mm/x-5_y0_z0_on_target_postscan_520.tif]

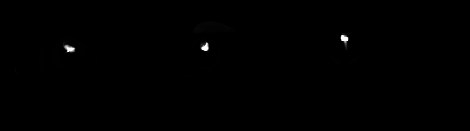

Supplement: Supplementary file 4 — Source Data [file 41467_2024_46407_MOESM4_ESM.zip › Data_Sorted_by_Figures/Fig_4/4H/Mixing/230227 x -5mm/x-5_y0_z0_off-target_postscan_520.tif]

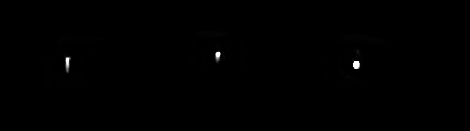

Supplement: Supplementary file 4 — Source Data [file 41467_2024_46407_MOESM4_ESM.zip › Data_Sorted_by_Figures/Fig_4/4H/Mixing/230227 x -5mm/x-5_y0_z0_on_target_prescan_520.tif]

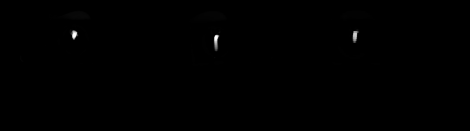

Supplement: Supplementary file 4 — Source Data [file 41467_2024_46407_MOESM4_ESM.zip › Data_Sorted_by_Figures/Fig_4/4H/Mixing/230227 x -5mm/x-5_y0_z0_off_target_prescan_520.tif]

**(-5,0,0) B 17A W 49A RMF off**

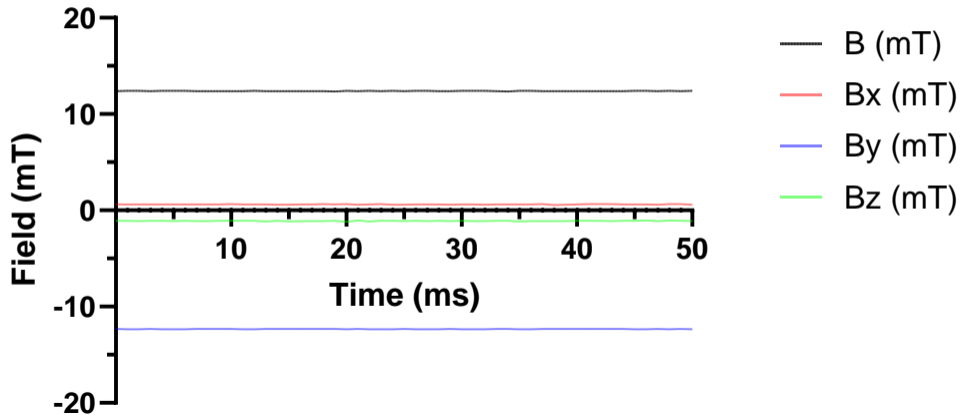

Supplement: Supplementary file 4 — Source Data [file 41467_2024_46407_MOESM4_ESM.zip › Data_Sorted_by_Figures/Fig_4/4H/Field Characterization/x axis measurements/(-5,0,0) B 17A W 49A RMF off.pdf]

# **(5,0,0) B 17A W 49A RMF off**

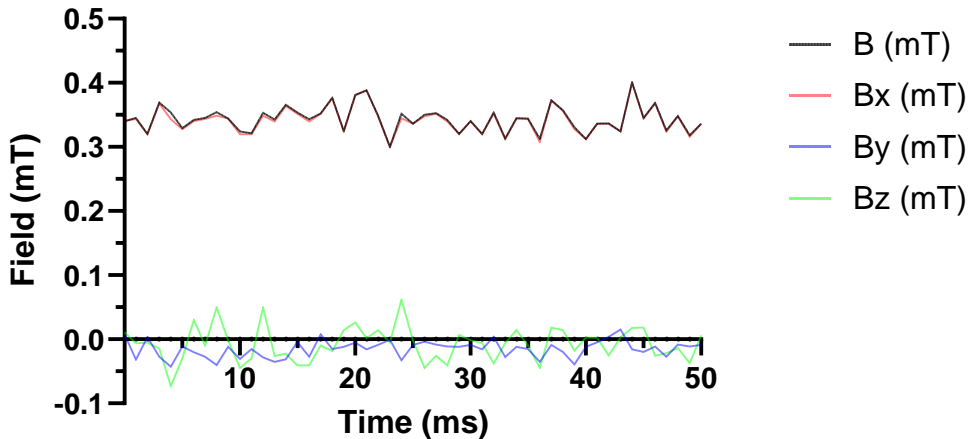

Supplement: Supplementary file 4 — Source Data [file 41467_2024_46407_MOESM4_ESM.zip › Data_Sorted_by_Figures/Fig_4/4H/Field Characterization/x axis measurements/(5,0,0) B 17A W 49A RMF off.pdf]

# **(-5,0,0) B-16A W-49A RMF on**

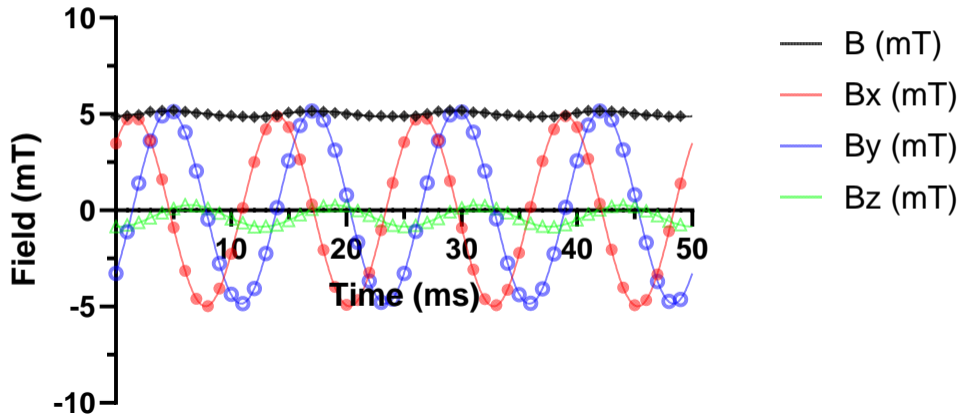

Supplement: Supplementary file 4 — Source Data [file 41467_2024_46407_MOESM4_ESM.zip › Data_Sorted_by_Figures/Fig_4/4H/Field Characterization/x axis measurements/(-5,0,0) B-16A W-49A RMF on.pdf]

**(-5,0,0) B 17A W 49A RMF on**

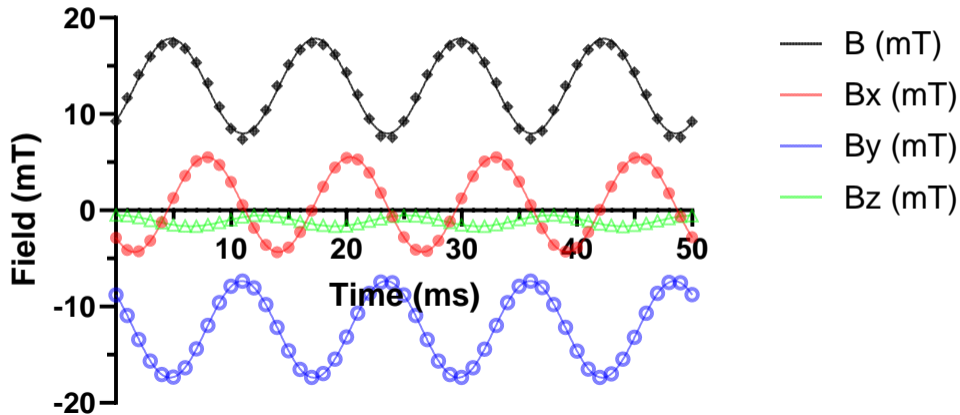

Supplement: Supplementary file 4 — Source Data [file 41467_2024_46407_MOESM4_ESM.zip › Data_Sorted_by_Figures/Fig_4/4H/Field Characterization/x axis measurements/(-5,0,0) B 17A W 49A RMF on.pdf]

**(-5,0,0) B -16A W -49A RMF off**

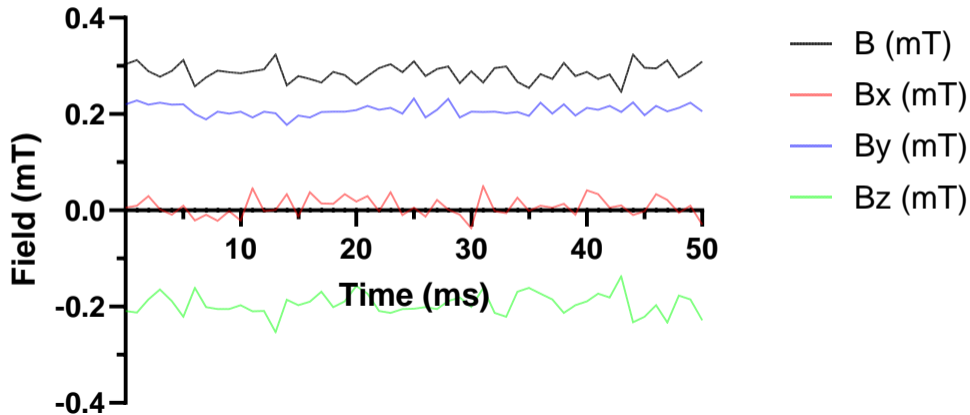

Supplement: Supplementary file 4 — Source Data [file 41467_2024_46407_MOESM4_ESM.zip › Data_Sorted_by_Figures/Fig_4/4H/Field Characterization/x axis measurements/(-5,0,0) B -16A W -49A RMF off.pdf]

# **(5,0,0) B0 W0 RMF off**

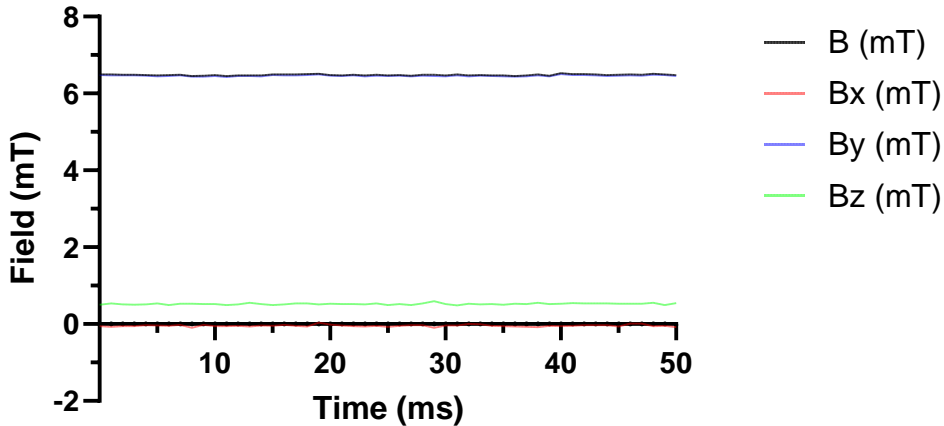

Supplement: Supplementary file 4 — Source Data [file 41467_2024_46407_MOESM4_ESM.zip › Data_Sorted_by_Figures/Fig_4/4H/Field Characterization/x axis measurements/(5,0,0) B0 W0 RMF off.pdf]

# **(5,0,0) B-16A W-49A RMF on**

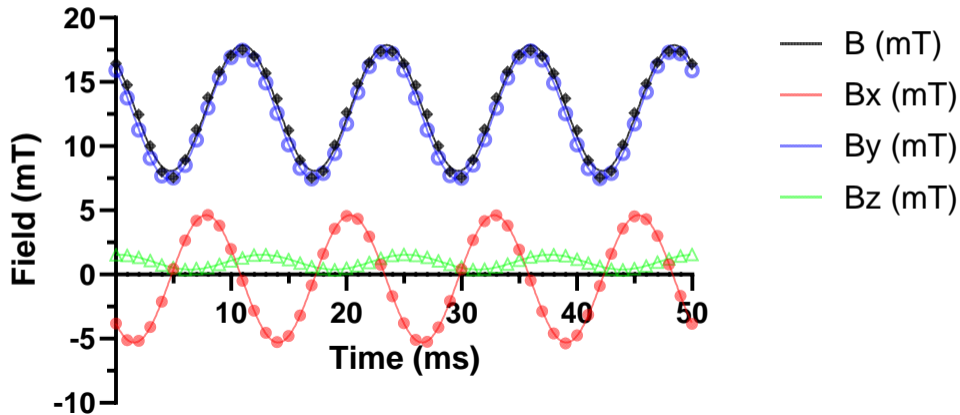

Supplement: Supplementary file 4 — Source Data [file 41467_2024_46407_MOESM4_ESM.zip › Data_Sorted_by_Figures/Fig_4/4H/Field Characterization/x axis measurements/(5,0,0) B-16A W-49A RMF on.pdf]

**(5,0,0) B -16A W -49A RMF off**

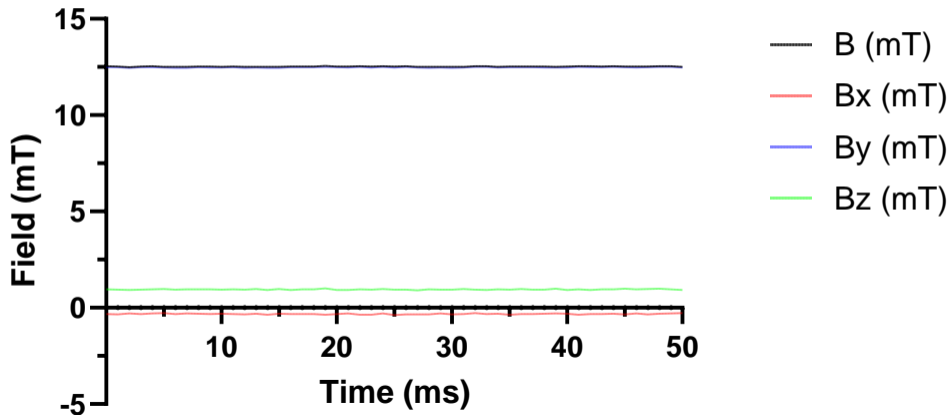

Supplement: Supplementary file 4 — Source Data [file 41467_2024_46407_MOESM4_ESM.zip › Data_Sorted_by_Figures/Fig_4/4H/Field Characterization/x axis measurements/(5,0,0) B -16A W -49A RMF off.pdf]

# **(5,0,0) B 17A W 49A RMF on**

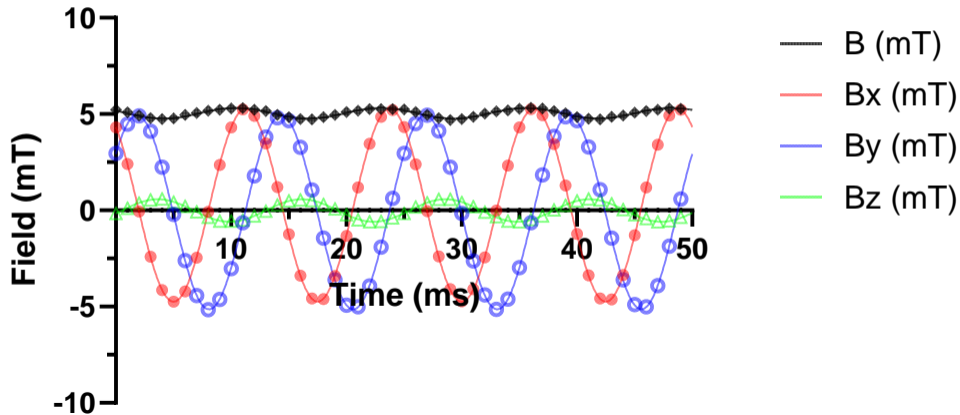

Supplement: Supplementary file 4 — Source Data [file 41467_2024_46407_MOESM4_ESM.zip › Data_Sorted_by_Figures/Fig_4/4H/Field Characterization/x axis measurements/(5,0,0) B 17A W 49A RMF on.pdf]

**(-5,0,0) B0 W0 RMF off**

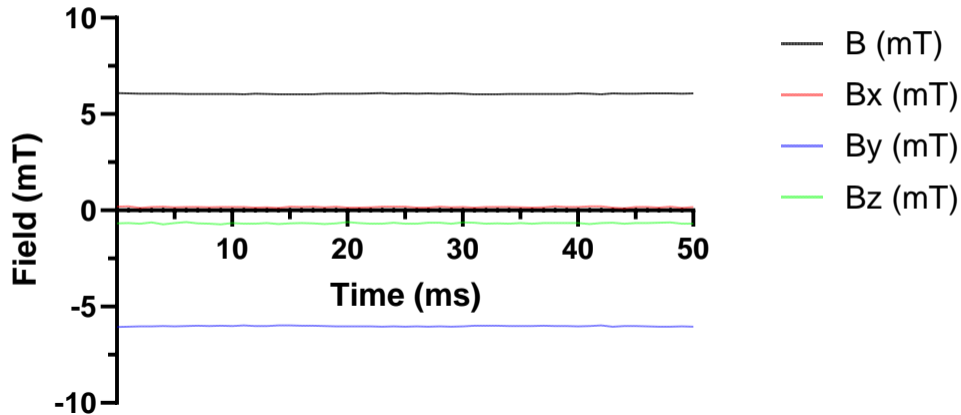

Supplement: Supplementary file 4 — Source Data [file 41467_2024_46407_MOESM4_ESM.zip › Data_Sorted_by_Figures/Fig_4/4H/Field Characterization/x axis measurements/(-5,0,0) B0 W0 RMF off.pdf]

**(0,-5,0) B -12.8A W 24.7A RMF off**

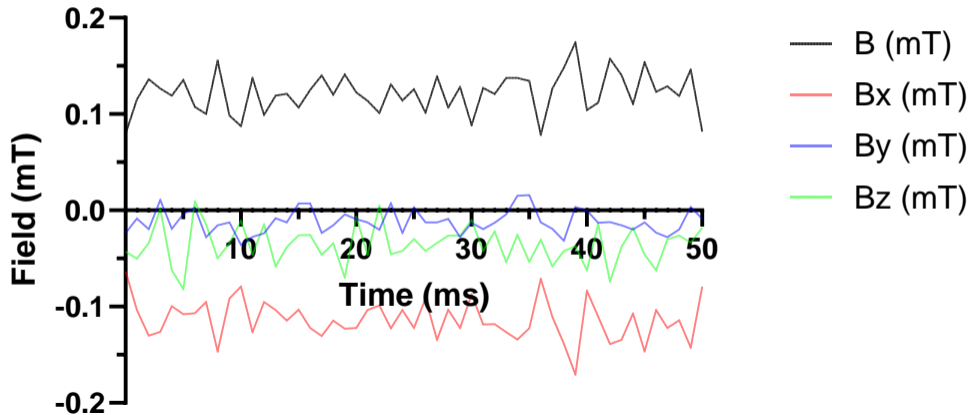

Supplement: Supplementary file 4 — Source Data [file 41467_2024_46407_MOESM4_ESM.zip › Data_Sorted_by_Figures/Fig_4/4I/Field Characterization/y axis measurements/(0,-5,0) B -12.8A W 24.7A RMF off.pdf]

**(0,5,0) B 13.4A W -29A RMF on**

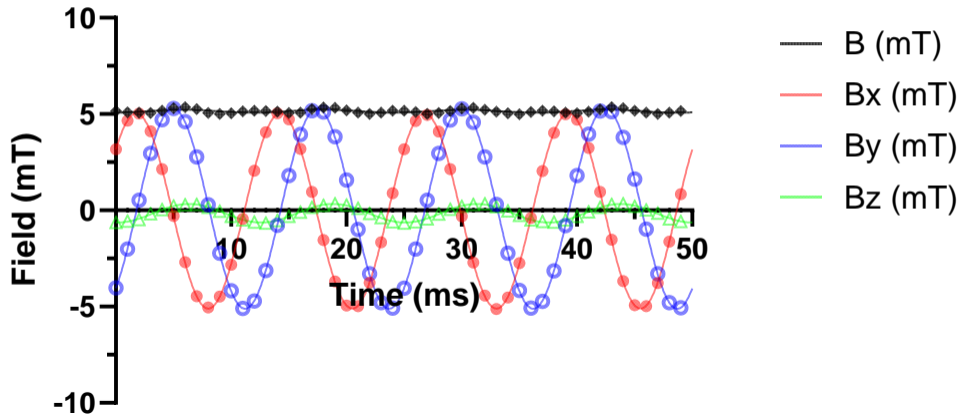

Supplement: Supplementary file 4 — Source Data [file 41467_2024_46407_MOESM4_ESM.zip › Data_Sorted_by_Figures/Fig_4/4I/Field Characterization/y axis measurements/(0,5,0) B 13.4A W -29A RMF on.pdf]

**(0,-5,0) B 13.4A W -29A RMF on**

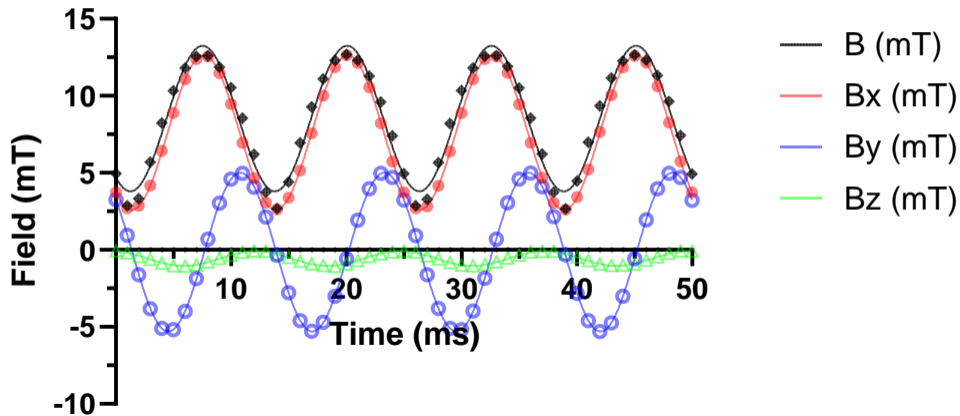

Supplement: Supplementary file 4 — Source Data [file 41467_2024_46407_MOESM4_ESM.zip › Data_Sorted_by_Figures/Fig_4/4I/Field Characterization/y axis measurements/(0,-5,0) B 13.4A W -29A RMF on.pdf]

# **(0,-5,0) B0 W0 RMF off**

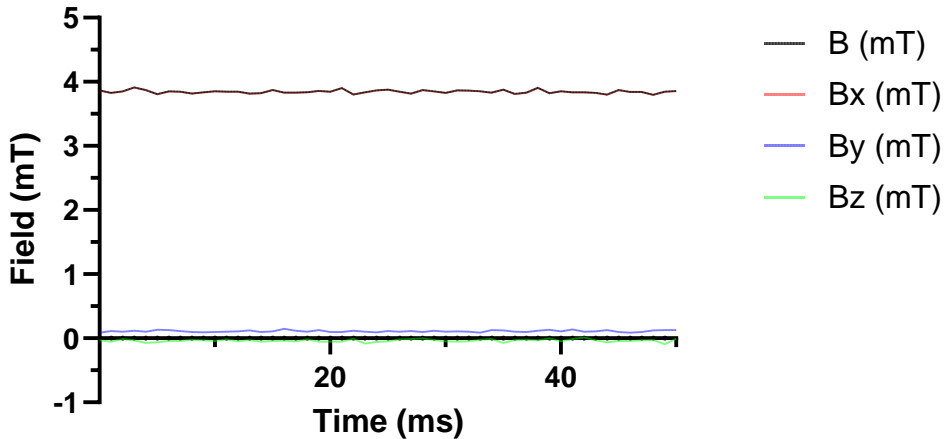

Supplement: Supplementary file 4 — Source Data [file 41467_2024_46407_MOESM4_ESM.zip › Data_Sorted_by_Figures/Fig_4/4I/Field Characterization/y axis measurements/(0,-5,0) B0 W0 RMF off.pdf]

# **(0,5,0) B 13.4A W -29A RMF off**

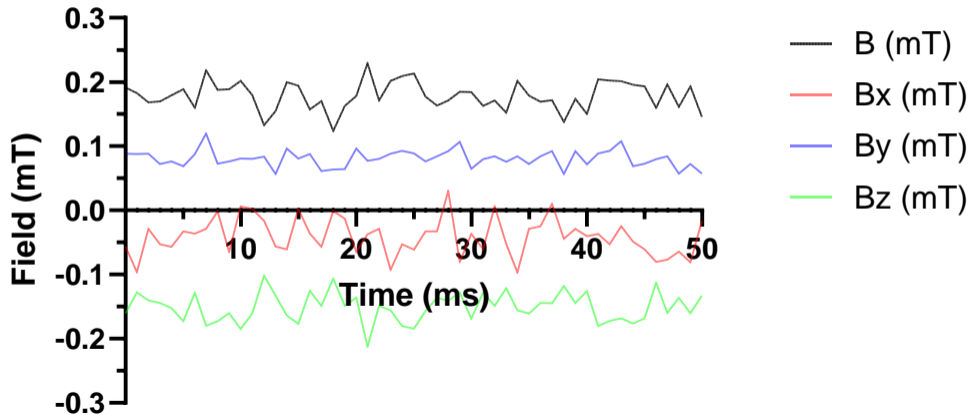

Supplement: Supplementary file 4 — Source Data [file 41467_2024_46407_MOESM4_ESM.zip › Data_Sorted_by_Figures/Fig_4/4I/Field Characterization/y axis measurements/(0,5,0) B 13.4A W -29A RMF off.pdf]

# **(0,-5,0) B-12.8A W24.7A RMF on**

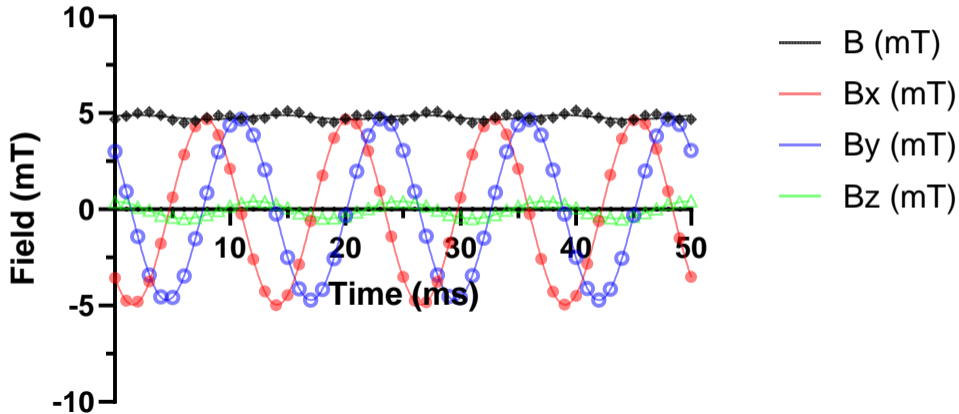

Supplement: Supplementary file 4 — Source Data [file 41467_2024_46407_MOESM4_ESM.zip › Data_Sorted_by_Figures/Fig_4/4I/Field Characterization/y axis measurements/(0,-5,0) B-12.8A W24.7A RMF on.pdf]

# **(0,5,0) B-12.8A W24.7A RMF on**

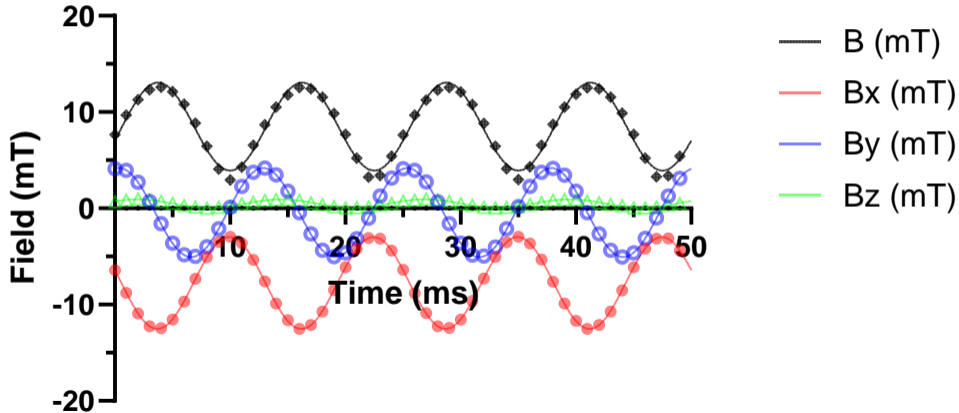

Supplement: Supplementary file 4 — Source Data [file 41467_2024_46407_MOESM4_ESM.zip › Data_Sorted_by_Figures/Fig_4/4I/Field Characterization/y axis measurements/(0,5,0) B-12.8A W24.7A RMF on.pdf]

**(0,-5,0) B 13.4A W -29A RMF off**

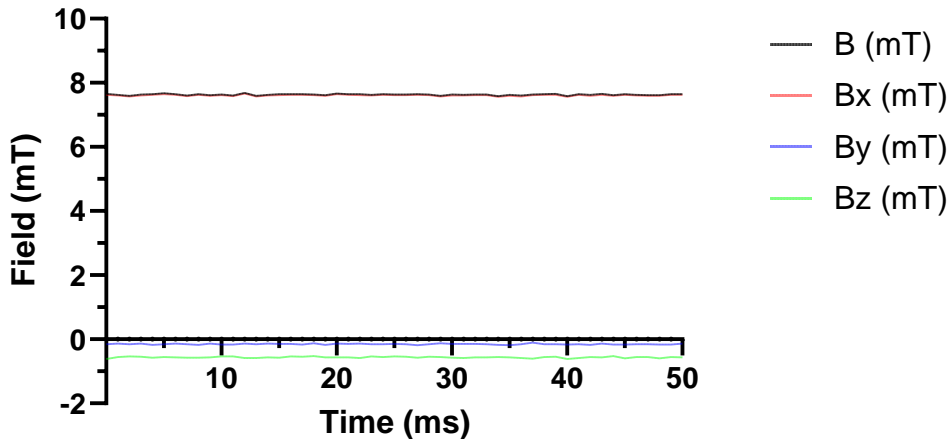

Supplement: Supplementary file 4 — Source Data [file 41467_2024_46407_MOESM4_ESM.zip › Data_Sorted_by_Figures/Fig_4/4I/Field Characterization/y axis measurements/(0,-5,0) B 13.4A W -29A RMF off.pdf]

**(0,-5,0) B0 W0 RMF on**

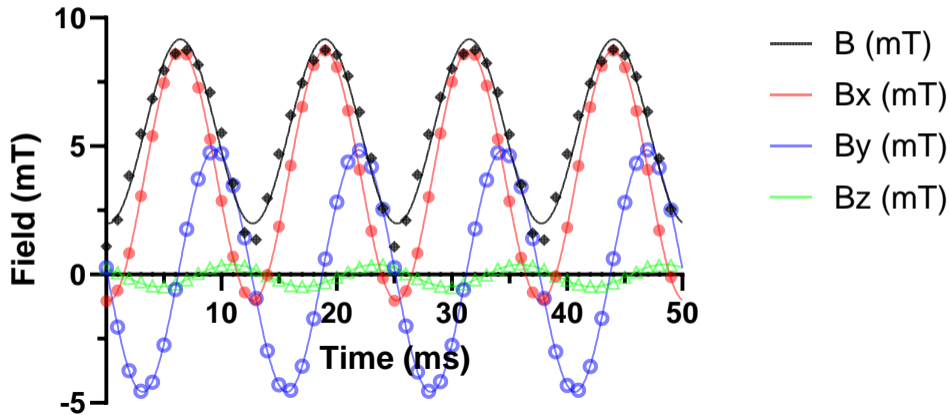

Supplement: Supplementary file 4 — Source Data [file 41467_2024_46407_MOESM4_ESM.zip › Data_Sorted_by_Figures/Fig_4/4I/Field Characterization/y axis measurements/(0,-5,0) B0 W0 RMF on.pdf]

# $(0,5,0)$ B0 W0 RMF on

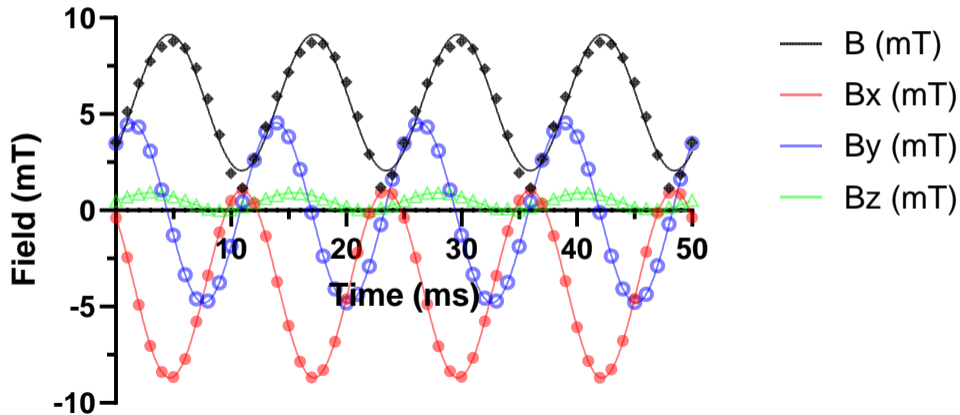

Supplement: Supplementary file 4 — Source Data [file 41467_2024_46407_MOESM4_ESM.zip › Data_Sorted_by_Figures/Fig_4/4I/Field Characterization/y axis measurements/(0,5,0) B0 W0 RMF on.pdf]

# **(0,5,0) B0 W0 RMF off**

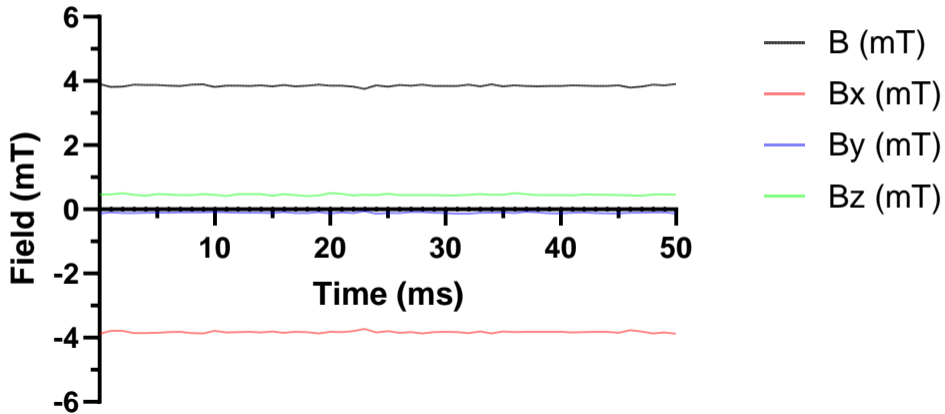

Supplement: Supplementary file 4 — Source Data [file 41467_2024_46407_MOESM4_ESM.zip › Data_Sorted_by_Figures/Fig_4/4I/Field Characterization/y axis measurements/(0,5,0) B0 W0 RMF off.pdf]

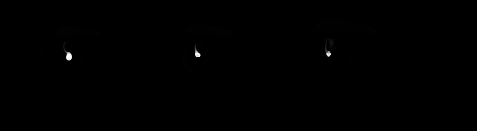

Supplement: Supplementary file 4 — Source Data [file 41467_2024_46407_MOESM4_ESM.zip › Data_Sorted_by_Figures/Fig_4/4H/Mixing/230227 x +5mm/x5_y0_z0/x5_y0_z0_on_target_prescan_520.tif]

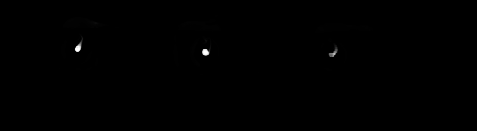

Supplement: Supplementary file 4 — Source Data [file 41467_2024_46407_MOESM4_ESM.zip › Data_Sorted_by_Figures/Fig_4/4H/Mixing/230227 x +5mm/x5_y0_z0/x5_y0_z0_off_target_prescan_520.tif]

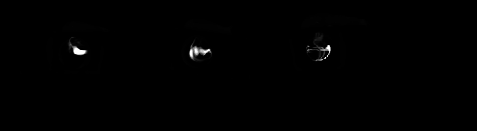

Supplement: Supplementary file 4 — Source Data [file 41467_2024_46407_MOESM4_ESM.zip › Data_Sorted_by_Figures/Fig_4/4H/Mixing/230227 x +5mm/x5_y0_z0/x5_y0_z0_on_target_postscan_520.tif]

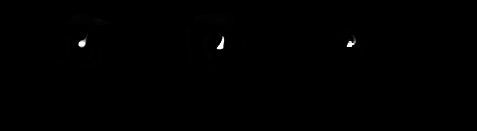

Supplement: Supplementary file 4 — Source Data [file 41467_2024_46407_MOESM4_ESM.zip › Data_Sorted_by_Figures/Fig_4/4H/Mixing/230227 x +5mm/x5_y0_z0/x5_y0_z0_off_target_postscan_520.tif]
