# Supplementary material for: Spatially selective delivery of living magnetic microrobots through torque-focusing
Source: Nat Commun. 2024 Mar 9;15:2160. doi: 10.1038/s41467-024-46407-4 (PMC10924878; doi:10.1038/s41467-024-46407-4)
Supplement: Supplementary file 4 — Source Data [file 41467_2024_46407_MOESM4_ESM.zip › Data_Sorted_by_Figures/Fig_4/4I/Field Characterization/y axis measurements/x0_y5_z0/ReadingHallProbeData.pdf]

```
NotebookDirectory[]
```

Notebook–Verzeichnis

```
P:\Michael\MetroLabHallProbe\230224\x0_y0_z0\
```

(0,5,0) Blue offset 0A White Offset 0A RMF off

```
Data = Import[NotebookDirectory[] <> "x0_y5_z0_B0_W0_RMFoff.txt", "Table"];
```

importi··· Notebook–Verzeichnis Tabelle

```
Bvst = Table[{AbsoluteTime[Data[[i]][[9]]] - AbsoluteTime[Data[[2]][[9]]],
    AbsoluteTime[Data[[i]][[2]]]}, {i, 2, Length[Data]}];
Bxvst = Table[{AbsoluteTime[Data[[i]][[9]]] - AbsoluteTime[Data[[2]][[9]]],
    AbsoluteTime[Data[[i]][[3]]]}, {i, 2, Length[Data]}];
Byvst = Table[{AbsoluteTime[Data[[i]][[9]]] - AbsoluteTime[Data[[2]][[9]]],
    AbsoluteTime[Data[[i]][[4]]]}, {i, 2, Length[Data]}];
Bzvst = Table[{AbsoluteTime[Data[[i]][[9]]] - AbsoluteTime[Data[[2]][[9]]],
    AbsoluteTime[Data[[i]][[5]]]}, {i, 2, Length[Data]}];
```

Tabelle absolute Zeit seit 1900 absolute Zeit seit 1900 Länge

```
ListPlot[{Bvst, Bxvst, Byvst, Bzvst}, Joined → True]
```

listenbezogene Graphik verknüpft? wahr

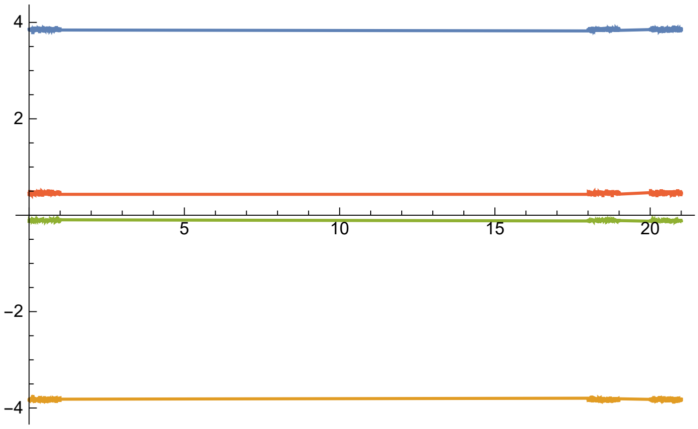


```
Start = 100;
Stop = 1000;
```

```
ListPlot[{Table[{Bvst[[i]][[1]], Bvst[[i]][[2]]}, {i, Start, Stop}],
listenbezo⋯ Tabelle
  Table[{Bxvst[[i]][[1]], Bxvst[[i]][[2]]}, {i, Start, Stop}],
  Tabelle
  Table[{Byvst[[i]][[1]], Byvst[[i]][[2]]}, {i, Start, Stop}],
  Tabelle
  Table[{Bzvst[[i]][[1]], Bzvst[[i]][[2]]}, {i, Start, Stop}]}, Joined → True]
  Tabelle                                                        verknüpft? wahr
```

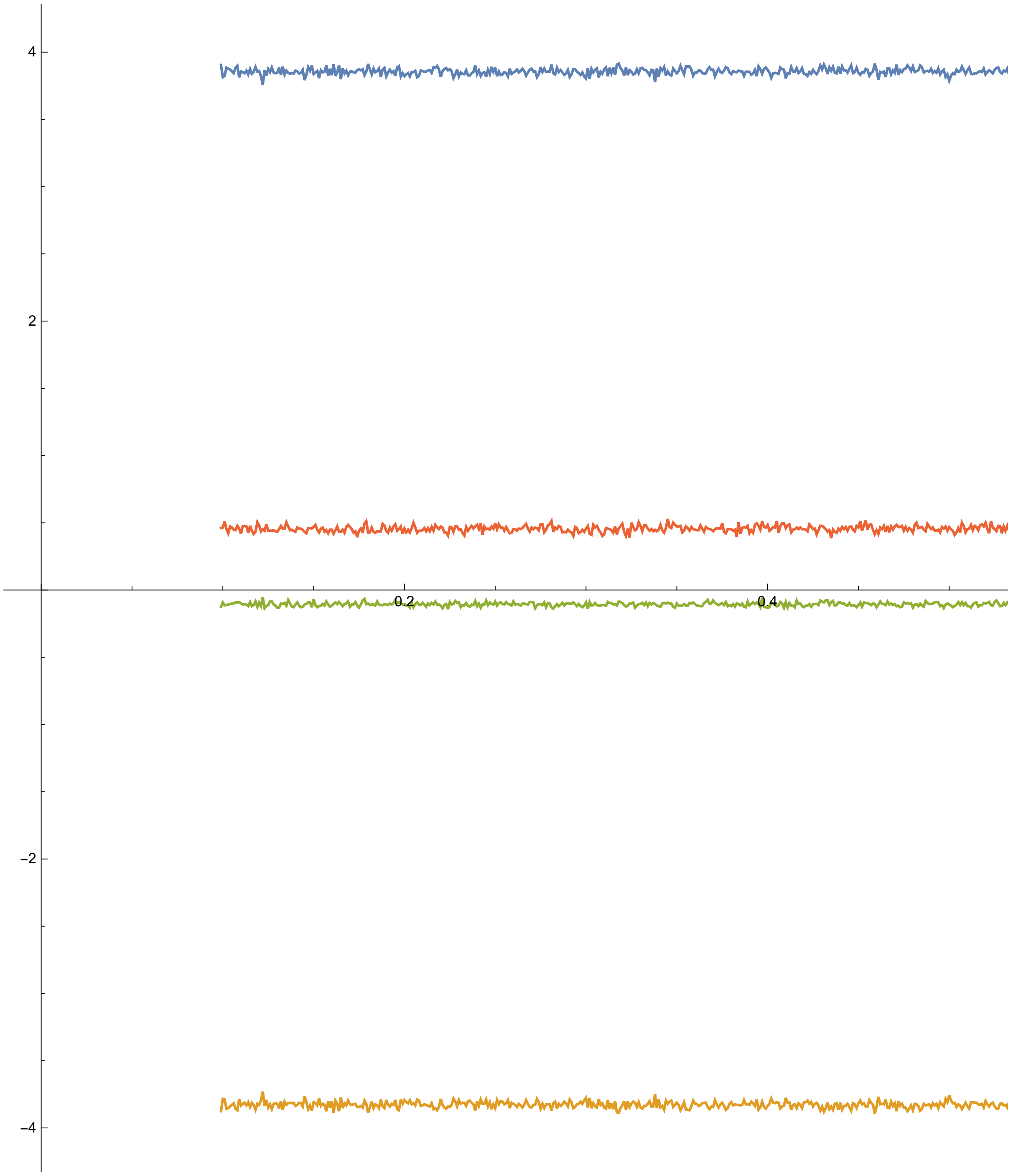

```
ListPlot[{Table[{Bvst[[i]][[1]], Bvst[[i]][[2]]}, {i, Start, Start + 50}],
  Table[{Bxvst[[i]][[1]], Bxvst[[i]][[2]]}, {i, Start, Start + 50}],
  Table[{Byvst[[i]][[1]], Byvst[[i]][[2]]}, {i, Start, Start + 50}],
  Table[{Bzvst[[i]][[1]], Bzvst[[i]][[2]]}, {i, Start, Start + 50}]}, Joined → True]
```

listenbezo··· Tabelle Tabelle Tabelle Tabelle verknüpft? wahr

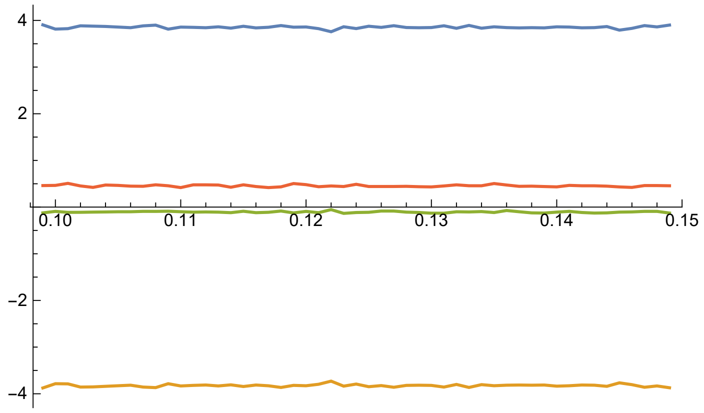


```
TableForm[{Mean[Table[Bvst[[i]][[2]], {i, Start, Stop}]],
  Mean[Table[Bxvst[[i]][[2]], {i, Start, Stop}]],
  Mean[Table[Byvst[[i]][[2]], {i, Start, Stop}]],
  Mean[Table[Bzvst[[i]][[2]], {i, Start, Stop}]]}, TableDirections → Row]
```

Tabellendars··· arit··· Tabelle arit··· Tabelle arit··· Tabelle arit··· Tabelle Richtung der Tabellen··· Zeile

3.85607	−3.82729	−0.107122	0.457014

```
TableForm[Table[{(Bvst[[i]][[1]] - Bvst[[Start]][[1]]) * 1000, Bvst[[i]][[2]],
 Bxvst[[i]][[2]], Byvst[[i]][[2]], Bzvst[[i]][[2]]}, {i, Start, Start + 50}]]
```
Tabellendar·· Tabelle

0.	3.90463	-3.87531	-0.124048	0.461138
0.999928	3.81385	-3.78431	-0.092005	0.46469
1.99986	3.82347	-3.78784	-0.112679	0.508418
2.99978	3.88455	-3.85641	-0.111904	0.453127
3.99971	3.87728	-3.85273	-0.107427	0.422152
5.00011	3.87065	-3.84021	-0.104192	0.473081
6.00004	3.85774	-3.8283	-0.100056	0.46502
6.99997	3.84407	-3.81647	-0.099797	0.448899
7.9999	3.88324	-3.85651	-0.091785	0.445434
8.99982	3.89864	-3.86818	-0.092287	0.477552
9.99975	3.8129	-3.7844	-0.088883	0.456737
10.9997	3.8568	-3.83279	-0.099343	0.418069
12.0001	3.85133	-3.82017	-0.108229	0.476814
13.	3.84329	-3.81217	-0.10422	0.476792
13.9999	3.8628	-3.83221	-0.108182	0.472938
14.9999	3.83424	-3.80868	-0.119434	0.425515
15.9998	3.87474	-3.84419	-0.089259	0.477351
16.9997	3.83976	-3.81253	-0.119668	0.440554
17.9996	3.85311	-3.82877	-0.111337	0.417845
19.0001	3.88982	-3.86464	-0.084612	0.433625
20.	3.85506	-3.81986	-0.124654	0.504564
20.9999	3.85937	-3.82814	-0.092301	0.48114
21.9998	3.82344	-3.79657	-0.119588	0.43639
22.9998	3.75724	-3.72948	-0.053761	0.452706
23.9997	3.86407	-3.83651	-0.134695	0.440572
25.0001	3.82513	-3.79204	-0.116378	0.488401
26.	3.87534	-3.84853	-0.111712	0.441047
27.	3.85086	-3.82456	-0.084686	0.441212
27.9999	3.88666	-3.86056	-0.084729	0.441583
28.9998	3.84782	-3.8205	-0.10774	0.444819
29.9997	3.84321	-3.81657	-0.115612	0.436657
30.9997	3.84721	-3.8206	-0.130554	0.43247
32.0001	3.88509	-3.85639	-0.130902	0.452837
33.	3.83128	-3.80018	-0.100206	0.47673
33.9999	3.89273	-3.86438	-0.103976	0.457331
34.9998	3.83292	-3.80439	-0.095906	0.456836
35.9998	3.86277	-3.82787	-0.116665	0.504769
36.9997	3.84618	-3.81625	-0.073167	0.473308
38.0001	3.83967	-3.81251	-0.099731	0.444859
39.	3.8447	-3.81644	-0.123794	0.448533
40.	3.84	-3.81252	-0.127667	0.440432
40.9999	3.86248	-3.83662	-0.107576	0.432986
41.9998	3.85756	-3.82831	-0.092057	0.465142
42.9997	3.84136	-3.81237	-0.115913	0.456612
43.9997	3.84569	-3.81636	-0.127916	0.45647
45.0001	3.86856	-3.84044	-0.123823	0.44878
46.	3.79188	-3.76563	-0.107492	0.432255
46.9999	3.82944	-3.80474	-0.10337	0.421719
47.9999	3.88892	-3.86035	-0.092034	0.461472
48.9998	3.8611	-3.83235	-0.092	0.461184
49.9997	3.90034	-3.87135	-0.127981	0.457036

(0,5,0) Blue offset 0A White Offset 0A RMF on

```
Data = Import[NotebookDirectory[] <> "x0_y5_z0_B0_W0_RMFon.txt", "Table"];
```
importi··· Notebook-Verzeichnis Tabelle

```
Bvst = Table[{AbsoluteTime[Data[[i]][[9]]] - AbsoluteTime[Data[[2]][[9]]],
        Tabelle  absolute Zeit seit 1900                absolute Zeit seit 1900
    AbsoluteTime[Data[[i]][[2]]]}, {i, 2, Length[Data]}];
    absolute Zeit seit 1900                              Länge
Bxvst = Table[{AbsoluteTime[Data[[i]][[9]]] - AbsoluteTime[Data[[2]][[9]]],
         Tabelle  absolute Zeit seit 1900                absolute Zeit seit 1900
    AbsoluteTime[Data[[i]][[3]]]}, {i, 2, Length[Data]}];
    absolute Zeit seit 1900                              Länge
Byvst = Table[{AbsoluteTime[Data[[i]][[9]]] - AbsoluteTime[Data[[2]][[9]]],
         Tabelle  absolute Zeit seit 1900                absolute Zeit seit 1900
    AbsoluteTime[Data[[i]][[4]]]}, {i, 2, Length[Data]}];
    absolute Zeit seit 1900                              Länge
Bzvst = Table[{AbsoluteTime[Data[[i]][[9]]] - AbsoluteTime[Data[[2]][[9]]],
         Tabelle  absolute Zeit seit 1900                absolute Zeit seit 1900
    AbsoluteTime[Data[[i]][[5]]]}, {i, 2, Length[Data]}];
    absolute Zeit seit 1900                              Länge
```

```
ListPlot[{Bvst, Bxvst, Byvst, Bzvst}, Joined → True]
listenbezogene Graphik                 verknüpft? wahr
```

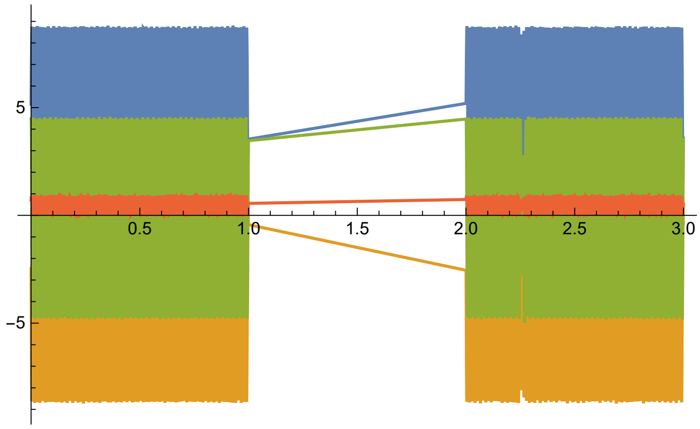


```
Start = 100;
Stop = 1000;
```

```
ListPlot[{Table[{Bvst[[i]][[1]], Bvst[[i]][[2]]}, {i, Start, Stop}],
  Table[{Bxvst[[i]][[1]], Bxvst[[i]][[2]]}, {i, Start, Stop}],
  Table[{Byvst[[i]][[1]], Byvst[[i]][[2]]}, {i, Start, Stop}],
  Table[{Bzvst[[i]][[1]], Bzvst[[i]][[2]]}, {i, Start, Stop}]}, Joined → True]
```

listenbezo··· Tabelle Tabelle Tabelle Tabelle verknüpft? wahr

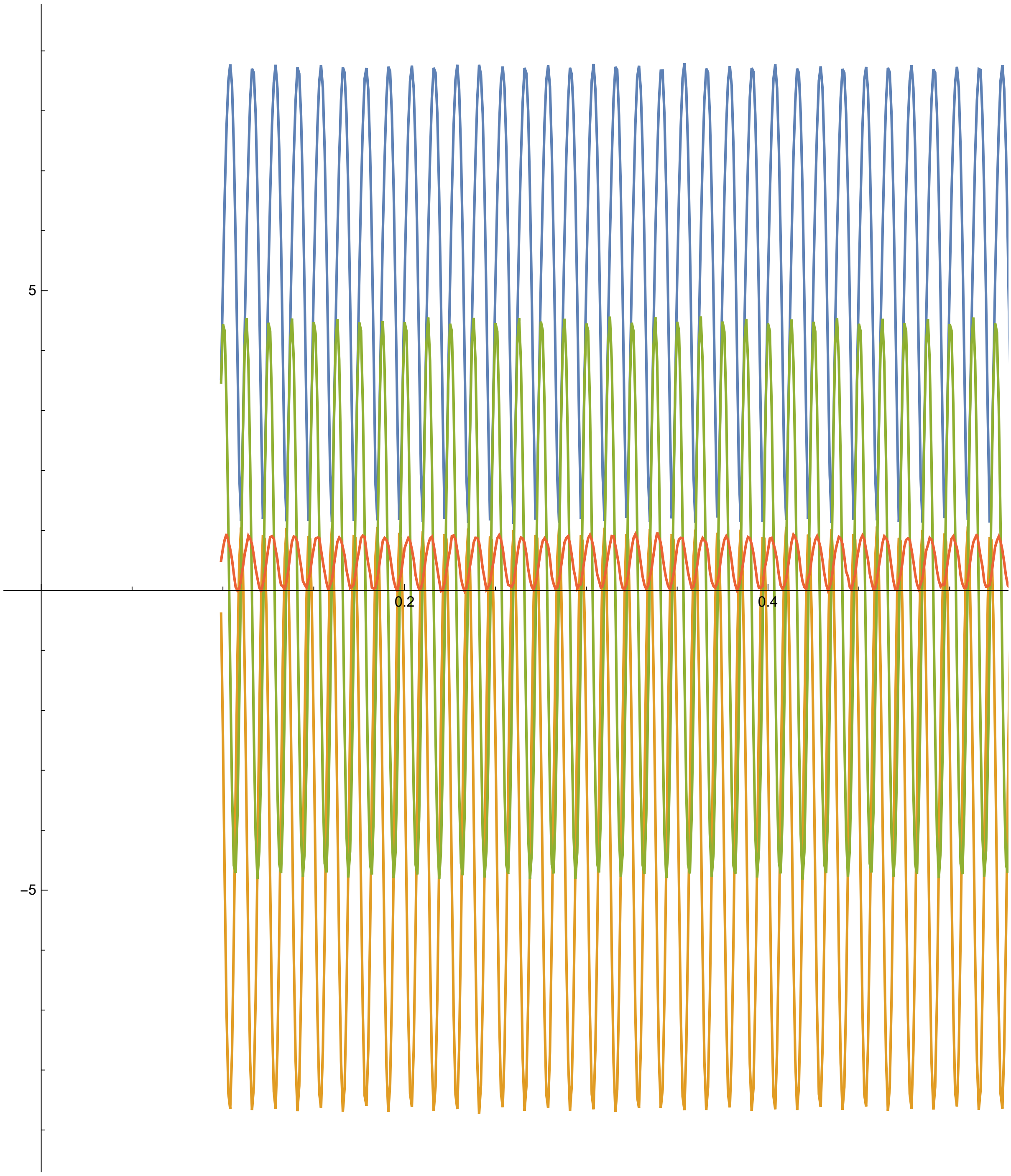

```
ListPlot[{Table[{Bvst[[i]][[1]], Bvst[[i]][[2]]}, {i, Start, Start + 50}],
 listenbezo··· Tabelle
  Table[{Bxvst[[i]][[1]], Bxvst[[i]][[2]]}, {i, Start, Start + 50}],
  Tabelle
  Table[{Byvst[[i]][[1]], Byvst[[i]][[2]]}, {i, Start, Start + 50}],
  Tabelle
  Table[{Bzvst[[i]][[1]], Bzvst[[i]][[2]]}, {i, Start, Start + 50}]}, Joined → True]
  Tabelle                                                              verknüpft? wahr
```

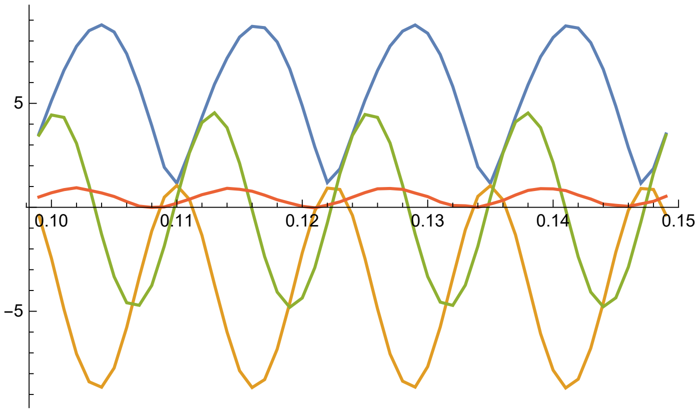


```
TableForm[{Mean[Table[Bvst[[i]][[2]], {i, Start, Stop}]],
 Tabellendars···arit··· Tabelle
  Mean[Table[Bxvst[[i]][[2]], {i, Start, Stop}]],
  arit··· Tabelle
  Mean[Table[Byvst[[i]][[2]], {i, Start, Stop}]],
  arit··· Tabelle
  Mean[Table[Bzvst[[i]][[2]], {i, Start, Stop}]]}, TableDirections → Row]
  arit··· Tabelle                                   Richtung der Tabellen··· Zeile
```

```
5.5885  -3.82433  -0.124151  0.463105
```

```
TableForm[Table[{(Bvst[[i]][[1]] - Bvst[[Start]][[1]]) * 1000, Bvst[[i]][[2]],
    Bxvst[[i]][[2]], Byvst[[i]][[2]], Bzvst[[i]][[2]]}, {i, Start, Start + 50}]]
```
Tabellendar·· Tabelle

0.	3.52613	-0.388031	3.46943	0.496052
0.999928	5.11984	-2.4492	4.44132	0.699174
1.99986	6.59095	-4.90165	4.32348	0.849685
2.99978	7.74521	-7.04535	3.07674	0.940767
3.99971	8.49597	-8.39026	1.05497	0.819747
4.99964	8.77488	-8.65081	-1.29762	0.691494
6.00004	8.43315	-7.72811	-3.33529	0.519844
6.99997	7.38432	-5.77905	-4.58866	0.273572
7.9999	5.79561	-3.37401	-4.71189	0.056859
8.99982	3.92754	-1.13964	-3.75856	-0.003633
9.99975	1.92295	0.487445	-1.86003	0.020581
10.9997	1.15746	1.05056	0.444124	0.196983
12.0001	2.67086	0.381913	2.61477	0.388048
13.	4.33464	-1.34466	4.07582	0.607191
13.9999	5.91692	-3.71179	4.54514	0.757786
14.9999	7.17236	-5.99347	3.83233	0.913454
15.9998	8.18643	-7.85902	2.12192	0.866567
16.9997	8.70258	-8.66737	-0.113754	0.773753
17.9996	8.63688	-8.28463	-2.37072	0.583318
19.0001	7.95287	-6.81978	-4.07506	0.364159
20.	6.66128	-4.60445	-4.80941	0.203081
20.9999	4.87734	-2.18643	-4.35953	0.049994
21.9998	2.90533	-0.171821	-2.90014	-0.024509
22.9998	1.193	0.924048	-0.746788	0.108091
23.9997	1.84901	0.858845	1.61604	0.263862
25.0001	3.5133	-0.388101	3.45755	0.48787
26.	5.14885	-2.46415	4.46518	0.707694
27.	6.58338	-4.88224	4.32589	0.889528
27.9999	7.75386	-7.05273	3.09228	0.905075
28.9998	8.47264	-8.36182	1.05833	0.863512
29.9997	8.76863	-8.6469	-1.28549	0.683638
30.9997	8.3699	-7.66116	-3.33115	0.515216
32.0001	7.34741	-5.75529	-4.56033	0.253757
33.	5.81114	-3.39367	-4.71641	0.088998
33.9999	3.89779	-1.08288	-3.74365	0.072023
34.9998	1.94556	0.527291	-1.87274	0.003974
35.9998	1.15185	1.04212	0.464722	0.157378
36.9997	2.65057	0.362348	2.60461	0.332085
37.9996	4.35302	-1.32185	4.10509	0.5914
39.	5.89464	-3.67521	4.53632	0.813284
40.	7.24018	-6.07663	3.83247	0.898311
40.9999	8.16854	-7.83084	2.14865	0.886688
41.9998	8.72493	-8.68705	-0.106252	0.805076
42.9997	8.62107	-8.26558	-2.37875	0.587
43.9997	7.92026	-6.77941	-4.07556	0.399741
45.0001	6.6342	-4.59693	-4.78073	0.159434
46.	4.85746	-2.15496	-4.35221	0.096789
46.9999	2.87417	-0.143112	-2.87021	0.047664
47.9999	1.15846	0.904582	-0.704672	0.164944
48.9998	1.86288	0.85908	1.62767	0.288041
49.9997	3.52811	-0.367702	3.46896	0.527841

(0,5,0) Blue offset -12.8A White Offset 24.7A RMF on

```
Data = Import[NotebookDirectory[] <> "x0_y5_z0_B-12_8_W24_7_RMFon.txt", "Table"];
```
importi··· Notebook-Verzeichnis Tabelle

```
Bvst = Table[{AbsoluteTime[Data[[i]][[9]]] - AbsoluteTime[Data[[2]][[9]]],
         Tabelle  absolute Zeit seit 1900                  absolute Zeit seit 1900
    AbsoluteTime[Data[[i]][[2]]]}, {i, 2, Length[Data]}];
    absolute Zeit seit 1900                       Länge
Bxvst = Table[{AbsoluteTime[Data[[i]][[9]]] - AbsoluteTime[Data[[2]][[9]]],
          Tabelle  absolute Zeit seit 1900                  absolute Zeit seit 1900
    AbsoluteTime[Data[[i]][[3]]]}, {i, 2, Length[Data]}];
    absolute Zeit seit 1900                       Länge
Byvst = Table[{AbsoluteTime[Data[[i]][[9]]] - AbsoluteTime[Data[[2]][[9]]],
          Tabelle  absolute Zeit seit 1900                  absolute Zeit seit 1900
    AbsoluteTime[Data[[i]][[4]]]}, {i, 2, Length[Data]}];
    absolute Zeit seit 1900                       Länge
Bzvst = Table[{AbsoluteTime[Data[[i]][[9]]] - AbsoluteTime[Data[[2]][[9]]],
          Tabelle  absolute Zeit seit 1900                  absolute Zeit seit 1900
    AbsoluteTime[Data[[i]][[5]]]}, {i, 2, Length[Data]}];
    absolute Zeit seit 1900                       Länge
```

```
ListPlot[{Bvst, Bxvst, Byvst, Bzvst}, Joined → True]
listenbezogene Graphik                      verknüpft? wahr
```

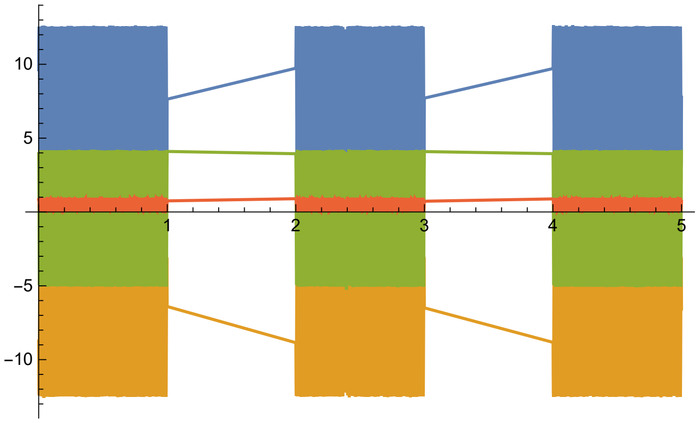


```
Start = 100;
Stop = 1000;
```

```
ListPlot[{Table[{Bvst[[i]][[1]], Bvst[[i]][[2]]}, {i, Start, Stop}],
  Table[{Bxvst[[i]][[1]], Bxvst[[i]][[2]]}, {i, Start, Stop}],
  Table[{Byvst[[i]][[1]], Byvst[[i]][[2]]}, {i, Start, Stop}],
  Table[{Bzvst[[i]][[1]], Bzvst[[i]][[2]]}, {i, Start, Stop}]}, Joined → True]
```

listenbezo··· Tabelle Tabelle Tabelle Tabelle verknüpft? wahr

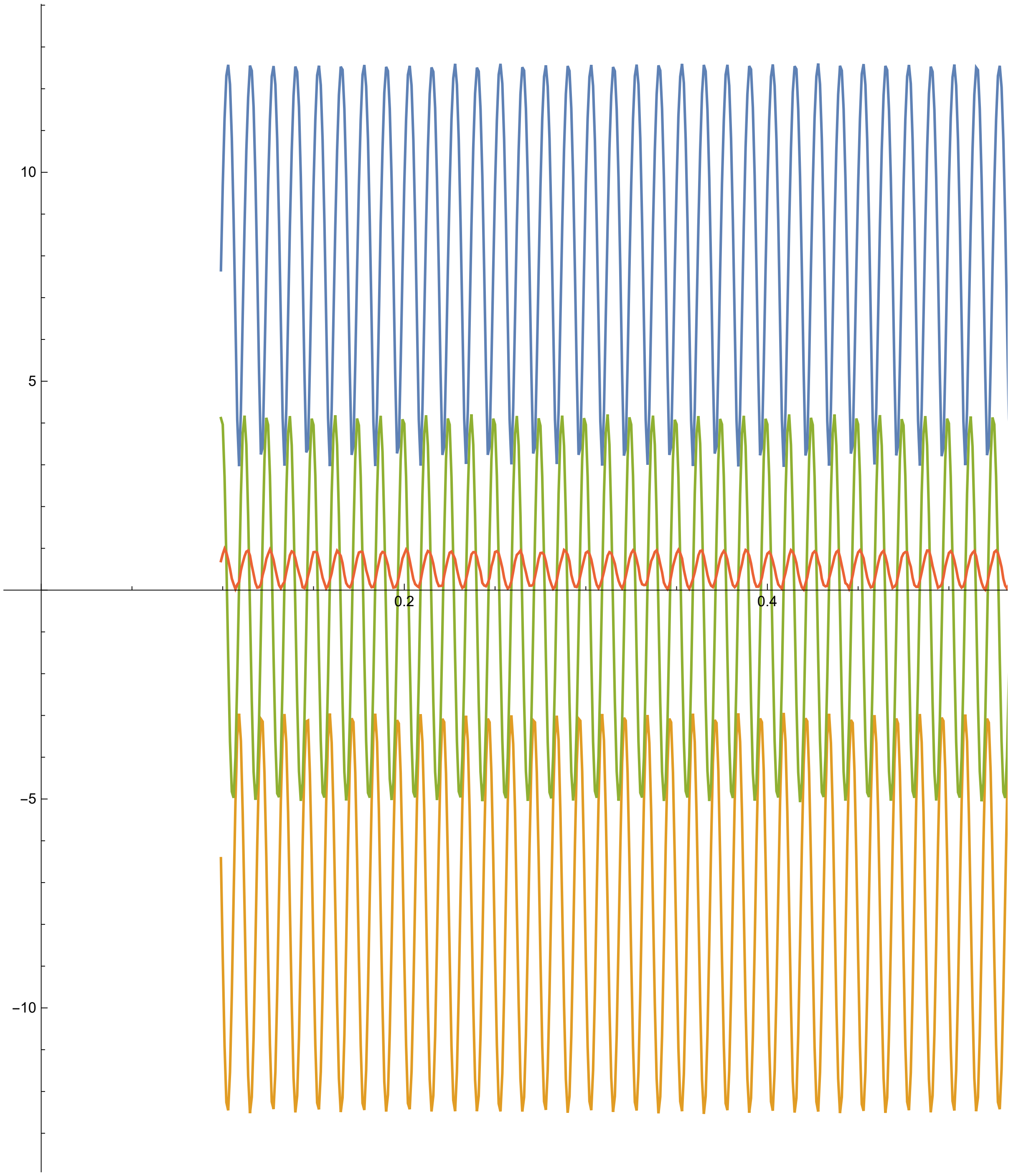

```
ListPlot[{Table[{Bvst[[i]][[1]], Bvst[[i]][[2]]}, {i, Start, Start + 50}],
  Table[{Bxvst[[i]][[1]], Bxvst[[i]][[2]]}, {i, Start, Start + 50}],
  Table[{Byvst[[i]][[1]], Byvst[[i]][[2]]}, {i, Start, Start + 50}],
  Table[{Bzvst[[i]][[1]], Bzvst[[i]][[2]]}, {i, Start, Start + 50}]}, Joined → True]
```

listenbezo··· Tabelle Tabelle Tabelle Tabelle verknüpft? wahr

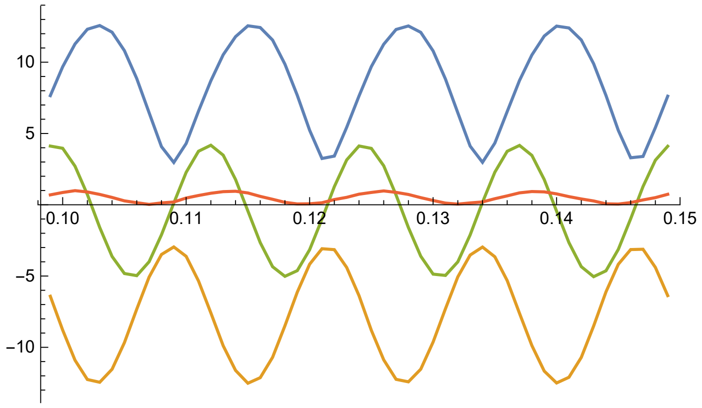


```
TableForm[{Mean[Table[Bvst[[i]][[2]], {i, Start, Stop}]],
  Mean[Table[Bxvst[[i]][[2]], {i, Start, Stop}]],
  Mean[Table[Byvst[[i]][[2]], {i, Start, Stop}]],
  Mean[Table[Bzvst[[i]][[2]], {i, Start, Stop}]]}, TableDirections → Row]
```

Tabellendars··· arit··· Tabelle arit··· Tabelle arit··· Tabelle arit··· Tabelle Richtung der Tabellen··· Zeile

8.47925 −7.74351 −0.43015 0.498344

```
TableForm[Table[{(Bvst[[i]][[1]] - Bvst[[Start]][[1]]) * 1000, Bvst[[i]][[2]],
Tabellendar·· Tabelle
   Bxvst[[i]][[2]], Byvst[[i]][[2]], Bzvst[[i]][[2]]}, {i, Start, Start + 50}]]
```

0.	7.65597	-6.41707	4.11726	0.695134
0.999928	9.68085	-8.79031	3.96326	0.861279
2.00033	11.2801	-10.9056	2.70697	0.989929
3.00026	12.3123	-12.2583	0.720743	0.898536
4.00019	12.5735	-12.4513	-1.59109	0.726202
5.00011	12.1051	-11.538	-3.62516	0.514697
6.00004	10.8111	-9.67198	-4.82271	0.274134
6.99997	8.85137	-7.32201	-4.97133	0.144616
7.9999	6.45795	-5.07227	-3.99707	0.025275
9.0003	4.0783	-3.48835	-2.10882	0.129791
10.0002	2.96605	-2.95731	0.120017	0.193354
11.0002	4.29728	-3.61603	2.27333	0.472067
12.0001	6.56451	-5.34505	3.755	0.650544
13.	8.69555	-7.58404	4.17634	0.808086
13.9999	10.5249	-9.89019	3.48017	0.920253
15.0003	11.7916	-11.619	1.77277	0.948014
16.0003	12.5546	-12.5196	-0.435593	0.828565
17.0002	12.4317	-12.1305	-2.65577	0.587616
18.0001	11.5555	-10.7005	-4.34539	0.384044
19.0001	9.86347	-8.48873	-5.01995	0.171907
20.	7.67829	-6.13124	-4.62175	0.058662
20.9999	5.22874	-4.17078	-3.15271	0.068879
22.0003	3.24642	-3.08351	-1.00547	0.142464
23.0002	3.41404	-3.14793	1.27035	0.36391
24.0002	5.42921	-4.39561	3.1439	0.520403
25.0001	7.63052	-6.37659	4.12457	0.742836
26.	9.69595	-8.81027	3.95519	0.864362
27.	11.2587	-10.8778	2.73629	0.971087
28.0004	12.2965	-12.2465	0.686188	0.869882
29.0003	12.5436	-12.4203	-1.599	0.721761
30.0002	12.0959	-11.5342	-3.60891	0.498903
31.0001	10.7917	-9.62765	-4.86608	0.30102
32.0001	8.81938	-7.2943	-4.95588	0.117562
33.	6.45362	-5.06006	-4.00536	0.045026
33.9999	4.11533	-3.52734	-2.11587	0.130085
35.0003	2.97685	-2.96532	0.17094	0.198215
36.0003	4.32643	-3.64854	2.28603	0.424588
37.0002	6.53644	-5.31632	3.75145	0.623189
38.0001	8.72388	-7.61974	4.16487	0.836284
39.	10.5089	-9.87809	3.46407	0.927883
40.	11.8354	-11.6674	1.76938	0.904454
40.9999	12.5325	-12.5013	-0.445592	0.764212
42.0003	12.4037	-12.1026	-2.65549	0.571331
43.0002	11.5622	-10.7082	-4.34176	0.408183
44.0001	9.89489	-8.50773	-5.04542	0.26673
45.0001	7.66902	-6.11115	-4.63284	0.066287
46.	5.21649	-4.17401	-3.12842	0.04928
46.9999	3.29706	-3.14341	-0.982709	0.154431
48.0003	3.38913	-3.12019	1.27875	0.339748
49.0003	5.41347	-4.39179	3.12519	0.501075
50.0002	7.61569	-6.37265	4.1047	0.734491

(0,5,0) Blue offset 13.4A White Offset -29A RMF on

```
Data = Import[NotebookDirectory[] <> "x0_y5_z0_B13_4_W-29_RMFon.txt", "Table"];
        importi··· Notebook-Verzeichnis                                  Tabelle
```

```
Bvst = Table[{AbsoluteTime[Data[[i]][[9]]] - AbsoluteTime[Data[[2]][[9]]],
        Tabelle   absolute Zeit seit 1900                absolute Zeit seit 1900
    AbsoluteTime[Data[[i]][[2]]]}, {i, 2, Length[Data]}];
    absolute Zeit seit 1900                     Länge
Bxvst = Table[{AbsoluteTime[Data[[i]][[9]]] - AbsoluteTime[Data[[2]][[9]]],
         Tabelle   absolute Zeit seit 1900                absolute Zeit seit 1900
    AbsoluteTime[Data[[i]][[3]]]}, {i, 2, Length[Data]}];
    absolute Zeit seit 1900                     Länge
Byvst = Table[{AbsoluteTime[Data[[i]][[9]]] - AbsoluteTime[Data[[2]][[9]]],
         Tabelle   absolute Zeit seit 1900                absolute Zeit seit 1900
    AbsoluteTime[Data[[i]][[4]]]}, {i, 2, Length[Data]}];
    absolute Zeit seit 1900                     Länge
Bzvst = Table[{AbsoluteTime[Data[[i]][[9]]] - AbsoluteTime[Data[[2]][[9]]],
         Tabelle   absolute Zeit seit 1900                absolute Zeit seit 1900
    AbsoluteTime[Data[[i]][[5]]]}, {i, 2, Length[Data]}];
    absolute Zeit seit 1900                     Länge
```

```
ListPlot[{Bvst, Bxvst, Byvst, Bzvst}, Joined → True]
listenbezogene Graphik                verknüpft? wahr
```

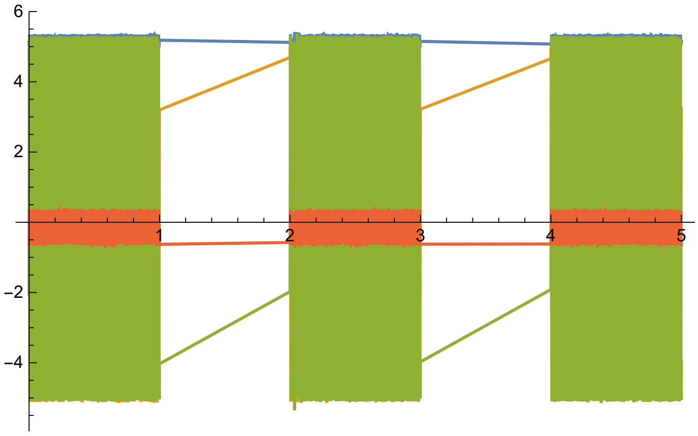


```
Start = 100;
Stop = 1000;
```

```
ListPlot[{Table[{Bvst[[i]][[1]], Bvst[[i]][[2]]}, {i, Start, Stop}],
listenbezo⋯ Tabelle
  Table[{Bxvst[[i]][[1]], Bxvst[[i]][[2]]}, {i, Start, Stop}],
  Tabelle
  Table[{Byvst[[i]][[1]], Byvst[[i]][[2]]}, {i, Start, Stop}],
  Tabelle
  Table[{Bzvst[[i]][[1]], Bzvst[[i]][[2]]}, {i, Start, Stop}]}, Joined → True]
  Tabelle                                                   verknüpft? wahr
```

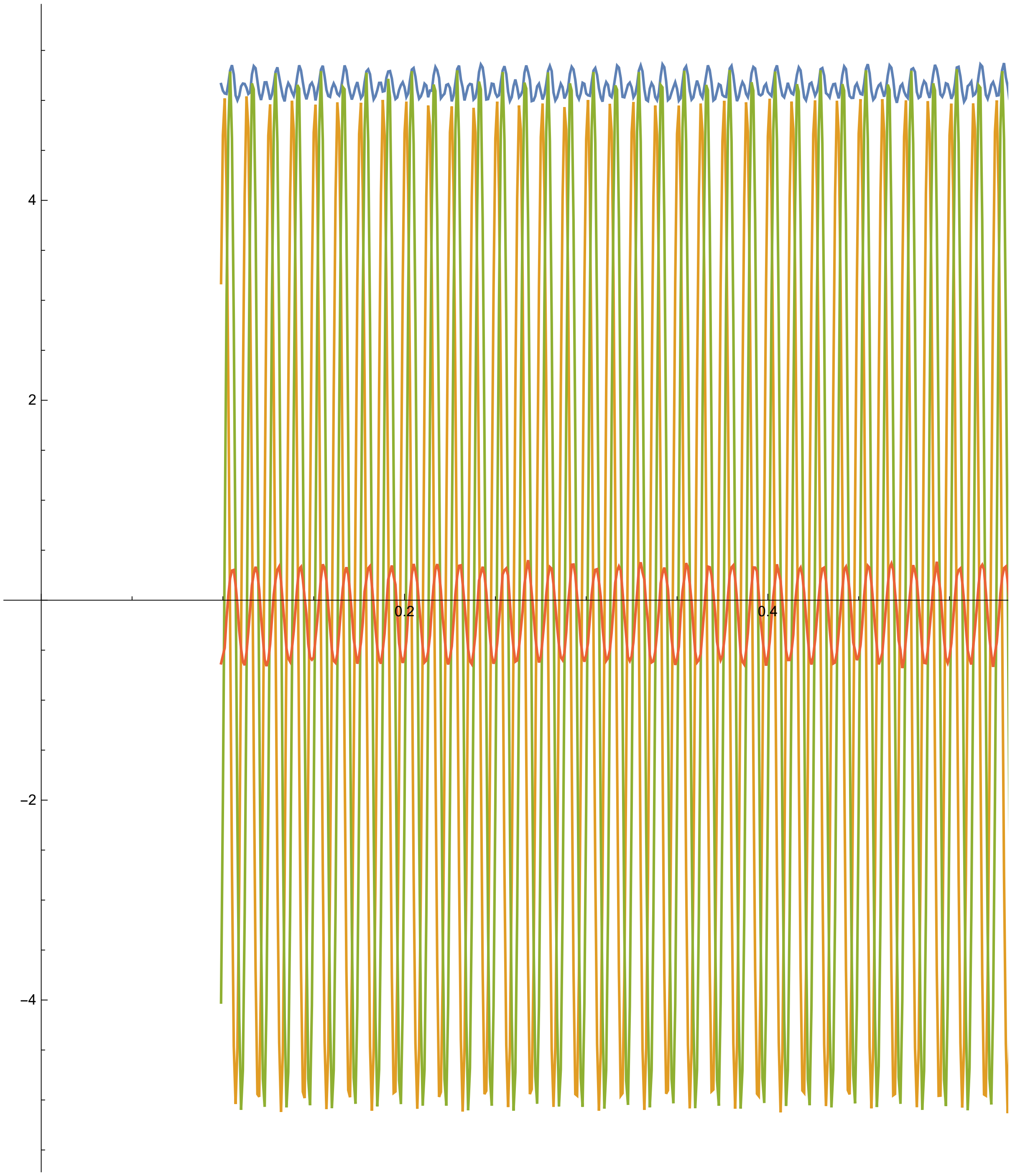

```
ListPlot[{Table[{Bvst[[i]][[1]], Bvst[[i]][[2]]}, {i, Start, Start + 50}],
listenbezo⋯ Tabelle
  Table[{Bxvst[[i]][[1]], Bxvst[[i]][[2]]}, {i, Start, Start + 50}],
  Tabelle
  Table[{Byvst[[i]][[1]], Byvst[[i]][[2]]}, {i, Start, Start + 50}],
  Tabelle
  Table[{Bzvst[[i]][[1]], Bzvst[[i]][[2]]}, {i, Start, Start + 50}]}, Joined → True]
  Tabelle                                                            verknüpft? wahr
```

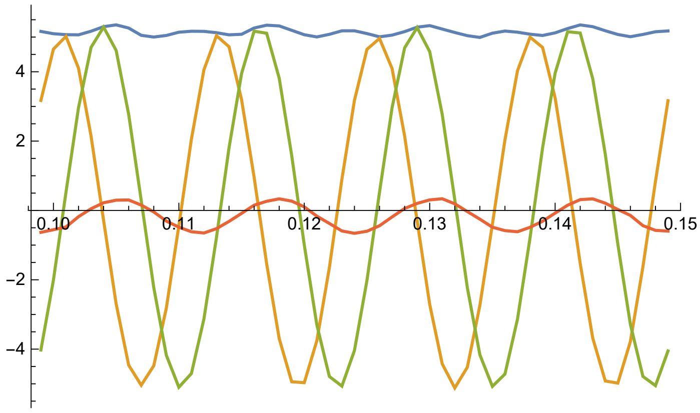


```
TableForm[{Mean[Table[Bvst[[i]][[2]], {i, Start, Stop}]],
Tabellendars⋯ arit⋯ Tabelle
  Mean[Table[Bxvst[[i]][[2]], {i, Start, Stop}]],
  arit⋯ Tabelle
  Mean[Table[Byvst[[i]][[2]], {i, Start, Stop}]],
  arit⋯ Tabelle
  Mean[Table[Bzvst[[i]][[2]], {i, Start, Stop}]]}, TableDirections → Row]
  arit⋯ Tabelle                                   Richtung der Tabellen⋯ Zeile
```

```
5.14463  -0.0314532  0.087873  -0.143724
```

```
TableForm[Table[{(Bvst[[i]][[1]] - Bvst[[Start]][[1]]) * 1000, Bvst[[i]][[2]],
Tabellendar·· Tabelle
   Bxvst[[i]][[2]], Byvst[[i]][[2]], Bzvst[[i]][[2]]}, {i, Start, Start + 50}]]
```

0.	5.16333	3.17245	-4.02456	-0.631257
1.0004	5.09716	4.65191	-2.00796	-0.55571
2.00033	5.06963	5.01973	0.524527	-0.477825
3.00026	5.06465	4.10185	2.96532	-0.180048
4.00019	5.16932	2.14504	4.70302	0.048694
5.00011	5.30041	-0.288006	5.28794	0.221737
6.00004	5.35233	-2.69257	4.61624	0.296286
7.00045	5.26047	-4.46241	2.76911	0.302333
8.00037	5.05063	-5.04002	0.288489	0.154392
9.0003	5.00153	-4.47905	-2.2252	-0.043801
10.0002	5.04656	-2.82035	-4.17372	-0.305694
11.0002	5.14056	-0.44195	-5.09867	-0.483366
12.0001	5.16878	2.05437	-4.70306	-0.614085
13.	5.16496	4.05723	-3.12925	-0.650713
14.0004	5.12626	5.03979	-0.776885	-0.524909
15.0003	5.06458	4.72186	1.8039	-0.316195
16.0003	5.08061	3.19662	3.94805	-0.084696
17.0002	5.2611	0.961473	5.17029	0.151047
18.0001	5.34288	-1.53443	5.11099	0.263893
19.0001	5.3222	-3.69329	3.81754	0.334415
20.	5.1951	-4.94137	1.58099	0.269127
21.0004	5.06789	-4.96803	-0.995803	0.103034
22.0003	5.00403	-3.76098	-3.29676	-0.163584
23.0002	5.07944	-1.65227	-4.78853	-0.375135
24.0002	5.18065	0.89897	-5.06763	-0.591735
25.0001	5.18129	3.17618	-4.04013	-0.659538
26.	5.10099	4.64747	-2.01529	-0.599783
27.0004	5.00758	4.9601	0.527906	-0.441153
28.0004	5.05671	4.09377	2.96243	-0.188011
29.0003	5.15736	2.14914	4.6879	0.056422
30.0002	5.28586	-0.304186	5.27321	0.202676
31.0001	5.33054	-2.70845	4.58111	0.303914
32.0001	5.23152	-4.42605	2.76862	0.336957
33.	5.13249	-5.11967	0.307842	0.191515
34.0004	5.04088	-4.52294	-2.22542	-0.03235
35.0003	4.99012	-2.7408	-4.16247	-0.251334
36.0003	5.11311	-0.397977	-5.07462	-0.483453
37.0002	5.17354	2.03772	-4.71956	-0.58216
38.0001	5.14162	4.02961	-3.13383	-0.614492
39.	5.08292	4.99721	-0.79253	-0.485702
40.0004	5.04569	4.7019	1.80382	-0.31199
41.0004	5.122	3.24768	3.95998	-0.077039
42.0003	5.24611	0.965489	5.15429	0.150762
43.0002	5.35113	-1.52994	5.11826	0.311966
44.0001	5.30022	-3.68127	3.79855	0.334002
45.0001	5.18282	-4.91803	1.62193	0.209501
46.	5.0748	-4.98083	-0.97166	0.027522
47.0004	5.01065	-3.78078	-3.28509	-0.143198
48.0003	5.07603	-1.62892	-4.78754	-0.438371
49.0003	5.15382	0.836122	-5.05295	-0.57486
50.0002	5.17675	3.16485	-4.05312	-0.595608

(0,5,0) Blue offset 13.4A White Offset -29A RMF off

```
Data = Import[NotebookDirectory[] <> "x0_y5_z0_B13_4_W-29_RMFoff.txt", "Table"];
       importi··· Notebook-Verzeichnis                                  Tabelle
```

```
Bvst = Table[{AbsoluteTime[Data[[i]][[9]]] - AbsoluteTime[Data[[2]][[9]]],
        Tabelle  absolute Zeit seit 1900              absolute Zeit seit 1900
    AbsoluteTime[Data[[i]][[2]]]}, {i, 2, Length[Data]}];
    absolute Zeit seit 1900                  Länge
Bxvst = Table[{AbsoluteTime[Data[[i]][[9]]] - AbsoluteTime[Data[[2]][[9]]],
        Tabelle  absolute Zeit seit 1900              absolute Zeit seit 1900
    AbsoluteTime[Data[[i]][[3]]]}, {i, 2, Length[Data]}];
    absolute Zeit seit 1900                  Länge
Byvst = Table[{AbsoluteTime[Data[[i]][[9]]] - AbsoluteTime[Data[[2]][[9]]],
        Tabelle  absolute Zeit seit 1900              absolute Zeit seit 1900
    AbsoluteTime[Data[[i]][[4]]]}, {i, 2, Length[Data]}];
    absolute Zeit seit 1900                  Länge
Bzvst = Table[{AbsoluteTime[Data[[i]][[9]]] - AbsoluteTime[Data[[2]][[9]]],
        Tabelle  absolute Zeit seit 1900              absolute Zeit seit 1900
    AbsoluteTime[Data[[i]][[5]]]}, {i, 2, Length[Data]}];
    absolute Zeit seit 1900                  Länge
```

```
ListPlot[{Bvst, Bxvst, Byvst, Bzvst}, Joined → True]
listenbezogene Graphik                verknüpft? wahr
```

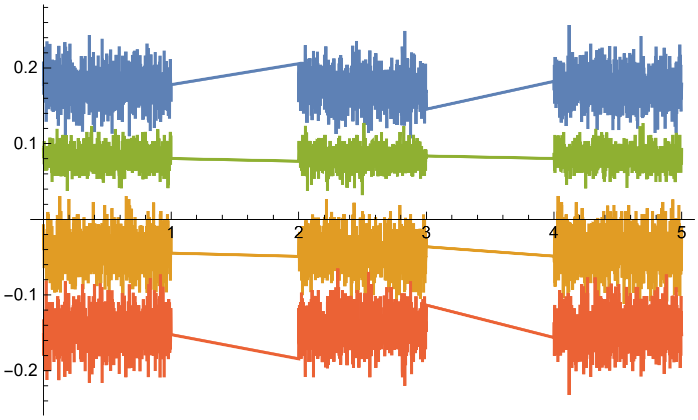


```
Start = 100;
Stop = 1000;
```

```
ListPlot[{Table[{Bvst[[i]][[1]], Bvst[[i]][[2]]}, {i, Start, Stop}],
  Table[{Bxvst[[i]][[1]], Bxvst[[i]][[2]]}, {i, Start, Stop}],
  Table[{Byvst[[i]][[1]], Byvst[[i]][[2]]}, {i, Start, Stop}],
  Table[{Bzvst[[i]][[1]], Bzvst[[i]][[2]]}, {i, Start, Stop}]}, Joined → True]
```

listenbezo··· Tabelle Tabelle Tabelle Tabelle verknüpft? wahr

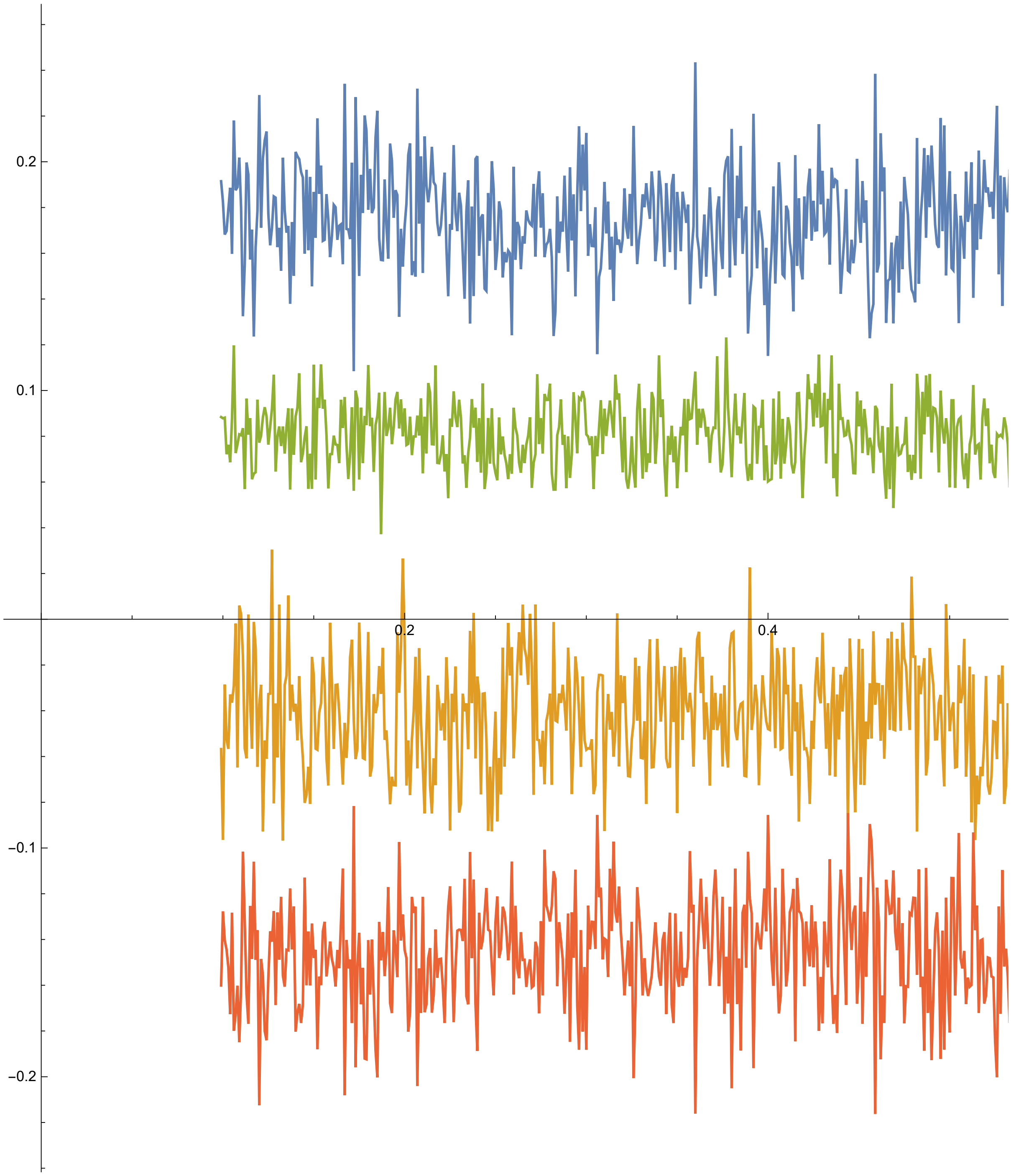

```
ListPlot[{Table[{Bvst[[i]][[1]], Bvst[[i]][[2]]}, {i, Start, Start + 50}],
  Table[{Bxvst[[i]][[1]], Bxvst[[i]][[2]]}, {i, Start, Start + 50}],
  Table[{Byvst[[i]][[1]], Byvst[[i]][[2]]}, {i, Start, Start + 50}],
  Table[{Bzvst[[i]][[1]], Bzvst[[i]][[2]]}, {i, Start, Start + 50}]}, Joined → True]
```

listenbezo··· Tabelle Tabelle Tabelle Tabelle verknüpft? wahr

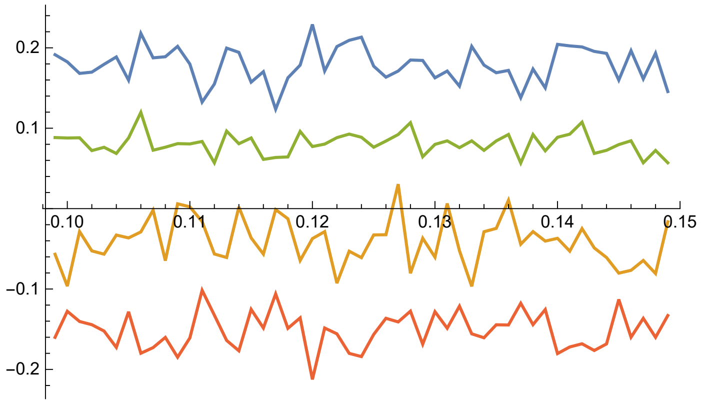


```
TableForm[{Mean[Table[Bvst[[i]][[2]], {i, Start, Stop}]],
  Mean[Table[Bxvst[[i]][[2]], {i, Start, Stop}]],
  Mean[Table[Byvst[[i]][[2]], {i, Start, Stop}]],
  Mean[Table[Bzvst[[i]][[2]], {i, Start, Stop}]]}, TableDirections → Row]
```

Tabellendars··· arit··· Tabelle arit··· Tabelle arit··· Tabelle arit··· Tabelle Richtung der Tabellen··· Zeile

0.1745	-0.0410572	0.0815737	-0.145821

```
TableForm[Table[{(Bvst[[i]][[1]] - Bvst[[Start]][[1]]) * 1000, Bvst[[i]][[2]],
Tabellendar·· Tabelle
   Bxvst[[i]][[2]], Byvst[[i]][[2]], Bzvst[[i]][[2]]}, {i, Start, Start + 50}]]
```

0.	0.191481	-0.056756	0.088398	-0.160092
0.999928	0.18254	-0.096424	0.087862	-0.127686
1.99986	0.168193	-0.028551	0.088126	-0.140384
2.99978	0.169756	-0.052575	0.07216	-0.144381
3.99971	0.179491	-0.056661	0.076277	-0.152277
5.00011	0.188661	-0.03286	0.068612	-0.172643
6.00004	0.159753	-0.036427	0.087933	-0.128304
6.99997	0.218041	-0.028995	0.119733	-0.179904
7.9999	0.187553	-0.001866	0.072648	-0.172901
8.99982	0.188915	-0.064743	0.07639	-0.160193
9.99975	0.201851	0.006002	0.08084	-0.184859
10.9997	0.179844	0.002249	0.080469	-0.160822
12.0001	0.132507	-0.016145	0.083545	-0.101575
13.	0.154939	-0.056435	0.056974	-0.132571
13.9999	0.199682	-0.060805	0.096453	-0.163928
14.9999	0.194381	0.002084	0.080713	-0.176819
15.9998	0.157321	-0.036396	0.087887	-0.125304
16.9997	0.170311	-0.056604	0.061218	-0.148507
18.0001	0.123665	-0.001167	0.063626	-0.106035
19.0001	0.162681	-0.01261	0.06427	-0.148914
20.	0.178463	-0.064516	0.096021	-0.135892
20.9999	0.229122	-0.037281	0.077217	-0.212473
21.9998	0.17121	-0.028625	0.080249	-0.148505
22.9998	0.201596	-0.092713	0.088294	-0.155723
23.9997	0.209344	-0.052966	0.092708	-0.180069
25.0001	0.213244	-0.061003	0.08876	-0.184047
26.	0.177203	-0.032703	0.076367	-0.156524
27.	0.16349	-0.032505	0.08406	-0.136405
27.9999	0.171137	0.030442	0.092195	-0.14093
28.9998	0.18484	-0.080444	0.106879	-0.127561
29.9997	0.184307	-0.036815	0.064547	-0.168664
31.0001	0.162681	-0.060417	0.079906	-0.128179
32.0001	0.171137	0.006369	0.08429	-0.148804
33.	0.152335	-0.052342	0.075809	-0.121324
33.9999	0.201775	-0.096708	0.08429	-0.155743
34.9998	0.17853	-0.028741	0.072433	-0.160625
35.9998	0.169024	-0.024588	0.084192	-0.144486
36.9997	0.17193	0.010402	0.092233	-0.144724
38.0001	0.137967	-0.044281	0.056759	-0.117697
39.	0.173623	-0.028596	0.092186	-0.144322
40.	0.150167	-0.040379	0.071884	-0.125508
40.9999	0.204316	-0.036963	0.088727	-0.180295
41.9998	0.202428	-0.052884	0.092585	-0.17207
42.9997	0.201132	-0.024859	0.107556	-0.16813
44.0001	0.195574	-0.048901	0.068654	-0.176478
45.0001	0.193083	-0.060822	0.072517	-0.168294
46.	0.159844	-0.080262	0.079653	-0.112976
46.9999	0.196451	-0.076751	0.084375	-0.159948
47.9999	0.161363	-0.064476	0.057026	-0.136488
48.9998	0.193352	-0.080738	0.072371	-0.16009
49.9997	0.145623	-0.016437	0.057022	-0.132983
