# Supplementary material for: Spatially selective delivery of living magnetic microrobots through torque-focusing
Source: Nat Commun. 2024 Mar 9;15:2160. doi: 10.1038/s41467-024-46407-4 (PMC10924878; doi:10.1038/s41467-024-46407-4)
Supplement: Supplementary file 4 — Source Data [file 41467_2024_46407_MOESM4_ESM.zip › Data_Sorted_by_Figures/Fig_4/4I/Field Characterization/y axis measurements/x0_y-5_z0/ReadingHallProbeData.pdf]

```
NotebookDirectory[]
```
Notebook–Verzeichnis

```
P:\Michael\MetroLabHallProbe\230224\x0_y-5_z0\
```

(0,-5,0) Blue offset 0A White Offset 0A RMF off

```
Data = Import[NotebookDirectory[] <> "x0_y-5_z0_B0_W0_RMFoff.txt", "Table"];
```
importi··· Notebook–Verzeichnis Tabelle

```
Bvst = Table[{AbsoluteTime[Data[[i]][[9]]] - AbsoluteTime[Data[[2]][[9]]],
    AbsoluteTime[Data[[i]][[2]]]}, {i, 2, Length[Data]}];
Bxvst = Table[{AbsoluteTime[Data[[i]][[9]]] - AbsoluteTime[Data[[2]][[9]]],
    AbsoluteTime[Data[[i]][[3]]]}, {i, 2, Length[Data]}];
Byvst = Table[{AbsoluteTime[Data[[i]][[9]]] - AbsoluteTime[Data[[2]][[9]]],
    AbsoluteTime[Data[[i]][[4]]]}, {i, 2, Length[Data]}];
Bzvst = Table[{AbsoluteTime[Data[[i]][[9]]] - AbsoluteTime[Data[[2]][[9]]],
    AbsoluteTime[Data[[i]][[5]]]}, {i, 2, Length[Data]}];
```
Tabelle absolute Zeit seit 1900 absolute Zeit seit 1900 absolute Zeit seit 1900 Länge (×4)

```
ListPlot[{Bvst, Bxvst, Byvst, Bzvst}, Joined → True]
```
listenbezogene Graphik verknüpft? wahr

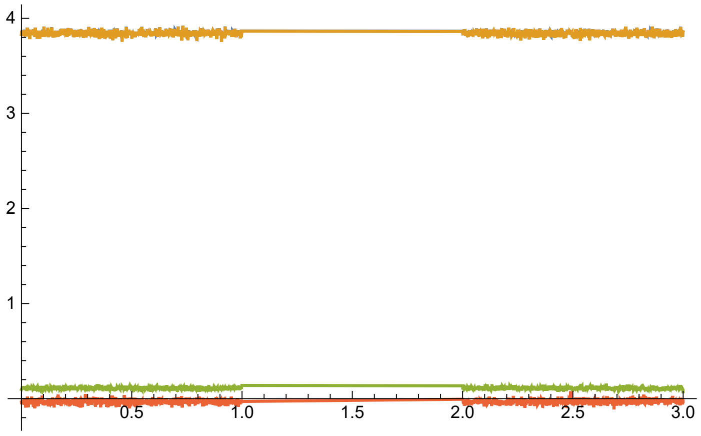


```
Start = 100;
Stop = 1000;
```

```
ListPlot[{Table[{Bvst[[i]][[1]], Bvst[[i]][[2]]}, {i, Start, Stop}],
listenbezo⋯  Tabelle
  Table[{Bxvst[[i]][[1]], Bxvst[[i]][[2]]}, {i, Start, Stop}],
  Tabelle
  Table[{Byvst[[i]][[1]], Byvst[[i]][[2]]}, {i, Start, Stop}],
  Tabelle
  Table[{Bzvst[[i]][[1]], Bzvst[[i]][[2]]}, {i, Start, Stop}]}, Joined → True]
  Tabelle                                                       verknüpft? wahr
```

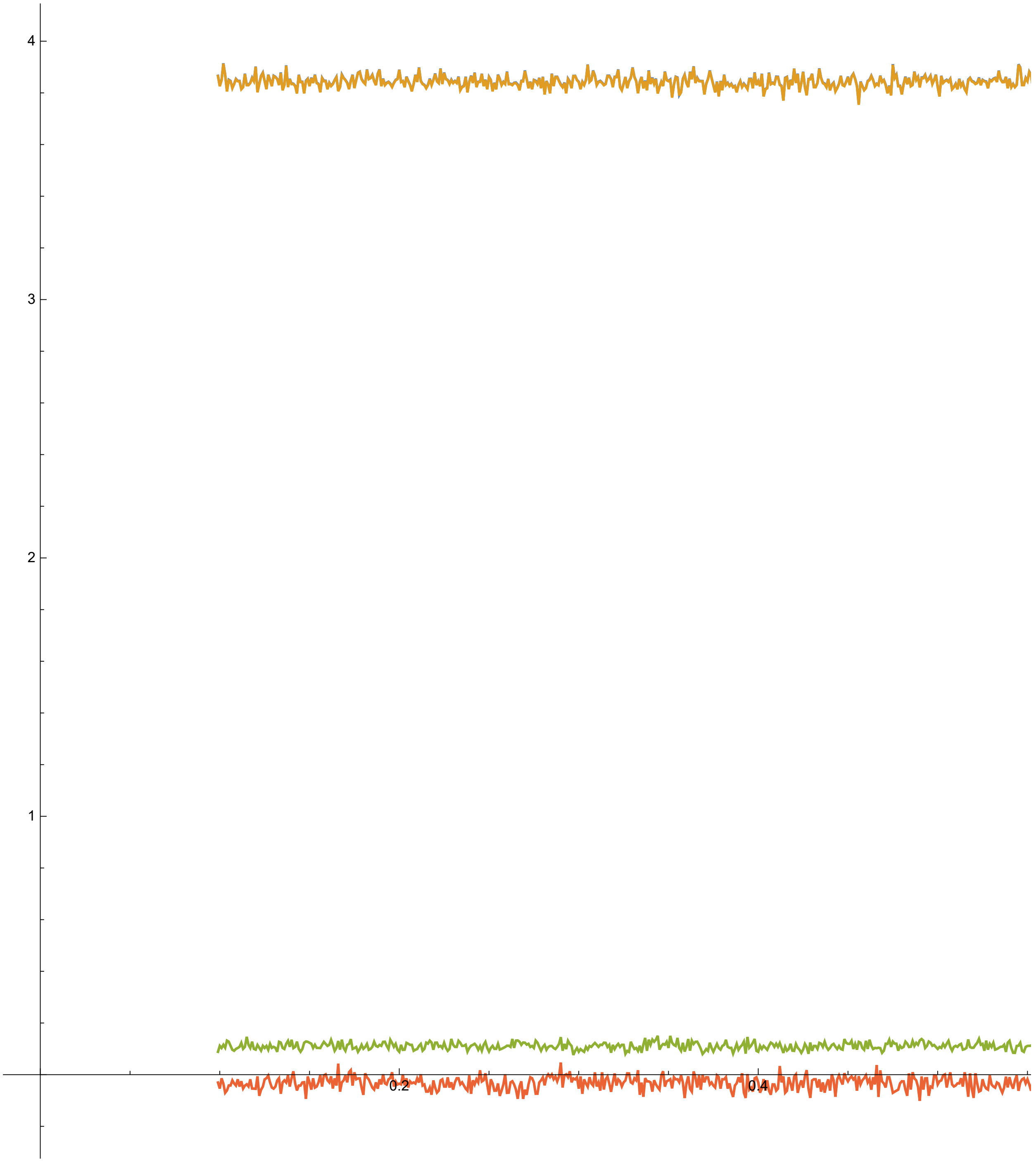

```
ListPlot[{Table[{Bvst[[i]][[1]], Bvst[[i]][[2]]}, {i, Start, Start + 50}],
  Table[{Bxvst[[i]][[1]], Bxvst[[i]][[2]]}, {i, Start, Start + 50}],
  Table[{Byvst[[i]][[1]], Byvst[[i]][[2]]}, {i, Start, Start + 50}],
  Table[{Bzvst[[i]][[1]], Bzvst[[i]][[2]]}, {i, Start, Start + 50}]}, Joined → True]
```

listenbezo⋯ Tabelle Tabelle Tabelle Tabelle verknüpft? wahr

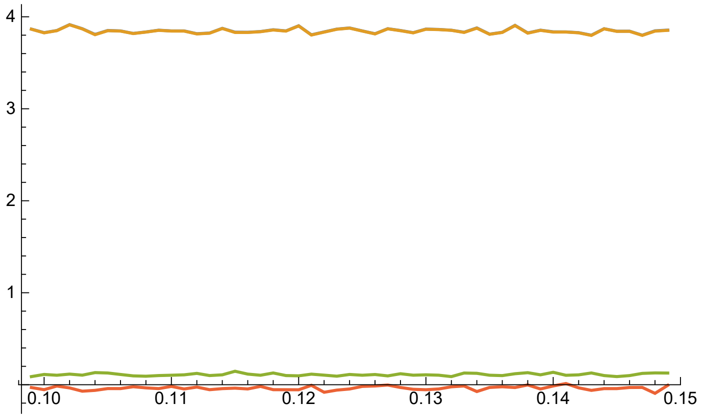


```
TableForm[{Mean[Table[Bvst[[i]][[2]], {i, Start, Stop}]],
  Mean[Table[Bxvst[[i]][[2]], {i, Start, Stop}]],
  Mean[Table[Byvst[[i]][[2]], {i, Start, Stop}]],
  Mean[Table[Bzvst[[i]][[2]], {i, Start, Stop}]]}, TableDirections → Row]
```

Tabellendars⋯ arit⋯ Tabelle arit⋯ Tabelle arit⋯ Tabelle arit⋯ Tabelle Richtung der Tabellen⋯ Zeile

3.84453 3.84273 0.109838 -0.0316375

```
TableForm[Table[{(Bvst[[i]][[1]] - Bvst[[Start]][[1]]) * 1000, Bvst[[i]][[2]],
Tabellendar·· Tabelle
   Bxvst[[i]][[2]], Byvst[[i]][[2]], Bzvst[[i]][[2]]}, {i, Start, Start + 50}]]
```

0.	3.86592	3.86479	0.088453	-0.0305
0.999928	3.82753	3.82552	0.111771	-0.053743
2.00033	3.85035	3.84894	0.103188	-0.014109
3.00026	3.91458	3.91272	0.115568	-0.034581
4.00019	3.8704	3.86836	0.104067	-0.070305
5.00011	3.80819	3.80542	0.131867	-0.06123
6.00004	3.85097	3.84863	0.127613	-0.041737
6.99997	3.84649	3.84464	0.11161	-0.04194
8.00037	3.81914	3.81787	0.096274	-0.021895
9.0003	3.83501	3.83375	0.092477	-0.034119
10.0002	3.85417	3.85265	0.099621	-0.042206
11.0002	3.84633	3.8449	0.103245	-0.018067
12.0001	3.84639	3.84461	0.107672	-0.046001
13.	3.81588	3.8138	0.123328	-0.025441
13.9999	3.82321	3.82153	0.099767	-0.053885
15.0003	3.87437	3.87265	0.107644	-0.04229
16.0003	3.83263	3.82965	0.146527	-0.037252
17.0002	3.83162	3.8296	0.115653	-0.045724
18.0001	3.83933	3.8379	0.103236	-0.017995
19.0001	3.85899	3.8565	0.127806	-0.053817
20.	3.84621	3.84453	0.099795	-0.054122
21.0004	3.90212	3.90053	0.096861	-0.054744
22.0003	3.80375	3.802	0.115024	-0.006443
23.0002	3.83553	3.83324	0.104209	-0.081943
24.0002	3.86605	3.8645	0.09288	-0.058434
25.0001	3.87848	3.8766	0.111709	-0.046269
26.	3.84633	3.8449	0.103245	-0.018067
27.	3.81558	3.81393	0.111146	-0.013626
28.0004	3.87025	3.86906	0.096045	-0.003423
29.0003	3.85073	3.84876	0.119431	-0.029861
30.0002	3.8273	3.82557	0.103711	-0.049866
31.0001	3.86641	3.86452	0.107817	-0.054205
32.0001	3.86228	3.86061	0.103691	-0.046227
33.	3.85395	3.85287	0.088317	-0.022378
33.9999	3.83205	3.82992	0.127163	-0.013546
35.0003	3.87899	3.8763	0.124135	-0.074081
36.0003	3.81129	3.80978	0.103386	-0.029704
37.0002	3.83121	3.82986	0.099288	-0.021973
38.0001	3.9067	3.90476	0.119497	-0.030438
39.	3.82427	3.82203	0.130985	-0.002405
40.	3.85439	3.85261	0.107681	-0.046083
41.0004	3.83631	3.83391	0.135167	-0.013465
42.0003	3.83562	3.83422	0.102758	0.013041
43.0002	3.82739	3.82573	0.107466	-0.033807
44.0001	3.80007	3.79742	0.127858	-0.061209
45.0001	3.87017	3.86865	0.09964	-0.042371
46.	3.84292	3.84167	0.088609	-0.042261
46.9999	3.84318	3.84178	0.099425	-0.030095
48.0003	3.79987	3.79776	0.12337	-0.029275
49.0003	3.84737	3.84409	0.128402	-0.093687
50.0002	3.85509	3.85299	0.127083	-0.006784

(0,-5,0) Blue offset 0A White Offset 0A RMF on

```
Data = Import[NotebookDirectory[] <> "x0_y-5_z0_B0_W0_RMFon.txt", "Table"];
       importi··· Notebook-Verzeichnis                              Tabelle

Bvst = Table[{AbsoluteTime[Data[[i]][[9]]] - AbsoluteTime[Data[[2]][[9]]],
       Tabelle absolute Zeit seit 1900           absolute Zeit seit 1900
    AbsoluteTime[Data[[i]][[2]]]}, {i, 2, Length[Data]}];
    absolute Zeit seit 1900                  Länge
Bxvst = Table[{AbsoluteTime[Data[[i]][[9]]] - AbsoluteTime[Data[[2]][[9]]],
        Tabelle absolute Zeit seit 1900           absolute Zeit seit 1900
    AbsoluteTime[Data[[i]][[3]]]}, {i, 2, Length[Data]}];
    absolute Zeit seit 1900                  Länge
Byvst = Table[{AbsoluteTime[Data[[i]][[9]]] - AbsoluteTime[Data[[2]][[9]]],
        Tabelle absolute Zeit seit 1900           absolute Zeit seit 1900
    AbsoluteTime[Data[[i]][[4]]]}, {i, 2, Length[Data]}];
    absolute Zeit seit 1900                  Länge
Bzvst = Table[{AbsoluteTime[Data[[i]][[9]]] - AbsoluteTime[Data[[2]][[9]]],
        Tabelle absolute Zeit seit 1900           absolute Zeit seit 1900
    AbsoluteTime[Data[[i]][[5]]]}, {i, 2, Length[Data]}];
    absolute Zeit seit 1900                  Länge

ListPlot[{Bvst, Bxvst, Byvst, Bzvst}, Joined → True]
listenbezogene Graphik                   verknüpft? wahr
```

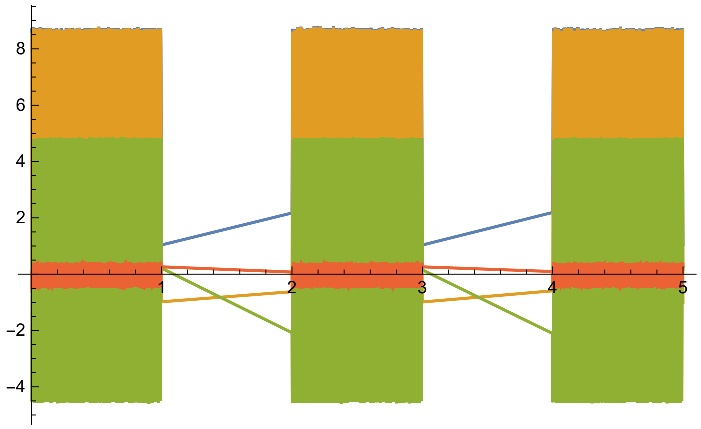


```
Start = 100;
Stop = 1000;
```

```
ListPlot[{Table[{Bvst[[i]][[1]], Bvst[[i]][[2]]}, {i, Start, Stop}],
  Table[{Bxvst[[i]][[1]], Bxvst[[i]][[2]]}, {i, Start, Stop}],
  Table[{Byvst[[i]][[1]], Byvst[[i]][[2]]}, {i, Start, Stop}],
  Table[{Bzvst[[i]][[1]], Bzvst[[i]][[2]]}, {i, Start, Stop}]}, Joined → True]
```

listenbezo… Tabelle Tabelle Tabelle Tabelle verknüpft? wahr

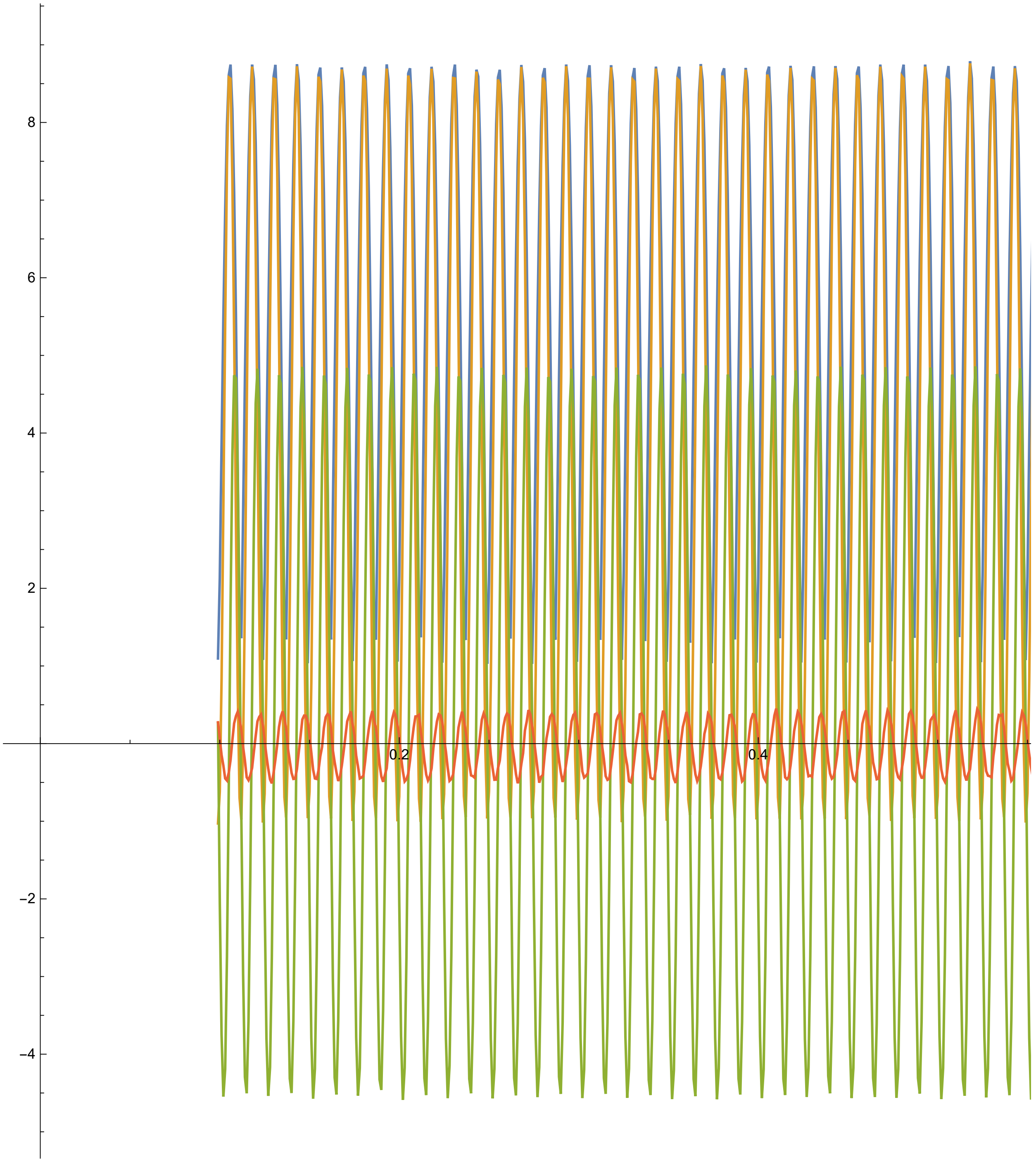

```
ListPlot[{Table[{Bvst[[i]][[1]], Bvst[[i]][[2]]}, {i, Start, Start + 50}],
  Table[{Bxvst[[i]][[1]], Bxvst[[i]][[2]]}, {i, Start, Start + 50}],
  Table[{Byvst[[i]][[1]], Byvst[[i]][[2]]}, {i, Start, Start + 50}],
  Table[{Bzvst[[i]][[1]], Bzvst[[i]][[2]]}, {i, Start, Start + 50}]}, Joined → True]
```

listenbezo··· Tabelle
Tabelle
Tabelle
Tabelle verknüpft? wahr

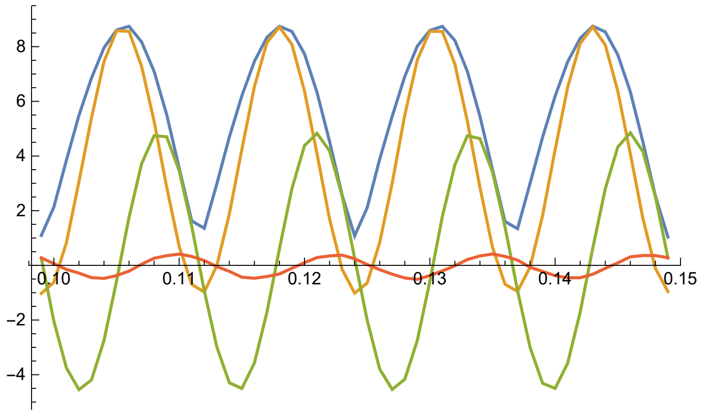


```
TableForm[{Mean[Table[Bvst[[i]][[2]], {i, Start, Stop}]],
  Mean[Table[Bxvst[[i]][[2]], {i, Start, Stop}]],
  Mean[Table[Byvst[[i]][[2]], {i, Start, Stop}]],
  Mean[Table[Bzvst[[i]][[2]], {i, Start, Stop}]]}, TableDirections → Row]
```

Tabellendars··· arit··· Tabelle
arit··· Tabelle
arit··· Tabelle
arit··· Tabelle Richtung der Tabellen··· Zeile

5.59842 3.84242 0.134307 −0.037275

```
TableForm[Table[{(Bvst[[i]][[1]] - Bvst[[Start]][[1]]) * 1000, Bvst[[i]][[2]],
Tabellendar·· Tabelle
   Bxvst[[i]][[2]], Byvst[[i]][[2]], Bzvst[[i]][[2]]}, {i, Start, Start + 50}]]
```

0.	1.09679	-1.02855	0.264791	0.273693
0.999928	2.13358	-0.618961	-2.04084	0.063235
1.99986	3.84229	0.826971	-3.74933	-0.147791
3.00026	5.48598	3.05765	-4.54606	-0.282976
4.00019	6.84809	5.39565	-4.19308	-0.448719
5.00011	7.95856	7.45376	-2.74838	-0.475886
6.00004	8.60771	8.58029	-0.575519	-0.37431
6.99997	8.7456	8.56319	1.76425	-0.211386
7.9999	8.17369	7.28141	3.71329	0.041628
9.0003	7.0943	5.26834	4.74399	0.261155
10.0002	5.52069	2.86621	4.7051	0.353342
11.0002	3.55994	0.696163	3.46683	0.41182
12.0001	1.62663	-0.695361	1.43344	0.328109
13.	1.35681	-0.976143	-0.925801	0.17596
13.9999	2.97406	-0.014928	-2.97369	-0.044253
14.9999	4.69082	1.87494	-4.29423	-0.218927
16.0003	6.20067	4.24011	-4.50354	-0.433543
17.0002	7.4585	6.52974	-3.5735	-0.470956
18.0001	8.34183	8.15229	-1.72059	-0.407385
19.0001	8.74534	8.71851	0.60785	-0.314659
20.	8.55483	8.07803	2.81422	-0.103336
20.9999	7.73574	6.37516	4.38057	0.098168
21.9998	6.32622	4.07744	4.82838	0.286726
23.0002	4.53179	1.69668	4.18796	0.345503
24.0002	2.57501	-0.144161	2.54342	0.37538
25.0001	1.07833	-1.01688	0.265294	0.241575
26.	2.10752	-0.647326	-2.00539	0.032063
27.	3.88617	0.834933	-3.79219	-0.156529
27.9999	5.45696	3.01423	-4.53751	-0.322404
29.0003	6.90921	5.49049	-4.16879	-0.461328
30.0002	8.01687	7.5124	-2.75178	-0.511549
31.0001	8.59287	8.56431	-0.591537	-0.37439
32.0001	8.74227	8.55525	1.78711	-0.202953
33.	8.22784	7.35988	3.67823	-0.014726
33.9999	7.08987	5.26386	4.7447	0.214204
34.9998	5.45491	2.8552	4.63522	0.344387
36.0003	3.50777	0.661255	3.41973	0.415462
37.0002	1.60459	-0.691337	1.41045	0.327716
38.0001	1.34184	-0.951979	-0.926017	0.19171
39.	3.00181	-0.063146	-3.00038	-0.068167
40.	4.69039	1.82692	-4.31422	-0.222738
40.9999	6.19238	4.23668	-4.5004	-0.37745
42.0003	7.44919	6.51791	-3.57776	-0.454896
43.0002	8.30584	8.11582	-1.70796	-0.450824
44.0001	8.75073	8.72238	0.624021	-0.325454
45.0001	8.54056	8.06697	2.80232	-0.110405
46.	7.70365	6.37117	4.32964	0.093431
46.9999	6.34994	4.08567	4.85102	0.310991
47.9999	4.52461	1.72386	4.16775	0.360917
49.0003	2.54064	-0.112296	2.5127	0.35858
50.0002	1.03621	-0.960478	0.268749	0.281053

(0,-5,0) Blue offset -12.8A White Offset 24.7A RMF off

```
Data = Import[NotebookDirectory[] <> "x0_y-5_z0_B-12_8_W24_7.txt", "Table"];
        importi··· Notebook–Verzeichnis                                        Tabelle

Bvst = Table[{AbsoluteTime[Data[[i]][[9]]] - AbsoluteTime[Data[[2]][[9]]],
        Tabelle  absolute Zeit seit 1900               absolute Zeit seit 1900
    AbsoluteTime[Data[[i]][[2]]]}, {i, 2, Length[Data]}];
    absolute Zeit seit 1900                        Länge
Bxvst = Table[{AbsoluteTime[Data[[i]][[9]]] - AbsoluteTime[Data[[2]][[9]]],
         Tabelle  absolute Zeit seit 1900               absolute Zeit seit 1900
    AbsoluteTime[Data[[i]][[3]]]}, {i, 2, Length[Data]}];
    absolute Zeit seit 1900                        Länge
Byvst = Table[{AbsoluteTime[Data[[i]][[9]]] - AbsoluteTime[Data[[2]][[9]]],
         Tabelle  absolute Zeit seit 1900               absolute Zeit seit 1900
    AbsoluteTime[Data[[i]][[4]]]}, {i, 2, Length[Data]}];
    absolute Zeit seit 1900                        Länge
Bzvst = Table[{AbsoluteTime[Data[[i]][[9]]] - AbsoluteTime[Data[[2]][[9]]],
         Tabelle  absolute Zeit seit 1900               absolute Zeit seit 1900
    AbsoluteTime[Data[[i]][[5]]]}, {i, 2, Length[Data]}];
    absolute Zeit seit 1900                        Länge
```

```
ListPlot[{Bvst, Bxvst, Byvst, Bzvst}, Joined → True]
listenbezogene Graphik                   verknüpft? wahr
```

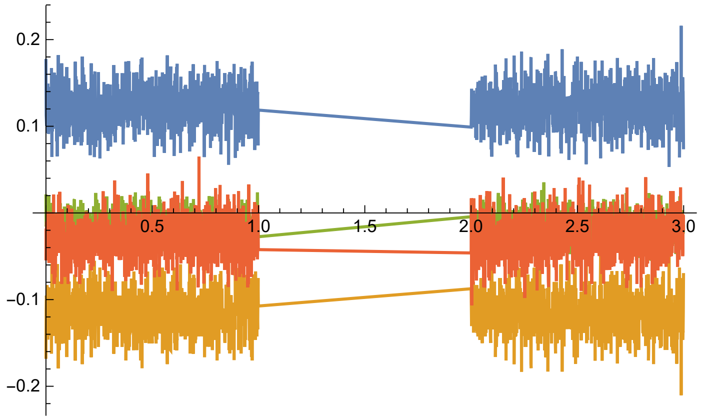


```
Start = 100;
Stop = 1000;
```

```
ListPlot[{Table[{Bvst[[i]][[1]], Bvst[[i]][[2]]}, {i, Start, Stop}],
  Table[{Bxvst[[i]][[1]], Bxvst[[i]][[2]]}, {i, Start, Stop}],
  Table[{Byvst[[i]][[1]], Byvst[[i]][[2]]}, {i, Start, Stop}],
  Table[{Bzvst[[i]][[1]], Bzvst[[i]][[2]]}, {i, Start, Stop}]}, Joined → True]
```

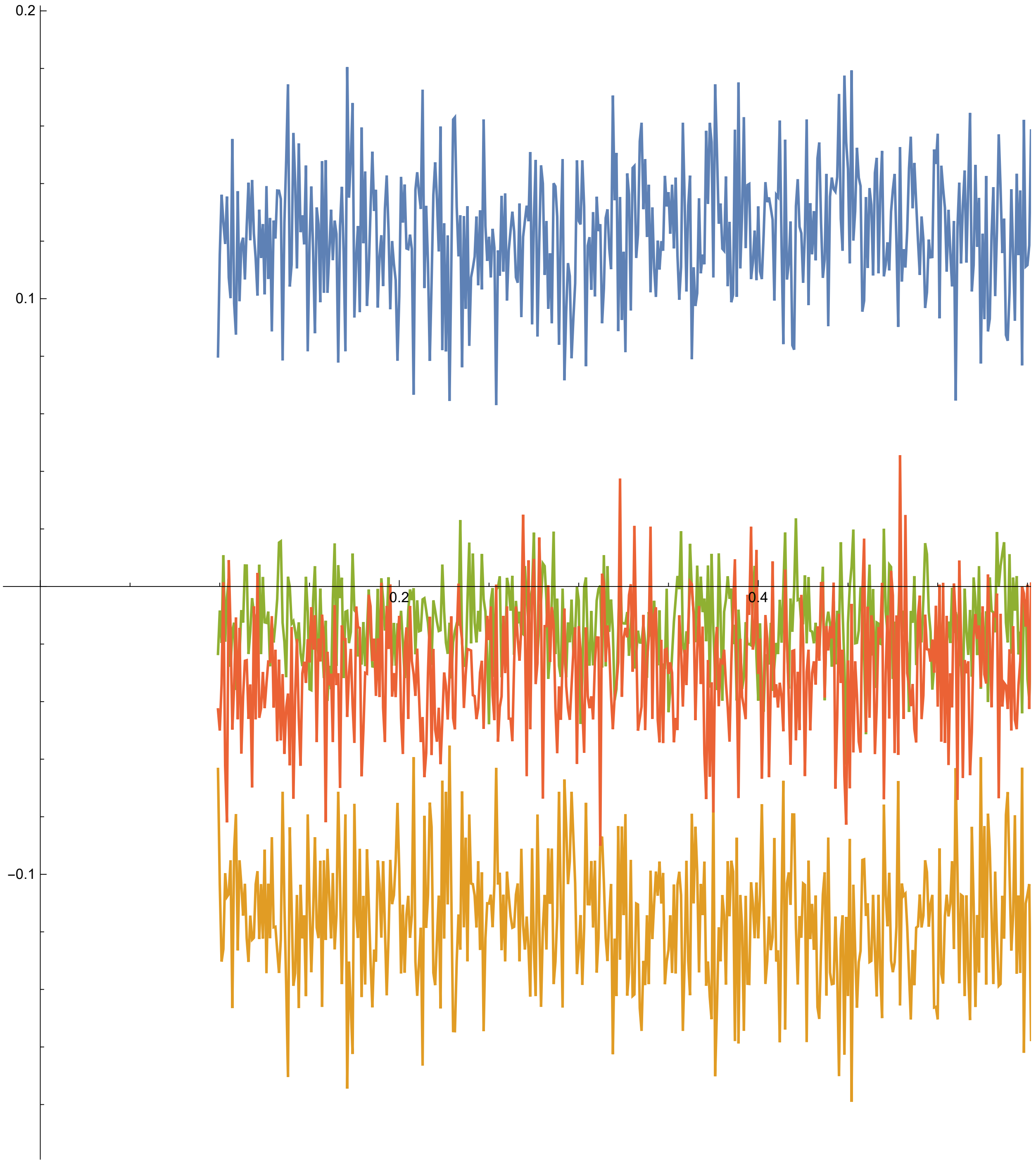

```
ListPlot[{Table[{Bvst[[i]][[1]], Bvst[[i]][[2]]}, {i, Start, Start + 50}],
  Table[{Bxvst[[i]][[1]], Bxvst[[i]][[2]]}, {i, Start, Start + 50}],
  Table[{Byvst[[i]][[1]], Byvst[[i]][[2]]}, {i, Start, Start + 50}],
  Table[{Bzvst[[i]][[1]], Bzvst[[i]][[2]]}, {i, Start, Start + 50}]}, Joined → True]
```

listenbezo··· Tabelle Tabelle Tabelle Tabelle verknüpft? wahr

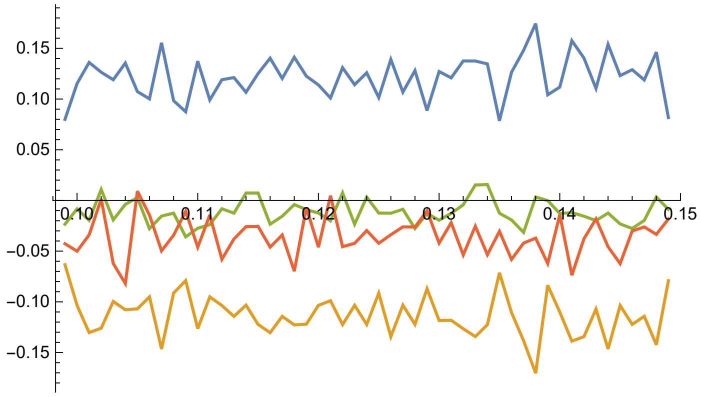


```
TableForm[{Mean[Table[Bvst[[i]][[2]], {i, Start, Stop}]],
  Mean[Table[Bxvst[[i]][[2]], {i, Start, Stop}]],
  Mean[Table[Byvst[[i]][[2]], {i, Start, Stop}]],
  Mean[Table[Bzvst[[i]][[2]], {i, Start, Stop}]]}, TableDirections → Row]
```

Tabellendars··· arit··· Tabelle arit··· Tabelle arit··· Tabelle arit··· Tabelle Richtung der Tabellen··· Zeile

0.122534 −0.115488 −0.0139197 −0.0278124

```
TableForm[Table[{(Bvst[[i]][[1]] - Bvst[[Start]][[1]]) * 1000, Bvst[[i]][[2]],
Tabellendar·· Tabelle
  Bxvst[[i]][[2]], Byvst[[i]][[2]], Bzvst[[i]][[2]]}, {i, Start, Start + 50}]]
```

0.	0.079962	-0.063415	-0.023415	-0.042711
0.999928	0.115287	-0.103511	-0.008343	-0.050069
2.00033	0.136107	-0.130333	-0.019618	-0.033962
3.00026	0.126479	-0.126005	0.010849	0.001465
4.00019	0.119038	-0.099624	-0.019153	-0.062276
5.00011	0.13551	-0.107845	-0.003859	-0.081961
6.00004	0.107341	-0.106915	0.002751	0.009146
6.99997	0.10017	-0.095121	-0.02788	-0.014447
7.9999	0.155477	-0.146501	-0.015393	-0.049733
9.0003	0.098362	-0.091343	-0.012572	-0.034256
10.0002	0.08751	-0.079072	-0.035922	-0.010735
11.0002	0.137364	-0.126449	-0.027429	-0.046123
12.0001	0.099126	-0.095125	-0.023881	-0.014386
13.	0.119042	-0.103593	-0.00822	-0.058067
13.9999	0.121186	-0.114383	-0.012538	-0.038019
15.0003	0.10671	-0.10328	0.007289	-0.025828
16.0003	0.125148	-0.122279	0.007267	-0.025633
17.0002	0.140303	-0.130453	-0.023434	-0.046021
18.0001	0.12032	-0.114338	-0.015599	-0.034065
19.0001	0.141244	-0.12272	-0.00406	-0.069809
20.	0.122593	-0.122067	-0.009022	-0.00688
20.9999	0.113961	-0.103466	-0.012403	-0.04613
22.0003	0.101079	-0.098933	-0.020176	0.004713
23.0002	0.130927	-0.122485	0.007572	-0.045629
24.0002	0.114167	-0.103413	-0.023463	-0.042299
25.0001	0.125913	-0.122316	0.003328	-0.029693
26.	0.101484	-0.091425	-0.01245	-0.042255
27.	0.139104	-0.13434	-0.012623	-0.033813
27.9999	0.10686	-0.103264	-0.008709	-0.026073
29.0003	0.128051	-0.122243	-0.027729	-0.026167
30.0002	0.088651	-0.087095	-0.012934	-0.010301
31.0001	0.127173	-0.118416	-0.019481	-0.042084
32.0001	0.120921	-0.118217	-0.012787	-0.02198
33.	0.137572	-0.126555	-0.004309	-0.05377
33.9999	0.137514	-0.134287	0.015251	-0.025387
35.0003	0.134663	-0.122576	0.015693	-0.053506
36.0003	0.078556	-0.071302	-0.012609	-0.030463
37.0002	0.126416	-0.110582	-0.019227	-0.058163
38.0001	0.148044	-0.138403	-0.031503	-0.042061
39.	0.174442	-0.170396	0.003393	-0.037198
40.	0.104207	-0.083644	-0.000137	-0.06215
40.9999	0.111777	-0.110135	-0.0129	-0.014064
42.0003	0.157601	-0.138752	-0.012017	-0.073765
43.0002	0.140474	-0.134378	-0.015562	-0.037859
44.0001	0.110499	-0.10717	-0.019835	-0.018201
45.0001	0.153929	-0.146463	-0.012454	-0.045688
46.	0.123102	-0.103619	-0.023158	-0.062296
46.9999	0.128953	-0.122284	-0.027668	-0.030167
48.0003	0.118849	-0.114252	-0.019721	-0.026128
49.0003	0.146281	-0.142356	0.003366	-0.033487
50.0002	0.08175	-0.079182	-0.008803	-0.018321

(0,-5,0) Blue offset -12.8A White Offset 24.7A RMF on

```
Data = Import[NotebookDirectory[] <> "x0_y-5_z0_B-12_8_W24_7_RMFon.txt", "Table"];
       importi··· Notebook-Verzeichnis                                Tabelle

Bvst = Table[{AbsoluteTime[Data[[i]][[9]]] - AbsoluteTime[Data[[2]][[9]]],
       Tabelle  absolute Zeit seit 1900          absolute Zeit seit 1900
    AbsoluteTime[Data[[i]][[2]]]}, {i, 2, Length[Data]}];
    absolute Zeit seit 1900                 Länge
Bxvst = Table[{AbsoluteTime[Data[[i]][[9]]] - AbsoluteTime[Data[[2]][[9]]],
        Tabelle  absolute Zeit seit 1900          absolute Zeit seit 1900
    AbsoluteTime[Data[[i]][[3]]]}, {i, 2, Length[Data]}];
    absolute Zeit seit 1900                 Länge
Byvst = Table[{AbsoluteTime[Data[[i]][[9]]] - AbsoluteTime[Data[[2]][[9]]],
        Tabelle  absolute Zeit seit 1900          absolute Zeit seit 1900
    AbsoluteTime[Data[[i]][[4]]]}, {i, 2, Length[Data]}];
    absolute Zeit seit 1900                 Länge
Bzvst = Table[{AbsoluteTime[Data[[i]][[9]]] - AbsoluteTime[Data[[2]][[9]]],
        Tabelle  absolute Zeit seit 1900          absolute Zeit seit 1900
    AbsoluteTime[Data[[i]][[5]]]}, {i, 2, Length[Data]}];
    absolute Zeit seit 1900                 Länge
```

```
ListPlot[{Bvst, Bxvst, Byvst, Bzvst}, Joined → True]
listenbezogene Graphik                 verknüpft? wahr
```

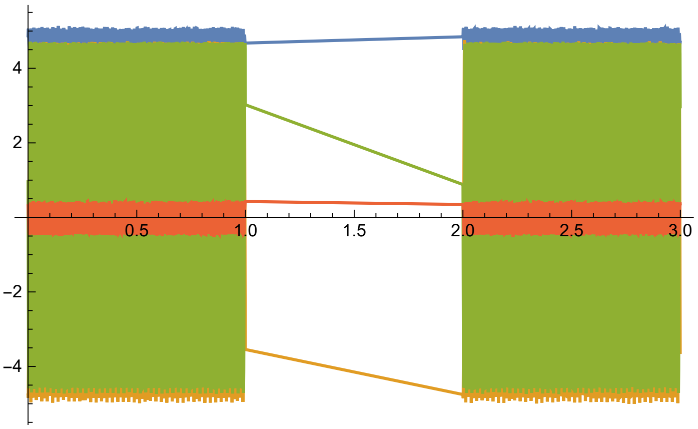


```
Start = 100;
Stop = 1000;
```

```
ListPlot[{Table[{Bvst[[i]][[1]], Bvst[[i]][[2]]}, {i, Start, Stop}],
  Table[{Bxvst[[i]][[1]], Bxvst[[i]][[2]]}, {i, Start, Stop}],
  Table[{Byvst[[i]][[1]], Byvst[[i]][[2]]}, {i, Start, Stop}],
  Table[{Bzvst[[i]][[1]], Bzvst[[i]][[2]]}, {i, Start, Stop}]}, Joined → True]
```

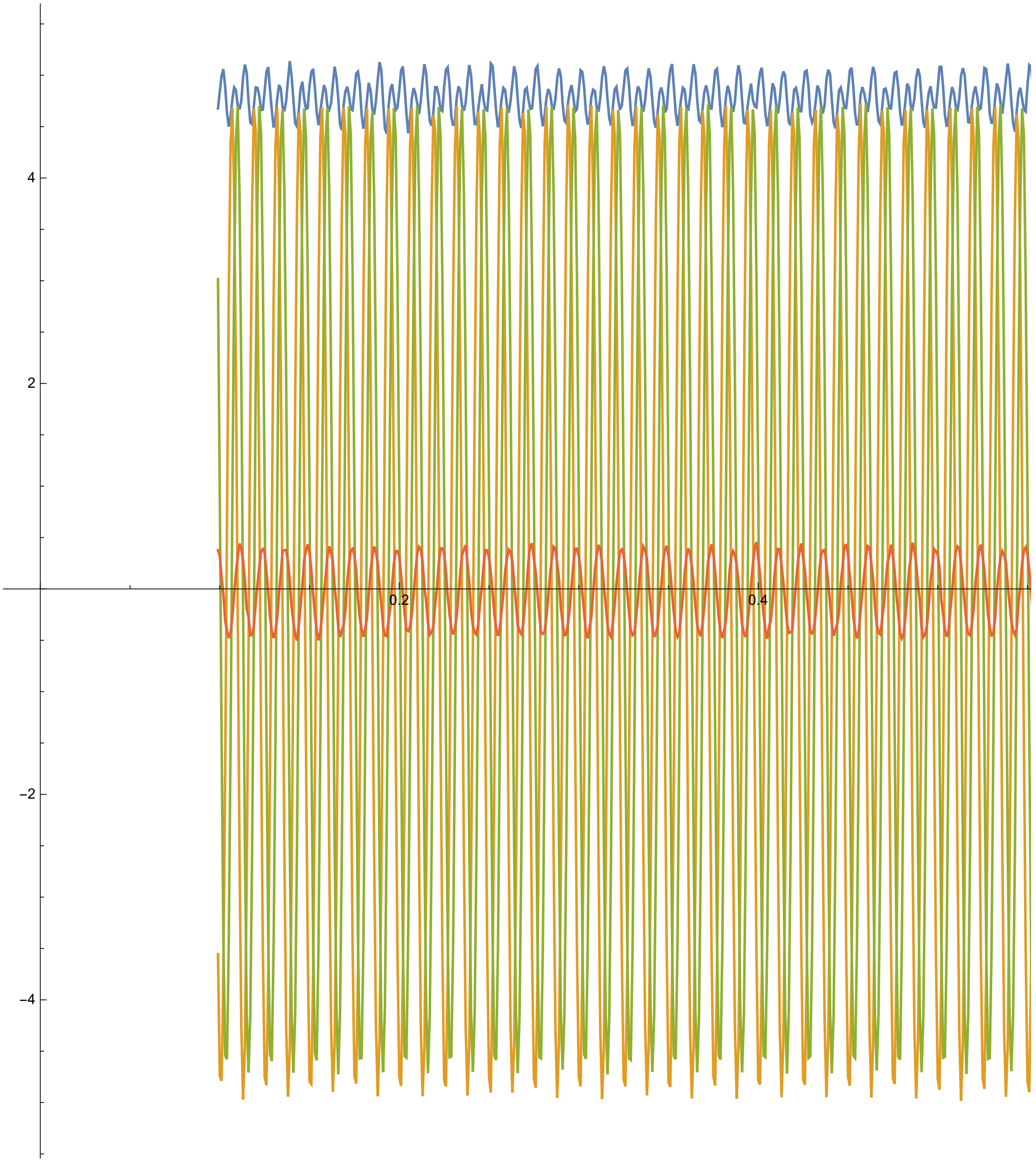

```
ListPlot[{Table[{Bvst[[i]][[1]], Bvst[[i]][[2]]}, {i, Start, Start + 50}],
  Table[{Bxvst[[i]][[1]], Bxvst[[i]][[2]]}, {i, Start, Start + 50}],
  Table[{Byvst[[i]][[1]], Byvst[[i]][[2]]}, {i, Start, Start + 50}],
  Table[{Bzvst[[i]][[1]], Bzvst[[i]][[2]]}, {i, Start, Start + 50}]}, Joined → True]
```

listenbezo··· Tabelle Tabelle Tabelle Tabelle verknüpft? wahr

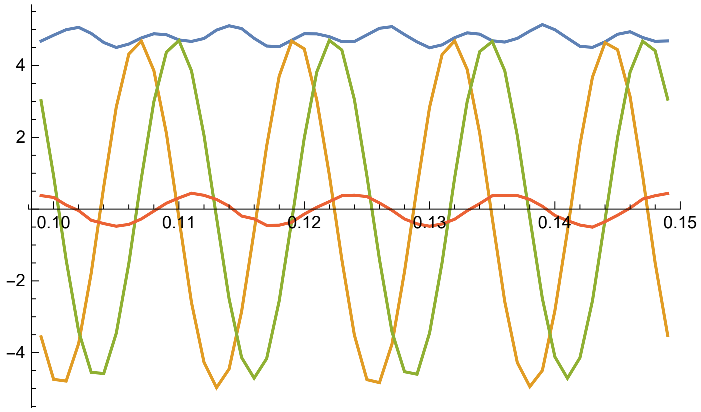


```
TableForm[{Mean[Table[Bvst[[i]][[2]], {i, Start, Stop}]],
  Mean[Table[Bxvst[[i]][[2]], {i, Start, Stop}]],
  Mean[Table[Byvst[[i]][[2]], {i, Start, Stop}]],
  Mean[Table[Bzvst[[i]][[2]], {i, Start, Stop}]]}, TableDirections → Row]
```

Tabellendars··· arit··· Tabelle arit··· Tabelle arit··· Tabelle arit··· Tabelle Richtung der Tabellen··· Zeile

4.78597 −0.125122 0.0100439 −0.0252413

```
TableForm[Table[{(Bvst[[i]][[1]] - Bvst[[Start]][[1]]) * 1000, Bvst[[i]][[2]],
Tabellendar·· Tabelle
   Bxvst[[i]][[2]], Byvst[[i]][[2]], Bzvst[[i]][[2]]}, {i, Start, Start + 50}]]
```

0.	4.67778	-3.5559	3.01595	0.375768
0.999928	4.84113	-4.74102	0.924279	0.324044
1.99986	4.99407	-4.78841	-1.41383	0.113802
2.99978	5.05946	-3.7396	-3.4076	-0.042476
3.99971	4.8908	-1.78291	-4.54362	-0.311038
5.00011	4.6384	0.622313	-4.57873	-0.403387
6.00004	4.49971	2.84034	-3.45719	-0.477145
6.99997	4.59443	4.31564	-1.51722	-0.426729
7.9999	4.7645	4.67941	0.853121	-0.275266
8.99982	4.88132	3.85709	2.99116	-0.055109
9.99975	4.85501	2.11061	4.36919	0.162972
10.9997	4.70935	-0.247377	4.69254	0.311244
12.0001	4.66785	-2.59916	3.85203	0.44168
13.	4.74954	-4.26572	2.05299	0.383386
13.9999	4.98408	-4.97122	-0.236248	0.268691
14.9999	5.10397	-4.45344	-2.49213	0.081881
15.9998	5.02608	-2.85424	-4.13246	-0.193696
16.9997	4.75492	-0.615265	-4.70701	-0.273564
18.0001	4.53959	1.75337	-4.16277	-0.452706
19.0001	4.52156	3.70162	-2.55764	-0.448286
20.	4.70113	4.67589	-0.322662	-0.364195
20.9999	4.88185	4.46146	1.97696	-0.139838
21.9998	4.87815	3.03614	3.81782	0.048993
22.9998	4.80004	0.955616	4.69936	0.207928
23.9997	4.66185	-1.40551	4.4294	0.371177
25.0001	4.66498	-3.50783	3.05084	0.386807
26.	4.84955	-4.74876	0.919904	0.348062
27.	5.03339	-4.83184	-1.39974	0.170474
27.9999	5.0796	-3.7595	-3.41574	-0.034394
28.9998	4.85964	-1.74255	-4.52812	-0.275212
29.9997	4.65513	0.602252	-4.59763	-0.41147
31.0001	4.49102	2.83634	-3.4492	-0.476981
32.0001	4.57199	4.30373	-1.48642	-0.414134
33.	4.77152	4.68331	0.868246	-0.283076
33.9999	4.90657	3.88917	2.99108	-0.04744
34.9998	4.87087	2.11447	4.38538	0.151177
35.9998	4.68584	-0.24673	4.66463	0.370821
36.9997	4.65014	-2.57581	3.85302	0.378444
38.0001	4.75235	-4.27278	2.04609	0.376353
39.	4.95646	-4.94322	-0.243214	0.268296
40.	5.13695	-4.49348	-2.48811	0.078354
40.9999	4.99381	-2.8271	-4.11268	-0.177671
41.9998	4.76122	-0.615755	-4.71027	-0.321617
42.9997	4.53257	1.79343	-4.13885	-0.444752
43.9997	4.50224	3.67705	-2.54883	-0.502907
45.0001	4.65616	4.63299	-0.302899	-0.351449
46.	4.86693	4.43711	1.99242	-0.171357
46.9999	4.93731	3.13494	3.81423	0.028916
47.9999	4.77703	0.940373	4.67526	0.278729
48.9998	4.67115	-1.49648	4.4093	0.371808
49.9997	4.68224	-3.51534	3.0621	0.435065

(0,-5,0) Blue offset 13.4A White Offset -29A RMF off

```
Data = Import[NotebookDirectory[] <> "x0_y-5_z0_B13_4_W-29_RMFoff.txt", "Table"];
```

```
Bvst = Table[{AbsoluteTime[Data[[i]][[9]]] - AbsoluteTime[Data[[2]][[9]]],
    AbsoluteTime[Data[[i]][[2]]]}, {i, 2, Length[Data]}];
Bxvst = Table[{AbsoluteTime[Data[[i]][[9]]] - AbsoluteTime[Data[[2]][[9]]],
    AbsoluteTime[Data[[i]][[3]]]}, {i, 2, Length[Data]}];
Byvst = Table[{AbsoluteTime[Data[[i]][[9]]] - AbsoluteTime[Data[[2]][[9]]],
    AbsoluteTime[Data[[i]][[4]]]}, {i, 2, Length[Data]}];
Bzvst = Table[{AbsoluteTime[Data[[i]][[9]]] - AbsoluteTime[Data[[2]][[9]]],
    AbsoluteTime[Data[[i]][[5]]]}, {i, 2, Length[Data]}];
```

```
ListPlot[{Bvst, Bxvst, Byvst, Bzvst}, Joined → True]
```

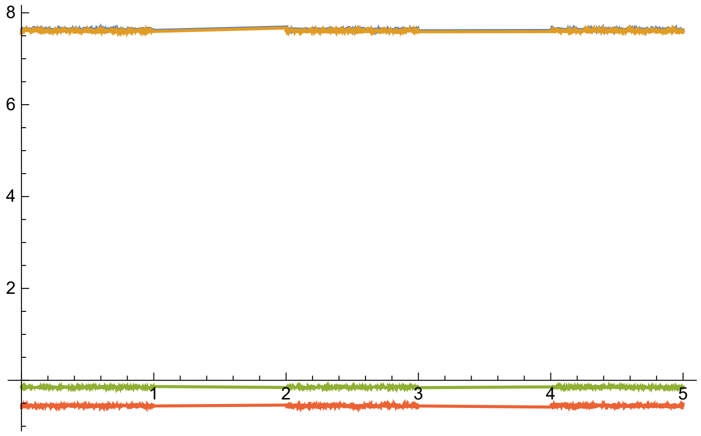


```
Start = 100;
Stop = 1000;
```

```
ListPlot[{Table[{Bvst[[i]][[1]], Bvst[[i]][[2]]}, {i, Start, Stop}],
 Table[{Bxvst[[i]][[1]], Bxvst[[i]][[2]]}, {i, Start, Stop}],
 Table[{Byvst[[i]][[1]], Byvst[[i]][[2]]}, {i, Start, Stop}],
 Table[{Bzvst[[i]][[1]], Bzvst[[i]][[2]]}, {i, Start, Stop}]}, Joined → True]
```

listenbezo··· Tabelle Tabelle Tabelle Tabelle verknüpft? wahr

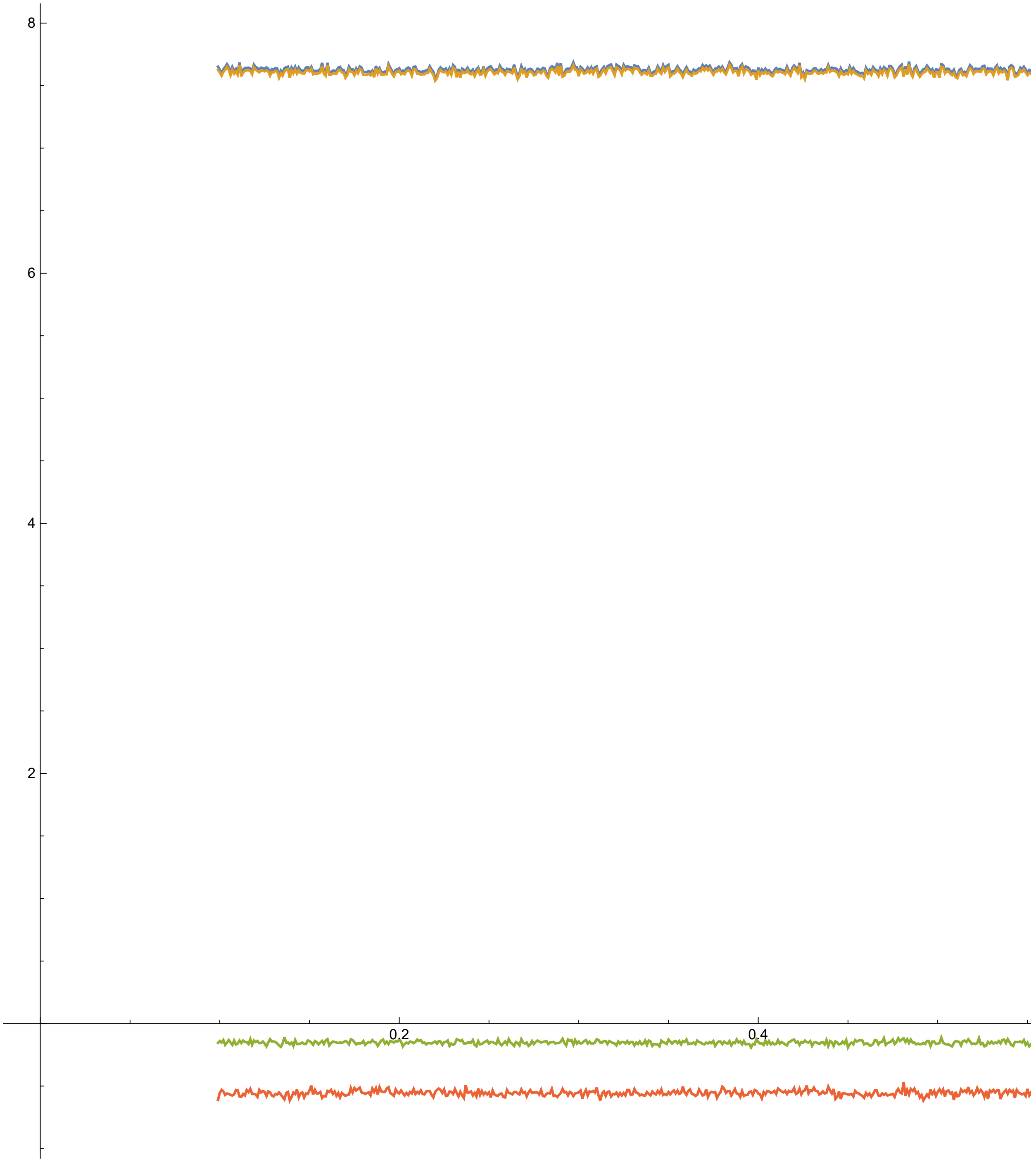

```
ListPlot[{Table[{Bvst[[i]][[1]], Bvst[[i]][[2]]}, {i, Start, Start + 50}],
  Table[{Bxvst[[i]][[1]], Bxvst[[i]][[2]]}, {i, Start, Start + 50}],
  Table[{Byvst[[i]][[1]], Byvst[[i]][[2]]}, {i, Start, Start + 50}],
  Table[{Bzvst[[i]][[1]], Bzvst[[i]][[2]]}, {i, Start, Start + 50}]}, Joined → True]
```

listenbezo··· Tabelle Tabelle Tabelle Tabelle verknüpft? wahr

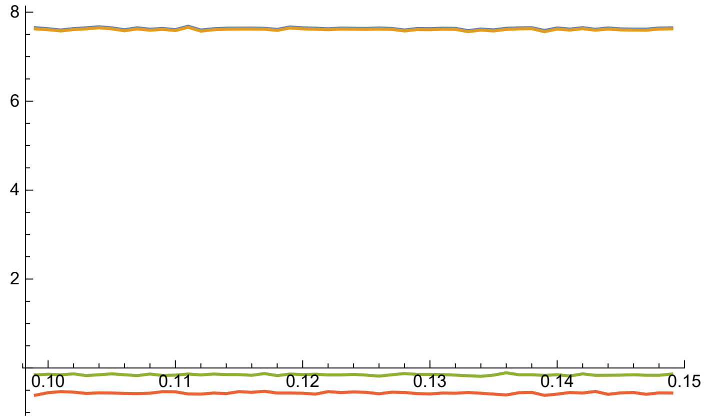


```
TableForm[{Mean[Table[Bvst[[i]][[2]], {i, Start, Stop}]],
  Mean[Table[Bxvst[[i]][[2]], {i, Start, Stop}]],
  Mean[Table[Byvst[[i]][[2]], {i, Start, Stop}]],
  Mean[Table[Bzvst[[i]][[2]], {i, Start, Stop}]]}, TableDirections → Row]
```

Tabellendars··· arit··· Tabelle arit··· Tabelle arit··· Tabelle arit··· Tabelle Richtung der Tabellen··· Zeile

7.62824 7.60653 -0.152139 -0.554076

```
TableForm[Table[{(Bvst[[i]][[1]] - Bvst[[Start]][[1]]) * 1000, Bvst[[i]][[2]],
Tabellendar·· Tabelle
   Bxvst[[i]][[2]], Byvst[[i]][[2]], Bzvst[[i]][[2]]}, {i, Start, Start + 50}]]
```

0.	7.65132	7.6253	-0.154835	-0.611082
1.0004	7.62734	7.60587	-0.139716	-0.554642
2.00033	7.5992	7.57913	-0.152113	-0.530551
3.00026	7.63044	7.60998	-0.131895	-0.542563
4.00019	7.649	7.62573	-0.170444	-0.571318
5.00011	7.67175	7.64983	-0.151601	-0.559277
6.00004	7.64764	7.62577	-0.131571	-0.562724
7.00045	7.60566	7.58271	-0.151498	-0.570586
8.00037	7.64926	7.62569	-0.170383	-0.575318
9.0003	7.61704	7.59474	-0.135546	-0.566465
10.0002	7.63447	7.61414	-0.16707	-0.531141
11.0002	7.6076	7.58709	-0.160041	-0.534755
12.0001	7.68492	7.66157	-0.135222	-0.583152
13.	7.59882	7.57455	-0.155262	-0.586562
14.0004	7.62776	7.60578	-0.135594	-0.562579
15.0003	7.64075	7.61767	-0.147395	-0.574884
16.0003	7.64202	7.62212	-0.148062	-0.530933
17.0002	7.64334	7.62197	-0.163816	-0.547174
18.0001	7.63711	7.61818	-0.125192	-0.522541
19.0001	7.61357	7.59082	-0.170608	-0.56296
20.	7.66767	7.64578	-0.135547	-0.562991
21.0004	7.64822	7.62575	-0.147508	-0.566968
22.0003	7.64137	7.61754	-0.139213	-0.58676
23.0002	7.62623	7.60613	-0.15608	-0.53089
24.0002	7.6434	7.62192	-0.155756	-0.551051
25.0001	7.63842	7.61803	-0.143945	-0.538829
26.	7.63527	7.61397	-0.159826	-0.547031
27.0004	7.64583	7.62166	-0.182325	-0.579459
28.0004	7.63484	7.614	-0.151888	-0.542909
29.0003	7.59986	7.57889	-0.124811	-0.550135
30.0002	7.63268	7.60966	-0.143405	-0.574741
31.0001	7.62922	7.60558	-0.143288	-0.582698
32.0001	7.63998	7.61779	-0.147579	-0.562886
33.	7.63651	7.61376	-0.159521	-0.567028
34.0004	7.58499	7.56295	-0.174824	-0.550734
35.0003	7.62122	7.59779	-0.186537	-0.567275
36.0003	7.6029	7.57856	-0.159257	-0.586664
37.0002	7.63816	7.6133	-0.107916	-0.606242
38.0001	7.64755	7.62588	-0.151691	-0.555031
39.	7.65105	7.62996	-0.151808	-0.547073
40.0004	7.58902	7.56228	-0.165848	-0.614601
41.0004	7.64561	7.62155	-0.151207	-0.586984
42.0003	7.62001	7.59795	-0.178782	-0.551156
43.0002	7.65163	7.62977	-0.131566	-0.562765
44.0001	7.6152	7.59518	-0.164154	-0.5269
45.0001	7.64615	7.62152	-0.163145	-0.591167
46.	7.62404	7.60185	-0.159657	-0.558905
47.0004	7.61937	7.59792	-0.151785	-0.550743
48.0003	7.61922	7.59452	-0.163177	-0.590889
49.0003	7.64408	7.62185	-0.163633	-0.559172
50.0002	7.64772	7.62578	-0.135571	-0.562785

(0,-5,0) Blue offset 13.4A White Offset -29A RMF on

```
Data = Import[NotebookDirectory[] <> "x0_y-5_z0_B13_4_W-29_RMFon.txt", "Table"];
        importi··· Notebook-Verzeichnis                                  Tabelle

Bvst = Table[{AbsoluteTime[Data[[i]][[9]]] - AbsoluteTime[Data[[2]][[9]]],
        Tabelle  absolute Zeit seit 1900          absolute Zeit seit 1900
    AbsoluteTime[Data[[i]][[2]]]}, {i, 2, Length[Data]}];
    absolute Zeit seit 1900                     Länge
Bxvst = Table[{AbsoluteTime[Data[[i]][[9]]] - AbsoluteTime[Data[[2]][[9]]],
         Tabelle  absolute Zeit seit 1900          absolute Zeit seit 1900
    AbsoluteTime[Data[[i]][[3]]]}, {i, 2, Length[Data]}];
    absolute Zeit seit 1900                     Länge
Byvst = Table[{AbsoluteTime[Data[[i]][[9]]] - AbsoluteTime[Data[[2]][[9]]],
         Tabelle  absolute Zeit seit 1900          absolute Zeit seit 1900
    AbsoluteTime[Data[[i]][[4]]]}, {i, 2, Length[Data]}];
    absolute Zeit seit 1900                     Länge
Bzvst = Table[{AbsoluteTime[Data[[i]][[9]]] - AbsoluteTime[Data[[2]][[9]]],
         Tabelle  absolute Zeit seit 1900          absolute Zeit seit 1900
    AbsoluteTime[Data[[i]][[5]]]}, {i, 2, Length[Data]}];
    absolute Zeit seit 1900                     Länge
```

```
ListPlot[{Bvst, Bxvst, Byvst, Bzvst}, Joined → True]
listenbezogene Graphik                  verknüpft? wahr
```

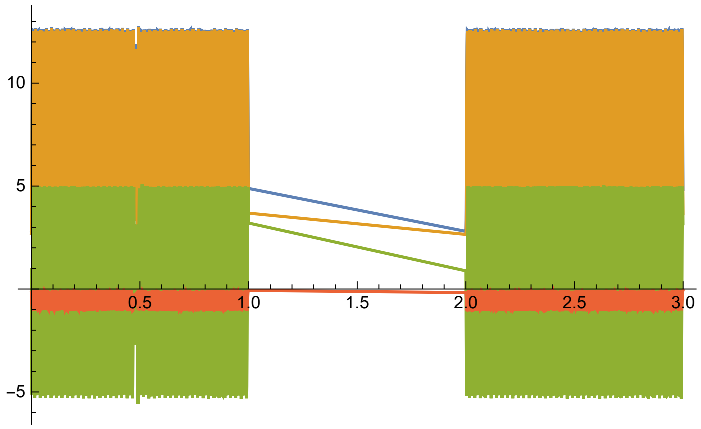


```
Start = 100;
Stop = 1000;
```

```
ListPlot[{Table[{Bvst[[i]][[1]], Bvst[[i]][[2]]}, {i, Start, Stop}],
  Table[{Bxvst[[i]][[1]], Bxvst[[i]][[2]]}, {i, Start, Stop}],
  Table[{Byvst[[i]][[1]], Byvst[[i]][[2]]}, {i, Start, Stop}],
  Table[{Bzvst[[i]][[1]], Bzvst[[i]][[2]]}, {i, Start, Stop}]}, Joined → True]
```

listenbezo··· Tabelle Tabelle Tabelle Tabelle verknüpft? wahr

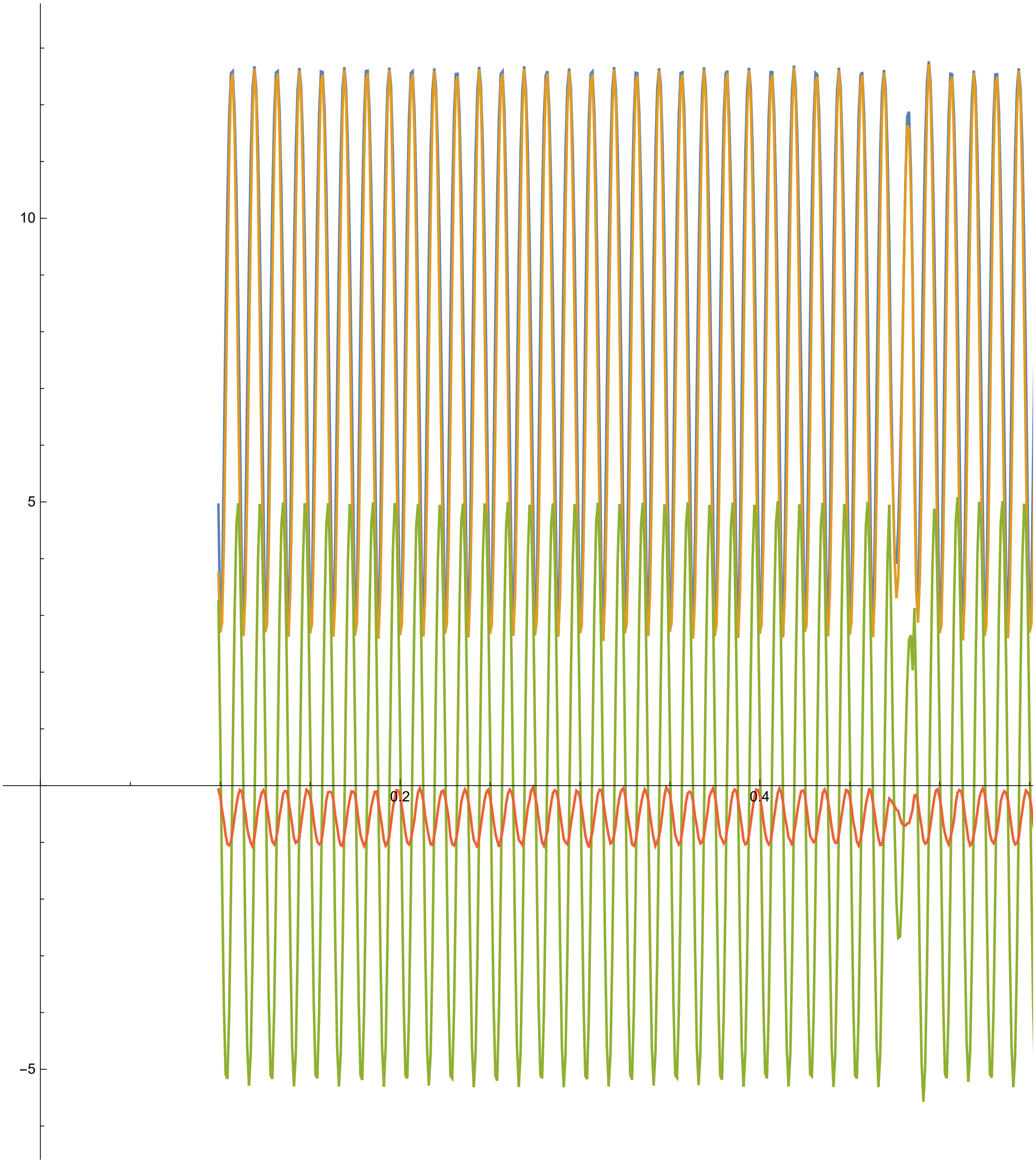

```
ListPlot[{Table[{Bvst[[i]][[1]], Bvst[[i]][[2]]}, {i, Start, Start + 50}],
listenbezo⋯ Tabelle
  Table[{Bxvst[[i]][[1]], Bxvst[[i]][[2]]}, {i, Start, Start + 50}],
  Tabelle
  Table[{Byvst[[i]][[1]], Byvst[[i]][[2]]}, {i, Start, Start + 50}],
  Tabelle
  Table[{Bzvst[[i]][[1]], Bzvst[[i]][[2]]}, {i, Start, Start + 50}]}, Joined → True]
  Tabelle                                                               verknüpft? wahr
```

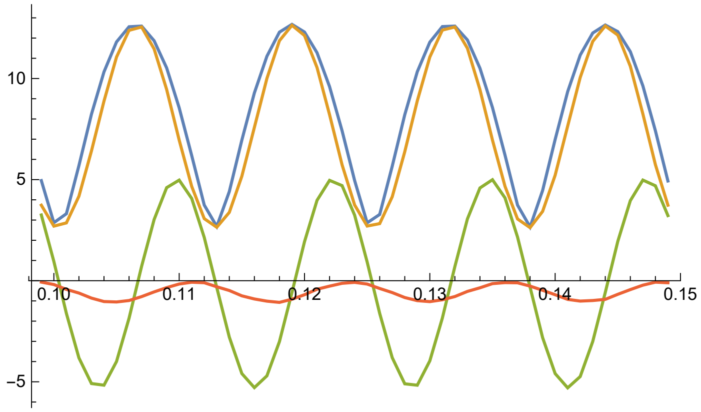


```
TableForm[{Mean[Table[Bvst[[i]][[2]], {i, Start, Stop}]],
Tabellendars⋯ arit⋯ Tabelle
  Mean[Table[Bxvst[[i]][[2]], {i, Start, Stop}]],
  arit⋯ Tabelle
  Mean[Table[Byvst[[i]][[2]], {i, Start, Stop}]],
  arit⋯ Tabelle
  Mean[Table[Bzvst[[i]][[2]], {i, Start, Stop}]]}, TableDirections → Row]
  arit⋯ Tabelle                                      Richtung der Tabellen⋯ Zeile
```

```
8.51554  7.60434  -0.139318  -0.555449
```

```
TableForm[Table[{(Bvst[[i]][[1]] - Bvst[[Start]][[1]]) * 1000, Bvst[[i]][[2]],
  Bxvst[[i]][[2]], Byvst[[i]][[2]], Bzvst[[i]][[2]]}, {i, Start, Start + 50}]]
```

Tabellendar·· Tabelle

0.	4.95144	3.73463	3.25029	-0.069893
0.999928	2.86822	2.69907	0.951782	-0.189328
1.99986	3.31256	2.85865	-1.61769	-0.429242
2.99978	5.6929	4.17846	-3.81781	-0.611469
3.99971	8.23418	6.41752	-5.08684	-0.860969
4.99964	10.3284	8.883	-5.16772	-1.03065
6.00004	11.809	11.0625	-3.99602	-1.05124
6.99997	12.5595	12.3828	-1.8565	-0.980173
7.9999	12.5932	12.5487	0.706031	-0.787729
8.99982	11.8683	11.4633	3.02362	-0.554117
9.99975	10.524	9.45852	4.60152	-0.343322
10.9997	8.55434	6.95679	4.97524	-0.163794
11.9996	6.18929	4.66365	4.06842	-0.074982
13.	3.74241	3.0686	2.13994	-0.099983
13.9999	2.68221	2.64345	-0.340729	-0.300506
14.9999	4.40872	3.3795	-2.78945	-0.48451
15.9998	6.95282	5.16602	-4.59279	-0.748505
16.9997	9.28799	7.58139	-5.28902	-0.903062
17.9996	11.1005	10.0027	-4.70581	-1.01114
19.0001	12.2937	11.8702	-3.01375	-1.07257
20.	12.6785	12.6319	-0.583086	-0.915289
20.9999	12.298	12.1262	1.9273	-0.693712
21.9998	11.2748	10.5425	3.97478	-0.42308
22.9998	9.59802	8.21079	4.96314	-0.268925
23.9997	7.41381	5.72941	4.70341	-0.128284
24.9996	4.95473	3.74551	3.24249	-0.082127
26.	2.86184	2.70334	0.924423	-0.165786
27.	3.27678	2.82311	-1.6184	-0.384879
27.9999	5.66216	4.1507	-3.80621	-0.587001
28.9998	8.20811	6.37782	-5.09931	-0.832746
29.9997	10.3351	8.89545	-5.16838	-0.986782
30.9997	11.7973	11.0626	-3.96527	-1.03477
32.0001	12.5678	12.3951	-1.84904	-0.94418
33.	12.5928	12.5488	0.70197	-0.783791
33.9999	11.9062	11.4955	3.05428	-0.529977
34.9998	10.5106	9.45045	4.58663	-0.351468
35.9998	8.56236	6.95296	4.99495	-0.144451
36.9997	6.21103	4.66742	4.09673	-0.094592
37.9996	3.7403	3.05254	2.15898	-0.103528
39.	2.65703	2.62376	-0.322243	-0.268015
40.	4.46161	3.42765	-2.81657	-0.473419
40.9999	6.98462	5.21559	-4.59357	-0.694019
41.9998	9.35064	7.64819	-5.29964	-0.923916
42.9997	11.1575	10.0498	-4.74081	-1.00816
43.9997	12.2505	11.8351	-3.0072	-0.980094
45.0001	12.6471	12.5999	-0.583002	-0.922959
46.	12.3197	12.1464	1.94602	-0.673631
46.9999	11.3291	10.6053	3.9601	-0.439955
47.9999	9.63884	8.25126	4.97745	-0.221116
48.9998	7.41782	5.74199	4.69557	-0.072524
49.9997	4.9318	3.73433	3.21978	-0.102361
