# Supplementary material for: Spatially selective delivery of living magnetic microrobots through torque-focusing
Source: Nat Commun. 2024 Mar 9;15:2160. doi: 10.1038/s41467-024-46407-4 (PMC10924878; doi:10.1038/s41467-024-46407-4)
Supplement: Supplementary file 4 — Source Data [file 41467_2024_46407_MOESM4_ESM.zip › Data_Sorted_by_Figures/Fig_4/4I/Field Characterization/y axis measurements/x0_y0_z0/ReadingHallProbeData.pdf]

```
NotebookDirectory[]
Notebook-Verzeichnis
```

P:\Michael\MetroLabHallProbe\230223 files\

(0,0,0) RMF off

```
Data = Import[NotebookDirectory[] <> "230223_zero_point_field_off.txt", "Table"];
         importi··· Notebook-Verzeichnis                                    Tabelle

Bvst = Table[{AbsoluteTime[Data[[i]][[9]]] - AbsoluteTime[Data[[2]][[9]]],
       Tabelle  absolute Zeit seit 1900              absolute Zeit seit 1900
    AbsoluteTime[Data[[i]][[2]]]}, {i, 2, Length[Data]}];
    absolute Zeit seit 1900                    Länge
Bxvst = Table[{AbsoluteTime[Data[[i]][[9]]] - AbsoluteTime[Data[[2]][[9]]],
        Tabelle  absolute Zeit seit 1900              absolute Zeit seit 1900
    AbsoluteTime[Data[[i]][[3]]]}, {i, 2, Length[Data]}];
    absolute Zeit seit 1900                    Länge
Byvst = Table[{AbsoluteTime[Data[[i]][[9]]] - AbsoluteTime[Data[[2]][[9]]],
        Tabelle  absolute Zeit seit 1900              absolute Zeit seit 1900
    AbsoluteTime[Data[[i]][[4]]]}, {i, 2, Length[Data]}];
    absolute Zeit seit 1900                    Länge
Bzvst = Table[{AbsoluteTime[Data[[i]][[9]]] - AbsoluteTime[Data[[2]][[9]]],
        Tabelle  absolute Zeit seit 1900              absolute Zeit seit 1900
    AbsoluteTime[Data[[i]][[5]]]}, {i, 2, Length[Data]}];
    absolute Zeit seit 1900                    Länge
```

```
ListPlot[{Bvst, Bxvst, Byvst, Bzvst}, Joined → True]
listenbezogene Graphik                  verknüpft? wahr
```

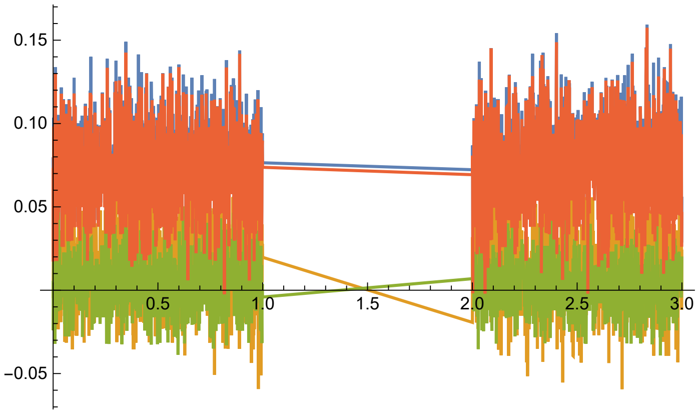


```
Start = 100;
Stop = 1000;
```

```
ListPlot[{Table[{Bvst[[i]][[1]], Bvst[[i]][[2]]}, {i, Start, Stop}],
  Table[{Bxvst[[i]][[1]], Bxvst[[i]][[2]]}, {i, Start, Stop}],
  Table[{Byvst[[i]][[1]], Byvst[[i]][[2]]}, {i, Start, Stop}],
  Table[{Bzvst[[i]][[1]], Bzvst[[i]][[2]]}, {i, Start, Stop}]}, Joined → True]
```

listenbezo··· Tabelle Tabelle Tabelle Tabelle verknüpft? wahr

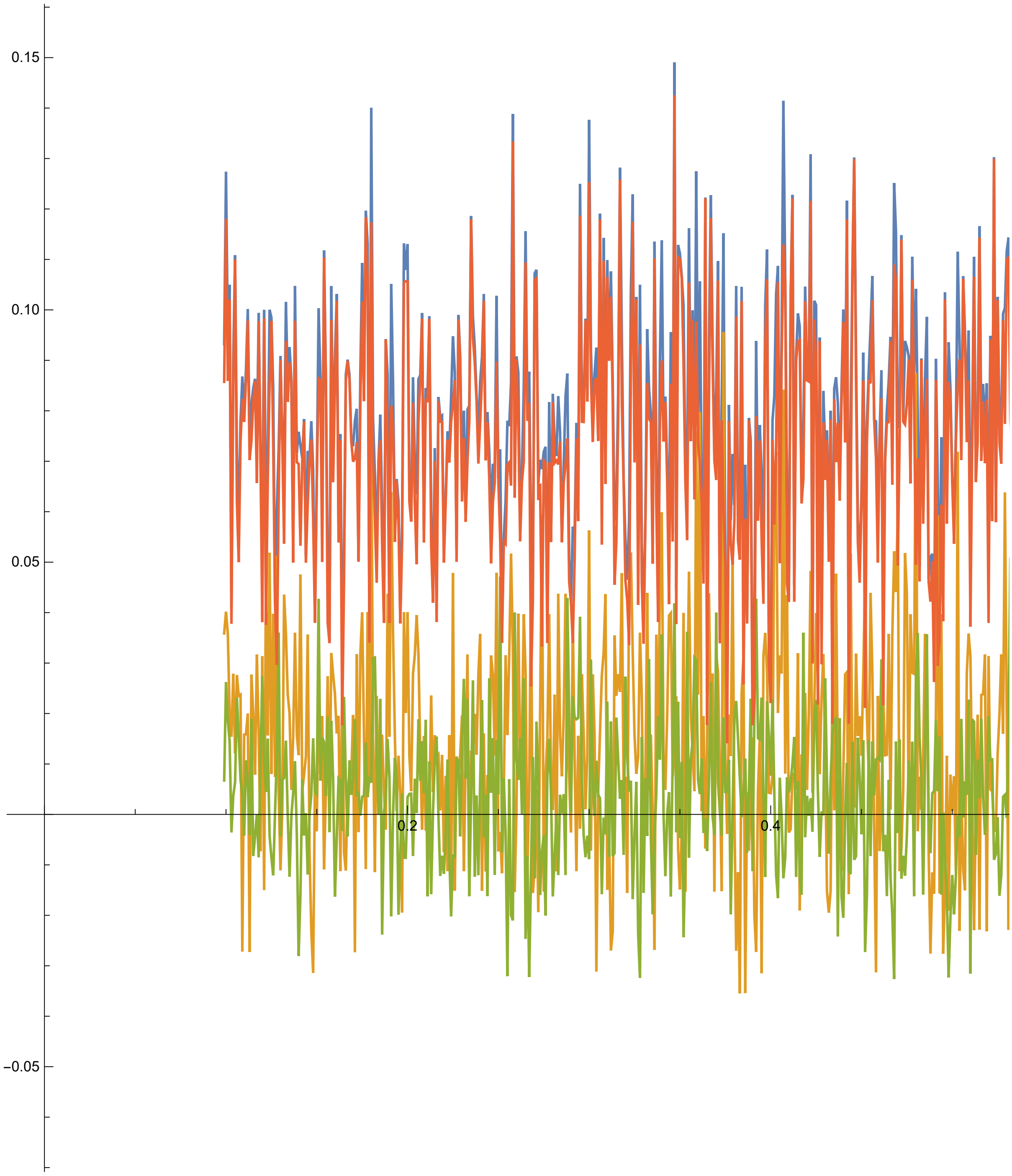

```
ListPlot[{Table[{Bvst[[i]][[1]], Bvst[[i]][[2]]}, {i, Start, Start + 50}],
  Table[{Bxvst[[i]][[1]], Bxvst[[i]][[2]]}, {i, Start, Start + 50}],
  Table[{Byvst[[i]][[1]], Byvst[[i]][[2]]}, {i, Start, Start + 50}],
  Table[{Bzvst[[i]][[1]], Bzvst[[i]][[2]]}, {i, Start, Start + 50}]}, Joined → True]
```

listenbezo··· Tabelle Tabelle Tabelle Tabelle verknüpft? wahr

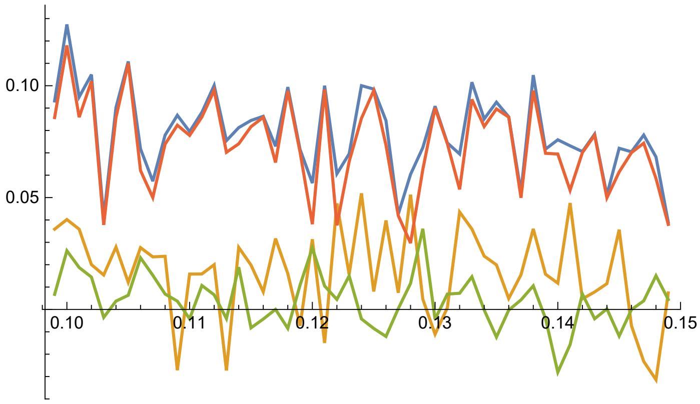


```
TableForm[{Mean[Table[Bvst[[i]][[2]], {i, Start, Stop}]],
  Mean[Table[Bxvst[[i]][[2]], {i, Start, Stop}]],
  Mean[Table[Byvst[[i]][[2]], {i, Start, Stop}]],
  Mean[Table[Bzvst[[i]][[2]], {i, Start, Stop}]]}, TableDirections → Row]
```

Tabellendars··· arit··· Tabelle arit··· Tabelle arit··· Tabelle arit··· Tabelle Richtung der Tabellen··· Zeile

```
0.0789356  0.0150732  0.00346714  0.0722405
```

```
TableForm[Table[{(Bvst[[i]][[1]] - Bvst[[Start]][[1]]) * 1000, Bvst[[i]][[2]],
    Bxvst[[i]][[2]], Byvst[[i]][[2]], Bzvst[[i]][[2]]}, {i, Start, Start + 50}]]
```
Tabellendar⋯ Tabelle

0.	0.093193	0.035877	0.006728	0.085747
0.999928	0.127393	0.040186	0.026241	0.118006
1.99986	0.094979	0.035864	0.018726	0.085931
2.99978	0.104981	0.020034	0.014463	0.102032
3.99971	0.040963	0.015394	-0.003562	0.037793
4.99964	0.090277	0.02788	0.003718	0.085784
6.00004	0.110838	0.012125	0.006333	0.10999
6.99997	0.071757	0.027613	0.023083	0.062078
7.9999	0.057315	0.023498	0.015262	0.049999
8.99982	0.077904	0.023753	0.006897	0.073873
9.99975	0.086793	-0.027158	0.003714	0.082351
10.9997	0.079486	0.015806	-0.004173	0.077787
12.0001	0.088119	0.015873	0.010703	0.086014
13.	0.100145	0.020001	0.006525	0.09791
13.9999	0.075452	-0.027274	-0.004102	0.07023
14.9999	0.081271	0.027741	0.0189	0.074015
15.9998	0.084463	0.019852	-0.008229	0.081684
16.9997	0.086337	0.007889	-0.004304	0.085868
17.9996	0.072925	0.031678	0.0000290462	0.065685
19.0001	0.099388	0.016017	-0.008478	0.097722
20.	0.07147	-0.00729	0.01092	0.070254
20.9999	0.056471	0.031361	0.027453	0.038102
21.9998	0.10002	-0.015001	0.010483	0.098331
22.9998	0.060647	0.047384	0.004475	0.037586
23.9997	0.069549	0.015663	0.015008	0.066079
24.9996	0.10003	0.051887	-0.004252	0.085415
26.	0.098499	0.008017	-0.008487	0.097804
27.	0.084368	0.039772	-0.012082	0.073418
27.9999	0.042591	0.007432	0.000367	0.041936
28.9998	0.060374	0.051294	0.011601	0.029653
29.9997	0.072312	0.004601	0.036054	0.062514
30.9997	0.090846	-0.011068	-0.004388	0.090062
32.0001	0.074431	0.000755	0.006869	0.07411
33.	0.069491	0.043546	0.007226	0.05367
33.9999	0.101573	0.035951	0.014605	0.093868
34.9998	0.08517	0.023843	-0.000225	0.081765
35.9998	0.092639	0.019938	-0.01235	0.089621
36.9997	0.086099	0.004885	-0.000308	0.08596
37.9996	0.05244	0.01551	0.004254	0.049914
39.	0.104752	0.035996	0.010544	0.097806
40.	0.071652	0.015724	-0.004051	0.069788
40.9999	0.075829	0.011749	-0.028052	0.069462
41.9998	0.073144	0.04757	-0.015767	0.053278
42.9997	0.070569	0.004713	0.006935	0.070069
43.9997	0.078371	0.007807	-0.004182	0.077869
45.0001	0.051205	0.011514	0.00025	0.049894
46.	0.072042	0.035649	-0.011904	0.061461
46.9999	0.070463	-0.007279	-0.0000782889	0.070086
47.9999	0.077955	-0.023241	0.003841	0.074311
48.9998	0.068147	-0.031417	0.015075	0.058564
49.9997	0.038962	0.007386	0.004428	0.037998

(0,0,0) RMF on

```
Data = Import[NotebookDirectory[] <> "230223_zero_point_field_on_5mT.txt", "Table"];
```
importi⋯ Notebook-Verzeichnis Tabelle

```
Bvst = Table[{AbsoluteTime[Data[[i]][[9]]] - AbsoluteTime[Data[[2]][[9]]],
        Tabelle  absolute Zeit seit 1900                absolute Zeit seit 1900
    AbsoluteTime[Data[[i]][[2]]]}, {i, 2, Length[Data]}];
    absolute Zeit seit 1900                   Länge
Bxvst = Table[{AbsoluteTime[Data[[i]][[9]]] - AbsoluteTime[Data[[2]][[9]]],
        Tabelle  absolute Zeit seit 1900                absolute Zeit seit 1900
    AbsoluteTime[Data[[i]][[3]]]}, {i, 2, Length[Data]}];
    absolute Zeit seit 1900                   Länge
Byvst = Table[{AbsoluteTime[Data[[i]][[9]]] - AbsoluteTime[Data[[2]][[9]]],
        Tabelle  absolute Zeit seit 1900                absolute Zeit seit 1900
    AbsoluteTime[Data[[i]][[4]]]}, {i, 2, Length[Data]}];
    absolute Zeit seit 1900                   Länge
Bzvst = Table[{AbsoluteTime[Data[[i]][[9]]] - AbsoluteTime[Data[[2]][[9]]],
        Tabelle  absolute Zeit seit 1900                absolute Zeit seit 1900
    AbsoluteTime[Data[[i]][[5]]]}, {i, 2, Length[Data]}];
    absolute Zeit seit 1900                   Länge
```

```
ListPlot[{Bvst, Bxvst, Byvst, Bzvst}, Joined → True]
listenbezogene Graphik                 verknüpft? wahr
```

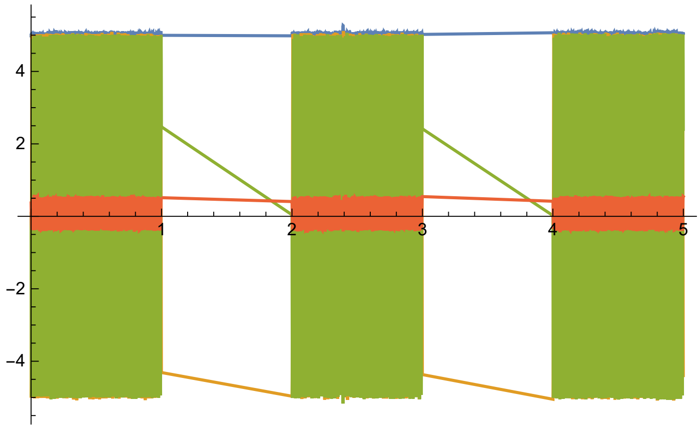


```
Start = 1;
Stop = 1000;
```

```
ListPlot[{Table[{Bvst[[i]][[1]], Bvst[[i]][[2]]}, {i, Start, Stop}],
  Table[{Bxvst[[i]][[1]], Bxvst[[i]][[2]]}, {i, Start, Stop}],
  Table[{Byvst[[i]][[1]], Byvst[[i]][[2]]}, {i, Start, Stop}],
  Table[{Bzvst[[i]][[1]], Bzvst[[i]][[2]]}, {i, Start, Stop}]}, Joined → True]
```

listenbezo⋯ Tabelle Tabelle Tabelle Tabelle verknüpft? wahr

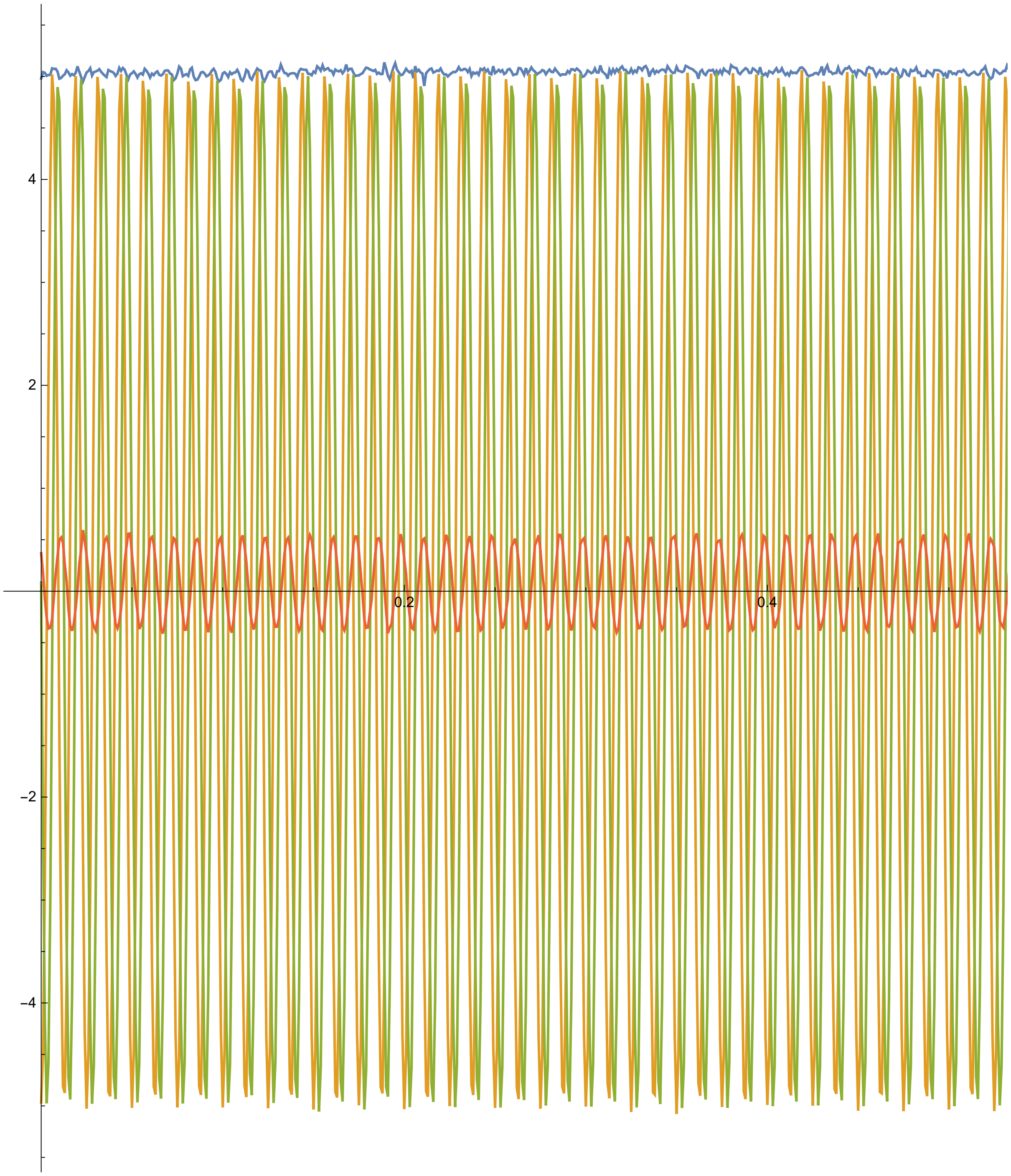

```
ListPlot[{Table[{Bvst[[i]][[1]], Bvst[[i]][[2]]}, {i, Start, Start + 50}],
  Table[{Bxvst[[i]][[1]], Bxvst[[i]][[2]]}, {i, Start, Start + 50}],
  Table[{Byvst[[i]][[1]], Byvst[[i]][[2]]}, {i, Start, Start + 50}],
  Table[{Bzvst[[i]][[1]], Bzvst[[i]][[2]]}, {i, Start, Start + 50}]},
 Joined → True, PlotLegends → {"B", "Bx", "By", "Bz"}]
```

listenbezo··· Tabelle Tabelle Tabelle Tabelle verknüpft? wahr Legenden der Graphik

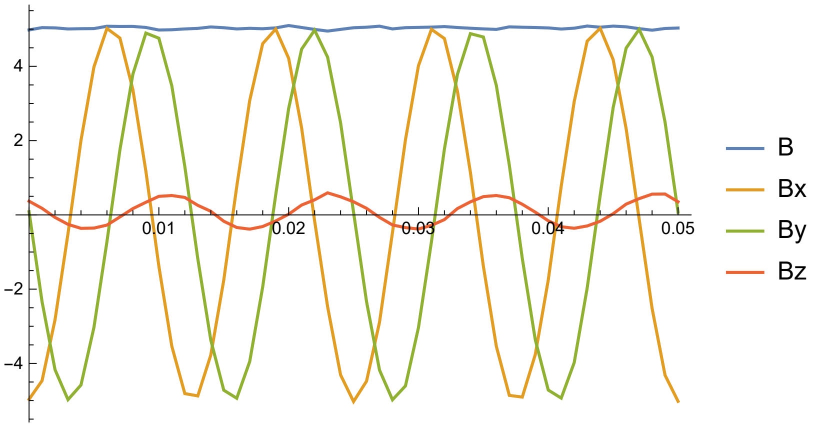


```
TableForm[{Mean[Table[Bvst[[i]][[2]], {i, Start, Stop}]],
  Mean[Table[Bxvst[[i]][[2]], {i, Start, Stop}]],
  Mean[Table[Byvst[[i]][[2]], {i, Start, Stop}]],
  Mean[Table[Bzvst[[i]][[2]], {i, Start, Stop}]]}, TableDirections → Row]
```

Tabellendars··· arit··· Tabelle arit··· Tabelle arit··· Tabelle arit··· Tabelle Richtung der Tabellen··· Zeile

```
5.04218  0.0161301  -0.00740934  0.0811664
```

```
TableForm[Table[{(Bvst[[i]][[1]] - Bvst[[Start]][[1]]) * 1000, Bvst[[i]][[2]],
Tabellendar·· Tabelle
   Bxvst[[i]][[2]], Byvst[[i]][[2]], Bzvst[[i]][[2]]}, {i, Start, Start + 50}]]
```

0.	4.98096	-4.96657	0.085268	0.368568
0.999928	5.04262	-4.46565	-2.33556	0.176413
1.99986	5.03586	-2.8249	-4.16836	-0.067525
2.99978	5.00713	-0.503747	-4.97521	-0.254806
3.99971	5.01396	2.00581	-4.58093	-0.362677
4.99964	5.01691	3.98515	-3.02683	-0.355356
6.00004	5.07906	5.0203	-0.71972	-0.274792
6.99997	5.07364	4.76161	1.75095	-0.055369
7.9999	5.07494	3.37043	3.79033	0.169147
8.99982	5.04722	1.17575	4.89659	0.339692
9.99975	4.97885	-1.38058	4.75751	0.49895
10.9997	4.98563	-3.53894	3.4728	0.521593
12.0001	5.00734	-4.81197	1.30303	0.469583
13.	5.02186	-4.87421	-1.18109	0.257264
13.9999	5.06058	-3.7742	-3.36977	0.097482
14.9999	5.0401	-1.75725	-4.72075	-0.170983
15.9998	5.00779	0.763406	-4.93761	-0.33931
16.9997	5.02369	3.0809	-3.94942	-0.384124
17.9996	5.01247	4.61133	-1.9397	-0.313208
19.0001	5.0353	5.00395	0.536709	-0.163423
20.	5.09873	4.21601	2.8674	0.018313
20.9999	5.04632	2.33856	4.46388	0.265085
21.9998	4.99438	-0.162735	4.97522	0.405703
22.9998	4.95004	-2.47106	4.24789	0.593429
23.9997	4.99494	-4.30817	2.48043	0.486367
25.0001	5.03957	-5.02672	0.069443	0.352944
26.	5.05326	-4.47765	-2.33558	0.176536
27.	5.08113	-2.88889	-4.17944	-0.067035
27.9999	5.00821	-0.46791	-4.97892	-0.271235
28.9998	5.04196	2.02604	-4.60421	-0.343238
29.9997	5.04851	4.01691	-3.03442	-0.379804
30.9997	5.05625	4.99622	-0.723626	-0.282605
32.0001	5.07133	4.74884	1.77503	-0.126882
33.	5.04574	3.33544	3.78229	0.169385
33.9999	5.02582	1.14788	4.8804	0.350734
34.9998	5.00883	-1.3807	4.78963	0.491441
35.9998	4.99402	-3.53895	3.4848	0.521776
36.9997	5.06318	-4.86009	1.34209	0.462674
37.9996	5.05243	-4.90593	-1.17355	0.285711
39.	5.04583	-3.76246	-3.36139	0.073485
40.	5.0347	-1.75313	-4.71693	-0.158965
40.9999	5.00469	0.775608	-4.9339	-0.319376
41.9998	5.0274	3.05318	-3.97782	-0.360267
42.9997	5.08482	4.67951	-1.96686	-0.298323
43.9997	5.05414	5.02388	0.524856	-0.171811
45.0001	5.08289	4.18112	2.89017	0.031023
46.	5.06485	2.32278	4.49149	0.289673
46.9999	5.01504	-0.106429	4.99479	0.437426
47.9999	4.97359	-2.51844	4.25238	0.55798
48.9998	5.01938	-4.31941	2.49427	0.561707
49.9997	5.03165	-5.01872	0.073452	0.352923

(0,0,2.5) RMF off

```
Data = Import[NotebookDirectory[] <> "230223_x0_y0_z2_5_field_off.txt", "Table"];
       importi··· Notebook-Verzeichnis                              Tabelle
```

```
Bvst = Table[{AbsoluteTime[Data[[i]][[9]]] - AbsoluteTime[Data[[2]][[9]]],
        Tabelle   absolute Zeit seit 1900                absolute Zeit seit 1900
    AbsoluteTime[Data[[i]][[2]]]}, {i, 2, Length[Data]}];
    absolute Zeit seit 1900                    Länge
Bxvst = Table[{AbsoluteTime[Data[[i]][[9]]] - AbsoluteTime[Data[[2]][[9]]],
         Tabelle   absolute Zeit seit 1900                absolute Zeit seit 1900
    AbsoluteTime[Data[[i]][[3]]]}, {i, 2, Length[Data]}];
    absolute Zeit seit 1900                    Länge
Byvst = Table[{AbsoluteTime[Data[[i]][[9]]] - AbsoluteTime[Data[[2]][[9]]],
         Tabelle   absolute Zeit seit 1900                absolute Zeit seit 1900
    AbsoluteTime[Data[[i]][[4]]]}, {i, 2, Length[Data]}];
    absolute Zeit seit 1900                    Länge
Bzvst = Table[{AbsoluteTime[Data[[i]][[9]]] - AbsoluteTime[Data[[2]][[9]]],
         Tabelle   absolute Zeit seit 1900                absolute Zeit seit 1900
    AbsoluteTime[Data[[i]][[5]]]}, {i, 2, Length[Data]}];
    absolute Zeit seit 1900                    Länge
```

```
ListPlot[{Bvst, Bxvst, Byvst, Bzvst}, Joined → True]
listenbezogene Graphik                  verknüpft? wahr
```

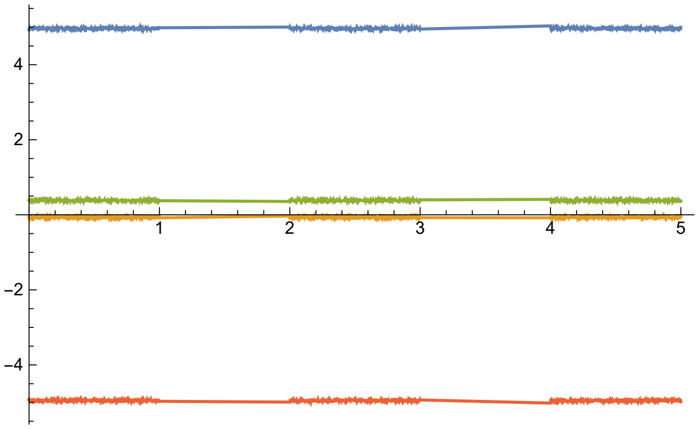


```
Start = 1;
Stop = 1000;
```

```
ListPlot[{Table[{Bvst[[i]][[1]], Bvst[[i]][[2]]}, {i, Start, Stop}],
  Table[{Bxvst[[i]][[1]], Bxvst[[i]][[2]]}, {i, Start, Stop}],
  Table[{Byvst[[i]][[1]], Byvst[[i]][[2]]}, {i, Start, Stop}],
  Table[{Bzvst[[i]][[1]], Bzvst[[i]][[2]]}, {i, Start, Stop}]}, Joined → True]
```

listenbezo··· Tabelle Tabelle Tabelle Tabelle verknüpft? wahr

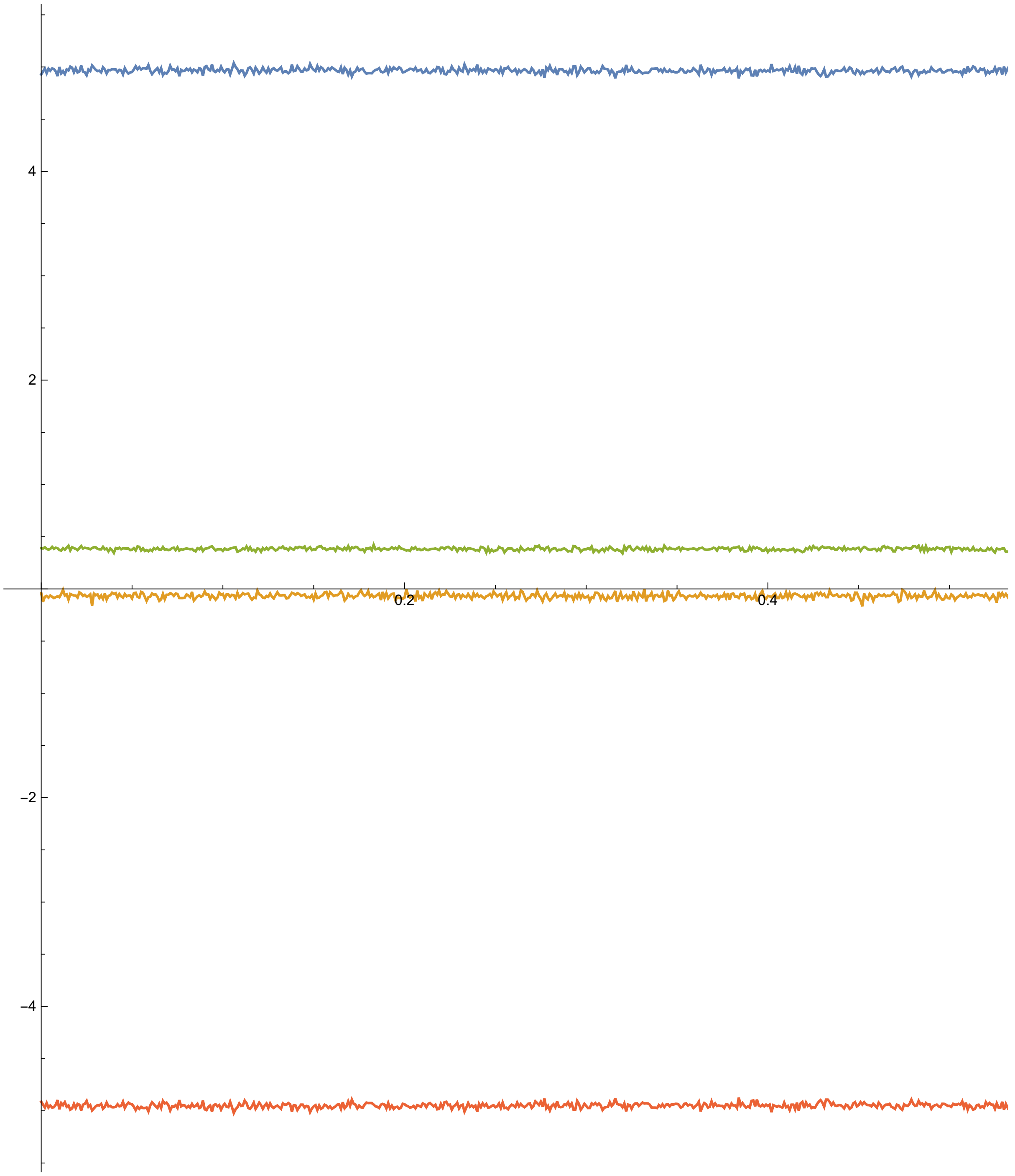

```
ListPlot[{Table[{Bvst[[i]][[1]], Bvst[[i]][[2]]}, {i, Start, Start + 50}],
listenbezo···  Tabelle
  Table[{Bxvst[[i]][[1]], Bxvst[[i]][[2]]}, {i, Start, Start + 50}],
  Tabelle
  Table[{Byvst[[i]][[1]], Byvst[[i]][[2]]}, {i, Start, Start + 50}],
  Tabelle
  Table[{Bzvst[[i]][[1]], Bzvst[[i]][[2]]}, {i, Start, Start + 50}]},
  Tabelle
 Joined → True, PlotLegends → {"B", "Bx", "By", "Bz"}]
 verknüpft? wahr  Legenden der Graphik
```

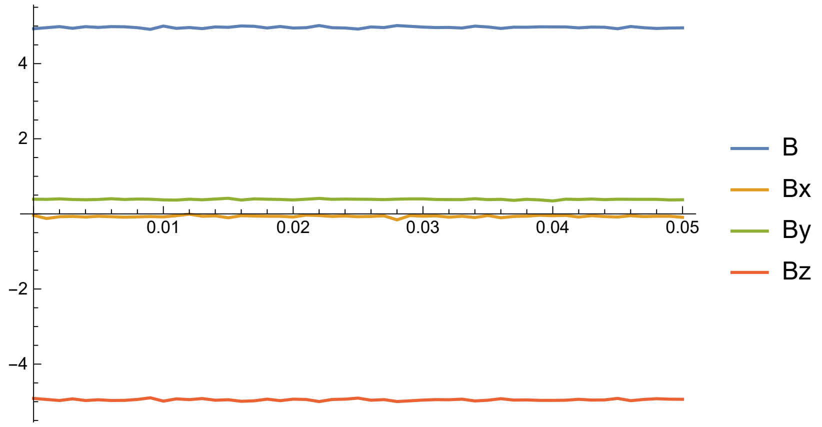


```
TableForm[{Mean[Table[Bvst[[i]][[2]], {i, Start, Stop}]],
Tabellendars···arit···  Tabelle
  Mean[Table[Bxvst[[i]][[2]], {i, Start, Stop}]],
  arit···  Tabelle
  Mean[Table[Byvst[[i]][[2]], {i, Start, Stop}]],
  arit···  Tabelle
  Mean[Table[Bzvst[[i]][[2]], {i, Start, Stop}]]}, TableDirections → Row]
  arit···  Tabelle                                Richtung der Tabellen··· Zeile
```

```
4.96573  -0.0678842  0.381995  -4.95048
```

```
TableForm[Table[{(Bvst[[i]][[1]] - Bvst[[Start]][[1]]) * 1000, Bvst[[i]][[2]],
    Bxvst[[i]][[2]], Byvst[[i]][[2]], Bzvst[[i]][[2]]}, {i, Start, Start + 50}]]
```

Tabellendar·· Tabelle

0.	4.92909	-0.040038	0.390074	-4.91347
0.999928	4.95735	-0.123319	0.387403	-4.94065
1.99986	4.98553	-0.075622	0.398886	-4.96897
2.99978	4.94064	-0.067153	0.383226	-4.92529
3.99971	4.98414	-0.083598	0.37588	-4.96924
4.99964	4.96458	-0.063401	0.383597	-4.94933
6.00004	4.98579	-0.075626	0.402886	-4.96891
6.99997	4.98066	-0.087565	0.383813	-4.96508
7.9999	4.95735	-0.079329	0.394455	-4.941
8.99982	4.91315	-0.070872	0.389793	-4.89715
9.99975	4.99985	-0.079759	0.372129	-4.98534
10.9997	4.93964	-0.048138	0.36725	-4.92573
12.0001	4.9612	-0.00837	0.3906	-4.94579
13.	4.93215	-0.063063	0.375109	-4.91746
13.9999	4.97723	-0.052537	0.394792	-4.96127
14.9999	4.96686	-0.103431	0.414546	-4.94845
15.9998	5.00353	-0.048798	0.368227	-4.98972
16.9997	4.99346	-0.056706	0.399031	-4.97717
17.9996	4.94905	-0.063243	0.390352	-4.93323
19.0001	4.98853	-0.063648	0.383963	-4.97333
20.	4.94797	-0.083223	0.37133	-4.93331
20.9999	4.95706	-0.032327	0.390511	-4.94154
21.9998	5.01421	-0.044925	0.411349	-4.99711
22.9998	4.95686	-0.067322	0.38747	-4.94123
23.9997	4.94929	-0.056248	0.39436	-4.93324
25.0001	4.92115	-0.074954	0.38991	-4.90511
26.	4.97682	-0.067529	0.387775	-4.96123
27.	4.96032	-0.052356	0.379549	-4.94551
27.9999	5.01407	-0.159898	0.391215	-4.99623
28.9998	4.99346	-0.044706	0.399045	-4.97729
29.9997	4.97351	-0.060499	0.398721	-4.95713
30.9997	4.96057	-0.05236	0.383549	-4.94544
32.0001	4.96445	-0.087396	0.379569	-4.94914
33.	4.94836	-0.063232	0.379353	-4.93339
33.9999	4.9979	-0.095749	0.403045	-4.98071
34.9998	4.9763	-0.044522	0.379803	-4.96159
35.9998	4.93714	-0.103114	0.387122	-4.92086
36.9997	4.97115	-0.067459	0.359717	-4.95765
37.9996	4.96881	-0.060447	0.387661	-4.9533
39.	4.97983	-0.036555	0.371875	-4.96579
40.	4.97827	-0.048527	0.344864	-4.96608
40.9999	4.97699	-0.040533	0.390807	-4.96146
41.9998	4.95269	-0.083276	0.38339	-4.93713
42.9997	4.97324	-0.048496	0.394736	-4.95732
43.9997	4.96835	-0.071438	0.379649	-4.95331
45.0001	4.92918	-0.083036	0.390023	-4.91302
46.	4.98877	-0.048653	0.387981	-4.97342
46.9999	4.95687	-0.071322	0.387465	-4.94119
47.9999	4.93688	-0.063117	0.387169	-4.92127
48.9998	4.94788	-0.063224	0.371354	-4.93352
49.9997	4.95226	-0.091268	0.375382	-4.93717

(0,0,2.5) RMF on

```
Data = Import[NotebookDirectory[] <> "230223_x0_y0_z2_5_field_on_5mT.txt", "Table"];
```

importi··· Notebook-Verzeichnis Tabelle

```
Bvst = Table[{AbsoluteTime[Data[[i]][[9]]] - AbsoluteTime[Data[[2]][[9]]],
        Tabelle  absolute Zeit seit 1900                absolute Zeit seit 1900
    AbsoluteTime[Data[[i]][[2]]]}, {i, 2, Length[Data]}];
    absolute Zeit seit 1900                         Länge
Bxvst = Table[{AbsoluteTime[Data[[i]][[9]]] - AbsoluteTime[Data[[2]][[9]]],
         Tabelle  absolute Zeit seit 1900                absolute Zeit seit 1900
    AbsoluteTime[Data[[i]][[3]]]}, {i, 2, Length[Data]}];
    absolute Zeit seit 1900                         Länge
Byvst = Table[{AbsoluteTime[Data[[i]][[9]]] - AbsoluteTime[Data[[2]][[9]]],
         Tabelle  absolute Zeit seit 1900                absolute Zeit seit 1900
    AbsoluteTime[Data[[i]][[4]]]}, {i, 2, Length[Data]}];
    absolute Zeit seit 1900                         Länge
Bzvst = Table[{AbsoluteTime[Data[[i]][[9]]] - AbsoluteTime[Data[[2]][[9]]],
         Tabelle  absolute Zeit seit 1900                absolute Zeit seit 1900
    AbsoluteTime[Data[[i]][[5]]]}, {i, 2, Length[Data]}];
    absolute Zeit seit 1900                         Länge
```

```
ListPlot[{Bvst, Bxvst, Byvst, Bzvst}, Joined → True]
listenbezogene Graphik                    verknüpft? wahr
```

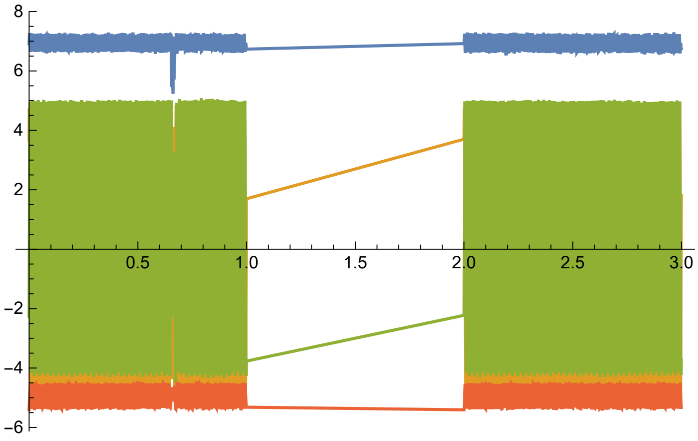


```
Start = 1;
Stop = 1000;
```

```
ListPlot[{Table[{Bvst[[i]][[1]], Bvst[[i]][[2]]}, {i, Start, Stop}],
  Table[{Bxvst[[i]][[1]], Bxvst[[i]][[2]]}, {i, Start, Stop}],
  Table[{Byvst[[i]][[1]], Byvst[[i]][[2]]}, {i, Start, Stop}],
  Table[{Bzvst[[i]][[1]], Bzvst[[i]][[2]]}, {i, Start, Stop}]}, Joined → True]
```

listenbezo··· Tabelle Tabelle Tabelle Tabelle verknüpft? wahr

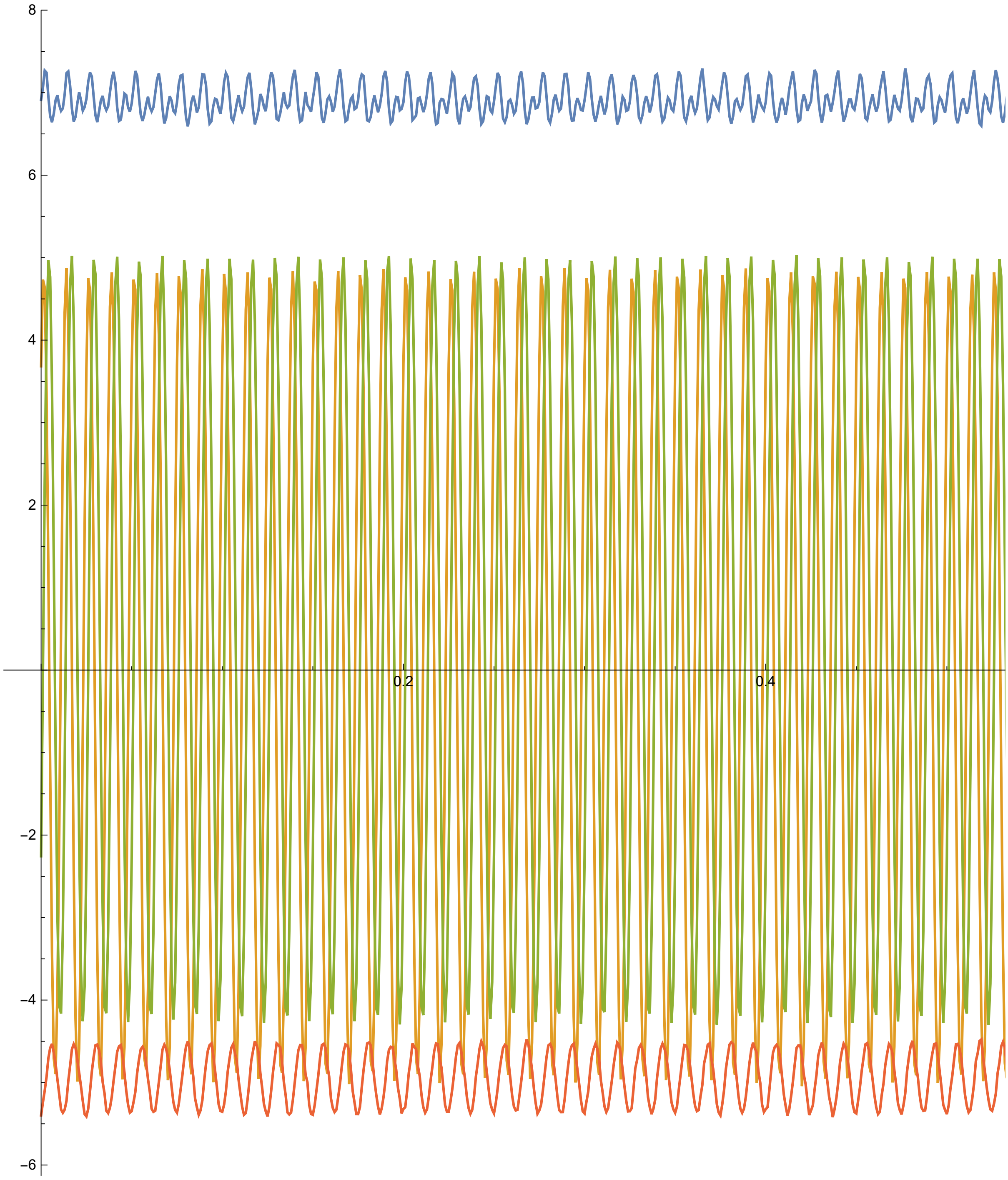

```
ListPlot[{Table[{Bvst[[i]][[1]], Bvst[[i]][[2]]}, {i, Start, Start + 50}],
listenbezo··· Tabelle
  Table[{Bxvst[[i]][[1]], Bxvst[[i]][[2]]}, {i, Start, Start + 50}],
  Tabelle
  Table[{Byvst[[i]][[1]], Byvst[[i]][[2]]}, {i, Start, Start + 50}],
  Tabelle
  Table[{Bzvst[[i]][[1]], Bzvst[[i]][[2]]}, {i, Start, Start + 50}]},
  Tabelle
 Joined → True, PlotLegends → {"B", "Bx", "By", "Bz"}]
 verknüpft? wahr  Legenden der Graphik
```

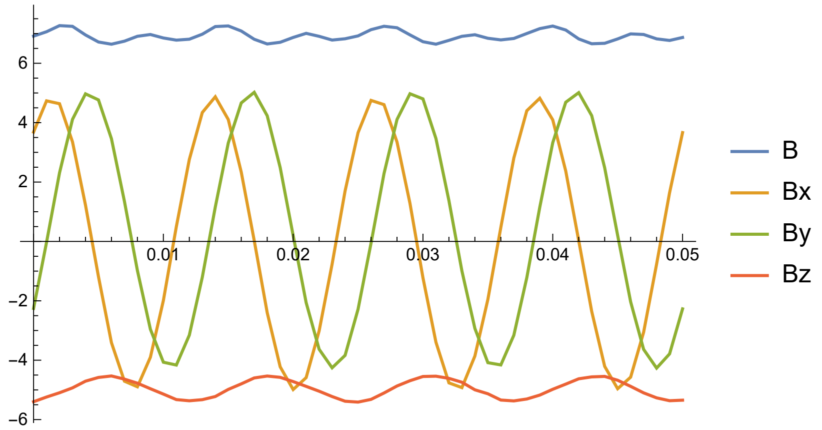


```
TableForm[{Mean[Table[Bvst[[i]][[2]], {i, Start, Stop}]],
Tabellendars···arit··· Tabelle
  Mean[Table[Bxvst[[i]][[2]], {i, Start, Stop}]],
  arit··· Tabelle
  Mean[Table[Byvst[[i]][[2]], {i, Start, Stop}]],
  arit··· Tabelle
  Mean[Table[Bzvst[[i]][[2]], {i, Start, Stop}]]}, TableDirections → Row]
  arit··· Tabelle                                  Richtung der Tabellen···Zeile
```

6.9006 −0.0707487 0.375529 −4.94659

```
TableForm[Table[{(Bvst[[i]][[1]] - Bvst[[Start]][[1]]) * 1000, Bvst[[i]][[2]],
    Bxvst[[i]][[2]], Byvst[[i]][[2]], Bzvst[[i]][[2]]}, {i, Start, Start + 50}]]
```

0.	6.91363	3.68331	-2.256	-5.39833
1.0004	7.06216	4.73435	-0.029071	-5.24014
2.00033	7.26778	4.63805	2.30991	-5.09642
3.00026	7.24323	3.35154	4.11228	-4.93161
4.00019	6.95394	1.22373	4.97075	-4.70653
5.00011	6.71606	-1.18487	4.76633	-4.5808
6.00004	6.64187	-3.40992	3.45812	-4.53082
6.99997	6.74428	-4.70659	1.35117	-4.63764
8.00037	6.90854	-4.89624	-0.98021	-4.7743
9.0003	6.96884	-3.89976	-2.95757	-4.96079
10.0002	6.85156	-1.9902	-4.06797	-5.14146
11.0002	6.77911	0.49814	-4.16359	-5.32661
12.0001	6.81024	2.75966	-3.15654	-5.36655
13.	6.97875	4.34181	-1.20526	-5.32907
14.0004	7.23636	4.87212	1.15895	-5.22342
15.0003	7.2574	4.106	3.31275	-4.9836
16.0003	7.08704	2.33521	4.66425	-4.79768
17.0002	6.80854	0.029724	5.02186	-4.59742
18.0001	6.65033	-2.39881	4.23329	-4.53341
19.0001	6.70649	-4.22329	2.48788	-4.57725
20.	6.86969	-4.98808	0.198994	-4.71934
21.0004	7.00787	-4.58704	-2.06039	-4.88099
22.0003	6.90757	-3.01377	-3.63146	-5.04423
23.0002	6.78167	-0.763761	-4.25647	-5.224
24.0002	6.82519	1.70025	-3.83747	-5.38203
25.0001	6.92184	3.66422	-2.28283	-5.41055
26.	7.13175	4.74957	-0.058843	-5.31977
27.	7.24608	4.607	2.286	-5.10446
28.0004	7.19612	3.3482	4.10731	-4.86864
29.0003	6.95561	1.27988	4.97357	-4.69106
30.0002	6.72602	-1.22458	4.8008	-4.54885
31.0001	6.64295	-3.39401	3.46526	-4.53888
32.0001	6.77104	-4.76335	1.37472	-4.61169
33.	6.90787	-4.9239	-0.992731	-4.7422
34.0004	6.96085	-3.86016	-2.92997	-4.99678
35.0003	6.84161	-1.95406	-4.08311	-5.13006
36.0003	6.78713	0.473973	-4.15938	-5.34229
37.0002	6.83519	2.80762	-3.16042	-5.37111
38.0001	7.00223	4.40206	-1.22053	-5.30692
39.	7.16617	4.82163	1.15116	-5.17501
40.	7.25313	4.09403	3.32468	-4.9793
41.0004	7.1196	2.3711	4.68741	-4.8057
42.0003	6.81973	0.025437	5.0103	-4.62656
43.0002	6.65697	-2.35511	4.23777	-4.5618
44.0001	6.67241	-4.20796	2.48041	-4.54552
45.0001	6.8198	-4.96063	0.202354	-4.67556
46.	6.98935	-4.57107	-2.03337	-4.88075
47.0004	6.96939	-3.05834	-3.63466	-5.09983
48.0003	6.82225	-0.764203	-4.2668	-5.26816
49.0003	6.7683	1.64036	-3.78679	-5.36464
50.0002	6.86848	3.66486	-2.27181	-5.34638

(0,0,5) RMF off

```
Data = Import[NotebookDirectory[] <> "230223_x0_y0_z5_field_off.txt", "Table"];
```

```
Bvst = Table[{AbsoluteTime[Data[[i]][[9]]] - AbsoluteTime[Data[[2]][[9]]],
        Tabelle   absolute Zeit seit 1900                absolute Zeit seit 1900
    AbsoluteTime[Data[[i]][[2]]]}, {i, 2, Length[Data]}];
    absolute Zeit seit 1900                      Länge
Bxvst = Table[{AbsoluteTime[Data[[i]][[9]]] - AbsoluteTime[Data[[2]][[9]]],
        Tabelle   absolute Zeit seit 1900                absolute Zeit seit 1900
    AbsoluteTime[Data[[i]][[3]]]}, {i, 2, Length[Data]}];
    absolute Zeit seit 1900                      Länge
Byvst = Table[{AbsoluteTime[Data[[i]][[9]]] - AbsoluteTime[Data[[2]][[9]]],
        Tabelle   absolute Zeit seit 1900                absolute Zeit seit 1900
    AbsoluteTime[Data[[i]][[4]]]}, {i, 2, Length[Data]}];
    absolute Zeit seit 1900                      Länge
Bzvst = Table[{AbsoluteTime[Data[[i]][[9]]] - AbsoluteTime[Data[[2]][[9]]],
        Tabelle   absolute Zeit seit 1900                absolute Zeit seit 1900
    AbsoluteTime[Data[[i]][[5]]]}, {i, 2, Length[Data]}];
    absolute Zeit seit 1900                      Länge
```

```
ListPlot[{Bvst, Bxvst, Byvst, Bzvst}, Joined → True]
listenbezogene Graphik                     verknüpft? wahr
```

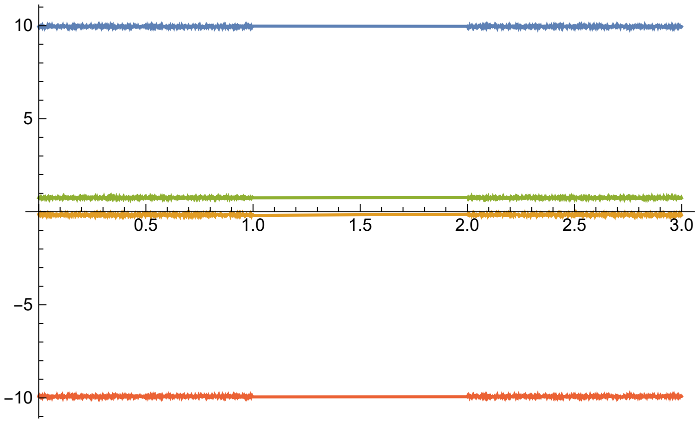


```
Start = 1;
Stop = 1000;
```

```
ListPlot[{Table[{Bvst[[i]][[1]], Bvst[[i]][[2]]}, {i, Start, Stop}],
 listenbezo···  Tabelle
   Table[{Bxvst[[i]][[1]], Bxvst[[i]][[2]]}, {i, Start, Stop}],
   Tabelle
   Table[{Byvst[[i]][[1]], Byvst[[i]][[2]]}, {i, Start, Stop}],
   Tabelle
   Table[{Bzvst[[i]][[1]], Bzvst[[i]][[2]]}, {i, Start, Stop}]}, Joined → True]
   Tabelle                                                        verknüpft?  wahr
```

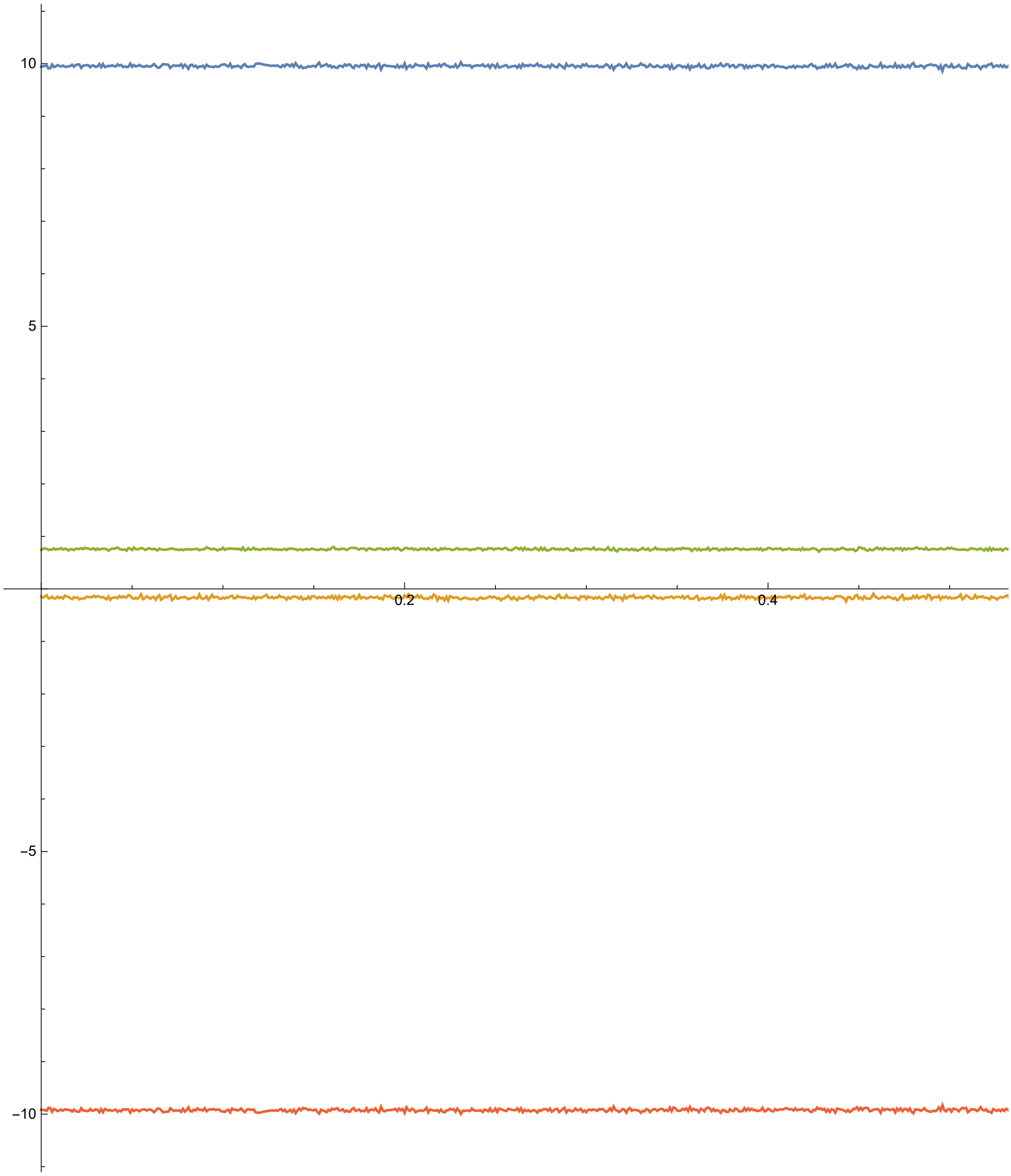

```
ListPlot[{Table[{Bvst[[i]][[1]], Bvst[[i]][[2]]}, {i, Start, Start + 50}],
 listenbezo⋯  Tabelle
  Table[{Bxvst[[i]][[1]], Bxvst[[i]][[2]]}, {i, Start, Start + 50}],
  Tabelle
  Table[{Byvst[[i]][[1]], Byvst[[i]][[2]]}, {i, Start, Start + 50}],
  Tabelle
  Table[{Bzvst[[i]][[1]], Bzvst[[i]][[2]]}, {i, Start, Start + 50}]},
  Tabelle
 Joined → True, PlotLegends → {"B", "Bx", "By", "Bz"}]
 verknüpft? wahr  Legenden der Graphik
```

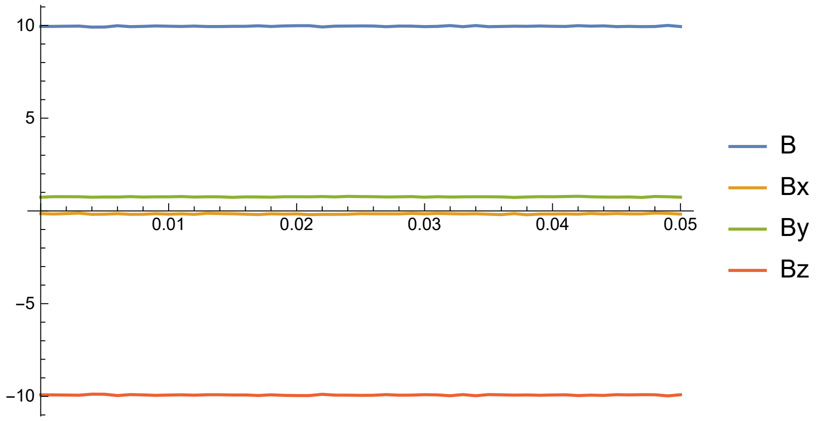


```
TableForm[{Mean[Table[Bvst[[i]][[2]], {i, Start, Stop}]],
 Tabellendars⋯ arit⋯  Tabelle
  Mean[Table[Bxvst[[i]][[2]], {i, Start, Stop}]],
  arit⋯  Tabelle
  Mean[Table[Byvst[[i]][[2]], {i, Start, Stop}]],
  arit⋯  Tabelle
  Mean[Table[Bzvst[[i]][[2]], {i, Start, Stop}]]}, TableDirections → Row]
  arit⋯  Tabelle                                    Richtung der Tabellen⋯ Zeile
```

9.95281 -0.164883 0.756619 -9.9226

```
TableForm[Table[{(Bvst[[i]][[1]] - Bvst[[Start]][[1]]) * 1000, Bvst[[i]][[2]],
  Bxvst[[i]][[2]], Byvst[[i]][[2]], Bzvst[[i]][[2]]}, {i, Start, Start + 50}]]
```
Tabellendar·· Tabelle

0.	9.94454	-0.143949	0.740437	-9.91589
0.999928	9.9503	-0.168016	0.767466	-9.91923
1.99986	9.95816	-0.1441	0.767617	-9.92748
3.00026	9.96582	-0.12018	0.763768	-9.93579
4.00019	9.90985	-0.183586	0.739855	-9.88049
5.00011	9.9145	-0.17564	0.751924	-9.88439
6.00004	9.98497	-0.148369	0.749042	-9.95572
6.99997	9.9347	-0.183855	0.771202	-9.90301
7.9999	9.94923	-0.179996	0.748454	-9.9194
9.0003	9.97371	-0.148258	0.760857	-9.94354
10.0002	9.95791	-0.176091	0.760579	-9.92726
11.0002	9.94673	-0.155984	0.775418	-9.91524
12.0001	9.96523	-0.184161	0.748693	-9.93536
13.	9.94189	-0.127932	0.763392	-9.91171
13.9999	9.94176	-0.143928	0.760373	-9.91159
14.9999	9.9521	-0.152023	0.73255	-9.92393
16.0003	9.95367	-0.176046	0.756519	-9.92332
17.0002	9.98154	-0.195329	0.752924	-9.95118
18.0001	9.9446	-0.155948	0.740422	-9.91577
19.0001	9.97403	-0.17226	0.763828	-9.94325
20.	9.98593	-0.160384	0.764026	-9.95537
20.9999	9.98605	-0.199379	0.76098	-9.95502
22.0003	9.91998	-0.183704	0.774973	-9.88795
23.0002	9.96172	-0.184128	0.756631	-9.93124
24.0002	9.96736	-0.180197	0.783694	-9.93486
25.0001	9.97439	-0.148269	0.771856	-9.94338
26.	9.97019	-0.156223	0.767786	-9.93935
27.	9.93334	-0.155837	0.752238	-9.90359
27.9999	9.96579	-0.160175	0.760721	-9.93542
29.0003	9.96235	-0.132146	0.771691	-9.93154
30.0002	9.93661	-0.155866	0.7403	-9.90777
31.0001	9.95014	-0.136018	0.767504	-9.91956
32.0001	9.99295	-0.148452	0.749164	-9.96372
33.	9.93387	-0.163844	0.760227	-9.90339
33.9999	9.99388	-0.152467	0.764157	-9.96345
34.9998	9.93414	-0.175847	0.763213	-9.90322
36.0003	9.94588	-0.198962	0.756369	-9.91508
37.0002	9.95981	-0.144101	0.728682	-9.93208
38.0001	9.95371	-0.20704	0.752482	-9.92306
39.	9.97026	-0.168223	0.767771	-9.93923
40.	9.95404	-0.168054	0.763527	-9.9233
40.9999	9.94676	-0.159984	0.775413	-9.9152
42.0003	9.98754	-0.176408	0.788004	-9.95484
43.0002	9.96187	-0.136138	0.763688	-9.93162
44.0001	9.97706	-0.164286	0.7489	-9.94756
45.0001	9.93678	-0.139871	0.744319	-9.90788
46.	9.94933	-0.160001	0.752477	-9.91955
46.9999	9.93593	-0.159853	0.728297	-9.90792
47.9999	9.94286	-0.115949	0.779404	-9.91159
49.0003	10.0018	-0.13255	0.764303	-9.97166
50.0002	9.94071	-0.171906	0.740342	-9.91161

(0,0,5) RMF on

```
Data = Import[NotebookDirectory[] <> "230223_x0_y0_z5_5mT_on.txt", "Table"];
```
importi··· Notebook-Verzeichnis Tabelle

```
Bvst = Table[{AbsoluteTime[Data[[i]][[9]]] - AbsoluteTime[Data[[2]][[9]]],
         Tabelle  absolute Zeit seit 1900                absolute Zeit seit 1900
     AbsoluteTime[Data[[i]][[2]]]}, {i, 2, Length[Data]}];
     absolute Zeit seit 1900                             Länge
Bxvst = Table[{AbsoluteTime[Data[[i]][[9]]] - AbsoluteTime[Data[[2]][[9]]],
          Tabelle  absolute Zeit seit 1900                absolute Zeit seit 1900
     AbsoluteTime[Data[[i]][[3]]]}, {i, 2, Length[Data]}];
     absolute Zeit seit 1900                             Länge
Byvst = Table[{AbsoluteTime[Data[[i]][[9]]] - AbsoluteTime[Data[[2]][[9]]],
          Tabelle  absolute Zeit seit 1900                absolute Zeit seit 1900
     AbsoluteTime[Data[[i]][[4]]]}, {i, 2, Length[Data]}];
     absolute Zeit seit 1900                             Länge
Bzvst = Table[{AbsoluteTime[Data[[i]][[9]]] - AbsoluteTime[Data[[2]][[9]]],
          Tabelle  absolute Zeit seit 1900                absolute Zeit seit 1900
     AbsoluteTime[Data[[i]][[5]]]}, {i, 2, Length[Data]}];
     absolute Zeit seit 1900                             Länge
```

```
ListPlot[{Bvst, Bxvst, Byvst, Bzvst}, Joined → True]
listenbezogene Graphik                   verknüpft? wahr
```

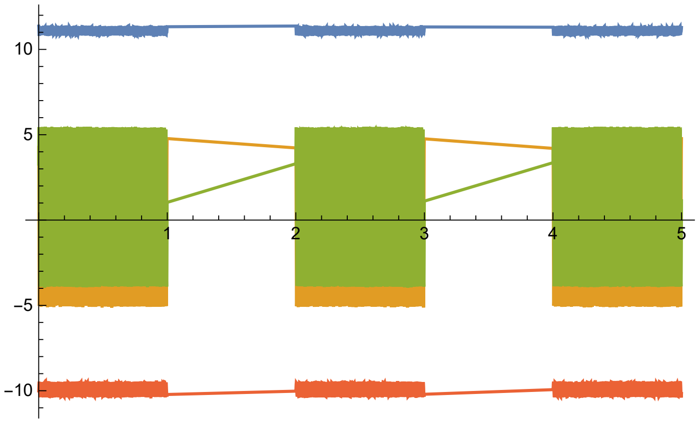


```
Start = 1;
Stop = 1000;
```

```
ListPlot[{Table[{Bvst[[i]][[1]], Bvst[[i]][[2]]}, {i, Start, Stop}],
listenbezo… Tabelle
  Table[{Bxvst[[i]][[1]], Bxvst[[i]][[2]]}, {i, Start, Stop}],
  Tabelle
  Table[{Byvst[[i]][[1]], Byvst[[i]][[2]]}, {i, Start, Stop}],
  Tabelle
  Table[{Bzvst[[i]][[1]], Bzvst[[i]][[2]]}, {i, Start, Stop}]}, Joined → True]
  Tabelle                                                       verknüpft? wahr
```

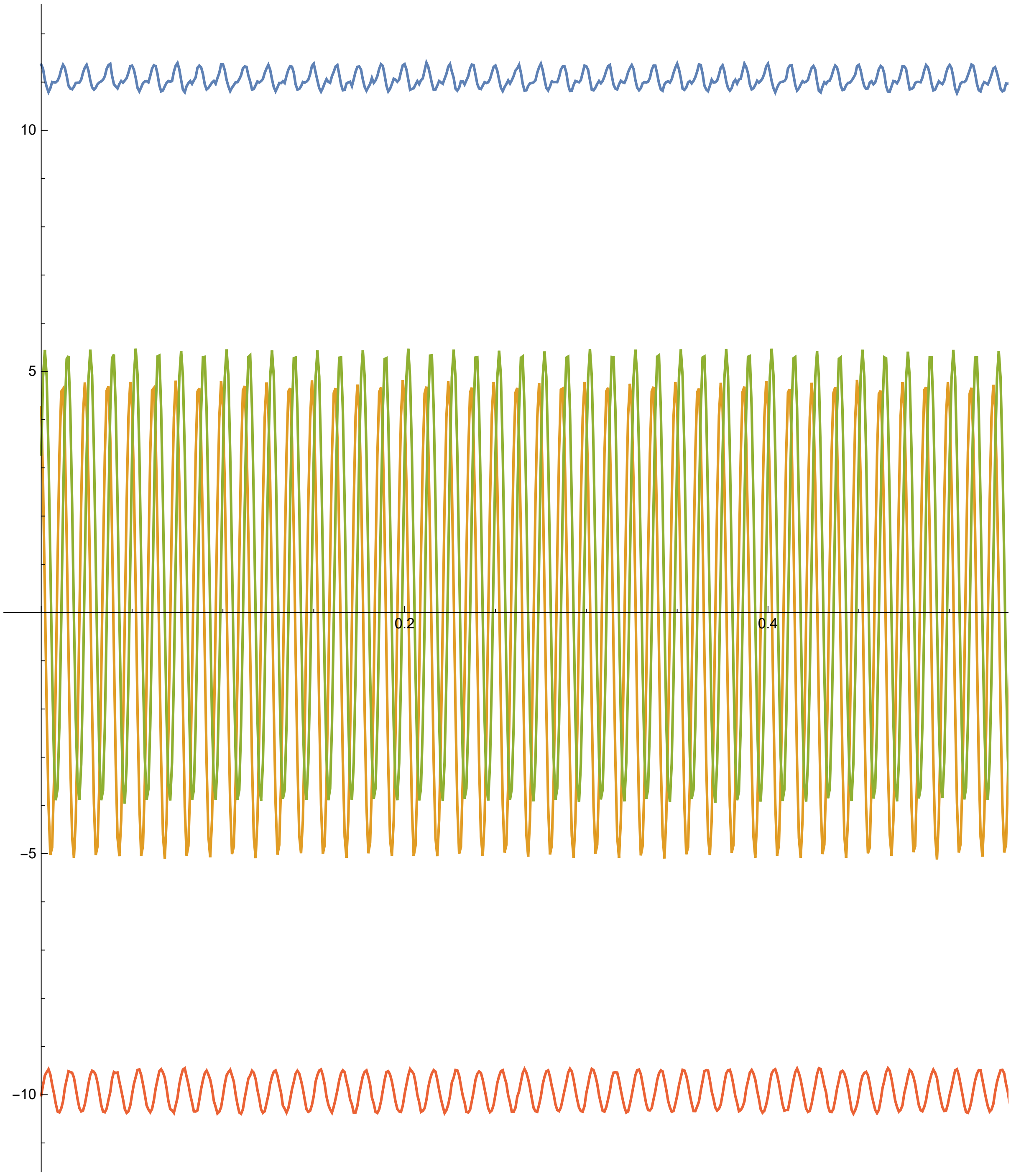

```
ListPlot[{Table[{Bvst[[i]][[1]], Bvst[[i]][[2]]}, {i, Start, Start + 50}],
listenbezo⋯ Tabelle
  Table[{Bxvst[[i]][[1]], Bxvst[[i]][[2]]}, {i, Start, Start + 50}],
  Tabelle
  Table[{Byvst[[i]][[1]], Byvst[[i]][[2]]}, {i, Start, Start + 50}],
  Tabelle
  Table[{Bzvst[[i]][[1]], Bzvst[[i]][[2]]}, {i, Start, Start + 50}]},
  Tabelle
 Joined → True, PlotLegends → {"B", "Bx", "By", "Bz"}]
 verknüpft? wahr  Legenden der Graphik
```

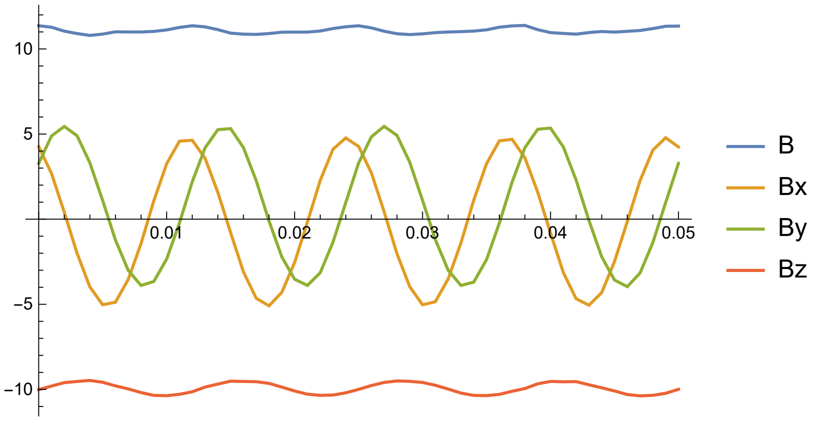


```
TableForm[{Mean[Table[Bvst[[i]][[2]], {i, Start, Stop}]],
Tabellendars⋯arit⋯ Tabelle
  Mean[Table[Bxvst[[i]][[2]], {i, Start, Stop}]],
  arit⋯ Tabelle
  Mean[Table[Byvst[[i]][[2]], {i, Start, Stop}]],
  arit⋯ Tabelle
  Mean[Table[Bzvst[[i]][[2]], {i, Start, Stop}]]}, TableDirections → Row]
  arit⋯ Tabelle                                    Richtung der Tabellen⋯ Zeile
```

```
11.0614  -0.162519  0.752233  -9.92123
```

```
TableForm[Table[{(Bvst[[i]][[1]] - Bvst[[Start]][[1]]) * 1000, Bvst[[i]][[2]],
    Bxvst[[i]][[2]], Byvst[[i]][[2]], Bzvst[[i]][[2]]}, {i, Start, Start + 50}]]
```
Tabellendar·· Tabelle

0.	11.3617	4.25931	3.2797	-10.0095
0.999928	11.2756	2.68346	4.88509	-9.80175
1.99986	11.0445	0.401753	5.44672	-9.59962
2.99978	10.9028	-2.00702	4.89913	-9.53113
3.99971	10.7926	-3.97862	3.309	-9.47107
4.99964	10.8718	-5.02516	1.09729	-9.57807
6.00004	11.004	-4.87356	-1.2286	-9.78917
6.99997	10.9958	-3.53125	-3.00566	-9.97017
7.9999	10.9951	-1.42828	-3.89234	-10.1834
8.99982	11.0294	1.08788	-3.6562	-10.3488
9.99975	11.1159	3.25725	-2.34053	-10.3671
10.9997	11.2623	4.58264	-0.222168	-10.2854
12.0001	11.3614	4.63731	2.19491	-10.137
13.	11.2962	3.58072	4.18685	-9.86167
13.9999	11.1326	1.57616	5.25722	-9.68563
14.9999	10.9281	-0.792271	5.31915	-9.51324
15.9998	10.8671	-3.09425	4.20592	-9.53051
16.9997	10.8522	-4.64517	2.25831	-9.54425
17.9996	10.904	-5.08741	-0.108906	-9.64385
19.0001	10.9834	-4.29711	-2.215	-9.86219
20.	10.9882	-2.54282	-3.52685	-10.0913
20.9999	10.9892	-0.174167	-3.88963	-10.2763
21.9998	11.0517	2.27836	-3.14695	-10.3463
22.9998	11.1973	4.1065	-1.34711	-10.3297
23.9997	11.3013	4.77213	0.984817	-10.1969
24.9996	11.3599	4.27142	3.29453	-9.99744
26.	11.237	2.71984	4.83765	-9.77084
27.	11.0385	0.425868	5.45356	-9.58776
27.9999	10.8917	-2.02676	4.92168	-9.50258
28.9998	10.8435	-3.96719	3.3408	-9.52271
29.9997	10.8846	-5.02928	1.10147	-9.58996
30.9997	10.9576	-4.84623	-1.2011	-9.75403
32.0001	10.9937	-3.51134	-2.99451	-9.97821
33.	11.0162	-1.38857	-3.89187	-10.2118
33.9999	11.0464	1.11592	-3.69616	-10.3497
34.9998	11.1216	3.26132	-2.37658	-10.3637
35.9998	11.2745	4.60261	-0.230082	-10.2897
36.9997	11.3533	4.68864	2.18349	-10.1067
37.9996	11.3805	3.59984	4.20015	-9.94568
39.	11.131	1.5883	5.27999	-9.66941
40.	10.9581	-0.812467	5.35037	-9.52856
40.9999	10.9094	-3.1305	4.24518	-9.54954
41.9998	10.8681	-4.67316	2.29721	-9.53937
42.9997	10.9612	-5.0533	-0.068602	-9.7266
43.9997	11.0239	-4.31353	-2.20741	-9.90191
45.0001	10.9882	-2.48773	-3.57384	-10.0886
46.	11.035	-0.142299	-3.96328	-10.2978
46.9999	11.0824	2.27006	-3.15849	-10.3774
47.9999	11.1962	4.05937	-1.36694	-10.3445
48.9998	11.3314	4.78385	0.981259	-10.2251
49.9997	11.3419	4.24448	3.27943	-9.99339

(0,0,7.5) RMF off

```
Data = Import[NotebookDirectory[] <> "230223_x0_y0_z7_5_field_off.txt", "Table"];
```
importi··· Notebook-Verzeichnis Tabelle

```
Bvst = Table[{AbsoluteTime[Data[[i]][[9]]] - AbsoluteTime[Data[[2]][[9]]],
        Tabelle  absolute Zeit seit 1900                  absolute Zeit seit 1900
    AbsoluteTime[Data[[i]][[2]]]}, {i, 2, Length[Data]}];
    absolute Zeit seit 1900                       Länge
Bxvst = Table[{AbsoluteTime[Data[[i]][[9]]] - AbsoluteTime[Data[[2]][[9]]],
         Tabelle  absolute Zeit seit 1900                  absolute Zeit seit 1900
    AbsoluteTime[Data[[i]][[3]]]}, {i, 2, Length[Data]}];
    absolute Zeit seit 1900                       Länge
Byvst = Table[{AbsoluteTime[Data[[i]][[9]]] - AbsoluteTime[Data[[2]][[9]]],
         Tabelle  absolute Zeit seit 1900                  absolute Zeit seit 1900
    AbsoluteTime[Data[[i]][[4]]]}, {i, 2, Length[Data]}];
    absolute Zeit seit 1900                       Länge
Bzvst = Table[{AbsoluteTime[Data[[i]][[9]]] - AbsoluteTime[Data[[2]][[9]]],
         Tabelle  absolute Zeit seit 1900                  absolute Zeit seit 1900
    AbsoluteTime[Data[[i]][[5]]]}, {i, 2, Length[Data]}];
    absolute Zeit seit 1900                       Länge
```

```
ListPlot[{Bvst, Bxvst, Byvst, Bzvst}, Joined → True]
listenbezogene Graphik                    verknüpft? wahr
```

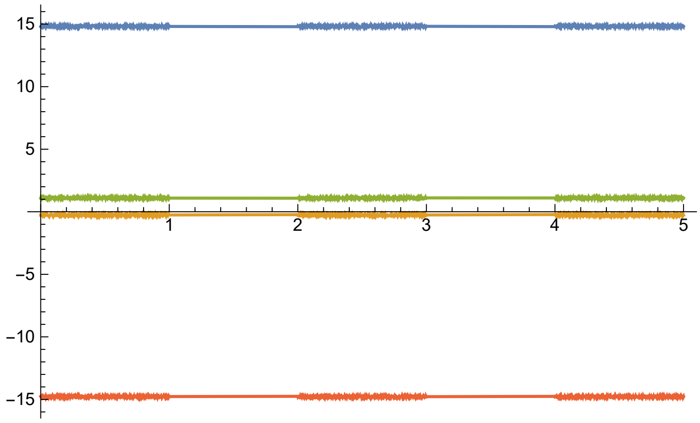


```
Start = 1;
Stop = 1000;
```

```
ListPlot[{Table[{Bvst[[i]][[1]], Bvst[[i]][[2]]}, {i, Start, Stop}],
listenbezo··· Tabelle
  Table[{Bxvst[[i]][[1]], Bxvst[[i]][[2]]}, {i, Start, Stop}],
  Tabelle
  Table[{Byvst[[i]][[1]], Byvst[[i]][[2]]}, {i, Start, Stop}],
  Tabelle
  Table[{Bzvst[[i]][[1]], Bzvst[[i]][[2]]}, {i, Start, Stop}]}, Joined → True]
  Tabelle                                                        verknüpft? wahr
```

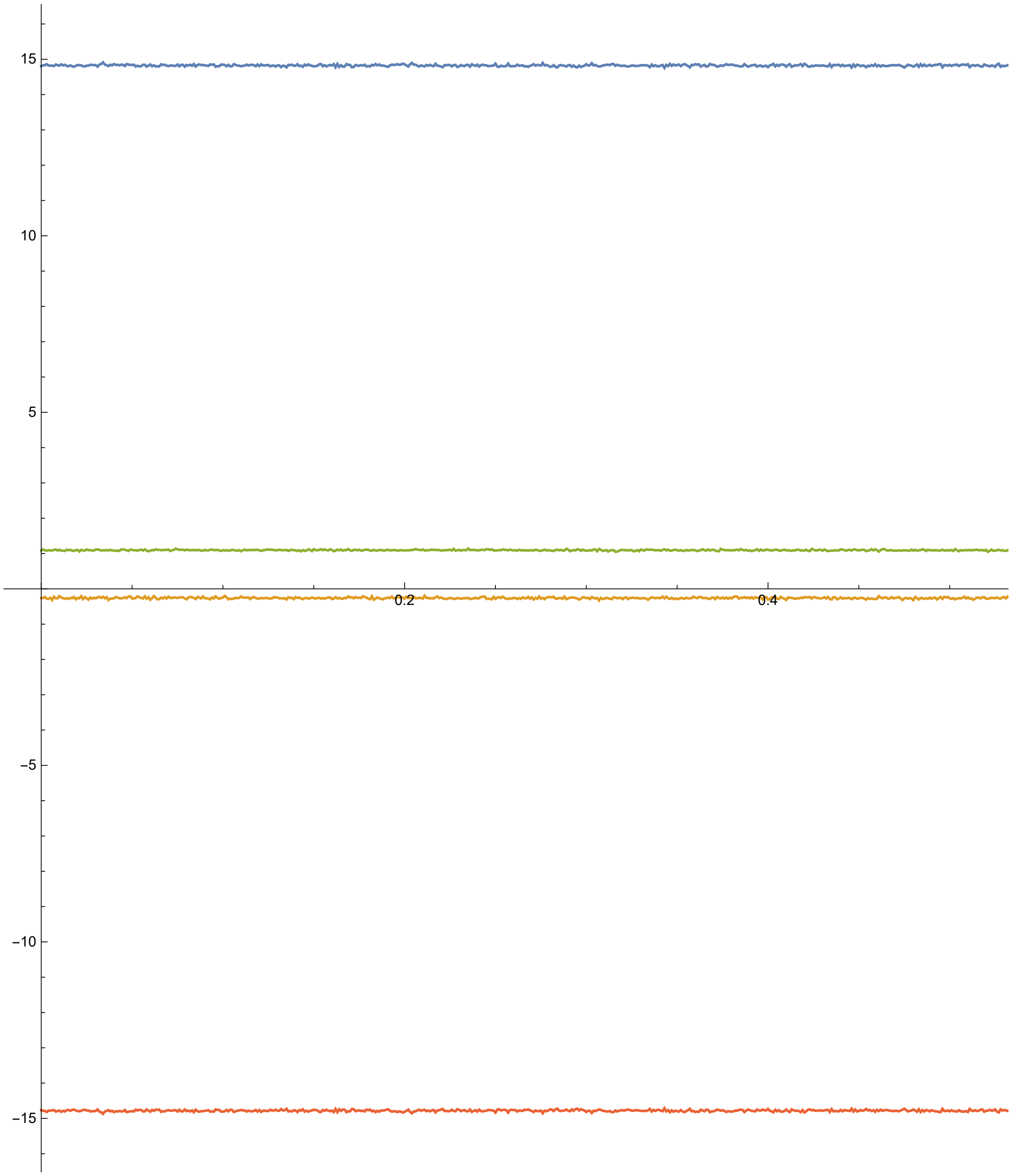

```
ListPlot[{Table[{Bvst[[i]][[1]], Bvst[[i]][[2]]}, {i, Start, Start + 50}],
 listenbezo⋯  Tabelle
  Table[{Bxvst[[i]][[1]], Bxvst[[i]][[2]]}, {i, Start, Start + 50}],
  Tabelle
  Table[{Byvst[[i]][[1]], Byvst[[i]][[2]]}, {i, Start, Start + 50}],
  Tabelle
  Table[{Bzvst[[i]][[1]], Bzvst[[i]][[2]]}, {i, Start, Start + 50}]},
  Tabelle
 Joined → True, PlotLegends → {"B", "Bx", "By", "Bz"}]
 verknüpft? wahr  Legenden der Graphik
```

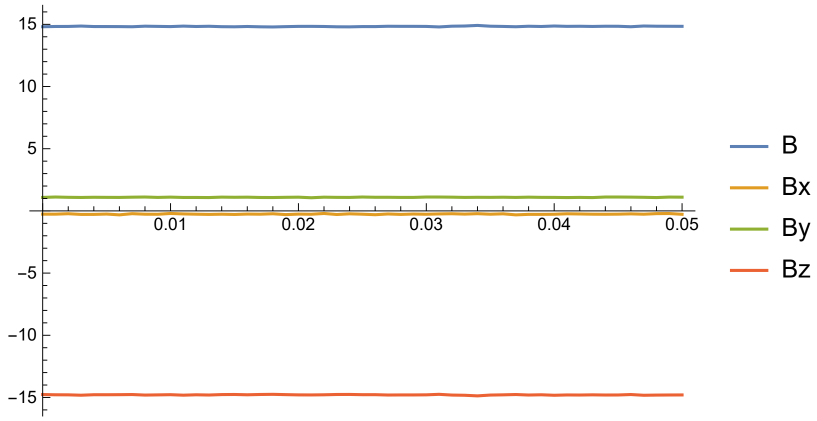


```
TableForm[{Mean[Table[Bvst[[i]][[2]], {i, Start, Stop}]],
Tabellendars⋯ arit⋯  Tabelle
  Mean[Table[Bxvst[[i]][[2]], {i, Start, Stop}]],
  arit⋯  Tabelle
  Mean[Table[Byvst[[i]][[2]], {i, Start, Stop}]],
  arit⋯  Tabelle
  Mean[Table[Bzvst[[i]][[2]], {i, Start, Stop}]]}, TableDirections → Row]
  arit⋯  Tabelle                                 Richtung der Tabellen⋯ Zeile
```

14.8239 −0.260618 1.0952 −14.7811

```
TableForm[Table[{(Bvst[[i]][[1]] - Bvst[[Start]][[1]]) * 1000, Bvst[[i]][[2]],
Tabellendar·· Tabelle
   Bxvst[[i]][[2]], Byvst[[i]][[2]], Bzvst[[i]][[2]]}, {i, Start, Start + 50}]]
```

0.	14.8063	-0.261266	1.08443	-14.7643
0.999928	14.8281	-0.261505	1.11573	-14.7838
1.99986	14.8305	-0.226524	1.09284	-14.7885
2.99978	14.8612	-0.281828	1.08125	-14.8191
4.00019	14.8231	-0.281442	1.09565	-14.7799
5.00011	14.8225	-0.254436	1.08868	-14.7803
6.00004	14.8188	-0.317387	1.08455	-14.7757
6.99997	14.8072	-0.222288	1.10348	-14.7644
7.9999	14.8511	-0.265741	1.11608	-14.8067
8.99982	14.8343	-0.269555	1.08485	-14.7922
9.99975	14.8194	-0.206417	1.10768	-14.7765
11.0002	14.8569	-0.242789	1.08123	-14.8155
12.0001	14.8261	-0.265468	1.08073	-14.7843
13.	14.8447	-0.281655	1.073	-14.8032
13.9999	14.8117	-0.265331	1.10748	-14.7679
14.9999	14.8032	-0.285235	1.09534	-14.7598
15.9998	14.8274	-0.254493	1.10374	-14.784
17.0002	14.8021	-0.265221	1.08037	-14.7603
18.0001	14.7897	-0.230095	1.07622	-14.7487
19.0001	14.8151	-0.297355	1.09251	-14.7718
20.	14.8354	-0.257575	1.10386	-14.792
20.9999	14.8368	-0.269568	1.05791	-14.7966
21.9998	14.8271	-0.206495	1.1038	-14.7845
22.9998	14.8028	-0.289228	1.08834	-14.7599
24.0002	14.7982	-0.234185	1.08434	-14.7565
25.0001	14.8202	-0.265422	1.11561	-14.7757
26.	14.8193	-0.305399	1.09556	-14.7756
27.	14.8458	-0.246681	1.09604	-14.8032
27.9999	14.8384	-0.281595	1.0849	-14.796
28.9998	14.8382	-0.250597	1.08493	-14.7964
30.0002	14.8322	-0.269545	1.11578	-14.7877
31.0001	14.7921	-0.242134	1.11521	-14.748
32.0001	14.8502	-0.226731	1.10413	-14.8073
33.	14.8652	-0.257875	1.08534	-14.8233
33.9999	14.9134	-0.22338	1.09411	-14.8715
34.9998	14.8495	-0.265714	1.08908	-14.8071
35.9998	14.831	-0.234531	1.09983	-14.7883
37.0002	14.8068	-0.317263	1.08436	-14.7637
38.0001	14.8465	-0.277687	1.10401	-14.8028
39.	14.8267	-0.277476	1.08872	-14.784
40.	14.8653	-0.269874	1.08532	-14.8232
40.9999	14.8374	-0.230586	1.07296	-14.7968
41.9998	14.8452	-0.246669	1.08504	-14.8034
43.0002	14.8296	-0.265501	1.07279	-14.7884
44.0001	14.8472	-0.2697	1.11601	-14.8027
45.0001	14.8441	-0.261669	1.11598	-14.7998
46.	14.8075	-0.238291	1.10746	-14.7641
46.9999	14.8657	-0.265883	1.09333	-14.8231
47.9999	14.8483	-0.2187	1.07314	-14.8079
48.9998	14.8436	-0.210668	1.11204	-14.8004
50.0002	14.8395	-0.273615	1.1039	-14.7958

(0,0,7.5) RMF on

```
Data = Import[NotebookDirectory[] <> "230223_x0_y0_z7_5_field_on_5mT.txt", "Table"];
        importi··· Notebook-Verzeichnis                                            Tabelle
```

```
Bvst = Table[{AbsoluteTime[Data[[i]][[9]]] - AbsoluteTime[Data[[2]][[9]]],
        Tabelle  absolute Zeit seit 1900              absolute Zeit seit 1900
    AbsoluteTime[Data[[i]][[2]]]}, {i, 2, Length[Data]}];
    absolute Zeit seit 1900                     Länge
Bxvst = Table[{AbsoluteTime[Data[[i]][[9]]] - AbsoluteTime[Data[[2]][[9]]],
        Tabelle  absolute Zeit seit 1900              absolute Zeit seit 1900
    AbsoluteTime[Data[[i]][[3]]]}, {i, 2, Length[Data]}];
    absolute Zeit seit 1900                     Länge
Byvst = Table[{AbsoluteTime[Data[[i]][[9]]] - AbsoluteTime[Data[[2]][[9]]],
        Tabelle  absolute Zeit seit 1900              absolute Zeit seit 1900
    AbsoluteTime[Data[[i]][[4]]]}, {i, 2, Length[Data]}];
    absolute Zeit seit 1900                     Länge
Bzvst = Table[{AbsoluteTime[Data[[i]][[9]]] - AbsoluteTime[Data[[2]][[9]]],
        Tabelle  absolute Zeit seit 1900              absolute Zeit seit 1900
    AbsoluteTime[Data[[i]][[5]]]}, {i, 2, Length[Data]}];
    absolute Zeit seit 1900                     Länge
```

```
ListPlot[{Bvst, Bxvst, Byvst, Bzvst}, Joined → True]
listenbezogene Graphik                    verknüpft? wahr
```

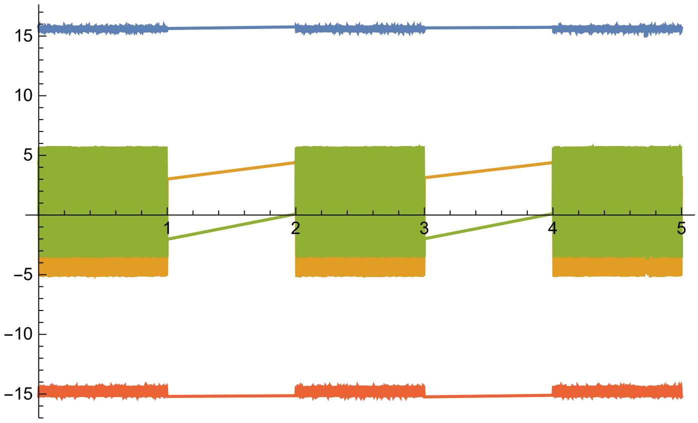


```
Start = 1;
Stop = 1000;
```

```
ListPlot[{Table[{Bvst[[i]][[1]], Bvst[[i]][[2]]}, {i, Start, Stop}],
  Table[{Bxvst[[i]][[1]], Bxvst[[i]][[2]]}, {i, Start, Stop}],
  Table[{Byvst[[i]][[1]], Byvst[[i]][[2]]}, {i, Start, Stop}],
  Table[{Bzvst[[i]][[1]], Bzvst[[i]][[2]]}, {i, Start, Stop}]}, Joined → True]
```

listenbezo··· Tabelle Tabelle Tabelle Tabelle verknüpft? wahr

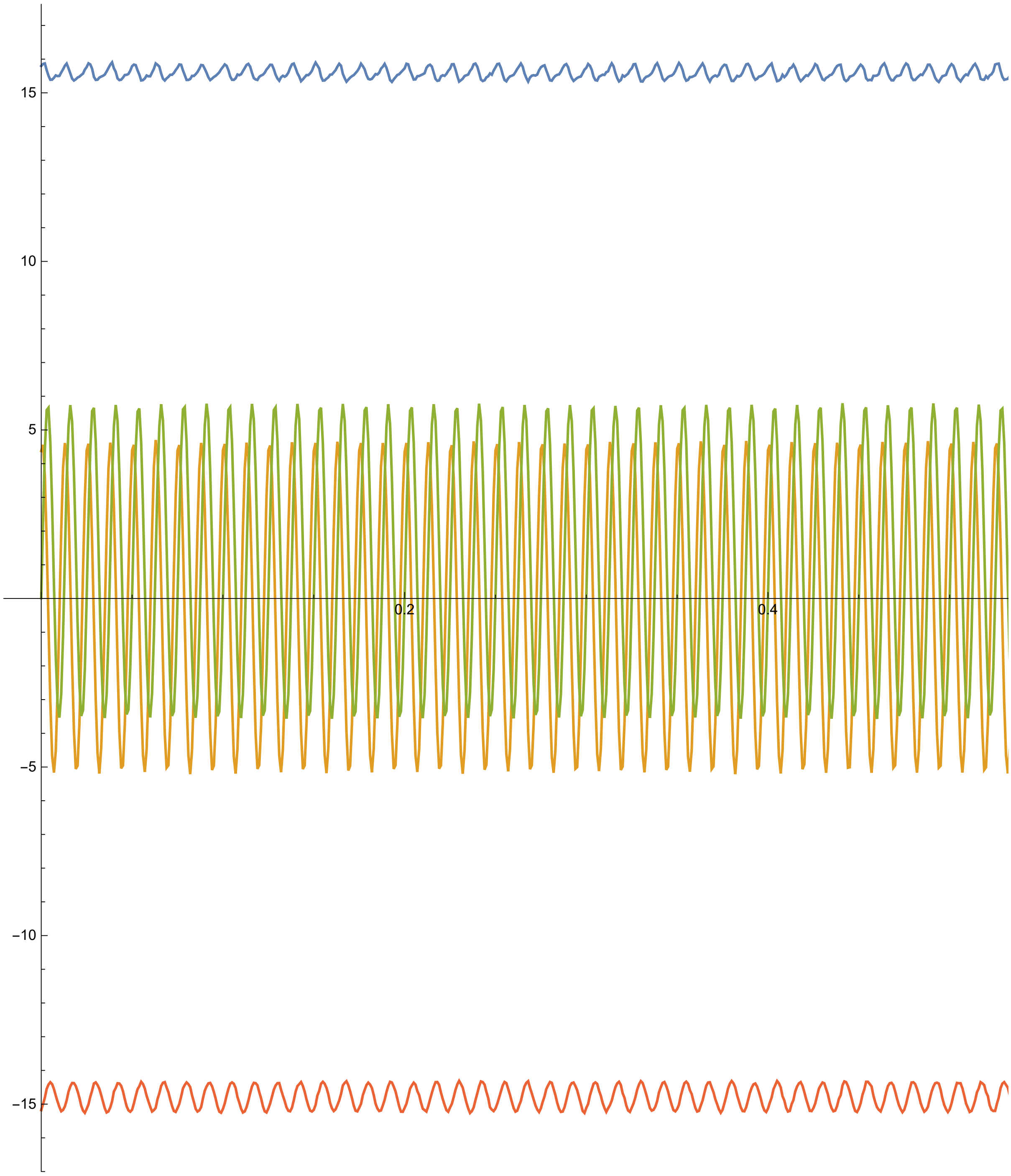

```
ListPlot[{Table[{Bvst[[i]][[1]], Bvst[[i]][[2]]}, {i, Start, Start + 50}],
listenbezo··· Tabelle
  Table[{Bxvst[[i]][[1]], Bxvst[[i]][[2]]}, {i, Start, Start + 50}],
  Tabelle
  Table[{Byvst[[i]][[1]], Byvst[[i]][[2]]}, {i, Start, Start + 50}],
  Tabelle
  Table[{Bzvst[[i]][[1]], Bzvst[[i]][[2]]}, {i, Start, Start + 50}]},
  Tabelle
 Joined → True, PlotLegends → {"B", "Bx", "By", "Bz"}]
 verknüpft? wahr  Legenden der Graphik
```

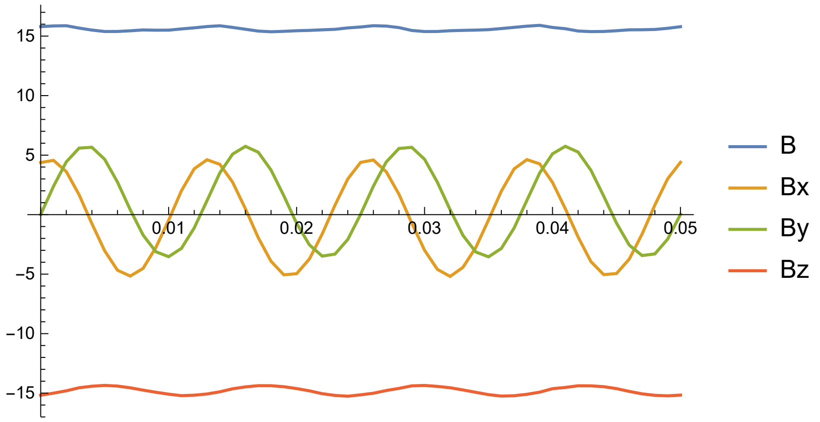


```
TableForm[{Mean[Table[Bvst[[i]][[2]], {i, Start, Stop}]],
Tabellendars···arit···  Tabelle
  Mean[Table[Bxvst[[i]][[2]], {i, Start, Stop}]],
  arit···  Tabelle
  Mean[Table[Byvst[[i]][[2]], {i, Start, Stop}]],
  arit···  Tabelle
  Mean[Table[Bzvst[[i]][[2]], {i, Start, Stop}]]}, TableDirections → Row]
  arit···  Tabelle                                 Richtung der Tabellen···Zeile
```

15.5834 -0.257941 1.09705 -14.7805

```
TableForm[Table[{(Bvst[[i]][[1]] - Bvst[[Start]][[1]]) * 1000, Bvst[[i]][[2]],
Tabellendar·· Tabelle
   Bxvst[[i]][[2]], Byvst[[i]][[2]], Bzvst[[i]][[2]]}, {i, Start, Start + 50}]]
```

0.	15.7971	4.37699	0.009407	-15.1786
1.0004	15.8572	4.55903	2.37915	-15.0002
2.00033	15.873	3.61755	4.43544	-14.8051
3.00026	15.6747	1.67272	5.58965	-14.5484
4.00019	15.5085	-0.756169	5.65919	-14.4193
5.00011	15.3885	-3.04139	4.64768	-14.3511
6.00004	15.3911	-4.67179	2.73564	-14.4075
7.00045	15.447	-5.16255	0.408081	-14.553
8.00037	15.5191	-4.519	-1.71554	-14.7471
9.0003	15.4962	-2.76408	-3.11717	-14.9256
10.0002	15.5022	-0.466117	-3.5344	-15.0867
11.0002	15.6044	2.00989	-2.83484	-15.2126
12.0001	15.6957	3.85434	-1.11333	-15.1743
13.	15.805	4.61172	1.18014	-15.0711
14.0004	15.8697	4.22582	3.51023	-14.8885
15.0003	15.7309	2.72449	5.08798	-14.6339
16.0003	15.5777	0.486233	5.73632	-14.4749
17.0002	15.4225	-1.93027	5.24723	-14.3734
18.0001	15.3654	-3.91863	3.75204	-14.3758
19.0001	15.4015	-5.056	1.58692	-14.4611
20.	15.4535	-4.96385	-0.728879	-14.6164
21.0004	15.4802	-3.7286	-2.5419	-14.8079
22.0003	15.5296	-1.62482	-3.4852	-15.046
23.0002	15.5708	0.795605	-3.31746	-15.1924
24.0002	15.6901	2.99657	-2.08603	-15.2593
25.0001	15.7658	4.38935	0.02387	-15.1425
26.	15.8758	4.59093	2.39031	-15.0084
27.0004	15.8441	3.60981	4.41807	-14.7813
28.0004	15.7174	1.71221	5.5595	-14.6013
29.0003	15.4755	-0.711838	5.65475	-14.3878
30.0002	15.3845	-2.99344	4.65179	-14.3556
31.0001	15.3899	-4.59703	2.72409	-14.4325
32.0001	15.4536	-5.19451	0.407982	-14.5487
33.	15.4827	-4.43081	-1.74268	-14.7324
34.0004	15.5057	-2.7721	-3.13712	-14.9298
35.0003	15.5435	-0.446518	-3.54576	-15.1271
36.0003	15.6353	1.96953	-2.84134	-15.2483
37.0002	15.7358	3.84591	-1.12068	-15.2173
38.0001	15.8364	4.62241	1.20358	-15.0988
39.	15.9059	4.26453	3.50671	-14.917
40.0004	15.7274	2.70555	5.11183	-14.6253
41.0004	15.6235	0.497744	5.74106	-14.5219
42.0003	15.4277	-1.87837	5.25841	-14.3818
43.0002	15.3788	-3.94573	3.7322	-14.3878
44.0001	15.3899	-5.03993	1.55589	-14.4577
45.0001	15.4524	-4.95191	-0.708806	-14.6202
46.	15.5302	-3.73306	-2.56418	-14.8552
47.0004	15.535	-1.63403	-3.43898	-15.0612
48.0003	15.5601	0.807671	-3.30157	-15.1843
49.0003	15.6577	3.01287	-2.0555	-15.227
50.0002	15.7912	4.39711	0.017247	-15.1666

(0,0,-2.5) RMF off

```
Data = Import[NotebookDirectory[] <> "230223_x0_y0_z-2_5_field_off.txt", "Table"];
       importi··· Notebook-Verzeichnis                                    Tabelle
```

```
Bvst = Table[{AbsoluteTime[Data[[i]][[9]]] - AbsoluteTime[Data[[2]][[9]]],
        Tabelle   absolute Zeit seit 1900                absolute Zeit seit 1900
    AbsoluteTime[Data[[i]][[2]]]}, {i, 2, Length[Data]}];
    absolute Zeit seit 1900                      Länge
Bxvst = Table[{AbsoluteTime[Data[[i]][[9]]] - AbsoluteTime[Data[[2]][[9]]],
         Tabelle   absolute Zeit seit 1900                absolute Zeit seit 1900
    AbsoluteTime[Data[[i]][[3]]]}, {i, 2, Length[Data]}];
    absolute Zeit seit 1900                      Länge
Byvst = Table[{AbsoluteTime[Data[[i]][[9]]] - AbsoluteTime[Data[[2]][[9]]],
         Tabelle   absolute Zeit seit 1900                absolute Zeit seit 1900
    AbsoluteTime[Data[[i]][[4]]]}, {i, 2, Length[Data]}];
    absolute Zeit seit 1900                      Länge
Bzvst = Table[{AbsoluteTime[Data[[i]][[9]]] - AbsoluteTime[Data[[2]][[9]]],
         Tabelle   absolute Zeit seit 1900                absolute Zeit seit 1900
    AbsoluteTime[Data[[i]][[5]]]}, {i, 2, Length[Data]}];
    absolute Zeit seit 1900                      Länge
```

```
ListPlot[{Bvst, Bxvst, Byvst, Bzvst}, Joined → True]
listenbezogene Graphik                    verknüpft? wahr
```

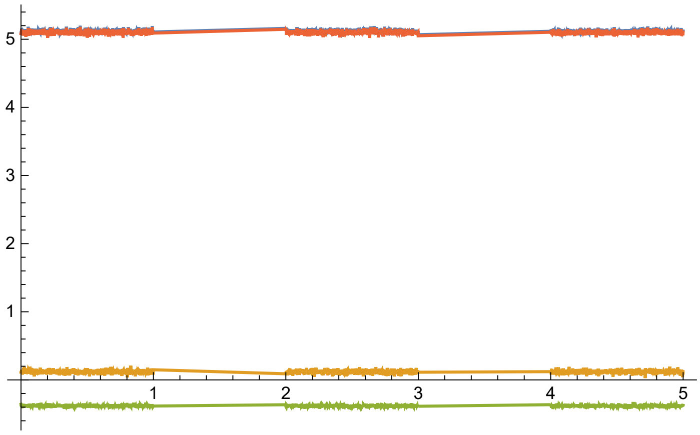


```
Start = 1;
Stop = 1000;
```

```
ListPlot[{Table[{Bvst[[i]][[1]], Bvst[[i]][[2]]}, {i, Start, Stop}],
  Table[{Bxvst[[i]][[1]], Bxvst[[i]][[2]]}, {i, Start, Stop}],
  Table[{Byvst[[i]][[1]], Byvst[[i]][[2]]}, {i, Start, Stop}],
  Table[{Bzvst[[i]][[1]], Bzvst[[i]][[2]]}, {i, Start, Stop}]}, Joined → True]
```

listenbezo··· Tabelle Tabelle Tabelle Tabelle verknüpft? wahr

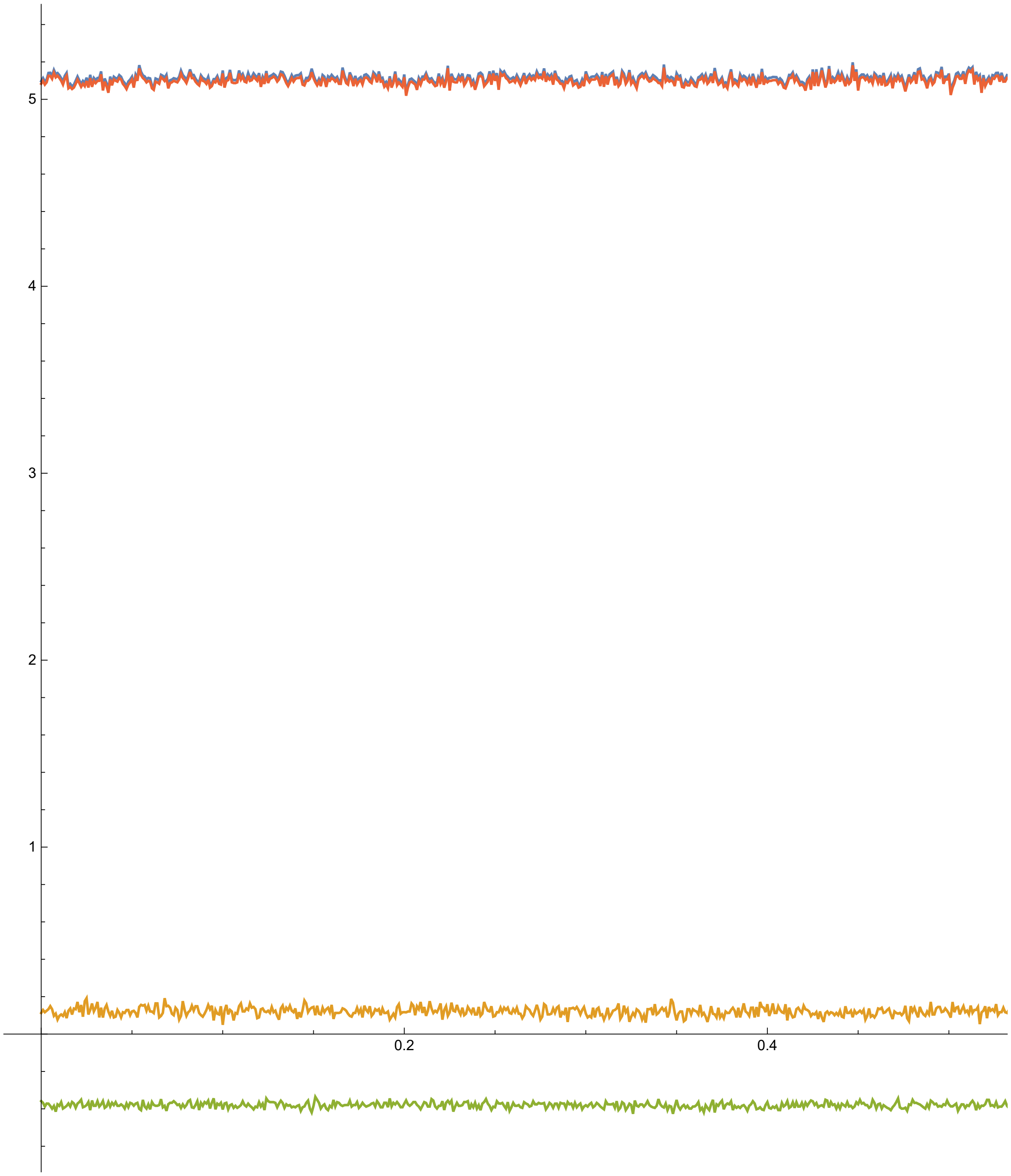

```
ListPlot[{Table[{Bvst[[i]][[1]], Bvst[[i]][[2]]}, {i, Start, Start + 50}],
listenbezo··· Tabelle
  Table[{Bxvst[[i]][[1]], Bxvst[[i]][[2]]}, {i, Start, Start + 50}],
  Tabelle
  Table[{Byvst[[i]][[1]], Byvst[[i]][[2]]}, {i, Start, Start + 50}],
  Tabelle
  Table[{Bzvst[[i]][[1]], Bzvst[[i]][[2]]}, {i, Start, Start + 50}]},
  Tabelle
 Joined → True, PlotLegends → {"B", "Bx", "By", "Bz"}]
 verknüpft? wahr  Legenden der Graphik
```

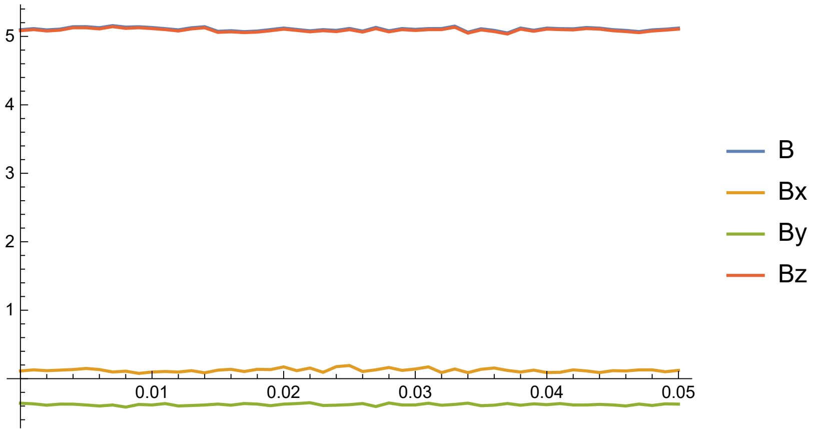


```
TableForm[{Mean[Table[Bvst[[i]][[2]], {i, Start, Stop}]],
Tabellendars···arit···  Tabelle
  Mean[Table[Bxvst[[i]][[2]], {i, Start, Stop}]],
  arit···  Tabelle
  Mean[Table[Byvst[[i]][[2]], {i, Start, Stop}]],
  arit···  Tabelle
  Mean[Table[Bzvst[[i]][[2]], {i, Start, Stop}]]}, TableDirections → Row]
  arit···  Tabelle                              Richtung der Tabellen···Zeile
```

```
5.1158  0.120107  -0.381107  5.10009
```

```
TableForm[Table[{(Bvst[[i]][[1]] - Bvst[[Start]][[1]]) * 1000, Bvst[[i]][[2]],
  Bxvst[[i]][[2]], Byvst[[i]][[2]], Bzvst[[i]][[2]]}, {i, Start, Start + 50}]]
```
Tabellendar·· Tabelle

0.	5.09711	0.112752	-0.358589	5.08323
0.999928	5.1138	0.128926	-0.367813	5.09892
1.99986	5.09484	0.116741	-0.38752	5.07874
2.99978	5.10598	0.124847	-0.371695	5.0909
3.99971	5.14104	0.133208	-0.37222	5.12581
5.00011	5.14201	0.149219	-0.3842	5.12547
6.00004	5.12669	0.133071	-0.398973	5.1094
6.99997	5.15727	0.097387	-0.384506	5.142
7.9999	5.13538	0.109171	-0.415121	5.11741
8.99982	5.1407	0.077215	-0.376286	5.12633
9.99975	5.12933	0.097098	-0.384078	5.114
10.9997	5.11327	0.104923	-0.363842	5.09923
12.0001	5.09531	0.096753	-0.398542	5.07878
13.	5.12603	0.117064	-0.391993	5.10968
13.9999	5.14121	0.085223	-0.384276	5.12613
14.9999	5.07404	0.124518	-0.371207	5.05891
15.9998	5.08316	0.136616	-0.387313	5.06654
16.9997	5.06934	0.104469	-0.36317	5.05524
18.0001	5.07821	0.136558	-0.371254	5.06278
19.0001	5.09949	0.132788	-0.394561	5.08247
20.	5.12183	0.172	-0.371869	5.10542
20.9999	5.10143	0.116798	-0.363645	5.08711
21.9998	5.08144	0.155578	-0.351295	5.06689
22.9998	5.09884	0.092787	-0.391609	5.08293
23.9997	5.08794	0.175655	-0.387328	5.07014
25.0001	5.11581	0.191935	-0.379737	5.09809
26.	5.07733	0.104552	-0.363293	5.06324
27.	5.13112	0.12912	-0.407038	5.11332
27.9999	5.08182	0.163581	-0.355285	5.06675
28.9998	5.11462	0.120942	-0.383821	5.09876
29.9997	5.10294	0.140818	-0.383614	5.08656
30.9997	5.11411	0.171914	-0.358763	5.09862
32.0001	5.11453	0.088948	-0.387858	5.09903
33.	5.14938	0.141294	-0.376332	5.13367
33.9999	5.06094	0.088382	-0.358068	5.04748
34.9998	5.11153	0.136912	-0.394739	5.09443
35.9998	5.0875	0.155656	-0.387351	5.07034
36.9997	5.04956	0.120262	-0.362846	5.03508
38.0001	5.12158	0.09702	-0.387956	5.10594
39.	5.08978	0.124678	-0.367452	5.07497
40.	5.12104	0.089012	-0.379966	5.10615
40.9999	5.11316	0.092923	-0.363856	5.09935
41.9998	5.11074	0.128901	-0.38375	5.09468
42.9997	5.12949	0.113098	-0.384059	5.11384
43.9997	5.12081	0.089008	-0.375967	5.10621
45.0001	5.09859	0.116778	-0.383581	5.0828
46.	5.08749	0.11267	-0.398401	5.07062
46.9999	5.0701	0.128476	-0.371141	5.05487
47.9999	5.09525	0.128744	-0.391505	5.07856
48.9998	5.10547	0.100845	-0.367724	5.09121
49.9997	5.1209	0.121002	-0.371929	5.10594

(0,0,-2.5) RMF on

```
Data = Import[NotebookDirectory[] <> "230223_x0_y0_z-2_5_field_on_5mT.txt", "Table"];
```
importi··· Notebook-Verzeichnis Tabelle

```
Bvst = Table[{AbsoluteTime[Data[[i]][[9]]] - AbsoluteTime[Data[[2]][[9]]],
        Tabelle  absolute Zeit seit 1900                  absolute Zeit seit 1900
    AbsoluteTime[Data[[i]][[2]]]}, {i, 2, Length[Data]}];
    absolute Zeit seit 1900                       Länge
Bxvst = Table[{AbsoluteTime[Data[[i]][[9]]] - AbsoluteTime[Data[[2]][[9]]],
         Tabelle  absolute Zeit seit 1900                  absolute Zeit seit 1900
    AbsoluteTime[Data[[i]][[3]]]}, {i, 2, Length[Data]}];
    absolute Zeit seit 1900                       Länge
Byvst = Table[{AbsoluteTime[Data[[i]][[9]]] - AbsoluteTime[Data[[2]][[9]]],
         Tabelle  absolute Zeit seit 1900                  absolute Zeit seit 1900
    AbsoluteTime[Data[[i]][[4]]]}, {i, 2, Length[Data]}];
    absolute Zeit seit 1900                       Länge
Bzvst = Table[{AbsoluteTime[Data[[i]][[9]]] - AbsoluteTime[Data[[2]][[9]]],
         Tabelle  absolute Zeit seit 1900                  absolute Zeit seit 1900
    AbsoluteTime[Data[[i]][[5]]]}, {i, 2, Length[Data]}];
    absolute Zeit seit 1900                       Länge
```

```
ListPlot[{Bvst, Bxvst, Byvst, Bzvst}, Joined → True]
listenbezogene Graphik                    verknüpft? wahr
```

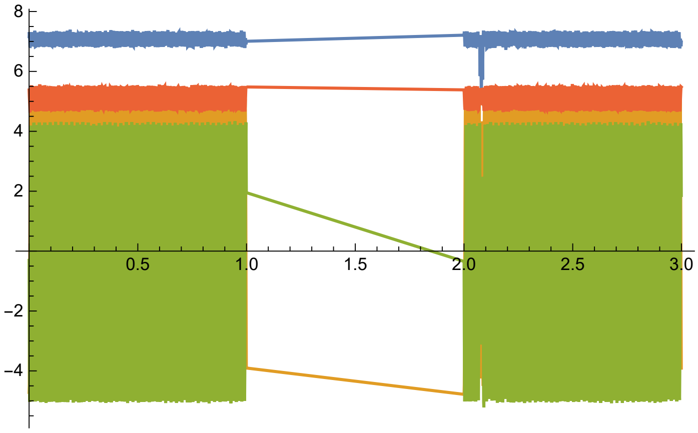


```
Start = 1;
Stop = 1000;
```

```
ListPlot[{Table[{Bvst[[i]][[1]], Bvst[[i]][[2]]}, {i, Start, Stop}],
listenbezo··· Tabelle
   Table[{Bxvst[[i]][[1]], Bxvst[[i]][[2]]}, {i, Start, Stop}],
   Tabelle
   Table[{Byvst[[i]][[1]], Byvst[[i]][[2]]}, {i, Start, Stop}],
   Tabelle
   Table[{Bzvst[[i]][[1]], Bzvst[[i]][[2]]}, {i, Start, Stop}]}, Joined → True]
   Tabelle                                               verknüpft? wahr
```

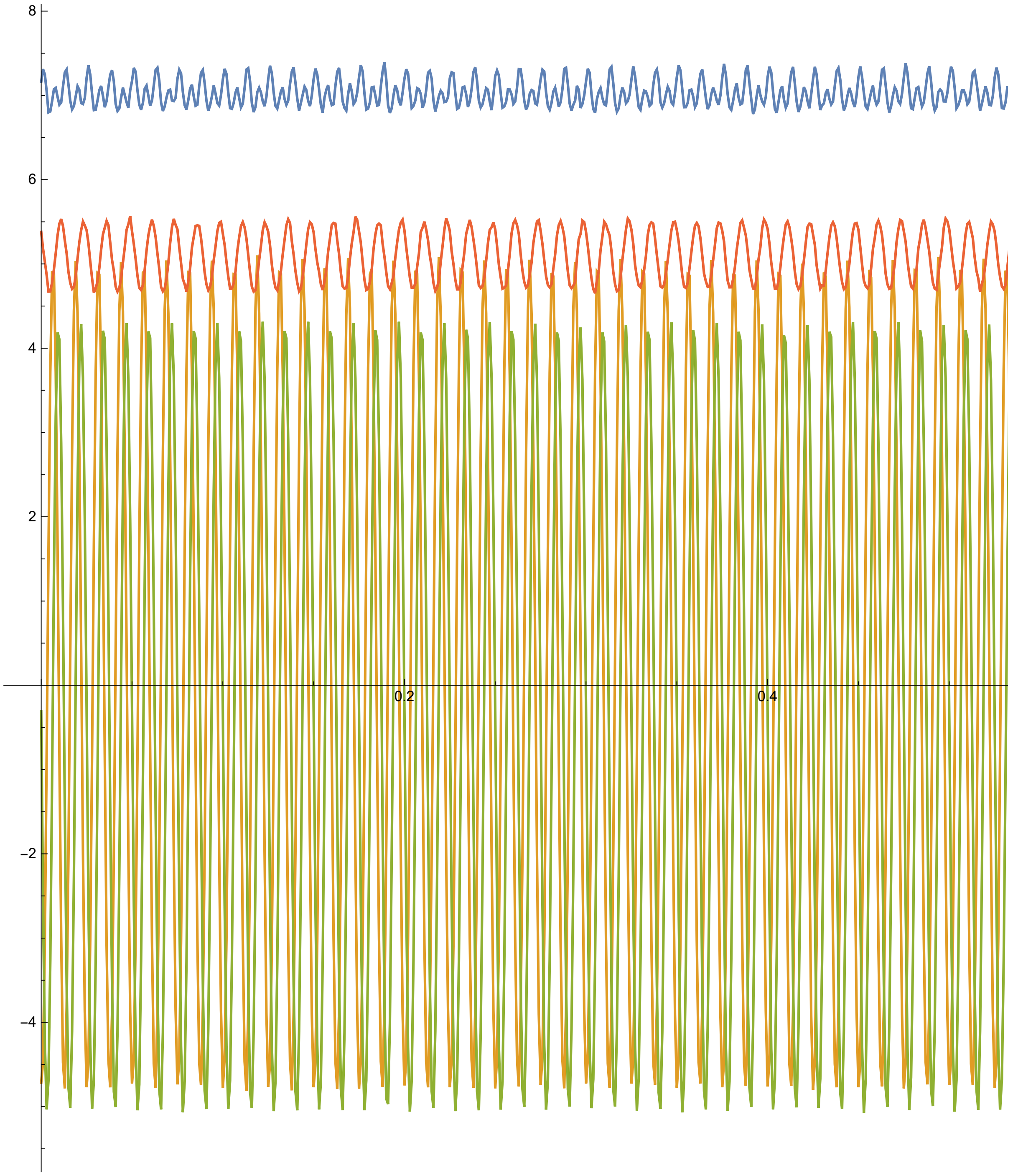

```
ListPlot[{Table[{Bvst[[i]][[1]], Bvst[[i]][[2]]}, {i, Start, Start + 50}],
 listenbezo⋯  Tabelle
  Table[{Bxvst[[i]][[1]], Bxvst[[i]][[2]]}, {i, Start, Start + 50}],
  Tabelle
  Table[{Byvst[[i]][[1]], Byvst[[i]][[2]]}, {i, Start, Start + 50}],
  Tabelle
  Table[{Bzvst[[i]][[1]], Bzvst[[i]][[2]]}, {i, Start, Start + 50}]},
  Tabelle
 Joined → True, PlotLegends → {"B", "Bx", "By", "Bz"}]
 verknüpft? wahr  Legenden der Graphik
```

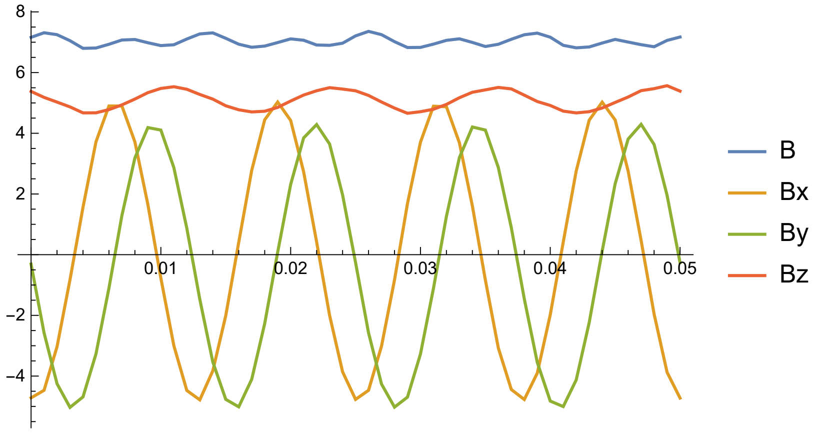


```
TableForm[{Mean[Table[Bvst[[i]][[2]], {i, Start, Stop}]],
Tabellendars⋯ arit⋯  Tabelle
  Mean[Table[Bxvst[[i]][[2]], {i, Start, Stop}]],
  arit⋯  Tabelle
  Mean[Table[Byvst[[i]][[2]], {i, Start, Stop}]],
  arit⋯  Tabelle
  Mean[Table[Bzvst[[i]][[2]], {i, Start, Stop}]]}, TableDirections → Row]
  arit⋯  Tabelle                                  Richtung der Tabellen⋯ Zeile
```

7.03396 0.119619 -0.38912 5.10364

```
TableForm[Table[{(Bvst[[i]][[1]] - Bvst[[Start]][[1]]) * 1000, Bvst[[i]][[2]],
   Bxvst[[i]][[2]], Byvst[[i]][[2]], Bzvst[[i]][[2]]}, {i, Start, Start + 50}]]
```
Tabellendar·· Tabelle

0.	7.16128	-4.71651	-0.310084	5.37981
0.999928	7.312	-4.46984	-2.57408	5.18266
1.99986	7.24864	-3.03238	-4.25143	5.02721
2.99978	7.05068	-0.824954	-5.03184	4.86951
4.00019	6.79879	1.55973	-4.68633	4.67217
5.00011	6.80948	3.71819	-3.26998	4.67454
6.00004	6.93264	4.89976	-1.10011	4.7795
6.99997	7.0728	4.89852	1.29379	4.93509
7.9999	7.08944	3.71519	3.18886	5.12725
8.99982	6.98321	1.65107	4.18762	5.33882
9.99975	6.88924	-0.773533	4.106	5.47759
11.0002	6.91635	-2.99561	2.87071	5.53365
12.0001	7.10555	-4.47517	0.869225	5.45033
13.	7.27354	-4.78318	-1.46975	5.27877
13.9999	7.30817	-3.82925	-3.53068	5.12645
14.9999	7.12938	-2.00796	-4.76758	4.90575
15.9998	6.93471	0.409108	-5.01311	4.77406
16.9997	6.83414	2.77843	-4.10648	4.70346
18.0001	6.87317	4.44458	-2.2689	4.72634
19.0001	6.9895	5.03012	0.083052	4.85223
20.	7.10991	4.4226	2.31547	5.06261
20.9999	7.06523	2.73072	3.84691	5.25947
21.9998	6.90869	0.420528	4.28742	5.40104
22.9998	6.89783	-1.99979	3.64629	5.50322
24.0002	6.96789	-3.86038	1.96789	5.45677
25.0001	7.20874	-4.76837	-0.259442	5.40012
26.	7.35744	-4.46918	-2.57506	5.24665
27.	7.24928	-3.01232	-4.26246	5.03084
27.9999	7.01879	-0.833364	-5.02325	4.83072
28.9998	6.82511	1.68661	-4.69399	4.65874
29.9997	6.82968	3.70657	-3.27354	4.71061
31.0001	6.93725	4.8919	-1.1123	4.7914
32.0001	7.06172	4.87472	1.26252	4.95086
33.	7.1124	3.68763	3.20016	5.17172
33.9999	6.99371	1.60318	4.20738	5.35161
34.9998	6.85912	-0.834024	4.10666	5.43022
35.9998	6.93271	-3.07485	2.87098	5.51047
37.0002	7.09691	-4.44307	0.888078	5.46229
38.0001	7.24668	-4.77143	-1.45738	5.25583
39.	7.29996	-3.90205	-3.54854	5.04691
40.	7.16598	-1.97978	-4.82272	4.91662
40.9999	6.89847	0.412646	-5.00443	4.73015
41.9998	6.81564	2.75812	-4.12602	4.67137
42.9997	6.84411	4.43238	-2.23668	4.71096
44.0001	6.97348	5.02587	0.118348	4.83281
45.0001	7.09279	4.43809	2.33521	5.01575
46.	7.00551	2.7541	3.80892	5.19464
46.9999	6.91951	0.467559	4.29541	5.40467
47.9999	6.85098	-1.95611	3.63083	5.47053
48.9998	7.05973	-3.87522	1.95518	5.56775
49.9997	7.17126	-4.72448	-0.263221	5.3886

(0,0,-5) RMF off

```
Data = Import[NotebookDirectory[] <> "230223_x0_y0_z-5_field_off.txt", "Table"];
```
importi··· Notebook-Verzeichnis Tabelle

```
Bvst = Table[{AbsoluteTime[Data[[i]][[9]]] - AbsoluteTime[Data[[2]][[9]]],
        Tabelle   absolute Zeit seit 1900                  absolute Zeit seit 1900
    AbsoluteTime[Data[[i]][[2]]]}, {i, 2, Length[Data]}];
    absolute Zeit seit 1900                       Länge
Bxvst = Table[{AbsoluteTime[Data[[i]][[9]]] - AbsoluteTime[Data[[2]][[9]]],
         Tabelle   absolute Zeit seit 1900                  absolute Zeit seit 1900
    AbsoluteTime[Data[[i]][[3]]]}, {i, 2, Length[Data]}];
    absolute Zeit seit 1900                       Länge
Byvst = Table[{AbsoluteTime[Data[[i]][[9]]] - AbsoluteTime[Data[[2]][[9]]],
         Tabelle   absolute Zeit seit 1900                  absolute Zeit seit 1900
    AbsoluteTime[Data[[i]][[4]]]}, {i, 2, Length[Data]}];
    absolute Zeit seit 1900                       Länge
Bzvst = Table[{AbsoluteTime[Data[[i]][[9]]] - AbsoluteTime[Data[[2]][[9]]],
         Tabelle   absolute Zeit seit 1900                  absolute Zeit seit 1900
    AbsoluteTime[Data[[i]][[5]]]}, {i, 2, Length[Data]}];
    absolute Zeit seit 1900                       Länge
```

```
ListPlot[{Bvst, Bxvst, Byvst, Bzvst}, Joined → True]
listenbezogene Graphik                  verknüpft? wahr
```

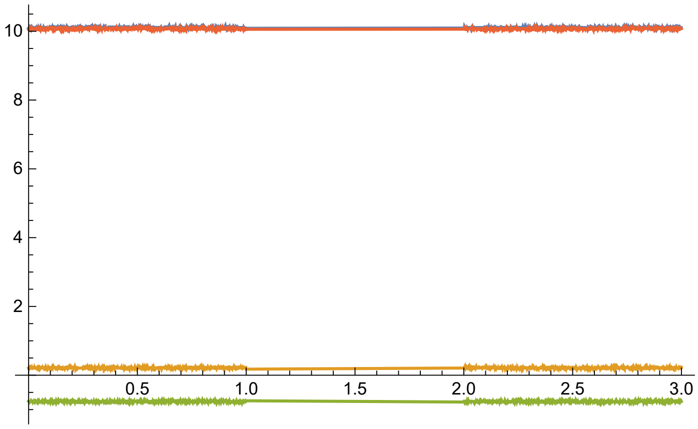


```
Start = 1;
Stop = 1000;
```

```
ListPlot[{Table[{Bvst[[i]][[1]], Bvst[[i]][[2]]}, {i, Start, Stop}],
  Table[{Bxvst[[i]][[1]], Bxvst[[i]][[2]]}, {i, Start, Stop}],
  Table[{Byvst[[i]][[1]], Byvst[[i]][[2]]}, {i, Start, Stop}],
  Table[{Bzvst[[i]][[1]], Bzvst[[i]][[2]]}, {i, Start, Stop}]}, Joined → True]
```

listenbezo··· Tabelle Tabelle Tabelle Tabelle verknüpft? wahr

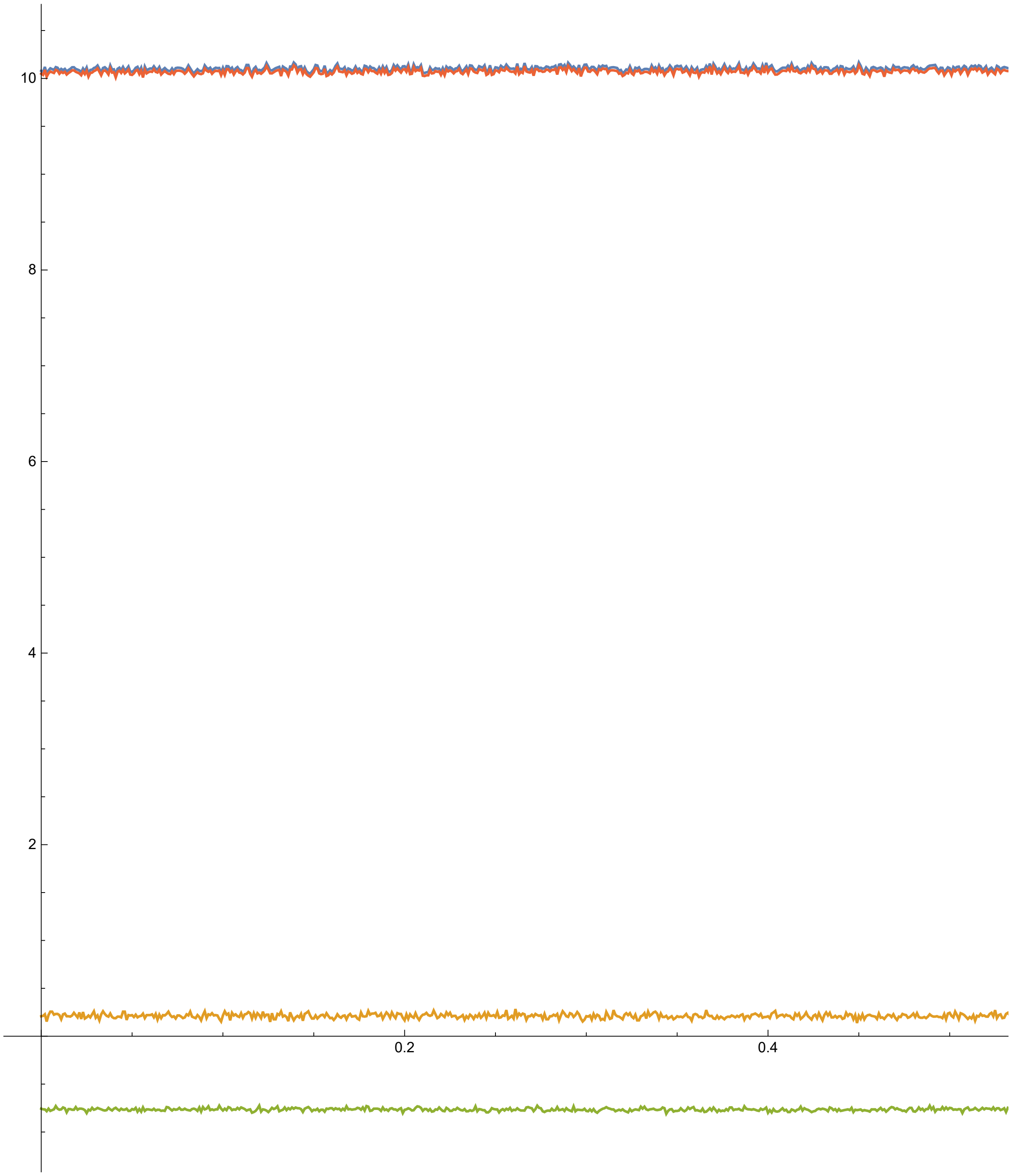

```
ListPlot[{Table[{Bvst[[i]][[1]], Bvst[[i]][[2]]}, {i, Start, Start + 50}],
listenbezo··· Tabelle
  Table[{Bxvst[[i]][[1]], Bxvst[[i]][[2]]}, {i, Start, Start + 50}],
  Tabelle
  Table[{Byvst[[i]][[1]], Byvst[[i]][[2]]}, {i, Start, Start + 50}],
  Tabelle
  Table[{Bzvst[[i]][[1]], Bzvst[[i]][[2]]}, {i, Start, Start + 50}]},
  Tabelle
 Joined → True, PlotLegends → {"B", "Bx", "By", "Bz"}]
 verknüpft? wahr   Legenden der Graphik
```

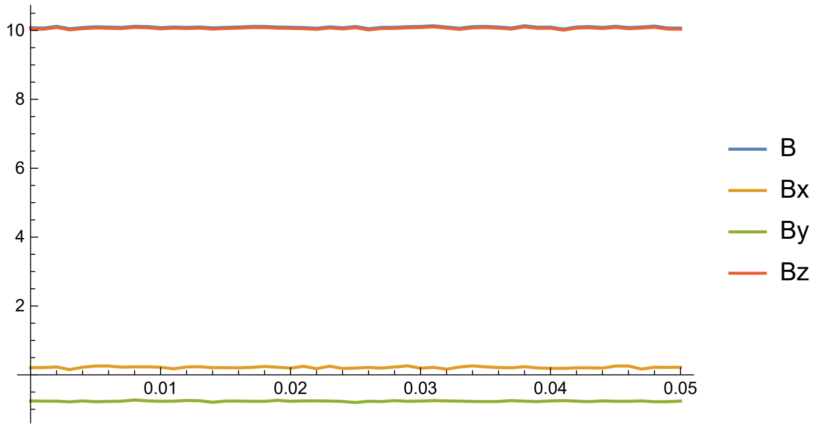


```
TableForm[{Mean[Table[Bvst[[i]][[2]], {i, Start, Stop}]],
Tabellendars···arit···  Tabelle
  Mean[Table[Bxvst[[i]][[2]], {i, Start, Stop}]],
  arit···  Tabelle
  Mean[Table[Byvst[[i]][[2]], {i, Start, Stop}]],
  arit···  Tabelle
  Mean[Table[Bzvst[[i]][[2]], {i, Start, Stop}]]}, TableDirections → Row]
  arit···  Tabelle                                  Richtung der Tabellen··· Zeile
```

10.1031 0.212885 −0.764734 10.0718

```
TableForm[Table[{(Bvst[[i]][[1]] - Bvst[[Start]][[1]]) * 1000, Bvst[[i]][[2]],
    Bxvst[[i]][[2]], Byvst[[i]][[2]], Bzvst[[i]][[2]]}, {i, Start, Start + 50}]]
```

Tabellendar·· Tabelle

0.	10.0796	0.208341	-0.756406	10.049
1.0004	10.0681	0.212225	-0.763217	10.0369
2.00033	10.1202	0.22876	-0.763992	10.0887
3.00026	10.0499	0.153052	-0.782995	10.0182
4.00019	10.0837	0.220382	-0.756453	10.0529
5.00011	10.1056	0.25661	-0.779713	10.0722
6.00004	10.0971	0.256519	-0.771591	10.0643
6.99997	10.0882	0.22443	-0.763508	10.0567
8.00037	10.1182	0.232724	-0.728991	10.0892
9.0003	10.1118	0.23267	-0.756866	10.0808
10.0002	10.0804	0.220352	-0.76739	10.0487
11.0002	10.0997	0.176556	-0.763748	10.0692
12.0001	10.087	0.228409	-0.743506	10.057
13.	10.0996	0.236541	-0.751679	10.0688
14.0004	10.074	0.208299	-0.795279	10.0404
15.0003	10.0878	0.208428	-0.760528	10.0569
16.0003	10.0998	0.204552	-0.760716	10.069
17.0002	10.1163	0.216723	-0.767945	10.0848
18.0001	10.1166	0.244722	-0.767911	10.0845
19.0001	10.0987	0.220529	-0.739699	10.0691
20.	10.0921	0.196477	-0.767602	10.061
21.0004	10.0841	0.24838	-0.75642	10.0526
22.0003	10.0634	0.180178	-0.756195	10.0333
23.0002	10.1043	0.25259	-0.76072	10.0725
24.0002	10.0763	0.184317	-0.771372	10.045
25.0001	10.1143	0.196719	-0.802903	10.0804
26.	10.0494	0.216033	-0.766922	10.0178
27.0004	10.0886	0.196444	-0.77554	10.0568
28.0004	10.0913	0.228455	-0.747566	10.0609
29.0003	10.1089	0.260638	-0.76777	10.0763
30.0002	10.1156	0.192717	-0.760974	10.0851
31.0001	10.135	0.216909	-0.748252	10.1051
32.0001	10.0992	0.16555	-0.756762	10.0694
33.	10.0643	0.228182	-0.763137	10.0327
34.0004	10.1091	0.260643	-0.77177	10.0762
35.0003	10.117	0.232731	-0.775925	10.0845
36.0003	10.1005	0.212563	-0.771705	10.0687
37.0002	10.071	0.20425	-0.74729	10.0412
38.0001	10.1362	0.236924	-0.764227	10.1046
39.	10.0926	0.200485	-0.775596	10.0608
40.0004	10.0954	0.188507	-0.756674	10.0652
41.0004	10.0399	0.187931	-0.746835	10.0104
42.0003	10.0999	0.204555	-0.763715	10.0689
43.0002	10.1126	0.204691	-0.775897	10.0808
44.0001	10.0875	0.196424	-0.756542	10.0571
45.0001	10.1208	0.256762	-0.767958	10.0883
46.	10.0849	0.256391	-0.767409	10.0523
47.0004	10.0992	0.16955	-0.756757	10.0694
48.0003	10.125	0.220818	-0.780061	10.0925
49.0003	10.0731	0.216282	-0.779272	10.0406
50.0002	10.068	0.220221	-0.760208	10.0368

(0,0,-5) RMF on

```
Data = Import[NotebookDirectory[] <> "230223_x0_y0_z-5_5mT_on.txt", "Table"];
```

importi··· Notebook-Verzeichnis Tabelle

```
Bvst = Table[{AbsoluteTime[Data[[i]][[9]]] - AbsoluteTime[Data[[2]][[9]]],
        Tabelle  absolute Zeit seit 1900                  absolute Zeit seit 1900
    AbsoluteTime[Data[[i]][[2]]]}, {i, 2, Length[Data]}];
    absolute Zeit seit 1900                           Länge
Bxvst = Table[{AbsoluteTime[Data[[i]][[9]]] - AbsoluteTime[Data[[2]][[9]]],
         Tabelle  absolute Zeit seit 1900                  absolute Zeit seit 1900
    AbsoluteTime[Data[[i]][[3]]]}, {i, 2, Length[Data]}];
    absolute Zeit seit 1900                           Länge
Byvst = Table[{AbsoluteTime[Data[[i]][[9]]] - AbsoluteTime[Data[[2]][[9]]],
         Tabelle  absolute Zeit seit 1900                  absolute Zeit seit 1900
    AbsoluteTime[Data[[i]][[4]]]}, {i, 2, Length[Data]}];
    absolute Zeit seit 1900                           Länge
Bzvst = Table[{AbsoluteTime[Data[[i]][[9]]] - AbsoluteTime[Data[[2]][[9]]],
         Tabelle  absolute Zeit seit 1900                  absolute Zeit seit 1900
    AbsoluteTime[Data[[i]][[5]]]}, {i, 2, Length[Data]}];
    absolute Zeit seit 1900                           Länge
```

```
ListPlot[{Bvst, Bxvst, Byvst, Bzvst}, Joined → True]
listenbezogene Graphik                  verknüpft? wahr
```

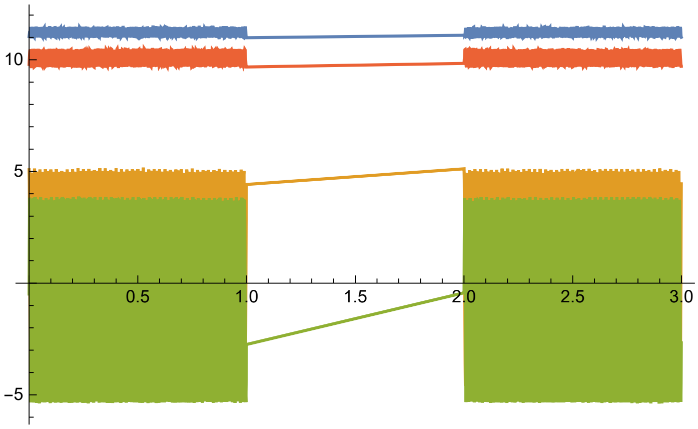


```
Start = 1;
Stop = 1000;
```

```
ListPlot[{Table[{Bvst[[i]][[1]], Bvst[[i]][[2]]}, {i, Start, Stop}],
listenbezo··· Tabelle
   Table[{Bxvst[[i]][[1]], Bxvst[[i]][[2]]}, {i, Start, Stop}],
   Tabelle
   Table[{Byvst[[i]][[1]], Byvst[[i]][[2]]}, {i, Start, Stop}],
   Tabelle
   Table[{Bzvst[[i]][[1]], Bzvst[[i]][[2]]}, {i, Start, Stop}]}, Joined → True]
   Tabelle                                                    verknüpft? wahr
```

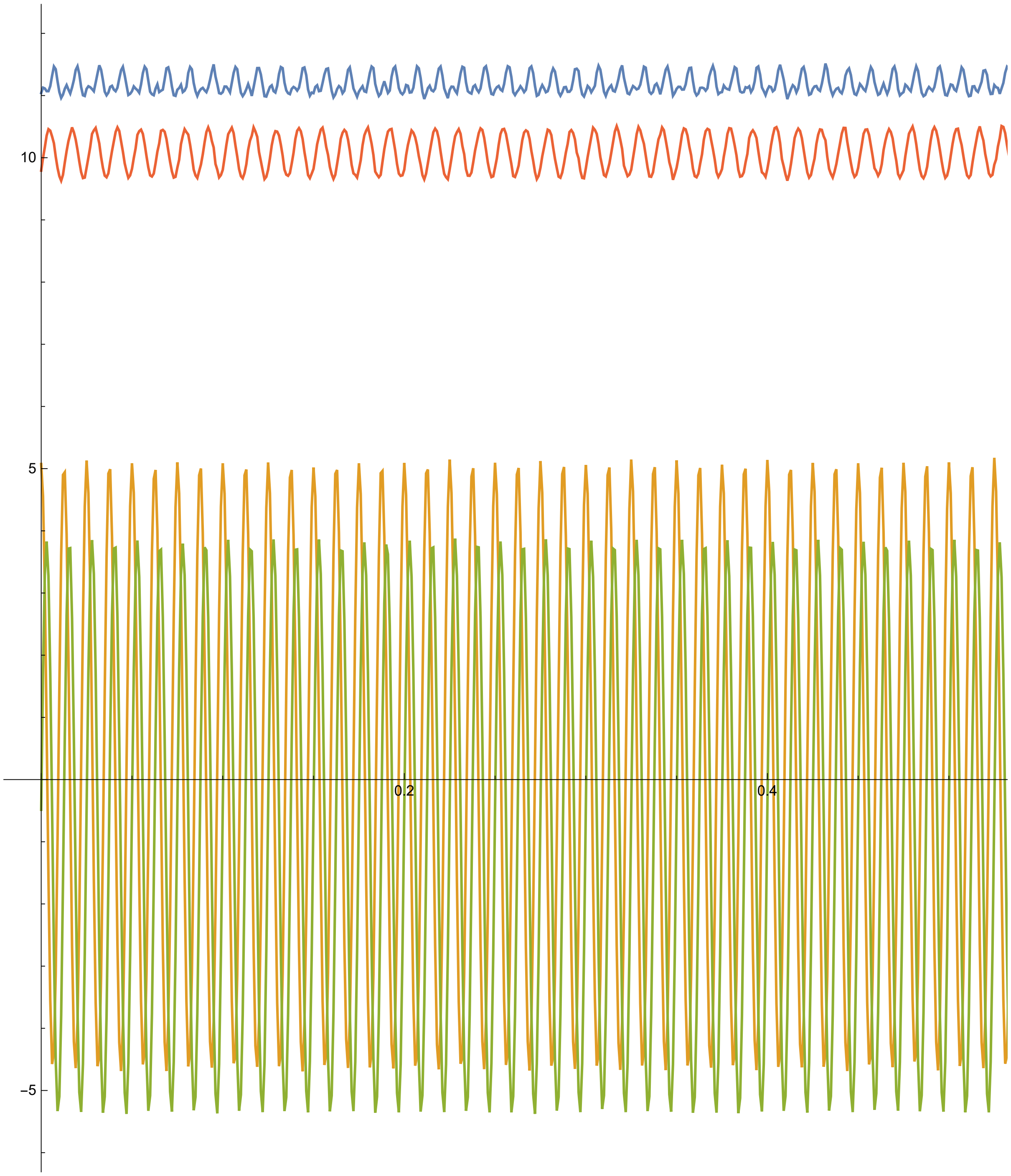

```
ListPlot[{Table[{Bvst[[i]][[1]], Bvst[[i]][[2]]}, {i, Start, Start + 50}],
listenbezo⋯  Tabelle
  Table[{Bxvst[[i]][[1]], Bxvst[[i]][[2]]}, {i, Start, Start + 50}],
  Tabelle
  Table[{Byvst[[i]][[1]], Byvst[[i]][[2]]}, {i, Start, Start + 50}],
  Tabelle
  Table[{Bzvst[[i]][[1]], Bzvst[[i]][[2]]}, {i, Start, Start + 50}]},
  Tabelle
 Joined → True, PlotLegends → {"B", "Bx", "By", "Bz"}]
 verknüpft? wahr  Legenden der Graphik
```

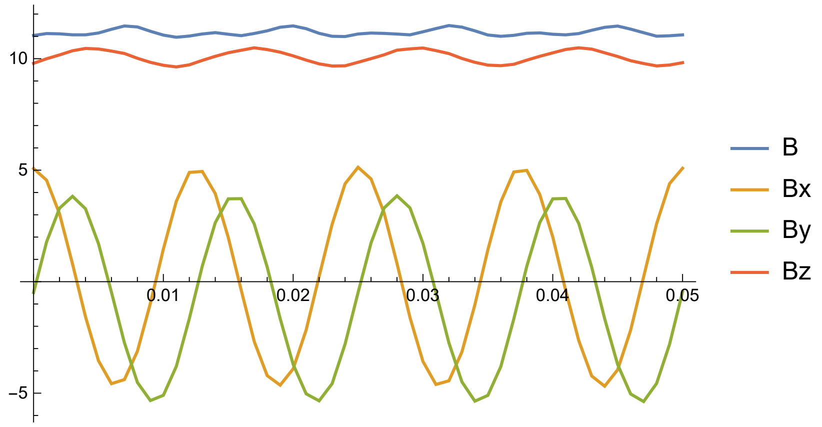


```
TableForm[{Mean[Table[Bvst[[i]][[2]], {i, Start, Stop}]],
Tabellendars⋯ arit⋯  Tabelle
  Mean[Table[Bxvst[[i]][[2]], {i, Start, Stop}]],
  arit⋯  Tabelle
  Mean[Table[Byvst[[i]][[2]], {i, Start, Stop}]],
  arit⋯  Tabelle
  Mean[Table[Bzvst[[i]][[2]], {i, Start, Stop}]]}, TableDirections → Row]
  arit⋯  Tabelle                                       Richtung der Tabellen⋯ Zeile
```

11.1791 0.21286 −0.771978 10.08

```
TableForm[Table[{(Bvst[[i]][[1]] - Bvst[[Start]][[1]]) * 1000, Bvst[[i]][[2]],
Tabellendar·· Tabelle
   Bxvst[[i]][[2]], Byvst[[i]][[2]], Bzvst[[i]][[2]]}, {i, Start, Start + 50}]]
```

0.	11.0403	5.07465	-0.485454	9.79293
1.0004	11.1272	4.55302	1.77917	9.99592
2.00033	11.1139	3.04392	3.29416	10.1686
3.00026	11.07	0.798093	3.82905	10.356
4.00019	11.0701	-1.57031	3.26997	10.4589
5.00011	11.152	-3.54281	1.70812	10.4354
6.00004	11.318	-4.57424	-0.472766	10.3417
7.00045	11.4674	-4.38966	-2.74118	10.2331
8.00037	11.4238	-3.11869	-4.5188	10.0179
9.0003	11.2297	-0.967485	-5.3309	9.83626
10.0002	11.0572	1.461	-5.09535	9.70379
11.0002	10.9616	3.60682	-3.79487	9.63051
12.0001	11.015	4.90033	-1.65673	9.72484
13.	11.1084	4.94257	0.701555	9.92344
14.0004	11.168	3.94609	2.64796	10.1065
15.0003	11.0963	1.98437	3.71463	10.266
16.0003	11.0326	-0.384612	3.72247	10.3786
17.0002	11.1304	-2.68126	2.58831	10.4879
18.0001	11.2493	-4.21285	0.652692	10.4102
19.0001	11.4107	-4.64035	-1.65522	10.2923
20.	11.4705	-3.90858	-3.70817	10.1264
21.0004	11.3458	-2.16587	-5.02864	9.93724
22.0003	11.1372	0.234899	-5.34363	9.76866
23.0002	11.0044	2.57212	-4.57467	9.67228
24.0002	10.9928	4.3932	-2.79771	9.68064
25.0001	11.1072	5.13013	-0.490106	9.8393
26.	11.1494	4.60915	1.73512	10.0027
27.0004	11.1339	3.09595	3.30216	10.1722
28.0004	11.1044	0.814346	3.85165	10.3832
29.0003	11.069	-1.60656	3.30923	10.4399
30.0002	11.2067	-3.57836	1.7194	10.4799
31.0001	11.3509	-4.60902	-0.480112	10.3619
32.0001	11.4864	-4.44469	-2.74519	10.2296
33.	11.4152	-3.1508	-4.49071	10.0106
34.0004	11.2489	-1.03545	-5.35798	9.83655
35.0003	11.0622	1.42909	-5.09951	9.71206
36.0003	11.0042	3.5804	-3.80274	9.68568
37.0002	11.0466	4.92058	-1.66807	9.74846
38.0001	11.1395	4.9907	0.69343	9.93482
39.	11.1566	3.91009	2.65192	10.1069
40.0004	11.096	2.00432	3.71472	10.2618
41.0004	11.0693	-0.408258	3.72991	10.4139
42.0003	11.1264	-2.63829	2.61536	10.4879
43.0002	11.2738	-4.22864	0.652368	10.4303
44.0001	11.4063	-4.68763	-1.66285	10.2647
45.0001	11.4623	-3.94886	-3.71579	10.0987
46.	11.3262	-2.17415	-5.03622	9.90921
47.0004	11.1652	0.215088	-5.37588	9.78337
48.0003	11.0085	2.59615	-4.5627	9.67622
49.0003	11.0273	4.39757	-2.80226	9.71653
50.0002	11.0688	5.08689	-0.450856	9.82034

(0,0,-7.5) RMF off

```
Data = Import[NotebookDirectory[] <> "230223_x0_y0_z-7_5_field_off.txt", "Table"];
       importi··· Notebook-Verzeichnis                                Tabelle
```

```
Bvst = Table[{AbsoluteTime[Data[[i]][[9]]] - AbsoluteTime[Data[[2]][[9]]],
        Tabelle  absolute Zeit seit 1900                absolute Zeit seit 1900
    AbsoluteTime[Data[[i]][[2]]]}, {i, 2, Length[Data]}];
    absolute Zeit seit 1900                           Länge
Bxvst = Table[{AbsoluteTime[Data[[i]][[9]]] - AbsoluteTime[Data[[2]][[9]]],
         Tabelle  absolute Zeit seit 1900               absolute Zeit seit 1900
    AbsoluteTime[Data[[i]][[3]]]}, {i, 2, Length[Data]}];
    absolute Zeit seit 1900                           Länge
Byvst = Table[{AbsoluteTime[Data[[i]][[9]]] - AbsoluteTime[Data[[2]][[9]]],
         Tabelle  absolute Zeit seit 1900               absolute Zeit seit 1900
    AbsoluteTime[Data[[i]][[4]]]}, {i, 2, Length[Data]}];
    absolute Zeit seit 1900                           Länge
Bzvst = Table[{AbsoluteTime[Data[[i]][[9]]] - AbsoluteTime[Data[[2]][[9]]],
         Tabelle  absolute Zeit seit 1900               absolute Zeit seit 1900
    AbsoluteTime[Data[[i]][[5]]]}, {i, 2, Length[Data]}];
    absolute Zeit seit 1900                           Länge
```

```
ListPlot[{Bvst, Bxvst, Byvst, Bzvst}, Joined → True]
listenbezogene Graphik                    verknüpft? wahr
```

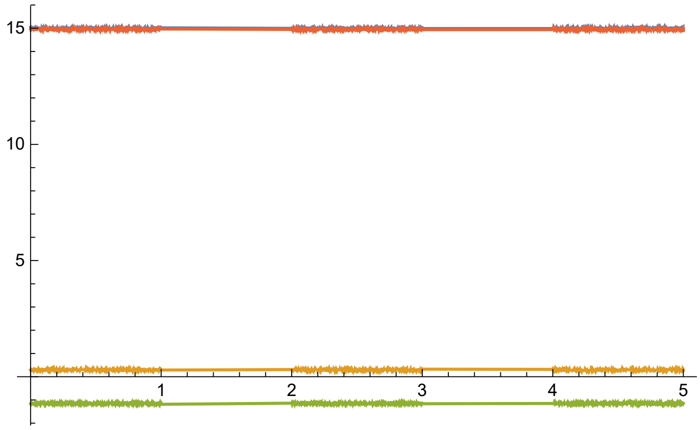


```
Start = 1;
Stop = 1000;
```

```
ListPlot[{Table[{Bvst[[i]][[1]], Bvst[[i]][[2]]}, {i, Start, Stop}],
  Table[{Bxvst[[i]][[1]], Bxvst[[i]][[2]]}, {i, Start, Stop}],
  Table[{Byvst[[i]][[1]], Byvst[[i]][[2]]}, {i, Start, Stop}],
  Table[{Bzvst[[i]][[1]], Bzvst[[i]][[2]]}, {i, Start, Stop}]}, Joined → True]
```

listenbezo··· Tabelle Tabelle Tabelle Tabelle verknüpft? wahr

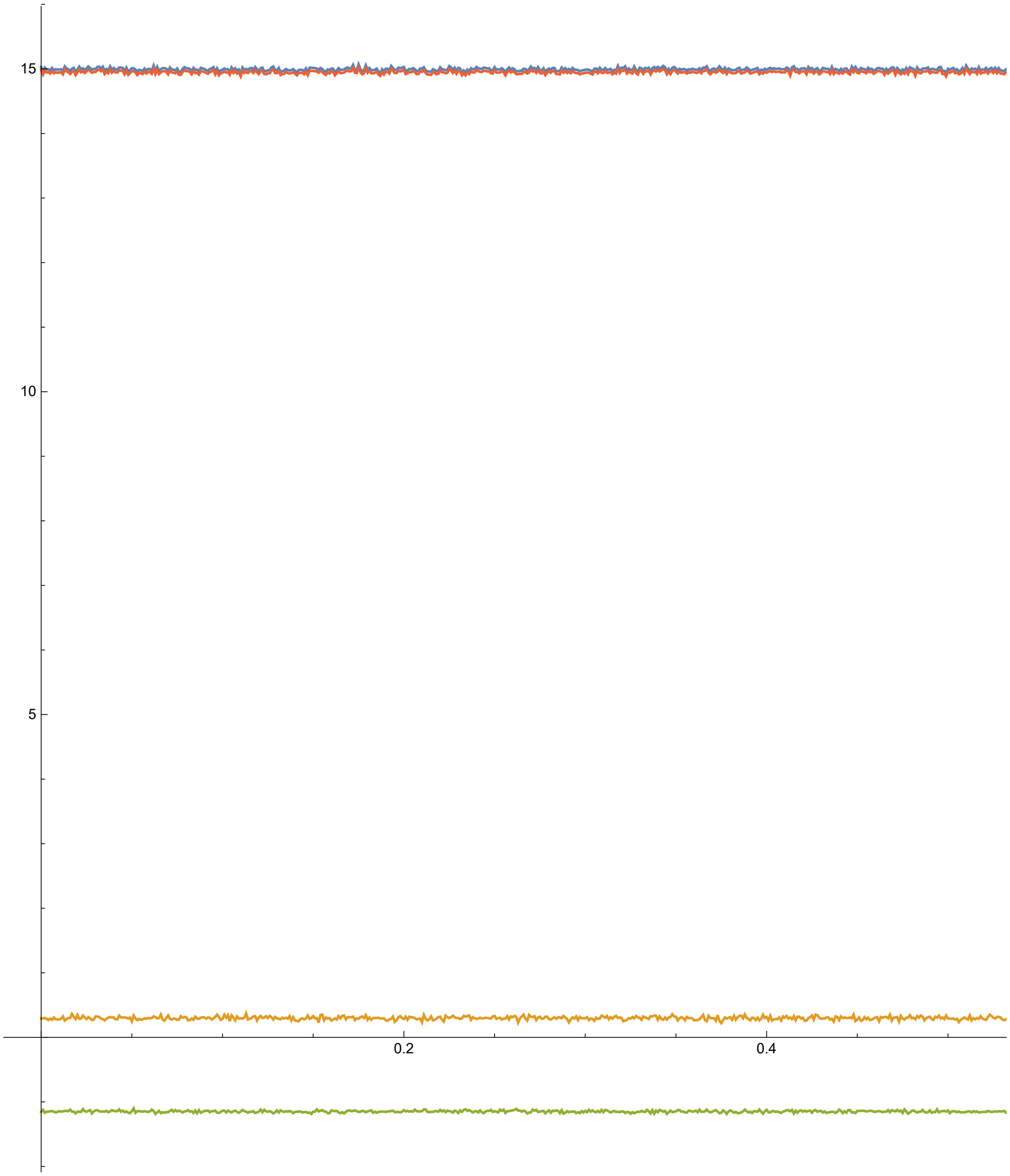

```
ListPlot[{Table[{Bvst[[i]][[1]], Bvst[[i]][[2]]}, {i, Start, Start + 50}],
 listenbezo…  Tabelle
  Table[{Bxvst[[i]][[1]], Bxvst[[i]][[2]]}, {i, Start, Start + 50}],
  Tabelle
  Table[{Byvst[[i]][[1]], Byvst[[i]][[2]]}, {i, Start, Start + 50}],
  Tabelle
  Table[{Bzvst[[i]][[1]], Bzvst[[i]][[2]]}, {i, Start, Start + 50}]},
  Tabelle
 Joined → True, PlotLegends → {"B", "Bx", "By", "Bz"}]
 verknüpft? wahr  Legenden der Graphik
```

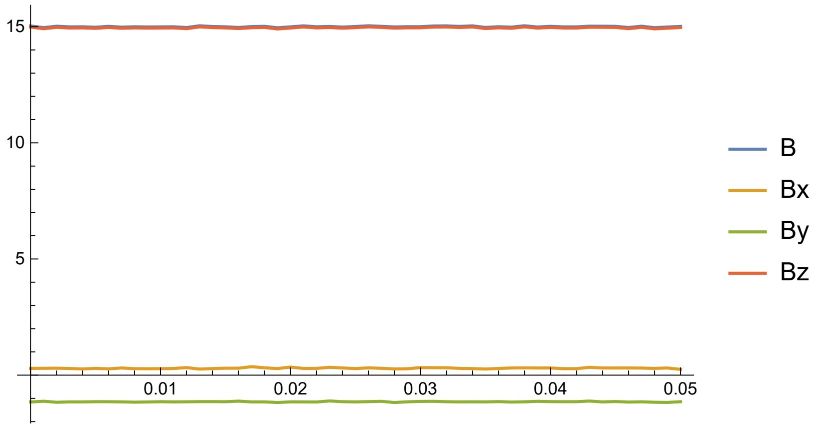


```
TableForm[{Mean[Table[Bvst[[i]][[2]], {i, Start, Stop}]],
Tabellendars… arit…  Tabelle
  Mean[Table[Bxvst[[i]][[2]], {i, Start, Stop}]],
  arit…  Tabelle
  Mean[Table[Byvst[[i]][[2]], {i, Start, Stop}]],
  arit…  Tabelle
  Mean[Table[Bzvst[[i]][[2]], {i, Start, Stop}]]}, TableDirections → Row]
  arit…  Tabelle                                  Richtung der Tabellen… Zeile
```

14.9958 0.296684 -1.14917 14.9487

```
TableForm[Table[{(Bvst[[i]][[1]] - Bvst[[Start]][[1]]) * 1000, Bvst[[i]][[2]],
   Bxvst[[i]][[2]], Byvst[[i]][[2]], Bzvst[[i]][[2]]}, {i, Start, Start + 50}]]
```

0.	15.0312	0.291613	-1.15576	14.9839
1.0004	14.9575	0.294838	-1.12266	14.9124
2.00033	15.0201	0.299502	-1.16757	14.9716
3.00026	14.991	0.287197	-1.15116	14.944
4.00019	14.9949	0.267239	-1.15124	14.9482
5.00011	14.9746	0.291024	-1.14291	14.9281
6.00004	15.0144	0.271437	-1.14354	14.9683
7.00045	14.9792	0.308072	-1.15095	14.9318
8.00037	14.9957	0.279251	-1.16323	14.9479
9.0003	14.9872	0.275161	-1.15511	14.9401
10.0002	14.9905	0.279189	-1.14317	14.9442
11.0002	14.995	0.287238	-1.15122	14.948
12.0001	14.9632	0.323902	-1.14669	14.9157
13.	15.038	0.260681	-1.13992	14.9925
14.0004	15.0062	0.28335	-1.13941	14.9602
15.0003	14.9946	0.299229	-1.14321	14.948
16.0003	14.9652	0.298916	-1.11878	14.9204
17.0002	15.0039	0.363317	-1.15125	14.9552
18.0001	15.0153	0.316443	-1.15149	14.9677
19.0001	14.9528	0.282813	-1.17755	14.9036
20.0005	14.9917	0.347194	-1.15109	14.9434
21.0004	15.0349	0.287651	-1.15183	14.988
22.0003	14.9953	0.291242	-1.15521	14.9479
23.0002	15.0091	0.336359	-1.11141	14.9641
24.0002	14.9827	0.308105	-1.14301	14.9359
25.0001	15.007	0.283362	-1.15141	14.96
26.	15.0384	0.312678	-1.13986	14.9919
27.0004	15.0136	0.295419	-1.12752	14.9683
28.0004	14.9886	0.267185	-1.17812	14.9398
29.0003	14.9987	0.275276	-1.1473	14.9522
30.0002	14.9982	0.324257	-1.13124	14.9519
31.0001	15.0298	0.320583	-1.12773	14.984
32.0001	15.0347	0.316641	-1.1438	14.9878
33.0005	15.011	0.291403	-1.15146	14.964
34.0004	15.0347	0.283647	-1.14784	14.9881
35.0003	14.9669	0.262951	-1.15082	14.9203
36.0003	14.9983	0.287267	-1.13928	14.9522
37.0002	14.9797	0.30808	-1.15895	14.9317
38.0001	15.0389	0.312686	-1.14786	14.9918
39.	14.9858	0.30813	-1.12708	14.9402
40.0004	15.0144	0.308431	-1.1395	14.968
41.0004	14.9905	0.279189	-1.14317	14.9442
42.0003	14.9905	0.279189	-1.14317	14.9442
43.0002	15.0213	0.336487	-1.11559	14.976
44.0001	15.0192	0.308485	-1.15156	14.9718
45.0001	15.0142	0.308427	-1.1355	14.968
46.	14.9638	0.307915	-1.15871	14.9157
47.0004	15.0189	0.304481	-1.14757	14.9719
48.0003	14.9561	0.290842	-1.1666	14.9077
49.0003	14.9847	0.308137	-1.17401	14.9354
50.0002	15.0102	0.248397	-1.14351	14.9645

(0,0,-7.5) RMF on

```
Data = Import[NotebookDirectory[] <> "230223_x0_y0_z-7_5_field_on_5mT.txt", "Table"];
```

```
Bvst = Table[{AbsoluteTime[Data[[i]][[9]]] - AbsoluteTime[Data[[2]][[9]]],
          Tabelle   absolute Zeit seit 1900                  absolute Zeit seit 1900
    AbsoluteTime[Data[[i]][[2]]]}, {i, 2, Length[Data]}];
    absolute Zeit seit 1900                           Länge
Bxvst = Table[{AbsoluteTime[Data[[i]][[9]]] - AbsoluteTime[Data[[2]][[9]]],
          Tabelle   absolute Zeit seit 1900                  absolute Zeit seit 1900
    AbsoluteTime[Data[[i]][[3]]]}, {i, 2, Length[Data]}];
    absolute Zeit seit 1900                           Länge
Byvst = Table[{AbsoluteTime[Data[[i]][[9]]] - AbsoluteTime[Data[[2]][[9]]],
          Tabelle   absolute Zeit seit 1900                  absolute Zeit seit 1900
    AbsoluteTime[Data[[i]][[4]]]}, {i, 2, Length[Data]}];
    absolute Zeit seit 1900                           Länge
Bzvst = Table[{AbsoluteTime[Data[[i]][[9]]] - AbsoluteTime[Data[[2]][[9]]],
          Tabelle   absolute Zeit seit 1900                  absolute Zeit seit 1900
    AbsoluteTime[Data[[i]][[5]]]}, {i, 2, Length[Data]}];
    absolute Zeit seit 1900                           Länge
```

```
ListPlot[{Bvst, Bxvst, Byvst, Bzvst}, Joined → True]
listenbezogene Graphik                 verknüpft? wahr
```

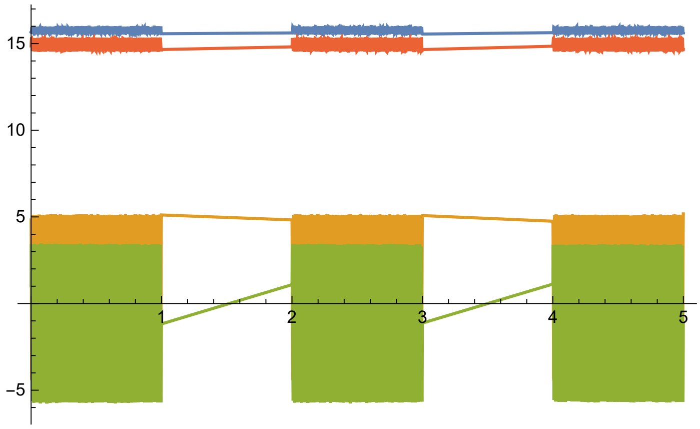


```
Start = 1;
Stop = 1000;
```

```
ListPlot[{Table[{Bvst[[i]][[1]], Bvst[[i]][[2]]}, {i, Start, Stop}],
   Table[{Bxvst[[i]][[1]], Bxvst[[i]][[2]]}, {i, Start, Stop}],
   Table[{Byvst[[i]][[1]], Byvst[[i]][[2]]}, {i, Start, Stop}],
   Table[{Bzvst[[i]][[1]], Bzvst[[i]][[2]]}, {i, Start, Stop}]}, Joined → True]
```

listenbezo··· Tabelle Tabelle Tabelle Tabelle verknüpft? wahr

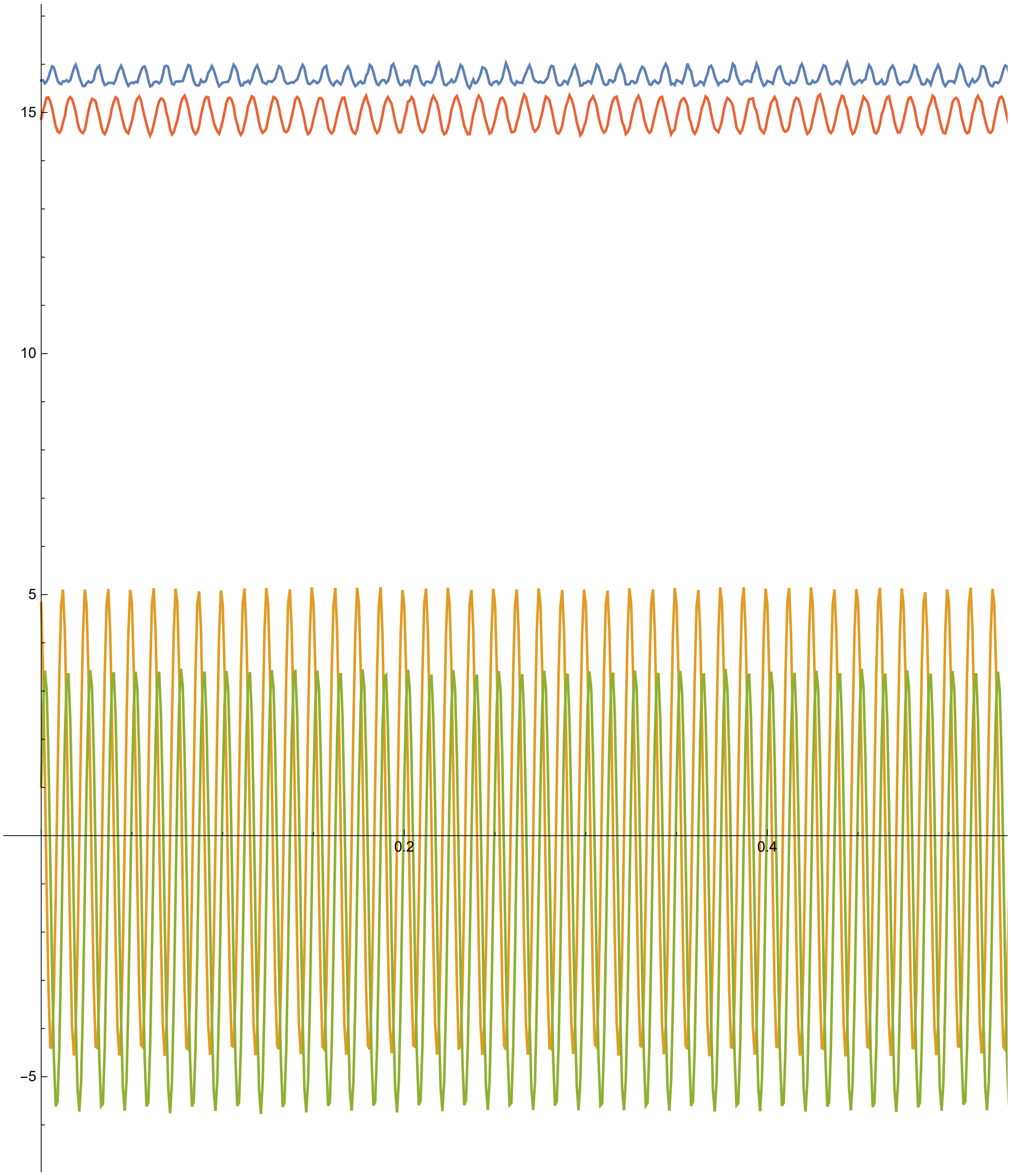

```
ListPlot[{Table[{Bvst[[i]][[1]], Bvst[[i]][[2]]}, {i, Start, Start + 50}],
listenbezo⋯  Tabelle
   Table[{Bxvst[[i]][[1]], Bxvst[[i]][[2]]}, {i, Start, Start + 50}],
   Tabelle
   Table[{Byvst[[i]][[1]], Byvst[[i]][[2]]}, {i, Start, Start + 50}],
   Tabelle
   Table[{Bzvst[[i]][[1]], Bzvst[[i]][[2]]}, {i, Start, Start + 50}]},
   Tabelle
 Joined → True, PlotLegends → {"B", "Bx", "By", "Bz"}]
 verknüpft? wahr  Legenden der Graphik
```

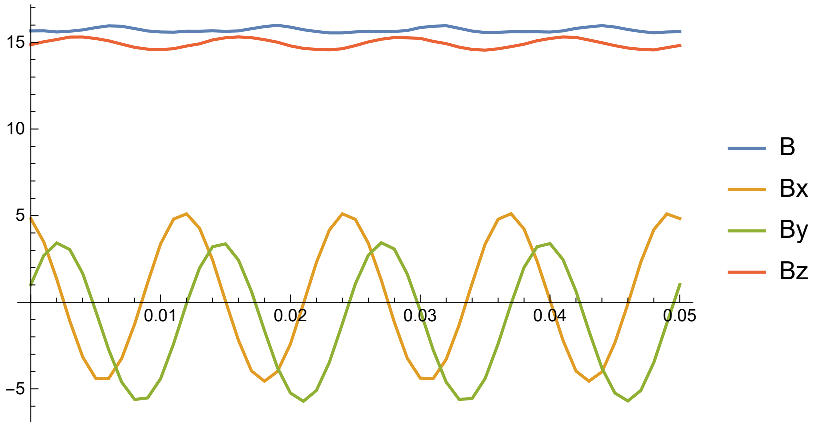


```
TableForm[{Mean[Table[Bvst[[i]][[2]], {i, Start, Stop}]],
Tabellendars⋯ arit⋯  Tabelle
   Mean[Table[Bxvst[[i]][[2]], {i, Start, Stop}]],
   arit⋯  Tabelle
   Mean[Table[Byvst[[i]][[2]], {i, Start, Stop}]],
   arit⋯  Tabelle
   Mean[Table[Bzvst[[i]][[2]], {i, Start, Stop}]]}, TableDirections → Row]
   arit⋯  Tabelle                                  Richtung der Tabellen⋯ Zeile
```

15.7209 0.297276 −1.14272 14.9452

```
TableForm[Table[{(Bvst[[i]][[1]] - Bvst[[Start]][[1]]) * 1000, Bvst[[i]][[2]],
Tabellendar·· Tabelle
   Bxvst[[i]][[2]], Byvst[[i]][[2]], Bzvst[[i]][[2]]}, {i, Start, Start + 50}]]
```

0.	15.6637	4.82307	1.02393	14.8674
0.999928	15.6701	3.48077	2.70115	15.0379
1.99986	15.6033	1.32512	3.42005	15.1661
2.99978	15.645	-1.07611	3.04437	15.3082
3.99971	15.7233	-3.16253	1.65799	15.3125
4.99964	15.8539	-4.39193	-0.499193	15.2252
5.99957	15.9566	-4.39865	-2.73743	15.0921
6.99997	15.932	-3.24734	-4.61352	14.8996
7.9999	15.7985	-1.26196	-5.61276	14.7138
8.99982	15.6609	1.123	-5.52572	14.6105
9.99975	15.6037	3.3735	-4.41382	14.5813
10.9997	15.5896	4.80076	-2.40096	14.6363
11.9996	15.6471	5.10161	-0.068753	14.7919
12.9995	15.6443	4.27444	1.9767	14.9186
13.9999	15.6706	2.4482	3.20253	15.1432
14.9999	15.637	0.07117	3.37018	15.2693
15.9998	15.666	-2.20233	2.43298	15.3184
16.9997	15.7914	-3.96375	0.625649	15.273
17.9996	15.9124	-4.55829	-1.63546	15.1576
18.9996	15.9883	-4.00122	-3.77492	15.0122
20.	15.8832	-2.4136	-5.24019	14.7984
20.9999	15.7329	-0.091367	-5.71969	14.6561
21.9998	15.629	2.28641	-5.09526	14.5971
22.9998	15.5488	4.17032	-3.47074	14.5715
23.9997	15.5498	5.10539	-1.24649	14.6348
24.9996	15.6057	4.78651	1.05168	14.8162
25.9995	15.6465	3.41263	2.71325	15.0268
27.	15.6193	1.28127	3.43575	15.1828
27.9999	15.6293	-1.13942	3.07572	15.2813
28.9998	15.6882	-3.22698	1.63164	15.2658
29.9997	15.8578	-4.37988	-0.475304	15.2335
30.9997	15.9312	-4.40696	-2.75295	15.06
31.9996	15.967	-3.29806	-4.60601	14.9282
33.	15.8124	-1.30584	-5.60899	14.7263
33.9999	15.6561	1.09088	-5.55852	14.5954
34.9998	15.5702	3.35319	-4.40541	14.5526
35.9998	15.5826	4.78872	-2.40391	14.6324
36.9997	15.6165	5.11326	-0.095186	14.7553
37.9996	15.6143	4.22617	2.00899	14.8966
38.9996	15.6161	2.38472	3.20318	15.0969
40.	15.5987	0.110708	3.38688	15.2262
40.9999	15.6668	-2.19841	2.46404	15.3149
41.9998	15.8095	-3.9716	0.637394	15.2893
42.9997	15.8955	-4.55847	-1.65916	15.1372
43.9997	15.9686	-4.01752	-3.80245	14.9799
44.9996	15.8874	-2.33743	-5.24435	14.8135
46.	15.745	-0.09118	-5.70099	14.6764
46.9999	15.6327	2.32241	-5.09122	14.5968
47.9999	15.5521	4.19429	-3.47465	14.5672
48.9998	15.602	5.09797	-1.21642	14.6954
49.9997	15.6239	4.83062	1.02062	14.8233
